# Naked Singularities in the Einstein-Euler System

**DOI:** 10.1007/s40818-022-00144-3

**Published:** 2023-02-07

**Authors:** Yan Guo, Mahir Hadzic, Juhi Jang

**Affiliations:** 1 Division of Applied Mathematics, Brown University, Providence, RI 02912 USA; 2 Department of Mathematics, University College London, London, UK; 3 Department of Mathematics, University of Southern California, Los Angeles, CA 90089 USA; 4 Korea Institute for Advanced Study, Seoul, Korea

**Keywords:** Self-similar solutions, Einstein-Euler system, Implosion, Naked singularities

## Abstract

In 1990, based on numerical and formal asymptotic analysis, Ori and Piran predicted the existence of selfsimilar spacetimes, called relativistic Larson-Penston solutions, that can be suitably flattened to obtain examples of spacetimes that dynamically form naked singularities from smooth initial data, and solve the radially symmetric Einstein-Euler system. Despite its importance, a rigorous proof of the existence of such spacetimes has remained elusive, in part due to the complications associated with the analysis across the so-called sonic hypersurface. We provide a rigorous mathematical proof. Our strategy is based on a delicate study of nonlinear invariances associated with the underlying non-autonomous dynamical system to which the problem reduces after a selfsimilar reduction. Key technical ingredients are a monotonicity lemma tailored to the problem, an ad hoc shooting method developed to construct a solution connecting the sonic hypersurface to the so-called Friedmann solution, and a nonlinear argument to construct the maximal analytic extension of the solution. Finally, we reformulate the problem in double-null gauge to flatten the selfsimilar profile and thus obtain an asymptotically flat spacetime with an isolated naked singularity.

## Introduction

We study the Einstein-Euler system which couples the Einstein field equations to the Euler equations of fluid mechanics. The unknowns are the 4-dimensional Lorentzian spacetime $(\mathcal {M}, g)$, the fluid pressure *p*, the mass-density $\rho$, and the 4-velocity $u^\alpha$. In an arbitrary coordinate system, the Einstein-Euler equations read

1.1 $$\begin{aligned} \text {Ric}_{\alpha \beta }-\frac{1}{2}\mathcal {R}g_{\alpha \beta }&=T_{\alpha \beta }, \quad (\alpha ,\,\beta =\,0,1,2,3), \end{aligned}$$

1.2 $$\begin{aligned} \nabla _{\alpha }T^{\alpha \beta }&=0, \quad (\beta =0,1,2,3), \end{aligned}$$

1.3 $$\begin{aligned} g_{\alpha \beta } u^{\alpha } u^{\beta }&=-1,\, \end{aligned}$$

where $\text {Ric}_{\alpha \beta }$ is the Ricci curvature tensor, $\mathcal {R}$ the scalar curvature of $g_{\alpha \beta }$, and $T_{\alpha \beta }$ is the energy momentum tensor given by the formula

1.4 $$\begin{aligned} T_{\alpha \beta } = (\rho +p)u_{\alpha }u_{\beta } + p g_{\alpha \beta }, \quad ( \alpha , \, \beta =0,1,2,3). \end{aligned}$$

To close the system, we assume the linear equation of state

1.5 $$\begin{aligned} p = \varepsilon \rho , \end{aligned}$$

where $0<\varepsilon <1$ corresponds to the square of the speed of sound.

The system (1.1)–(1.5) is a fundamental model of a selfgravitating relativistic gas. We are interested in the existence of selfsimilar solutions to (1.1)–(1.5) under the assumption of radial symmetry. This amounts to the existence of a homothetic Killing vector field $\xi$ with the property

1.6 $$\begin{aligned} \mathcal {L}_\xi g = 2 g, \end{aligned}$$

where the left-hand side is the Lie derivative of the metric *g*. The presence of such a vector field induces a scaling symmetry, which allows us to look for selfsimilar solutions to (1.1)–(1.5). Study of selfsimilar solutions to Einstein-matter systems has a rich history in the physics literature. They in particular provide a way of constructing spacetimes with so-called naked singularities, a notion intimately tied to the validity of the weak cosmic censorship of Penrose [30], see the discussion in [7, 10, 32]. Naked singularities intuitively correspond to spacetime singularities that are “visible" to far away observers, which informally means that there exists a future outgoing null-geodesic “emanating" from the singularity and reaching the asymptotically flat region of the spacetime. We adopt here a precise mathematical definition from the work of Rodnianski and Shlapentokh-Rothman [32, Definition 1.1], which in turn is related to a formulation of weak cosmic censorship by Christodoulou [7].

In the absence of pressure ($\varepsilon =0$ in (1.5)) and under the assumption of radial symmetry, the problem simplifies considerably. The corresponding family of solutions was studied by Lemaître [21] and Tolman [34] in early 1930s (see also [1]). In their seminal work from 1939, Oppenheimer and Snyder [25] studied the causal structure of a subclass of Lemaître-Tolman solutions with space-homogeneous densities, thus exhibiting the first example of a dynamically forming (what later became known as) black hole. However, in 1984 Christodoulou [5] showed that, within the larger class of Lemaître-Tolman solutions with space-inhomogeneous densities, black holes are exceptional and instead naked singularities form generically.

Of course, in the context of astrophysics, one expects the role of pressure to be very important in the process of gravitational collapse for relativistic gases. In the late stages of collapse, the core region is expected to be very dense and the linear equation of state (1.5) is commonly used in such a setting, as it is compatible with the requirement that the speed of sound is smaller than the speed of light, $\sqrt{\varepsilon }<1$. In their pioneering works, Ori and Piran [26–28] found numerically selfsimilar solutions to (1.1)–(1.5), which are the relativistic analogues of the Larson-Penston selfsimilar collapsing solutions to the isothermal Euler-Poisson system, see [15, 20, 31]. Through both numerical and asymptotic analysis methods Ori and Piran investigated the causal structure of such relativistic Larson-Penston solutions, ascertaining the existence of spacetimes with naked singularities when $\varepsilon$ is smaller than a certain value. Our main goal is to justify the findings of Ori and Piran on rigorous mathematical grounds.

Broadly speaking, this manuscript consists of two parts. In the first part, which constitutes the bulk of our work, we construct a selfsimilar solution of the Einstein-Euler system (Sections 3–8), assuming that $\varepsilon$ – the square of the speed of sound – is sufficiently small.

### Theorem 1.1

(Existence of the relativistic Larson-Penston spacetimes) For any sufficiently small $0<\varepsilon \ll 1$ there exists a radially symmetric real-analytic selfsimilar solution to the Einstein-Euler system with a curvature singularity at the scaling origin and an outgoing null-geodesic emanating from it all the way to infinity. The resulting spacetime is called the relativistic Larson-Penston (RLP) spacetime.

It is not hard to see that the selfsimilar solution constructed in Theorem 1.1 is not asymptotically flat. In the second step (Section 9), using PDE techniques, we flatten the selfsimilar RLP-profile in a region away from the singularity and thus obtain an asymptotically flat solution with a naked singularity. Thus, our main theorem states that in the presence of pressure there do exist examples of naked singularities which form from smooth data.

### Theorem 1.2

(Existence of naked singularities) For sufficiently small $0<\varepsilon \ll 1$ there exist radially symmetric asymptotically flat solutions to the Einstein-Euler system that form a naked singularity in the sense of [32, Definition 1.1].

Other than the dust-Einstein model mentioned above, we are aware of two other rigorous results on the existence of naked singularities. In 1994 Christodoulou [6] provided a rigorous proof of the existence of radially symmetric solutions to the Einstein-scalar field system, which contain naked singularities (see also [8] for the proof of their instability). Very recently, Rodnianski and Shlapentokh-Rothman [32] proved the existence of solutions to the Einstein-vacuum equations which contain naked singularities and are (necessarily) not radially symmetric.

In the physics literature much attention has been given to selfsimilar solutions and naked singularities for the Einstein-Euler system, see for example [2, 14]. A selfsimilar reduction of the problem was first given in [33]. As explained above, a detailed analysis of the resulting equations, including the discussion of naked singularities, was given in [26–28]. Subsequent to [28], a further analysis of the causal structure, including the nonradial null-geodesics was presented in [19], see also [4]. There exist various approaches to the existence of solutions to the selfsimilar problem, most of them rely on numerics [3, 17, 28]. A dynamical systems approach with a discussion of some qualitative properties of the solutions was developed in [3, 13]. Numerical investigation of the stability of the RLP-spacetimes can be found in [17, 18]. selfsimilar relativistic perfect fluids play an important role in the study of the so-called critical phenomena - we refer to reviews [14, 24].

The proof of Theorem 1.1 relies on a careful study of the nonlinear invariances of the finite-dimensional non-autonomous dynamical system obtained through the selfsimilar reduction. The solutions we construct are real-analytic in a suitable choice of coordinates. A special role is played by the so-called sonic line (sonic point), the boundary of the backward sound cone emanating from the scaling origin $\mathcal {O}$. Many difficulties in the proof of Theorem 1.1 originate from possible singularities across this line, which together with the requirement of smoothness, puts severe limitations on the possible space of smooth selfsimilar solutions. We are aware of no general ODE theory for global existence in the presence of singular sonic points. Therefore, our proofs are all based on continuity arguments, where we extract many delicate invariant properties of the nonlinear flow, specific to the ODE system at hand. In particular, the discovery of a crucial monotonicity lemma enables us to apply an ad hoc shooting method, which was developed for the limiting Larson-Penston solution in the non-relativistic context by the authors. From the point of view of fluid mechanics, the singularity at $\mathcal {O}$ is an imploding one, as the energy density blows up on approach to $\mathcal {O}$. It is in particular not a shock singularity.

The asymptotic flattening in Theorem 1.2 requires solving a suitable characteristic problem for the Einstein-Euler system formulated in the double-null gauge. We do this in a semi-infinite characteristic rectangular domain wherefrom the resulting solution can be glued smoothly to the exact selfsimilar solution in the region around the singularity $\mathcal {O}$. The proof of Theorem 1.2 is given in Section 9.6.

Due to the complexity of our analysis, in Section 2 we give an extensive overview of our methods and key ideas behind the detailed proofs in Sections 3–9.

## Methodology and Outline

### Formulation of the Problem (Section 3)

Following [28] it is convenient to work with the comoving coordinates

2.7 $$\begin{aligned} g = -e^{2\mu (\tau , R)} d\tau ^2 + e^{2\lambda (\tau ,R)}dR^2 + r^2(\tau ,R) \,\gamma , \end{aligned}$$

where $\gamma = \gamma _{AB}dx^A\,dx^B$ is the standard metric on $\mathbb S^2$, $x^A$, $A=2,3$ are local coordinates on $\mathbb S^2$, and *r* is the areal radius. The vector field $\partial _\tau$ is chosen in such a way that the four velocity $u^\nu$ is parallel to $\partial _\tau$. The normalisation condition (1.3) then implies

2.8 $$\begin{aligned} u = e^{-\mu }\partial _\tau . \end{aligned}$$

The coordinate *R* acts as a particle label. The coordinates $(\tau , R)$ are then uniquely determined by fixing the remaining gauge freedoms in the problem, the value of $\mu (\tau ,R)\big |_{R=0}$ and by setting $r(-1, R) = R$, which states that on the hypersurface $\tau =-1$ the comoving label *R* coincides with the areal radius.

Introduce the radial velocity

2.9 $$\begin{aligned} \mathcal {V} : = e^{-\mu } \partial _\tau r, \end{aligned}$$

the Hawking (also known as Misner-Sharp) mass

2.10 $$\begin{aligned} m(\tau ,R){:}{=} 4\pi \int _0^{r(\tau ,R)} \rho s^2 \,ds = 4\pi \int _0^R \rho r^2 \partial _Rr\,d\bar{R}, \end{aligned}$$

and the mean density

2.11 $$\begin{aligned} G(\tau ,R): = \frac{m(\tau ,R)}{\frac{4\pi }{3}r(\tau ,R)^3} = \frac{3}{r(\tau ,R)^3} \int _0^{R} \rho (\tau , \bar{R}) r(\tau ,\bar{R})^2 \partial _Rr(\tau ,\bar{R})\,d\bar{R} . \end{aligned}$$

Recalling the equation of state (1.5), the spherically symmetric Einstein-Euler system in comoving coordinates reads (see [12, 23])

2.12 $$\begin{aligned} \partial _\tau \rho + (1+\varepsilon )\rho \left( \frac{\partial _R\mathcal {V}}{\partial _Rr}+2\frac{\mathcal {V}}{r}\right) e^\mu&=0, \end{aligned}$$

2.13 $$\begin{aligned} \partial _\tau \lambda&= e^\mu \frac{\partial _R{\mathcal {V}}}{\partial _Rr}, \end{aligned}$$

2.14 $$\begin{aligned} e^{-\mu }\partial _\tau \mathcal {V} + \frac{\varepsilon }{1+\varepsilon }\frac{\partial _Rr e^{-2\lambda }}{\rho } \partial _R\rho + 4\pi r \left( \frac{1}{3} G+\varepsilon \rho \right)&=0, \end{aligned}$$

2.15 $$\begin{aligned} (\partial _Rr)^2 e^{-2\lambda }&= 1+ \mathcal {V}^2 - \frac{8\pi }{3} G r^2, \end{aligned}$$

where we recall (2.11). The well-known Tolman-Oppenheimer-Volkov relation reads $(\rho +p) \partial _R\mu + \partial _Rp=0$, which after plugging in (1.5) further gives the relation

2.16 $$\begin{aligned} \partial _R\mu = - \frac{\varepsilon }{1+\varepsilon }\frac{\partial _R\rho }{\rho }. \end{aligned}$$

#### Comoving Selfsimilar Formulation

It is straightforward to check that the system (2.12)–(2.15) is invariant under the scaling transformation

2.17 $$\begin{aligned} \rho \mapsto a^{-2}\rho (s,y), \quad r\mapsto a r(s,y), \quad \mathcal {V} \mapsto \mathcal {V}(s,y), \quad \lambda \mapsto \lambda (s,y), \quad \mu \mapsto \mu (s,y), \end{aligned}$$

where the comoving “time" $\tau$ and the particle label *R* scale according to

2.18 $$\begin{aligned} s = \frac{\tau }{a}, \quad y = \frac{R}{a}, \quad a>0. \end{aligned}$$

Motivated by the scaling invariance (2.17)–(2.18), we look for selfsimilar spacetimes of the form

2.19 $$\begin{aligned} \rho (\tau ,R)&= \frac{1}{2\pi \tau ^2} \Sigma (y), \end{aligned}$$

2.20 $$\begin{aligned} r(\tau , R)&= -\sqrt{\varepsilon }\tau \tilde{r}(y) , \end{aligned}$$

2.21 $$\begin{aligned} \mathcal {V}(\tau ,R)&= \sqrt{\varepsilon }V(y) , \end{aligned}$$

2.22 $$\begin{aligned} \lambda (\tau ,R)&= \lambda (y), \end{aligned}$$

2.23 $$\begin{aligned} \mu (\tau ,R)&= \mu (y), \end{aligned}$$

2.24 $$\begin{aligned} G(\tau , R)&= \frac{1}{4\pi \tau ^2} \tilde{G}(y) = \frac{3}{2\pi \tau ^2\tilde{r}^3}\int _0^y \Sigma (\tilde{y}) \tilde{r}^2 \tilde{r}' \,d\tilde{y}, \end{aligned}$$

where

2.25 $$\begin{aligned} y= \frac{R}{-\sqrt{\varepsilon }\tau }. \end{aligned}$$

Associated with the comoving selfsimilar coordinates are the two fundamental unknowns:

2.26 $$\begin{aligned} \textbf{d}&: = \Sigma ^{\frac{1-\varepsilon }{1+\varepsilon }}, \end{aligned}$$

2.27 $$\begin{aligned} \textbf{w}&: = (1+\varepsilon ) \frac{e^\mu V + \tilde{r} }{\tilde{r}} - \varepsilon . \end{aligned}$$

For future use it is convenient to sometimes consider the quantity

2.28 $$\begin{aligned} {\varvec{\chi }}(y): = \frac{\tilde{r}(y)}{y}, \end{aligned}$$

instead of $\tilde{r}$. Quantity $\textbf{d}$ corresponds to the selfsimilar number density, while $\textbf{w}$ is referred to as the relative velocity. It is shown in Proposition 3.4 that the radial Einstein-Euler system under the selfsimilar ansatz above reduces to the following system of ODE:

2.29 $$\begin{aligned} \textbf{d}'&= - \frac{ 2(1-\varepsilon ) \textbf{d}(\textbf{d}-\textbf{w})}{(1+\varepsilon )y(e^{2\mu - 2\lambda } y^{-2} -1)} , \end{aligned}$$

2.30 $$\begin{aligned} \textbf{w}'&= \frac{(\textbf{w}+\varepsilon )(1 -3\textbf{w})}{(1+\varepsilon )y} + \frac{2\textbf{w}(\textbf{d}-\textbf{w})}{y(e^{2\mu - 2\lambda } y^{-2} -1)}. \end{aligned}$$

This formulation of the selfsimilar problem highlights the danger from possible singularities associated with the vanishing of the denominators on the right-hand side of (2.29)–(2.30). Such points play a distinguished role in our analysis, and as we shall show shortly, are unavoidable in the study of physically interesting selfsimilar solutions.

##### Definition 2.1

(The sonic point) For any smooth solution to (2.29)–(2.30) we refer to a point $y_*\in (0,\infty )$ satisfying

2.31 $$\begin{aligned} y_*^2 = e^{2\mu (y_*)-2\lambda (y_*)} \end{aligned}$$

as the *sonic point*.

#### Schwarzschild Selfsimilar Formulation

The comoving formulation (2.29)–(2.30) as written does not form a closed system of ODE. To do so, we must express the metric coefficients $\mu ,\lambda$ as functions of $\textbf{d},\textbf{w}$, which can be done at the expense of working with $\tilde{r}$ (or equivalently ${\varvec{\chi }}$) as a further unknown. To avoid this, it is possible to introduce the so-called Schwarzschild selfsimilar coordinate:

2.32 $$\begin{aligned} x{:}{=} \tilde{r} (y) \end{aligned}$$

so that

2.33 $$\begin{aligned} \frac{dx}{dy} = \tilde{r} ' = \frac{x (\textbf{w}+\varepsilon )}{y(1+\varepsilon )}. \end{aligned}$$

In this coordinate system the problem takes on a form analogous to the Eulerian formulation of the selfsimilar Euler-Poisson system from [15]. It is shown in Lemma 3.5 that the new unknowns

2.34 $$\begin{aligned} D(x){:}{=}\,\textbf{d}(y), \quad W(x){:}{=}\,\textbf{w}(y), \end{aligned}$$

solve the system

2.35 $$\begin{aligned} D'(x)&= - \frac{ 2x(1-\varepsilon ) D(W + \varepsilon ) (D-W) }{B}, \end{aligned}$$

2.36 $$\begin{aligned} W'(x)&= \frac{(1 -3W )}{x} + \frac{2x(1+\varepsilon ) W (W + \varepsilon ) (D-W)}{B}, \end{aligned}$$

where

2.37 $$\begin{aligned} B=B[x;D,W] : = D^{-\eta } -\left[ ( W + \varepsilon )^2 - \varepsilon (W - 1)^2 + 4\varepsilon DW \right] x^2, \end{aligned}$$

and

2.38 $$\begin{aligned} \eta {:}{=}\frac{2\varepsilon }{1-\varepsilon }. \end{aligned}$$

The Greek letter $\eta$ will always be used to mean (2.38) in the rest of the paper. In this formulation, sonic points correspond to zeroes of $B=B[x;D,W]$, i.e. if $y_*$ is a sonic point in the sense of Definition 2.1, then $x_*{:}{=}\tilde{r}(y_*)$ is a zero of the denominator *B*.

#### Friedmann, Far-Field, and the Necessity of the Sonic Point

There are two exact solutions to (2.35)–(2.36). The far-field solution

2.39 $$\begin{aligned} D_f(x) = (1-\varepsilon )^{-\frac{2}{1+\eta }} x^{-\frac{2}{1+\eta }}, \quad W_f(x) = 1, \end{aligned}$$

features a density $D$ that blows up at $x=0$ and decays to 0 as $x\rightarrow \infty$. On the other hand, the Friedmann solution

2.40 $$\begin{aligned} D_F(x) = \frac{1}{3}, \quad W_F(x) = \frac{1}{3}, \end{aligned}$$

is bounded at $x=0$, but the density does not decay as $x\rightarrow \infty$. Our goal is to construct a smooth solution to (2.35)-(2.36) which qualitatively behaves like the far-field solution as $x\rightarrow \infty$ and like the Friedmann solution as $x\rightarrow 0^+$. We can therefore think of it as a heteroclinic orbit for the dynamical system (2.35)-(2.36). It is then easy to see that any such solution has the property $\lim _{x\rightarrow \infty }B=-\infty$ and $\lim _{x\rightarrow 0^+}B(x)>0$. By the intermediate value theorem there must exist a point where *B* vanishes, i.e. a sonic point.

It is important to understand the formal Newtonian limit, which is obtained by letting $\varepsilon =0$ in (2.35)–(2.36). This yields the system

2.41 $$\begin{aligned} \tilde{D}'(x)&= - \frac{ 2x \tilde{D} \tilde{W} (\tilde{D}-\tilde{W}) }{1-x^2\tilde{W}^2} , \end{aligned}$$

2.42 $$\begin{aligned} \tilde{W}'(x)&= \frac{(1 -3\tilde{W} )}{x} + \frac{2x \tilde{W}^2 (\tilde{D}-\tilde{W})}{1-x^2 \tilde{W}^2}, \end{aligned}$$

which is precisely the selfsimilar formulation of the isothermal Euler-Poisson system. In [15] we showed that there exists a solution to (2.41)–(2.42) satisfying $\tilde{W}(0)=\frac{1}{3}$, $\lim _{x\rightarrow \infty }\tilde{W}(x)=1$, $\tilde{D}(0)>\frac{1}{3}$, and $\tilde{D}\asymp _{x\rightarrow \infty }x^{-2}$.^1^ The behaviour of the relativistic solutions when $0<\varepsilon \ll 1$ in the region $x\in [0,\infty )$ is modelled on this solution, called the Larson-Penston (LP) solution.

The system (2.35)–(2.37) is a non-autonomous $2\times 2$ system of ODE which is at the heart of the proof of Theorem 1.1 and is used to show the existence of the RLP-solution in the region $x\in [0,\infty )$. As $x\rightarrow \infty$, we are forced to switch back to a version of the comoving variables in order to extend the solution beyond $x=\infty$ in a unique way, see the discussion in Section 2.5. A version of the comoving formulation (2.29)–(2.30) plays a crucial role in that extension. In our analysis of the radial null-geodesics (Section 8) and the nonradial ones (Appendix B), we often switch between different choices of coordinates to facilitate our calculations.

Even though the smallness of $\varepsilon$ is crucial for the validity of our estimates, we emphasise that we do not use perturbation theory to construct the relativistic solution, by for example perturbing away from the Newtonian one. Such an argument is a priori challenging due to the singular nature of the sonic point, as well as complications arising from boundary conditions.

We mention that selfsimilar imploding flows for the compressible Euler system, featuring a sonic point, were constructed recently in the pioneering work of Merle, Raphaël, Rodnianski, and Szeftel [22] - here the associated $2\times 2$ dynamical system is autonomous. In the context of the Euler-Poisson system with polytropic gas law, selfsimilar collapsing solutions featuring a sonic point were recently constructed in [16].

### The Sonic Point Analysis (Section 4)

The solution we are trying to construct is on one hand assumed to be smooth, but it also must feature an a priori unknown sonic point, which we name $x_*$. This is a singular point for the dynamical system and the assumption of smoothness therefore imposes a hierarchy of constraints on the Taylor coefficients of a solution in a neighbourhood of $x_*$. More precisely, we look for solutions $(D,W)$ to (2.35)-(2.36) of the form

2.43 $$\begin{aligned} D= \sum _{N=0}^\infty D_N (x-x_*)^N, \quad W = \sum _{N=0}^\infty W_N (x-x_*)^N. \end{aligned}$$

It is clear that the first constraint reads $D_0=W_0$, as the numerator in (2.35) must vanish at $x_*$ for the solution to be smooth. Together with the condition $B(x_*)=0$, we can show that for any $\varepsilon >0$ sufficiently small, $D_0=W_0$ is a function of $x_*$, which converges to $\frac{1}{x_*}$ as $\varepsilon \rightarrow 0$ in accordance with the limiting Newtonian problem (2.41)–(2.42). Our goal is to express $D_N, W_N$ recursively in terms of $D_0,\dots , D_{N-1}$, $W_0,\dots , W_{N-1}$ and thus obtain a hierarchy of algebraic relations that allows to compute the Taylor coefficients up to an arbitrary order.

However, an intriguing dichotomy emerges. At the next order, one obtains a cubic equation for $W_1$, see Lemma 4.7. One of the solutions is a “ghost" solution and therefore unphysical, while the remaining two roots, when real, are both viable candidates for $W_1$, given as a function of $D_0$ (and therefore $x_*$). This is related to an analogous dichotomy in the Newtonian case - where one choice of the root leads to so-called Larson-Penston-type (LP type) solutions, while the other choice of the root leads to Hunter-type solutions. Based on this, for any $0<\varepsilon \ll 1$ sufficiently small, we select $W_1=W_1(\varepsilon )$ to correspond to the choice of the branch converging to the LP-type coefficient as $\varepsilon \rightarrow 0$. This is a de facto selection principle which allows us to introduce formal Taylor expansions of the relativistic Larson-Penston-type (RLP type), see Definition 4.11. Upon fixing the choice of $W_1$ (and thereby $D_1$), all the higher-order coefficients $(D_N,W_N)$, $N\ge 2$, are then uniquely determined through a recursive relation, see Section 4.3. We mention that $D_1$ and $W_1$ cease to exist as real numbers before $x_*$ reaches 2 from above, which led us to define $x_{\text {crit}}(\varepsilon )$, see Lemma 4.10. This should be contrasted to the Newtonian problem where $D_1$ and $W_1$ are real-valued as $x_*$ passes below 2^2^. The existence of a forbidden range for $x_*$ is related to the band structure in the space of all smooth solutions, see [13, 28].

Guided by the intuition developed in the construction of the (non-relativistic) Larson-Penston solution [15], our next goal is to identify the so-called sonic window - a closed interval $[x_{\text {min}},x_{\text {max}}]$ within which we will find a sonic point for the global solution of the ODE system on $[0,\infty )$. In fact, by Lemma 5.12 and Lemma 5.9, the set $W<\frac{1}{3}$ and the set $W>\frac{1}{2-2\eta }=\frac{1}{2}+O(\varepsilon )$ are invariant under the flow to the left of the sonic point. Motivated by this, we choose $x_{\text {max}}=x_{\text {max}}(\varepsilon )<3$ so that the zero order coefficient $W_0$ coincides with the Friedmann solution (2.40): $W_0|_{x_*= x_{\text {max}}}= \frac{1}{3}$ (see (4.245)) and fix $x_{\text {min}}=2+\delta _0>x_{\text {crit}}$ for $\delta _0>0$ sufficiently small but independent of $\varepsilon$, so that $W(x;x_{\text {min}})> \frac{1}{2-2\eta }$ for some $x<x_{\text {min}}$ (see (4.285)).

The main result of Section 4 is Theorem 4.18, which states that there exists an $0<\varepsilon _0\ll 1$ sufficiently small such that for all $0<\varepsilon \le \varepsilon _0$ and for any choice of $x_*$ in the sonic window, there in fact exists a local-in-*x* real analytic solution around $x=x_*$. The proof of this theorem relies on a delicate combinatorial argument, where enumeration of indices and *N*-dependent growth bounds for the coefficients $(D_N,W_N)$ are moved to Appendix A.

Having fixed the sonic window $[x_{\text {min}},x_{\text {max}}]\subset [2,3]$, our strategy is to determine what values of $x_*\in [x_{\text {min}},x_{\text {max}}]$ allow for RLP-type solutions that exist on the whole real line. We approach this problem by splitting it into two subquestions. We identify those $x_*$ which give global solutions to the left, i.e. all the way from $x=x_*$ to $x=0$, and separately to the right, i.e. on $[x_*,\infty )$.

### The Friedmann Connection (Section 5)

The main goal of Section 5 is to identify a value $\bar{x}_*\in [x_{\text {min}},x_{\text {max}}]$, so that the associated local solution $(D(\cdot ;\bar{x}_*), W(\cdot ;\bar{x}_*))$ exists on $[0,\bar{x}_*]$ and is real analytic everywhere.

From the technical point of view the main obstruction to our analysis is the possibility that the flow features more than one sonic point. By the precise analysis around $x=x_*$ in Section 4 we know that *B* is strictly positive/negative locally around $x_*$ to the left/right respectively (and vanishes at $x=x_*$). Our strategy is to propagate these signs dynamically. To do so we develop a technical tool, referred to as the monotonicity lemma, even though it is an exact identity, see Lemma 3.7. It is a first order differential equation for a quantity $f(x) = J[x;D]-xD$ with a source term depending on the solution, but with good sign properties in the regime we are interested in, hence – monotonicity lemma. Here $J[x;D]-xW$ is a factor of *B* in (2.37): $B=(1-\varepsilon )(J-xW)(J+2\eta (1+D)x+ xW)$ (see Lemmas 3.6–3.7). Roughly speaking, the function *f* allows us to relate the sign of *B* to the sign of the difference $(D-W)$ in a precise dynamic way, so that we eventually show that away from the sonic point $D>W$ and $B>0$ to the left, while $D<W$ and $B<0$ to the right of the sonic point for the relativistic Larson-Penston solution.

To construct the solution to the left of the sonic point we develop a shooting-type method, which we refer to as *shooting toward Friedmann*. Namely, the requirement of smoothness at $x=0$ is easily seen to imply $W(0)=\frac{1}{3}$, which precisely agrees with the value of $W_F$, see (2.40).

**Fig. 1:**
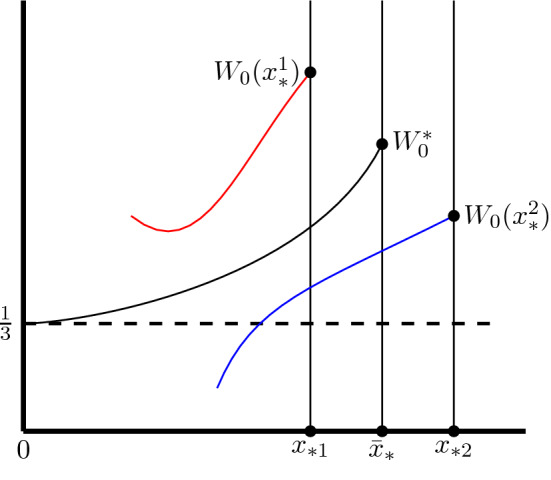
Schematic depiction of the shooting argument. Here $x_{*1}\in \mathcal {X}_{>\frac{1}{3}}$, $x_{*2}\in \mathcal {X}_{\frac{1}{3}}$. The critical point $\bar{x}_*$ is obtained by sliding to the left in $\mathcal {X}_{\frac{1}{3}}$ until we reach the boundary of its first connected component *X*

The key idea is to separate the sonic window $[x_{\text {min}},x_{\text {max}}]$ into the sets of sonic points $x_*$ that launch solutions $W(\cdot ;x_*)$ which either stay above the Friedmann value $W_F=\frac{1}{3}$ on its maximum interval of existence $(s(x_*),x_*]$ or cross it, see Figure 1. This motivates the following definition.

#### Definition 2.2

($\mathcal {X}_{>\frac{1}{3}}$, $\mathcal {X}_{\frac{1}{3}}$, $\mathcal {X}_{<\frac{1}{3}}$, and *X*) Let $\varepsilon _0>0$ be a small constant introduced in Section 2.2 (see also Theorem 4.18). For any $\varepsilon \in (0,\varepsilon _0]$ and $x_*\in [x_{\text {min}},x_{\text {max}}]$ we consider the associated RLP-type solution $(D(\cdot ;x_*), W(\cdot ;x_*))$. We introduce the sets

2.44 $$\begin{aligned} \mathcal {X}_{>\frac{1}{3}}&: = \left\{ x_*\in [x_{\text {min}},x_{\text {max}}]\,\Big | \inf _{x\in (s(x_*),x_*)}W(x;x_*)>\frac{1}{3} \right\} , \end{aligned}$$

2.45 $$\begin{aligned} \mathcal {X}_{\frac{1}{3}}&: = \left\{ x_*\in [x_{\text {min}},x_{\text {max}}]\,\big | \ \exists \ x\in (s(x_*), x_*) \quad \text {such that } W(x;x_*)=\frac{1}{3}\right\} , \end{aligned}$$

2.46 $$\begin{aligned} \mathcal {X}_{<\frac{1}{3}}&: = \left\{ x_*\in [x_{\text {min}},x_{\text {max}}]\,\Big | W(x;x_*)>\frac{1}{3} \quad \text { for all } \ x\in (s(x_*),x_*) \; \text {and} \; \inf _{x\in (s(x_*),x_*)}W(x;x_*)\le \frac{1}{3}\right\} . \end{aligned}$$

Finally, we introduce the fundamental set $X\subset \mathcal {X}_{\frac{1}{3}}$ given by

2.47 $$\begin{aligned} X: = \left\{ x_*\in [x_{\text {min}},x_{\text {max}}]\,\big | \quad \tilde{x}_*\in \mathcal {X}_{\frac{1}{3}}\quad \text { for all } \quad \tilde{x}_*\in [x_*,x_{\text {max}}] \right\} . \end{aligned}$$

The basic observation is that solutions that correspond to the set $\mathcal {X}_{\frac{1}{3}}$ have the property that once they take on value $\frac{1}{3}$, they never go back up above it. This is a nonlinear invariance of the flow, which guides our shooting argument idea. It is possible to show that both sets $\mathcal {X}_{>\frac{1}{3}}$ and $\mathcal {X}_{\frac{1}{3}}$ are non-empty. In Lemma 5.9 we show that $x_{\text {min}}\in \mathcal {X}_{>\frac{1}{3}}$ and that for some $\kappa >0$, $(x_{\text {max}}-\kappa ,x_{\text {max}}]\subset \mathcal {X}_{\frac{1}{3}}$. Intuitively, we then slide down $x_*$ starting from $x_{\text {max}}$, until we reach the first value of $x_*$ that does not belong to $\mathcal {X}_{\frac{1}{3}}$, i.e. we let

2.48 $$\begin{aligned} \bar{x}_*: = \inf _{x_*\in X } x_*, \end{aligned}$$

see Figure 1. This is the candidate for the value of $x_*$ which gives a real-analytic solution on $[0,\bar{x}_*]$. Using the nonlinear invariances of the flow and its continuity properties, in Proposition 5.15, we show that the solution $(D(\cdot ;\bar{x}_*),W(\cdot ,\bar{x}_*))$ exists on the semi-open interval $(0,\bar{x}_*]$.

To show that the solution is indeed analytic all the way to $x=0$, $W(0;\bar{x}_*)=\frac{1}{3}$, and $D(0;\bar{x}_*)>\frac{1}{3}$, we adapt the strategy developed for the classical LP solution in [15]. Using the method of *upper* and *lower* solutions (Definition 5.23), we show that there exists a choice of $D_0>\frac{1}{3}$ such that the solution $(D(\cdot ;\bar{x}_*),W(\cdot ,\bar{x}_*))$ coincides with a unique real analytic solution to (2.35)–(2.36), with data $D(0)=D_0$, $W(0)=\frac{1}{3}$, solving from $x=0$ to the right. Detailed account of this strategy is contained in Sections 5.3–5.4. Finally, combining the above results we can prove the central statement of Section 5:

#### Theorem 2.3

There exists an $\varepsilon _0>0$ sufficiently small, such that for any $0<\varepsilon \le \varepsilon _0$, the solution of RLP-type to (2.35)–(2.36) launched at $\bar{x}_*$ (defined by (2.48)) extends (to the left) to the closed interval $[0,\bar{x}_*]$, is real analytic, and satisfies $W(0;\bar{x}_*)=\frac{1}{3}$, $D(0;\bar{x}_*)>\frac{1}{3}$.

This theorem is formally proved at the very end of Section 5.4.

### The Far-Field Connection (Section 6)

By contrast to establishing the existence of the Friedmann connection in Section 5, we show that for any choice of $x_*$ in our sonic window $[x_{\text {min}},x_{\text {max}}]$, there exists a global solution to the right, i.e. we prove the following theorem:

#### Theorem 2.4

Let $x_*\in [x_{\text {min}},x_{\text {max}}]$. There exists an $0<\varepsilon _0\ll 1$ such that the unique RLP-type solution $(W,D)$ exists globally to the right for all $\varepsilon \in (0,\varepsilon _0]$.

The proof relies on a careful study of nonlinear invariances of the flow, and once again the monotonicity properties encoded in Lemma 3.7 play a critical role in our proof. This is highlighted in Lemma 6.3, which allows us to propagate the negativity of the sonic denominator *B* to the right of the sonic point. Importantly, we may now let $x_*= \bar{x}_*$ defined in (2.48) to obtain a real-analytic RLP-type solution defined globally on $[0,\infty )$. As it turns out, the obtained spacetime is not maximally extended, and to address this issue we need to understand the asymptotic behaviour of our solutions as $x\rightarrow \infty$.

In Lemma 6.5 we show that solutions from Theorem 2.4 honour the asymptotic behaviour

2.49 $$\begin{aligned} \lim _{x\rightarrow \infty } W(x;x_*)=1, \quad \lim _{x\rightarrow \infty } \left[ D(x;x_*)x^{\frac{2}{1+\eta }}\right] >0; \end{aligned}$$

hence the name far-field connection, see (2.39). This is however not enough for the purposes of extending the solution beyond $x=\infty$, as we also need sharp asymptotic behaviour of the relative velocity *W*. Working with the nonlinear flow (2.35)–(2.36), in Proposition 6.8 we show that the leading order behaviour of *W* is given by the relation

2.50 $$\begin{aligned} 1-W \asymp _{x\rightarrow \infty } x^{-\frac{1}{1+\eta }}. \end{aligned}$$

### Maximal Analytic Extension (Section 7)

Asymptotic relations (2.49)–(2.50) suggest that our unknowns are asymptotically “regular" only if thought of as functions of $x^{-\frac{1}{1+\eta }}$. In fact, it turns out to be more convenient to interpret this in the original comoving selfsimilar variable *y*, see (2.25). Due to (2.49) and (2.32)–(2.33), it is easy to see that asymptotically

2.51 $$\begin{aligned} x\asymp _{x\rightarrow \infty } y. \end{aligned}$$

Moreover, by (2.49) and (2.34), we have $\textbf{d}(y)\asymp _{y\rightarrow \infty }y^{-\frac{2}{1+\eta }}$. Furthermore by (3.101) we have the relation

2.52 $$\begin{aligned} e^{2\mu }= \frac{1}{(1+\varepsilon )^2} \Sigma ^{-\frac{\eta }{1+\eta }} = \frac{1}{(1+\varepsilon )^2} \textbf{d}^{-\eta }. \end{aligned}$$

As a consequence of (2.49) we then conclude that

2.53 $$\begin{aligned} e^{2\mu }\asymp _{y\rightarrow \infty } y^{\frac{4\varepsilon }{1+\varepsilon }}, \end{aligned}$$

which implies that the metric *g* given by (2.7) becomes singular as $y\rightarrow \infty$ (or equivalently $x\rightarrow \infty$). In Section 7 we show that this is merely a coordinate singularity, and the spacetime extends smoothly (in fact analytically in a suitable choice of coordinates) across the surface $\{(\tau \equiv 0,R)\, \big |\, R>0\}$.

Motivated by the above considerations, we switch to an adapted comoving chart $(\tilde{\tau },R)$, defined through

2.54 $$\begin{aligned} \tilde{\tau }(\tau ,R) =- \varepsilon ^{-\frac{\varepsilon }{1+\varepsilon }} R^{\frac{2\varepsilon }{1+\varepsilon }}(-\tau )^{\frac{1-\varepsilon }{1+\varepsilon }}, \quad \tau <0, \ R>0. \end{aligned}$$

We introduce the selfsimilar variable

2.55 $$\begin{aligned} Y: = \frac{-\sqrt{\varepsilon }\tilde{\tau }}{R}, \end{aligned}$$

which is then easily checked to be equivalent to the change of variables

2.56 $$\begin{aligned} Y = y^{-\frac{1}{1+\eta }}, \quad y = Y^{-1-\eta }, \end{aligned}$$

where we recall $\eta = \eta (\varepsilon )=\frac{2\varepsilon }{1-\varepsilon }$. To formulate the extension problem, it is natural to define the new variables

2.57 $$\begin{aligned} \chi (Y): = {\varvec{\chi }}(y), \quad d(Y) : = \textbf{d}(y), \quad w(Y) : = \textbf{w}(y), \end{aligned}$$

where we recall the fundamental variables $\textbf{d},\textbf{w},{\varvec{\chi }}$ from (2.26)–(2.28). Note that by (2.28) and (2.56) we have $\chi (Y)=Y^{1+\eta } \tilde{r}(y)$. It is shown in Lemma 7.1 that the original system (2.29)–(2.30) in the new variables reads

2.58 $$\begin{aligned} d'&= \frac{2\chi ^2}{Y} \frac{ d(w+\varepsilon )^2(d-w)}{\mathcal {C}}, \end{aligned}$$

2.59 $$\begin{aligned} w'&= -\frac{(w+\varepsilon )(1-3w)}{(1-\varepsilon )Y} -\frac{2(1+\varepsilon )\chi ^2}{(1-\varepsilon )Y} \frac{w(w+\varepsilon )^2(d-w)}{\mathcal {C}}, \end{aligned}$$

2.60 $$\begin{aligned} \chi '&= \frac{1-w}{(1-\varepsilon )Y} \chi , \end{aligned}$$

where

2.61 $$\begin{aligned} \mathcal {C} : = \left( dY^{-2}\right) ^{-\eta }Y^2 - \chi ^2 \left[ (w+ \varepsilon )^2-\varepsilon (w-1)^2 + 4\varepsilon wd\right] . \end{aligned}$$

The first main result of Section 7 is Theorem 7.4, where we prove the local existence of a real analytic solution in an open neighbourhood of $Y=0$, which provides the local extension of the solution from $Y=0^+$ to $Y=0^-$. Initial data at $Y=0$ are read off from the asymptotic behaviour (2.49)–(2.50), see Remark 7.2. The most important result of the section is the maximal extension theorem of the solution to the negative *Y*-s:

#### Theorem 2.5

(Maximal extension) There exists an $0<\varepsilon _0\ll 1$ sufficiently small such that for any $\varepsilon \in (0,\varepsilon _0]$ there exists a $Y^{\text {ms}}<0$ such that the unique solution to the initial value problem (2.58)–(2.60) exists on the interval $(Y^{\text {ms}},0]$, and

2.62 $$\begin{aligned}&\lim _{Y\rightarrow (Y^{\text {ms}})^-}w(Y) = \lim _{Y\rightarrow (Y^{\text {ms}})^-}d(Y) = \infty , \end{aligned}$$

2.63 $$\begin{aligned}&\chi (Y) >0, \quad Y\in (Y^{\text {ms}},0], \end{aligned}$$

2.64 $$\begin{aligned}&\lim _{Y\rightarrow (Y^{\text {ms}})^-}\chi (Y) = 0. \end{aligned}$$

**Fig. 2:**
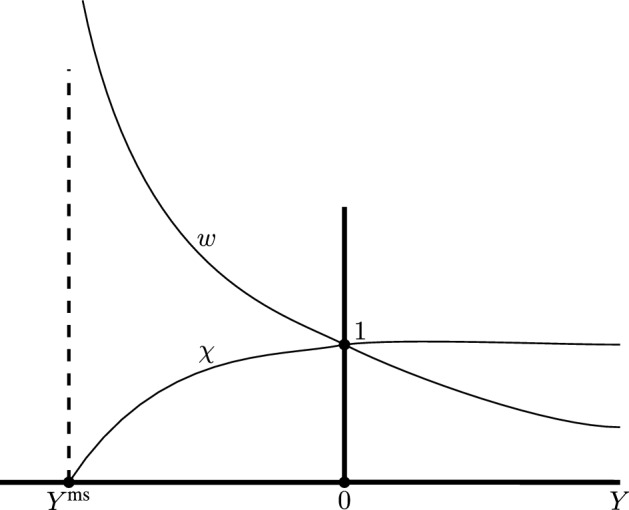
Schematic depiction of the behaviour of $\chi (Y)$ and $w(Y)$ in the maximal extension. As *Y* approaches $Y^{\text {ms}}$ from the right, $\chi$ approaches 0 and $w$ blows up to $\infty$

We see from the statement of the theorem that the maximal extension is characterised by the simultaneous blow-up of $w, d,$ and $\frac{1}{\chi }$ at the terminal point $Y^{\text {ms}}$. By (2.55), in the adapted comoving chart, the point $Y^{\text {ms}}$ coincides with the hypersurface

$$\begin{aligned} \mathcal {M}\mathcal {S}_\varepsilon {:}{=}\left\{ (\tilde{\tau },R)\,\big | \tilde{\tau }= \frac{1}{\sqrt{\varepsilon }} |Y^{\text {ms}}| R\right\} \setminus \{(0,0)\} , \end{aligned}$$

which we refer to as the *massive singularity*, following the terminology in [28]. The proof of Theorem 2.5 relies on a careful understanding of nonlinear invariances associated with the dynamical system (2.58)–(2.60) and the key dynamic “sandwich" bound

$$\begin{aligned} 1\lesssim \frac{d}{w}<1, \quad \text { for } Y<Y_0<0, \end{aligned}$$

where $Y_0<0$ is small. This is shown in Lemma 7.9. The blow-up proof finally follows from a Ricatti-type ordinary differential inequality for the relative velocity $w$.

In Section 7.4 we compute the sharp asymptotics of $w, d,$ and $\chi$ on approach to the massive singularity $Y^{\text {ms}}$. This result is stated in Proposition 7.16, which is later crucially used in the study of the causal structure of such a maximally extended solution, where it is in particular shown that the spacetime curvature blows up on approach to the massive singularity.

#### Remark 2.6

The maximal selfsimilar extension makes sense in the Newtonian limit $\varepsilon \rightarrow 0$, which is one of the key observations of Ori and Piran [28]. Our proof of Theorem 2.5 easily extends to the simpler case $\varepsilon =0$, which in particular shows that the LP-solutions constructed in [15] have a natural maximal extension in the Lagrangian (comoving) coordinates.

### The RLP-Spacetime and Its Causal Structure (Section 8)

As a consequence of Theorems 2.3, 2.4, and 2.5, we can now formally introduce the exactly selfsimilar solution of the Einstein-Euler system by patching the solutions in the subsonic region $x\in [0,\bar{x}_*]$, the supersonic region $x\in [\bar{x}_*,\infty )$, and the extended region $Y\in (Y^{\text {ms}},0]$. Following Ori and Piran [28], we call this spacetime the relativistic Larson-Penston (RLP) solution^3^.

#### Definition 2.7

($g_{\text {RLP},\varepsilon }$-metric) We refer to the 1-parameter family of spherically symmetric selfsimilar spacetimes $(\mathcal {M}_{\text {RLP},\varepsilon }, g_{\text {RLP},\varepsilon })$ constructed above as the relativistic Larson-Penston spacetimes. In the adapted comoving coordinates $(\tilde{\tau },R)$ the metric takes the form

2.65 $$\begin{aligned} g_{\text {RLP},\varepsilon }&= - e^{2\tilde{\mu }}\,d\tilde{\tau }^2 -\frac{4\sqrt{\varepsilon }}{1+\varepsilon } Y e^{2\tilde{\mu }}\,d\tilde{\tau }\,dR + \left( e^{2\tilde{\lambda }}-\frac{4\varepsilon }{(1+\varepsilon )^2} Y^2 e^{2\tilde{\mu }} \right) \,dR^2 + r^2\; \gamma , \end{aligned}$$

where the metric coefficients are defined on the connected component of the $(\tilde{\tau },R)$ coordinate plane given by

2.66 $$\begin{aligned} \tilde{\mathcal {D}}_{\text {RLP},\varepsilon }: = \left\{ (\tilde{\tau }, R)\,\big | \, R>0, \quad Y\in (Y^{\text {ms}},\infty )\right\} . \end{aligned}$$

Here

2.67 $$\begin{aligned} r(\tilde{\tau },R) = \chi (Y) R, \quad \tilde{\mu }(\tilde{\tau },R) = \tilde{\mu }(Y), \quad \tilde{\lambda }(\tilde{\tau },R) = \tilde{\lambda }(Y), \end{aligned}$$

where $$ Y = -\frac{\sqrt{\varepsilon }\tilde{\tau }}{R}, $$

2.68 $$\begin{aligned} e^{2\tilde{\mu }(Y)} = \frac{(1+\varepsilon )^2}{(1-\varepsilon )^2} Y^{2\eta } e^{2\mu (y)}, \quad y>0, \end{aligned}$$

and

2.69 $$\begin{aligned} e^{2\tilde{\lambda }(Y)} = e^{2\lambda (y)}, \quad y>0. \end{aligned}$$

Metric coefficients $\tilde{\mu }(Y),\tilde{\lambda }(Y)$ for $Y\le 0$ are then defined by expressing them as appropriate functions of $d,w,\chi$ and extending to $Y\le 0$, see Proposition 7.16.

*The RLP-spacetime in the original comoving coordinates.* In the original comoving coordinates $(\tau ,R)$, the metric takes the form

2.70 $$\begin{aligned} g_{\text {RLP},\varepsilon }&= -e^{2\mu } d\tau ^2 + e^{2\lambda }dR^2 + r^2 \; \gamma . \end{aligned}$$

It is clear from (2.53) that the metric (2.70) becomes singular across the surface $\tau =0$ (equivalently $y=\infty$). Nevertheless, away from this surface (i.e. when $\tau >0$ and $\tau <0$) we can keep using comoving coordinates, whereby we define

2.71 $$\begin{aligned} \tau = |Y|^\eta \tilde{\tau }, \quad \tilde{\tau }>0, \end{aligned}$$

where we recall (2.55). Therefore the metric coefficients are defined on the union of the connected components of the $(\tau ,R)$ coordinate plane given by

2.72 $$\begin{aligned} \mathcal {D}_{\text {RLP},\varepsilon }: = \left\{ (\tau , R) \in (-\infty ,0)\times (0,\infty )\right\} \cup \left\{ (\tau , R) \in (0,\infty )\times (0,\infty )\,\big | \, y\in (-\infty , y^{\text {ms}})\right\} , \end{aligned}$$

where

$$\begin{aligned} y^{\text {ms}} : = - |Y^{\text {ms}}|^{-(1+\eta )}. \end{aligned}$$

Here

$$\begin{aligned} r(\tau ,R) = - \sqrt{\varepsilon }\tau \tilde{r}(y), \quad \mu (\tau ,R) = \mu (y), \quad \lambda (\tau ,R) = \lambda (y), \quad \text { for }\quad \tau <0, \end{aligned}$$

and

$$\begin{aligned} r = - \sqrt{\varepsilon }\tau \tilde{r}(y), \quad \tilde{r}(y) = {\varvec{\chi }}(y) y, \quad y = \frac{R}{-\sqrt{\varepsilon }\tau } =- |Y|^{-1-\eta }, \quad \text { for }\quad \tau >0, \end{aligned}$$

where $${\varvec{\chi }}(y)=\chi (Y)$$, $$y<0$$.

#### Remark 2.8

It is of interest to understand the leading order asymptotic behaviour of the radius $\tilde{r}$ as a function of the comoving selfsimilar variable *y*. It follows from (2.34) and the boundary condition $W(0)=\frac{1}{3}$ that $\lim _{y\rightarrow 0^+}\textbf{w}(y) = \frac{1}{3}$. Therefore, using (2.33) it is easy to see that the leading order behaviour of $\tilde{r}(y)$ at $y=0$ is of the form

2.73 $$\begin{aligned} \tilde{r}(y) = \tilde{r}_0 y^{\frac{1+3\varepsilon }{3(1+\varepsilon )}} + o_{y\rightarrow 0^+}(y^{\frac{1+3\varepsilon }{3(1+\varepsilon )}}), \quad \tilde{r}'(y) \asymp _{y\rightarrow 0^+} y^{-\frac{2}{3(1+\varepsilon )}}, \end{aligned}$$

for some $\tilde{r}_0>0$. It follows in particular that $y\mapsto \tilde{r}(y)$ is only $C^{0,\frac{1+3\varepsilon }{3(1+\varepsilon )}}$ at $y=0$. The selfsimilar reduction of the constraint equation (2.15) reads $\tilde{r}'^2 e^{-2\lambda } = 1+\varepsilon V^2 - \frac{2}{3} \varepsilon \tilde{G}\tilde{r}^2$ (see (3.95)). Using this and (2.73), it then follows

2.74 $$\begin{aligned} e^{\lambda (y)}\asymp _{y\rightarrow 0^+} \tilde{r}'(y) \rightarrow _{y\rightarrow 0^+} \infty , \end{aligned}$$

which shows that the metric *g* (2.7) is singular at $y=0$.

This singularity is not geometric, but instead caused by the Friedmann-like behaviour of the relative velocity *W* at $y=0$, see (2.40). It captures an important difference between the comoving and the Schwarzschild coordinates at the centre of symmetry $\{(\tau , R)\, \big | \, \tau <0, R=0\}$. It can be checked that the space-time is regular at the centre of symmetry by switching to the $(\tau ,r)$ coordinate. The same phenomenon occurs in the Newtonian setting, where it can be shown that the map ${\varvec{\chi }}= \frac{\tilde{r}}{y}$ associated with the LP-solution is exactly $C^{0,\frac{1}{3}}$ and therefore ${\varvec{\chi }}$ is not smooth at the labelling origin $y=0$.

#### Remark 2.9

Note that the trace of $T_{\mu \nu }$ is easily evaluated

$$\begin{aligned} g^{\mu \nu }T_{\mu \nu } = (\rho +p)g^{\mu \nu }u_\mu u_\nu + 4 p = - \rho +3p = -(1-3\varepsilon )\rho . \end{aligned}$$

On the other hand, the trace of the left-hand side of (1.1) is exactly $-\mathcal {R}$, and therefore the Ricci scalar of any classical solution of (1.1)–(1.5) satisfies the relation

2.75 $$\begin{aligned} \mathcal {R} = (1-3\varepsilon )\rho . \end{aligned}$$

This relation also implies that the blow-up of the Ricci scalar is equivalent to the blow up of the mass-energy density when $\varepsilon \ne \frac{1}{3}$.

#### Remark 2.10

In Christodolou’s work [6], across the boundary of the backward light cone $\mathcal {N}$ emanating from the first singularity, the selfsimilar solution has finite regularity (measured in the Hölder class). This is to be contrasted with the RLP solution which remains real analytic across both characteristic cones - the sonic line and the light cone.

#### The Outgoing Null-Geodesic

**Fig. 3:**
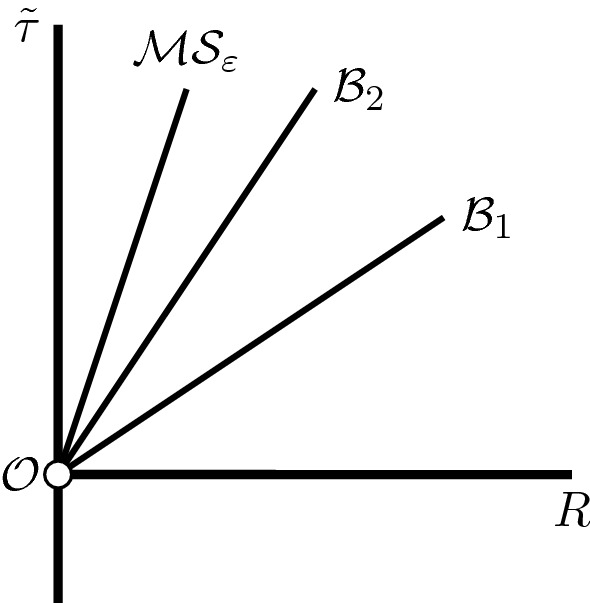
Schematic depiction of the outgoing null-geodesics $\mathcal {B}_1$, $\mathcal {B}_2$, and the massive singularity $\mathcal {M}\mathcal {S}_\varepsilon$

The maximally extended RLP spacetime constructed above has two singular boundary components - the scaling origin $\mathcal {O}$ and the massive singularity $\mathcal {M}\mathcal {S}_\varepsilon$ introduced in Section 2.5, see Figure 3. They are very different in nature, as the density (and the curvature components) blow up at two distinct rates.

The main result of Section 8 states that there exist an outgoing radial null-geodesic (RNG) emanating from the scaling origin $\mathcal {O}$ and reaching infinity. Following Ori and Piran we look for a so-called simple RNG:

##### Definition 2.11

(Simple radial null-geodesics) An RNG of the form

2.76 $$\begin{aligned} R(\tilde{\tau }) = \sigma \tilde{\tau }, \quad \sigma \in \mathbb R\setminus \{0\}, \end{aligned}$$

is called a simple radial null-geodesic (simple RNG).

Then the key result we prove is the following theorem.

##### Theorem 2.12

(Existence of global outgoing simple RNG-s) There exists an $0<\varepsilon _0\ll 1$ sufficiently small so that for any $\varepsilon \in (0,\varepsilon _0]$ there exist at least two and at most finitely many outgoing simple RNG-s emanating out of the singularity (0, 0). In other words, there exist

2.77 $$\begin{aligned} Y^{\text {ms}}< Y_n<\dots Y_1 <0, \quad n\ge 2, \end{aligned}$$

so that the associated simple RNG-s are given by

2.78 $$\begin{aligned} \mathcal {B}_i : = \{(\tilde{\tau }, R)\in \mathcal {M}_{\text {RLP},\varepsilon }\,\big | \, \frac{-\sqrt{\varepsilon }\tilde{\tau }}{R} = Y_{i}\}, \quad i=1,\dots n. \end{aligned}$$

The proof relies on a beautiful idea of Ori and Piran [28], which we make rigorous. Namely, one can show that the slopes of outgoing simple RNG-s must correspond to roots of a certain real-analytic function, see Lemma 8.2. Using the sharp asymptotic behaviour of the metric coefficients, brought about through our analysis in Sections 6–7, we can prove that this function converges to negative values at $Y=0$ and $Y=Y^{\text {ms}}$. On the other hand, by the local existence theory for the ODE-system (2.58)–(2.60), we can also ascertain the function in question peaks above 0 for a $Y_0\in (Y^{\text {ms}},0)$. Therefore, by the intermediate value theorem, we conclude the proof of Theorem 2.12, see Section 8, immediately after Proposition 8.4.

Informally, the null-hypersurface $\mathcal {B}_1$ is the “first" outgoing null-curve emanating from the singular scaling origin and reaching the infinity. It is easy to see that *r* grows to $+\infty$ along $\mathcal {B}_1$. Since the spacetime is not asymptotically flat, we perform a suitable truncation in Section 9 in order to interpret $\mathcal {O}$ as a naked singularity.

The maximal extension we construct is unique only if we insist on it being selfsimilar, otherwise there could exist other extensions in the causal future of $\mathcal {O}$. Nevertheless, in our analysis the role of the massive singularity $\mathcal {M}\mathcal {S}_\varepsilon$ is important, as we use the sharp blow-up asymptotics of our unknowns to run the intermediate value theorem-argument above. Conceptually, $\mathcal {M}\mathcal {S}_\varepsilon$ has a natural Newtonian limit as $\varepsilon \rightarrow 0$, see Remark 2.6, which makes it a useful object for our analysis.

**Fig. 4:**
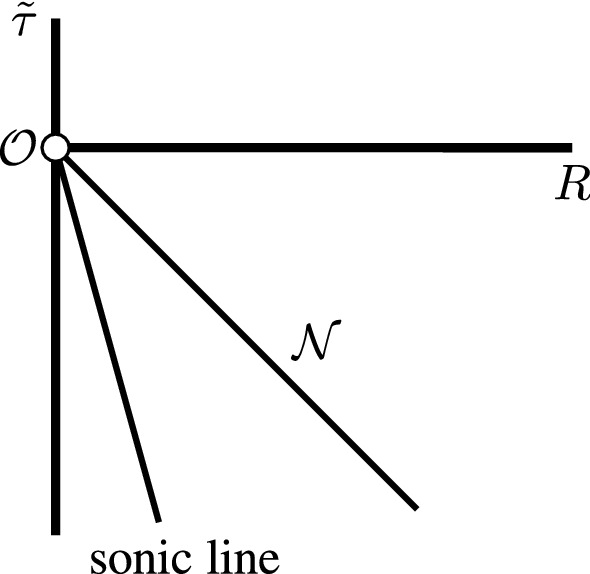
Schematic depiction of the ingoing null-curve $\mathcal {N}$ and the sonic line. The spacetime is smooth across both surfaces

**Fig. 5:**
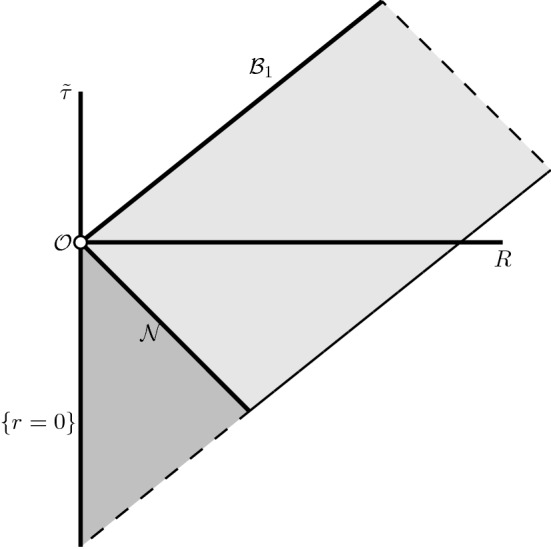
Schematic depiction of the interior (dark grey) and the exterior (light grey) region, see Definition 8.6

In the remainder of Section 8 we give a detailed account of radial null-geodesics, showing in particular that there is a unique ingoing null-geodesic $\mathcal {N}$ emanating from the scaling origin to the past, see Figure 4. This is the boundary of the backward light cone “emanating" from the scaling origin. Following the terminology in [6], it splits the spacetime into the *exterior* region (in the future of $\mathcal {N}$) and the *interior* region (in the past of $\mathcal {N}$), see Definition 8.6 and Figure 5. Moreover, the sonic line is contained strictly in the interior region. The complete analysis of nonradial null-geodesics is given in Appendix B.

### Double Null Gauge, Asymptotic Flattening, and Naked Singularities (Section 9)

The final step in the proof of Theorem 1.2 is to truncate the profile away from the scaling origin $\mathcal {O}$ and glue it to the already constructed selfsimilar solution. To do that we set up this problem in the double-null gauge:

2.79 $$\begin{aligned} g = - \Omega ^2 \, dp\,dq+ r^2 \gamma , \end{aligned}$$

where $p=\text {const.}$ corresponds to outgoing null-surfaces and $q=\text {const.}$ to the ingoing null-surfaces. A similar procedure for the scalar field model was implemented in [6], however due to the complications associated with the Euler evolution, a mere cut-off argument connecting the “inner region" to pure vacuum as we approach null-infinity is hard to do. Instead, we carefully design function spaces that capture the asymptotic decay of the fluid density toward future null-infinity in a way that is both consistent with the asymptotic flatness, and can be propagated dynamically.

#### Formulation of the Problem in Double-Null Gauge

In addition to the unknowns associated with the fluid, the metric unknowns are the conformal factor $\Omega =\Omega (p,q)$ and the areal radius $r=r(p,q)$. Clearly,

2.80 $$\begin{aligned} g_{pp}=g_{qq}=0, \quad g_{pq} = -\frac{1}{2}\Omega ^2, \quad g^{pq} = - 2\Omega ^{-2}. \end{aligned}$$

Let $u = (u^{p}, u^{q},0,0)$ be the components of the velocity 4-vector *u* in the frame $\{\partial _{p}, \ \partial _{q}, \ E_{2}, \ E_3\}$, where $\{E_A\}$, $A=2,3$, describe the generators of some local coordinates on $\mathbb S^2$. We have $g(\partial _{p},E_A) = g(\partial _{q}, E_A) = 0$, $A=2,3$. The normalisation condition (1.3) in the double-null gauge reads

2.81 $$\begin{aligned} -1 = g^{\mu \nu }u_{\mu }u_{\nu } = g^{pq} u_pu_q= -4\Omega ^{-2} u_pu_q, \end{aligned}$$

which therefore equivalently reads

2.82 $$\begin{aligned} u_pu_q= \frac{1}{4} \Omega ^2, \quad u^pu^q= \Omega ^{-2}. \end{aligned}$$

##### Lemma 2.13

(Einstein-Euler system in double-null gauge) In the double-null gauge (2.79), the Einstein field equations take the form

2.83 $$\begin{aligned} \partial _{p}\partial _{q}r&= - \frac{\Omega ^2}{4r} - \frac{1}{r} \partial _{p}r \partial _{q}r + \pi r\Omega ^4 T^{pq}, \end{aligned}$$

2.84 $$\begin{aligned} \partial _{p}\partial _{q}\log \Omega&= - (1+\eta )\pi \Omega ^4 T^{pq} +\frac{\Omega ^2}{4r^2} + \frac{1}{r^2} \partial _{p}r \partial _{q}r, \end{aligned}$$

2.85 $$\begin{aligned} \partial _{q}\left( \Omega ^{-2}\partial _{q}r\right)&= - \pi r \Omega ^2 T^{pp}, \end{aligned}$$

2.86 $$\begin{aligned} \partial _{p}\left( \Omega ^{-2}\partial _{p}r\right)&= - \pi r \Omega ^2 T^{qq}. \end{aligned}$$

Moreover, the components of the energy-momentum tensor satisfy

2.87 $$\begin{aligned} \partial _p(\Omega ^4r^2T^{pp}) + \frac{\Omega ^2}{r^{2\eta }} \partial _q( \Omega ^2r^{2+2\eta } T^{pq} )&=0, \end{aligned}$$

2.88 $$\begin{aligned} \partial _p( \Omega ^2r^{2+2\eta } T^{pq} ) + \frac{r^{2\eta }}{\Omega ^2}\partial _q(\Omega ^4r^2T^{qq})&= 0, \end{aligned}$$

where the energy-momentum tensor is given by the formulas

2.89 $$\begin{aligned}&T^{pp} = (1+\varepsilon )\rho (u^p)^2, \quad T^{qq} = (1+\varepsilon )\rho (u^q)^2, \quad T^{pq} = (1-\varepsilon )\rho \Omega ^{-2}, \end{aligned}$$

2.90 $$\begin{aligned}&T^{AB} = \varepsilon \rho r^{-2} \gamma ^{AB}, \quad T^{pA} = T^{qA} = 0, \quad A,B = 2,3. \end{aligned}$$

Moreover, its components are related through the algebraic relation

2.91 $$\begin{aligned} T^{pp} T^{qq} = (1+\eta )^2(T^{pq})^2 . \end{aligned}$$

##### Proof

The proof is a straightforward calculation and is given in Appendix C. $\square$

**Fig. 6:**
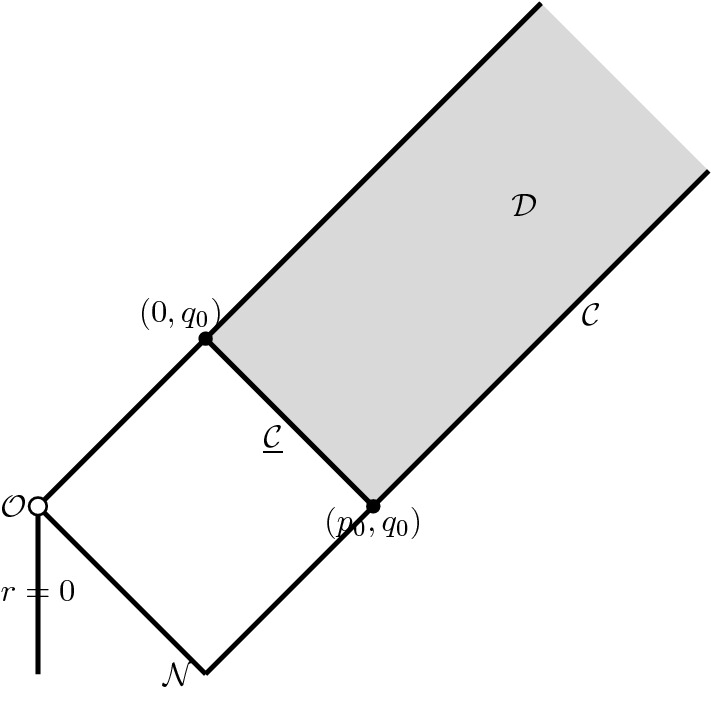
The grey shaded area is the region $\mathcal {D}$, where the truncation of the selfsimilar profile takes place. Data are prescribed on the two characteristic surfaces $\mathcal {C}$ and $\underline{\mathcal {C}}$

#### The Characteristic Cauchy Problem and Asymptotic Flattening

The idea is to choose a point $(p_0,q_0)$ in the exterior region (recall Figure 5) and solve the Einstein-Euler system in an infinite semi-rectangular domain (in the $(p,q)$-plane) $\mathcal {D}$ depicted in Figure 6. We normalise the choice of the double-null coordinates by making the outgoing curve $\mathcal {B}_1$ in the RLP-spacetime (see Figures 4–5) correspond to the $\{p=0\}$ level set, and the ingoing curve $\mathcal {N}$ (see Figure 5) to the $\{q=0\}$ level set. We have the freedom to prescribe the data along the ingoing boundary $\underline{\mathcal {C}}= \{(p,q)\,\big | \, p\in [p_0,0], q=q_0\}$ and the outgoing boundary $\mathcal {C}= \{(p,q)\,\big | \, p=p_0, q\ge q_0\}$. On $\underline{\mathcal {C}}$ we demand that data be given by the restriction of the selfsimilar RLP solution to $\underline{\mathcal {C}}$, and on the outgoing piece we make the data exactly selfsimilar on a subinterval $q\in [q_0,q_0+A_0]$ for some $A_0>0$. On the remaining part of the outgoing boundary $q\in [q_0+A_0,\infty )$, we prescribe asymptotically flat data.

The key result of Section 9 is Theorem 9.4, which states that the above described PDE is well-posed on $\mathcal {D}$, if we choose $|p_0|=\delta$ sufficiently small. We are not aware of such a well-posedness result for the Einstein-Euler system in the double-null gauge in the literature, and we therefore carefully develop the necessary theory. Precise statements are provided in Section 9.2. The idea is standard and relies on the method of characteristics. However, to make it work we rely on an effective “diagonalisation" of the Euler equations (2.87)–(2.88), which replaces these equations by two transport equations for the new unknowns $f^+$ and $f^-$, see Lemma 9.1. This change of variables highlights the role of the acoustic cone and allows us to track the acoustic domain of dependence by following the characteristics, see Figure 6. The analysis of the fluid characteristics is presented in Section 9.3. Various a priori bounds are given in Section 9.4. In Section 9.5 we finally introduce an iteration procedure and prove Theorem 9.4.

**Fig. 7:**
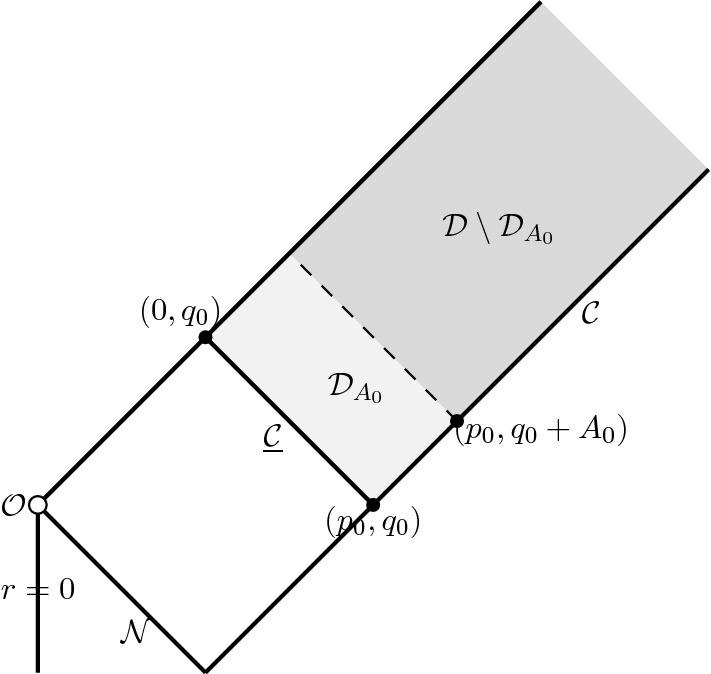
The light grey shaded area is the region $\mathcal {D}_{A_0}$, where the truncated solution from Theorem 9.4 coincides with the exact selfsimilar solution

#### Formation of Naked Singularities: Proof of Theorem 1.2

Since the data on $\underline{\mathcal {C}}$ and the portion of $\mathcal {C}$ with $q_0\le q\le q_0+A_0$ agrees with the RLP-solution, the solution must, by the finite-speed of propagation, coincide with the exact RLP solution in the region $\mathcal {D}_{A_0}$ depicted in Figure 7. By uniqueness the solution extends smoothly (in fact analytically) to the exact RLP-solution in the past of $\underline{\mathcal {C}}$, all the way to the regular centre $\{r=R=0\}$.

**Fig. 8:**
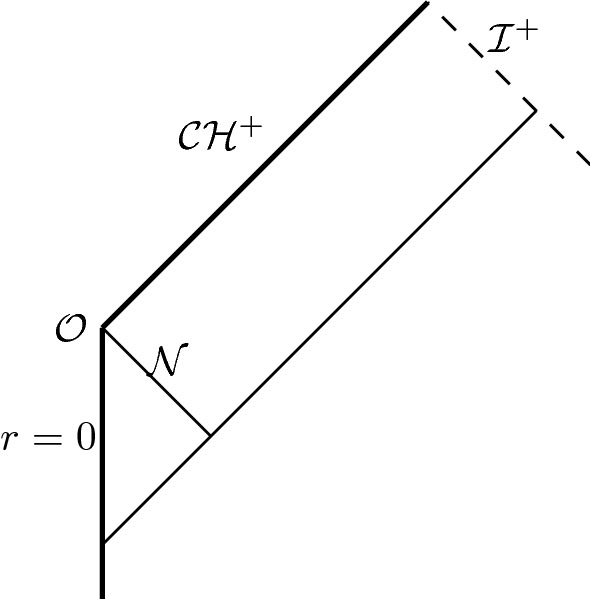
Penrose diagram of our spacetime with an incomplete future null-infinity

The future boundary of the maximal development is precisely the surface $\{p=0\}$, the Cauchy horizon for the new spacetime. The boundary of the null-cone corresponding to $\{p=p_0\}$ is complete, and we show in Section 9.6 that for any sequence of points $(p_0,q_n)$ with $q_n$ approaching infinity, the affine length of maximal future-oriented ingoing geodesics launched from $(p_0,q_n)$ and normalised so that the tangent vector corresponds to $\partial _{p}$, is bounded by a constant, uniformly-in-*n*. This shows that the future null-infinity is incomplete in the sense of [32, Definition 1.1], thus completing the proof of Theorem 1.2, see the Penrose diagram, Figure 8.

## Radially Symmetric Einstein-Euler System in Comoving Coordinates

### Selfsimilar Comoving Coordinates

We plug in (2.19)–(2.24) into (2.12)–(2.15) and obtain the selfsimilar formulation of the Einstein-Euler system:

3.92 $$\begin{aligned} 2\Sigma + y\Sigma '(y) +(1+\varepsilon )\Sigma \left( \frac{V'}{\tilde{r}'}+\frac{2V}{\tilde{r}}\right) e^{\mu }&= 0, \end{aligned}$$

3.93 $$\begin{aligned} y \lambda '(y) = e^{\mu }\frac{V'}{\tilde{r}'}, \end{aligned}$$

3.94 $$\begin{aligned} e^{-\mu } y V'(y) + \frac{1}{1+\varepsilon } \frac{\tilde{r}'(y) e^{-2\lambda (y)}}{\Sigma (y)}\Sigma '(y) + \tilde{r}\left( \frac{1}{3} \tilde{G}+ 2\varepsilon \Sigma \right)&= 0 , \end{aligned}$$

3.95 $$\begin{aligned} \tilde{r}'^2 e^{-2\lambda }&= 1+\varepsilon V^2 - \frac{2}{3} \varepsilon \tilde{G}\tilde{r}^2. \end{aligned}$$

#### Remark 3.1

Recall that the radial velocity $\mathcal {V}$ satisfies $\mathcal {V}=e^{-\mu }\partial _\tau r$ and therefore

3.96 $$\begin{aligned} V = e^{-\mu } (-\tilde{r}+ y\tilde{r}'(y)). \end{aligned}$$

From (3.92), (3.93), and (3.96) we obtain

$$\begin{aligned} 0&= 2 + y\frac{\Sigma '(y)}{\Sigma (y)} +(1+\varepsilon )\left( y \lambda '(y)+2 \left( y \frac{\tilde{r}'(y)}{\tilde{r}(y)}-1\right) \right) \end{aligned}$$

and therefore

3.97 $$\begin{aligned} \lambda '(y) = -\frac{1}{1+\varepsilon }\left( \frac{\Sigma '}{\Sigma }+\frac{2}{y}\right) -2 \left( \frac{\tilde{r}'}{\tilde{r}}-\frac{1}{y}\right) . \end{aligned}$$

For smooth solutions with strictly positive density $\Sigma$, an explicit integration of the identity (3.97) gives the formula

3.98 $$\begin{aligned} e^{\lambda } = \alpha \Sigma ^{-\frac{1}{1+\varepsilon }} y^{\frac{2\varepsilon }{1+\varepsilon }}\tilde{r}^{-2}, \end{aligned}$$

for some constant $\alpha >0$.

#### Remark 3.2

(Gauge normalisation) From (2.16) we also obtain

3.99 $$\begin{aligned} \mu '(y) = - \frac{\varepsilon }{1+\varepsilon } \frac{\Sigma '(y)}{\Sigma (y)}. \end{aligned}$$

For any given $0<\varepsilon <1$ we finally fix the remaining freedom in the problem by setting

3.100 $$\begin{aligned} c = e^{2\mu (0)}\Sigma (0)^\frac{2\varepsilon }{\varepsilon +1} = \frac{1}{(1+\varepsilon )^2}. \end{aligned}$$

Upon integrating (3.99) we obtain the identity

3.101 $$\begin{aligned} e^{\mu (y)} = \frac{1}{1+\varepsilon } \; \Sigma (y)^{-\frac{\varepsilon }{1+\varepsilon }}. \end{aligned}$$

Before we reduce the selfsimilar formulation (3.92)–(3.95) to a suitable $2\times 2$ nonautonomous ODE system, we first prove an auxiliary lemma.

#### Lemma 3.3

Let $(\Sigma ,V,\lambda ,\mu )$ be a smooth solution to (3.92)–(3.95).

*(a)*: The following important relationship holds:
  3.102 $$\begin{aligned} \frac{\textbf{w}+\varepsilon }{1+\varepsilon } = \frac{y\tilde{r}'}{\tilde{r}}, \end{aligned}$$
  with $\textbf{w}$ defined by (2.27).
*(b)*: (Local expression for the mean density) The selfsimilar mean density $\tilde{G}$ defined by
  3.103 $$\begin{aligned} \tilde{G}(y) = \frac{6}{\tilde{r}^3}\int _0^y \Sigma \tilde{r}^2 \tilde{r}' d\tilde{y} \end{aligned}$$
  satisfies the relation
  3.104 $$\begin{aligned} \tilde{G} = 6 \Sigma \textbf{w}. \end{aligned}$$
*(c)*: The expression *K* defined by
  3.105 $$\begin{aligned} K{:}{=}-\frac{\textbf{w}}{1+\varepsilon }+e^{2\mu }\left( \frac{1}{6}\frac{(1+\varepsilon )\tilde{G}}{\textbf{w}+\varepsilon }+\frac{\varepsilon (1+\varepsilon ) \Sigma }{\textbf{w}+\varepsilon }\right) . \end{aligned}$$
  satisfies the relation
  3.106 $$\begin{aligned} K = \frac{1}{1+\varepsilon }\left( \textbf{d}- \textbf{w}\right) , \end{aligned}$$
  with $\textbf{d}$ defined by (2.26).
*(d)*: The metric coefficient $e^{2\lambda }$ satisfies the formula
  3.107 $$\begin{aligned} e^{2\lambda } = \frac{\tilde{r}^2(\textbf{w}+\varepsilon )^2}{(1+\varepsilon )^2 y^2\left[ 1 + \varepsilon \Sigma ^{\frac{2\varepsilon }{1+\varepsilon }}\tilde{r}^2(\textbf{w}-1)^2-4\varepsilon \Sigma \textbf{w}\tilde{r}^2\right] }. \end{aligned}$$

#### Proof

*Proof of part (a).* This is a trivial consequence of  (3.96) and (2.27).

*Proof of part (b).* After multiplying (3.92) by $\tilde{r}^2 \tilde{r}'$ we obtain

3.108 $$\begin{aligned} 0&= 2\Sigma \tilde{r}^2 \tilde{r}' + \underbrace{y \tilde{r}' }_{= \tilde{r}+ Ve^\mu }\tilde{r}^2 \Sigma ' + (1+\varepsilon ) \Sigma (\tilde{r}^2 V' + 2 \tilde{r} \tilde{r}' V) e^\mu \end{aligned}$$

3.109 $$\begin{aligned}&= 2\Sigma \tilde{r}^2 \tilde{r}' + \Sigma ' \tilde{r}^3 + e^\mu \Sigma ' \tilde{r}^2 V + (1+\varepsilon ) e^\mu \Sigma (\tilde{r}^2 V)' \end{aligned}$$

3.110 $$\begin{aligned}&= 2\Sigma \tilde{r}^2 \tilde{r}' + \Sigma ' \tilde{r}^3 + (1+\varepsilon ) (e^\mu \Sigma \tilde{r}^2 V) ' \end{aligned}$$

where in the second line we used $y \tilde{r}' = \tilde{r}+ Ve^\mu$ (which follows from (3.96)) and in the last line the identity

$$\begin{aligned} (\Sigma e^\mu )' = \Sigma ' e^\mu + \Sigma e^\mu ( - \frac{\varepsilon }{1+\varepsilon } \frac{\Sigma '}{\Sigma }) = \frac{1}{1+\varepsilon } \Sigma ' e^\mu , \end{aligned}$$

which uses the field equation (3.99). After integrating over the interval [0, *y*] and integrating-by-parts, we obtain

3.111 $$\begin{aligned} \int _0^y \Sigma \tilde{r}^2 \tilde{r}' ds = \Sigma \tilde{r}^2 (\tilde{r} + (1+\varepsilon ) e^\mu V) = \Sigma \tilde{r}^3 \textbf{w}\end{aligned}$$

where we have used (2.27). Hence dividing by $\tilde{r}^3$ we obtain (3.104).

*Proof of part (c).* From (3.105) and (3.104) we immediately have

3.112 $$\begin{aligned} K = (1+\varepsilon )e^{2\mu } \Sigma \frac{\textbf{w}}{1+\varepsilon } =\frac{1}{1+\varepsilon } \left( \Sigma ^{\frac{1-\varepsilon }{1+\varepsilon }} - \textbf{w}\right) , \end{aligned}$$

where we have used (3.101).

*Proof of part (d).* The formula is a simple consequence of (3.95), (3.96), and (3.104). $\square$

#### Proposition 3.4

(The ODE system in comoving selfsimilar coordinates) Let $(\Sigma ,V,\lambda ,\mu )$ be a smooth solution to (3.92)–(3.95). Then the pair $(\textbf{d},\textbf{w})$ solves the system (2.29)–(2.30), where $\textbf{d},\textbf{w}$ are defined in (2.26)–(2.27).

#### Proof

A routine calculation starting from (3.92) and (3.94) (these can be thought of as the continuity and the momentum equation respectively) gives:

3.113 $$\begin{aligned}&\left( y+\varepsilon \frac{y}{\textbf{w}+\varepsilon }(\textbf{w}-1) \right) \Sigma ' + \frac{(1+\varepsilon )\Sigma y}{\textbf{w}+\varepsilon }\textbf{w}' - (1+3\varepsilon )\Sigma + 3 (\textbf{w}+\varepsilon )\Sigma = 0, \end{aligned}$$

3.114 $$\begin{aligned}&e^{-2\mu }y\Sigma \textbf{w}' + \frac{1}{1+\varepsilon }\left( \varepsilon e^{-2\mu } y (\textbf{w}-1)+\frac{(\textbf{w}+\varepsilon )e^{-2\lambda }}{y}\right) \Sigma '+e^{-2\mu }\Sigma \frac{(\textbf{w}+\varepsilon )(\textbf{w}-1)}{1+\varepsilon }\nonumber \\&\quad + (1+\varepsilon )\left( \frac{1}{3} \Sigma \tilde{G}+ 2\varepsilon \Sigma ^2\right) = 0. \end{aligned}$$

We may rewrite (3.113)–(3.114) in the form

3.115 $$\begin{aligned} \Sigma '&= \frac{2y(1+\varepsilon )\Sigma }{y^2-e^{2\mu -2\lambda }}\left( -\frac{\textbf{w}}{1+\varepsilon }+e^{2\mu }\left( \frac{1}{6}\frac{(1+\varepsilon )\tilde{G}}{\textbf{w}+\varepsilon }+\frac{\varepsilon (1+\varepsilon ) \Sigma }{\textbf{w}+\varepsilon }\right) \right) , \end{aligned}$$

3.116 $$\begin{aligned} \frac{\textbf{w}'}{1+\varepsilon }&= \frac{(\textbf{w}+\varepsilon )\left( 1-3\textbf{w}\right) }{(1+\varepsilon )^2y} - \frac{2y \textbf{w}}{y^2 - e^{2\mu -2\lambda }} \left( -\frac{\textbf{w}}{1+\varepsilon }+e^{2\mu }\left( \frac{1}{6}\frac{(1+\varepsilon )\tilde{G}}{\textbf{w}+\varepsilon }+\frac{\varepsilon (1+\varepsilon ) \Sigma }{\textbf{w}+\varepsilon }\right) \right) . \end{aligned}$$

With (3.105) in mind, equations (3.115)–(3.116) take the form

3.117 $$\begin{aligned} \Sigma '&= - \frac{ 2(1+\varepsilon ) \Sigma K }{y(e^{2\mu - 2\lambda } y^{-2} -1)} , \end{aligned}$$

3.118 $$\begin{aligned} \textbf{w}'&= \frac{(\textbf{w}+\varepsilon )(1 -3\textbf{w})}{(1+\varepsilon )y} + \frac{2(1+\varepsilon )\textbf{w}K}{y(e^{2\mu - 2\lambda } y^{-2} -1)}, \end{aligned}$$

where *K* is given by (3.105) and $\tilde{G}$ by (3.103). Equations (2.29)–(2.30) now follow from (2.26) and parts (b) and (c) of Lemma 3.3. $\square$

### Selfsimilar Schwarzschild Coordinates

We recall the selfsimilar Schwarzschild coordinate introduced in (2.32).

#### Lemma 3.5

(Selfsimilar Schwarzschild formulation) Let $(\Sigma ,\textbf{w})$ be a smooth solution to (3.117)–(3.118). Then the variables $(D,W)$ defined by (2.34) solve the system (2.35)–(2.36), with *B* given by (2.37) and $\eta =\eta (\varepsilon )=\frac{2\varepsilon }{1-\varepsilon }$.

#### Proof

With the above notation and Lemma 3.3 we have $K= \frac{1}{1+\varepsilon }\left( D-W\right)$. It is straightforward to see that the system (3.117)–(3.118) transforms into

3.119 $$\begin{aligned} D'&= - \frac{2(1-\varepsilon )xD(W+\varepsilon )(D-W)}{x^2(W+\varepsilon )^2(e^{2\mu - 2\lambda } y^{-2} -1)} , \end{aligned}$$

3.120 $$\begin{aligned} W'&= \frac{1-3W}{x} + \frac{2(1+\varepsilon )x W(W+\varepsilon )(D-W)}{x^2(W+\varepsilon )^2(e^{2\mu - 2\lambda } y^{-2} -1)}. \end{aligned}$$

From (2.33), the constraint equation (3.95), and (2.27)

3.121 $$\begin{aligned} \frac{(W+\varepsilon )^2}{(1+\varepsilon )^2} x^2e^{2\mu - 2\lambda } y^{-2}&= \tilde{r}'^2 e^{2\mu - 2\lambda } = e^{2\mu }(1+ \varepsilon V^2 - \frac{2}{3}\varepsilon \tilde{G} \tilde{r}^2)\nonumber \\&=e^{2\mu } \left( 1 +\varepsilon e^{-2\mu }x^2 \frac{(W-1)^2}{(1+\varepsilon )^2} - 4 \varepsilon \Sigma W x^2 \right) , \end{aligned}$$

where we have slightly abused notation by letting $\Sigma (x)=\Sigma (y)$. Therefore

3.122 $$\begin{aligned}{} & {} \frac{(W+\varepsilon )^2}{(1+\varepsilon )^2} x^2e^{2\mu - 2\lambda } y^{-2} -\frac{(W+\varepsilon )^2}{(1+\varepsilon )^2} x^2 = \frac{\Sigma ^{-\frac{2\varepsilon }{1+\varepsilon }} }{(1+\varepsilon )^2}\nonumber \\{} & {} \quad - \left[ \frac{(W+\varepsilon )^2}{(1+\varepsilon )^2} - \varepsilon \frac{(W-1)^2}{(1+\varepsilon )^2} + \frac{4\varepsilon }{(1+\varepsilon )^2} \Sigma ^{\frac{1-\varepsilon }{1+\varepsilon }}W\right] x^2, \end{aligned}$$

where we have used (3.101). Plugging this back into (3.119)–(3.119), the claim follows. $\square$

#### Lemma 3.6

(Algebraic structure of the sonic denominator *B*) Consider the denominator $B[x;D,W]$ defined in (2.37). We may factorise *B* in the form

3.123 $$\begin{aligned} B[x;D,W] = (1-\varepsilon ) (J[x;D] -xW(x)) (H[x;D] + xW(x)), \end{aligned}$$

where

3.124 $$\begin{aligned} J[x;D]=J&: = -\frac{2\varepsilon }{1-\varepsilon }(1 +D)x + \sqrt{ \frac{4\varepsilon ^2}{(1-\varepsilon )^2} (1 +D)^2 x^2+ \varepsilon x^2 + \frac{D^{-\eta }}{1-\varepsilon }} , \end{aligned}$$

3.125 $$\begin{aligned} H[x;D]=H&: = J[x;D] +\frac{4\varepsilon }{1-\varepsilon }(1+D)x. \end{aligned}$$

Moreover,

3.126 $$\begin{aligned} (1-\varepsilon )JH = D^{-\eta } + \varepsilon (1-\varepsilon ) x^2, \end{aligned}$$

and

3.127 $$\begin{aligned} J' = \frac{ -2\varepsilon J(1+D) + \varepsilon (1-\varepsilon )x - (2\varepsilon x J+ \frac{\varepsilon }{1-\varepsilon } D^{-\eta -1}) D' }{(1-\varepsilon ) J+ 2\varepsilon x (1+D)}. \end{aligned}$$

#### Proof

From (2.37) it is clear that we may view $B[x;D,W]$ as a quadratic polynomial in *W*:

$$\begin{aligned} W^2 + \frac{4\varepsilon }{1-\varepsilon } (1+D) W - \varepsilon - \frac{ x^{-2} D^{-\eta }}{1-\varepsilon }=0. \end{aligned}$$

Solving for *W*, we obtain two roots, which when multiplied by *x* give

3.128 $$\begin{aligned} xW_\pm = \frac{-2\varepsilon x(1 +D) \pm \sqrt{ 4\varepsilon ^2 x^2(1 +D)^2 + (1-\varepsilon ) \left( \varepsilon (1-\varepsilon ) + D^{-\eta } \right) } }{1-\varepsilon }. \end{aligned}$$

We now observe from (3.124)–(3.125) that the positive solution corresponds to $J[x;D]$ and the negative one to $- H[x;D]$. This in turn immediately gives (3.123). Property (3.126) is obvious from (3.124)–(3.125). Finally, to show (3.127) observe that $J$ solves

$$\begin{aligned} (1-\varepsilon ) J^2 + 4\varepsilon x (1+D) J- \varepsilon (1-\varepsilon ) x^2- D^{-\eta } =0. \end{aligned}$$

We differentiate the above equality, regroup terms and obtain (3.127). $\square$

### The Monotonicity Lemma

Controlling the sonic denominator *B* from below will be one of the central technical challenges in our analysis. From Lemma 3.6 it is clear that this can be accomplished by tracking the quantity $J[x;D]-xW(x)$. A related quantity, of fundamental importance in our analysis is given by

3.129 $$\begin{aligned} f(x) : = J[x;D]- xD. \end{aligned}$$

The goal of the next lemma is to derive a first order ODE satisfied by *f*, assuming that we have a smooth solution to the system (2.35)–(2.36). This lemma will play a central role in our analysis.

#### Lemma 3.7

Let $(D,W)$ be a smooth solution to the selfsimilar Einstein-Euler system (2.35)–(2.36) on some interval $I\subset (0,\infty )$, and let *f* be given by (3.129). Then, the function *f* satisfies the ODE

3.130 $$\begin{aligned} f'(x) + a[x;D,W] f(x) = b[x;D,W], \quad x\in I, \end{aligned}$$

where

$$\begin{aligned} a[x;D,W]=a_1[x;D,W] + a_2[x;D,W], \quad b[x;D,W]=b_1[x;D,W] + \varepsilon b_2[x;D,W] \end{aligned}$$

and

3.131 $$\begin{aligned} a_1[x;D,W]&= \left( \frac{2\varepsilon x J+ \frac{\varepsilon }{1-\varepsilon } D^{-\eta -1}}{(1-\varepsilon )J+2\varepsilon x\left( 1+D\right) }+x\right) \frac{2(1-\varepsilon )D\left( W+\varepsilon \right) }{B} \nonumber \\&\quad -2\varepsilon \left( \frac{1}{1-\varepsilon }\frac{ D^{-\eta }}{x} + (1-\varepsilon )x + (D+\varepsilon )f \right) Z^{-1}; \end{aligned}$$

3.132 $$\begin{aligned} a_2[x;D,W]&= 2\varepsilon \left[ \left( J-xW\right) \left( D-1\right) + 2f + 4\varepsilon x + xD\left( 5+\varepsilon \right) \right] Z^{-1}; \end{aligned}$$

3.133 $$\begin{aligned} b_1[x;D,W]&= \frac{D}{H+xW}(xW-J) + \varepsilon (xW-J)Z^{-1}\left( x\left( 2D^2-2D+1-\varepsilon \right) \right. \nonumber \\&\quad \left. +\frac{2}{(1-\varepsilon )x}D^{-\eta }\right) ; \end{aligned}$$

3.134 $$\begin{aligned} b_2[x,D,W]&= - 2 x^2\Big \{D\left[ D^2+(3+\varepsilon )D-(1-\varepsilon )\right] \nonumber \\&\quad +\varepsilon \left[ \frac{4}{1-\varepsilon }D^3+\frac{2(1+\varepsilon )}{1-\varepsilon }D(1+D) + \frac{5-\varepsilon }{1-\varepsilon }D^2+3D- 2+\varepsilon \right] \Big \}Z^{-1}; \end{aligned}$$

3.135 $$\begin{aligned} Z[x;D,W]&= \left[ (1-\varepsilon )J+ 2\varepsilon x \left( 1+D\right) \right] \left[ H+xW\right] . \end{aligned}$$

#### Proof

Since $f'= J' - xD'-D$, the goal is to find the desirable form (3.130) by using the dynamics of $J$ and $D$ (3.127) and (2.35). The factorisation of the denominator *B* in terms of $J$ and *H* given in (3.123) will be importantly used in the derivation.

Using (3.123) and $xD-xW= xD-J+J-xW$, we first rewrite $D'$ as

$$\begin{aligned} D' = - \frac{2x(1-\varepsilon ) D(W+ \varepsilon ) (D-W)}{B} = \frac{2(1-\varepsilon ) D(W+ \varepsilon ) }{B} f - \frac{2 D(W+ \varepsilon )}{H + xW} . \end{aligned}$$

Using further (3.127), it leads to

3.136 $$\begin{aligned} f'&=-\Big ( \frac{ 2\varepsilon x J+ \frac{\varepsilon }{1-\varepsilon } D^{-\eta -1} }{(1-\varepsilon ) J+ 2\varepsilon x (1+D) } +x \Big )\Big ( \frac{2(1-\varepsilon ) D(W+ \varepsilon ) }{B} \Big ) f \end{aligned}$$

3.137 $$\begin{aligned}&\quad - \left( \frac{ 2\varepsilon J(1+D) - \varepsilon (1-\varepsilon )x - (2\varepsilon x J+ \frac{\varepsilon }{1-\varepsilon } D^{-\eta -1}) \frac{2 D(W+ \varepsilon )}{H + xW} }{(1-\varepsilon ) J+ 2\varepsilon x (1+D) } \right) \end{aligned}$$

3.138 $$\begin{aligned}&\quad - \left( -x \frac{2 D(W+\varepsilon )}{H + xW} + D\right) . \end{aligned}$$

(3.136) is the form of $-a f$, and it corresponds to the first term of $a_1$ in (3.131).

We next examine (3.138). Using (3.125), we see that

3.139 $$\begin{aligned} - \,\text {(3.138)}&= \frac{D}{H+xW}\left( -2x (W + \varepsilon ) + H + xW\right) \nonumber \\&= \frac{D}{H+xW}\left( -2x (W +\varepsilon ) + J+ \frac{4\varepsilon }{1-\varepsilon } (1 + D) x + xW \right) \nonumber \\&=\frac{D}{H+xW}\left( J- xW\right) + \frac{D}{H+xW}\left( 2\varepsilon \frac{1+\varepsilon }{1-\varepsilon }x + \frac{4\varepsilon }{1-\varepsilon } Dx \right) \nonumber \\&{=}{:} I_1 + I_2. \end{aligned}$$

Then the first term $I_1$ corresponds to the first term of $b_1$ in (3.133). The second term $I_2$ will be combined together with the second line (3.137).

Let us rewrite $I_2$-(3.137) as

3.140 $$\begin{aligned} I_2-\text {(3.137)} = \frac{I_3}{ Z } \end{aligned}$$

where *Z* is given in (3.135) and the numerator $I_3$ reads as

3.141 $$\begin{aligned} I_3&= D( (1-\varepsilon ) J+ 2\varepsilon x (1+D))( 2\varepsilon \tfrac{1+\varepsilon }{1-\varepsilon }x + \tfrac{4\varepsilon }{1-\varepsilon } Dx ) +2\varepsilon J(1+D) (H+xW) \end{aligned}$$

3.142 $$\begin{aligned}&\quad - 2 (W + \varepsilon )(2\varepsilon x DJ+ \tfrac{\varepsilon }{1-\varepsilon } D^{-\eta }) -\varepsilon (1-\varepsilon )x (H+xW) \end{aligned}$$

3.143 $$\begin{aligned}&=D( (1-\varepsilon ) J+ 2\varepsilon x (1+D))(2\varepsilon \tfrac{1+\varepsilon }{1-\varepsilon }x + \tfrac{4\varepsilon }{1-\varepsilon } Dx ) +2\varepsilon J(1+D) (H+J) \end{aligned}$$

3.144 $$\begin{aligned}&\quad - 2 (\tfrac{J}{x} +\varepsilon )(2\varepsilon x DJ+ \tfrac{\varepsilon }{1-\varepsilon } D^{-\eta }) - \varepsilon (1-\varepsilon ) x (H+J)\end{aligned}$$

3.145 $$\begin{aligned}&\quad - 2 (W- \frac{J}{x})(2\varepsilon x DJ+ \tfrac{\varepsilon }{1-\varepsilon } D^{-\eta }) - \varepsilon (1-\varepsilon )x (xW-J) + 2\varepsilon J(1+D) (xW-J) . \end{aligned}$$

We first observe that (3.145) can be written into the form of $a f - b$:

3.146 $$\begin{aligned} \text {(3.145)}&=2\varepsilon (J-xW) ( D-1 ) f \end{aligned}$$

3.147 $$\begin{aligned}&\quad + \varepsilon (J-xW) \left( x\left( 2D^2 - 2D+ 1-\varepsilon \right) + \tfrac{2}{1-\varepsilon } \tfrac{D^{-\eta }}{x} \right) \end{aligned}$$

where the first line corresponds to the first term of $a_2$ in (3.132) and the second line corresponds to the second term of $b_1$ in (3.133).

We next examine (3.143) and (3.144). Using (3.125) to replace *H*, and (3.126) to replace $D^{-\eta }$, and writing $J= f + xD$ to replace $J$, we arrive at

3.148 $$\begin{aligned} \text {(3.143)} +&\text {(3.144)} =\tilde{a} f \end{aligned}$$

3.149 $$\begin{aligned}&+D( (1-\varepsilon ) xD+ 2\varepsilon x (1+D))(2\varepsilon \tfrac{1+\varepsilon }{1-\varepsilon }x + \tfrac{4\varepsilon }{1-\varepsilon } Dx ) \end{aligned}$$

3.150 $$\begin{aligned}&+ 4\varepsilon x^2D^2 (1+D) + \tfrac{8\varepsilon ^2}{1-\varepsilon } x^2 D(1+D)^2 \end{aligned}$$

3.151 $$\begin{aligned}&+2\varepsilon ^2 x^2(D+\varepsilon ) \end{aligned}$$

3.152 $$\begin{aligned}&- 2 (D+\varepsilon ) (2\varepsilon x^2 D^2 + \varepsilon x^2D^2 + \tfrac{4\varepsilon ^2}{1-\varepsilon }(1 +D)x^2D) \end{aligned}$$

3.153 $$\begin{aligned}&-\varepsilon (1-\varepsilon )x (2xD+ \tfrac{4\varepsilon }{1-\varepsilon }(1+D)x ), \end{aligned}$$

where

3.154 $$\begin{aligned} \tilde{a}&= D(1-\varepsilon ) (2\varepsilon \tfrac{1+\varepsilon }{1-\varepsilon }x + \tfrac{4\varepsilon }{1-\varepsilon } Dx ) \end{aligned}$$

3.155 $$\begin{aligned}&\quad + 2\varepsilon (1+D) \left[ 2 f + 2x D+ (2xD+ \tfrac{4\varepsilon }{1-\varepsilon } (1+D) x) \right] \end{aligned}$$

3.156 $$\begin{aligned}&\quad - 2 \tfrac{1}{x} \left[ 2\varepsilon xDf + 2\varepsilon x^2 D^2+ \tfrac{\varepsilon }{1-\varepsilon } D^{-\eta } \right] \end{aligned}$$

3.157 $$\begin{aligned}&\quad -2(D+\varepsilon ) \varepsilon \left[ 2xD+ f +(2xD+ \tfrac{4\varepsilon }{1-\varepsilon } (1+D) x) \right] \end{aligned}$$

3.158 $$\begin{aligned}&\quad -2\varepsilon (1-\varepsilon ) x \end{aligned}$$

3.159 $$\begin{aligned}&= -2\varepsilon \left( \tfrac{1}{(1-\varepsilon )x} D^{-\eta } + (1-\varepsilon ) x + (D+ \varepsilon )f \right) \end{aligned}$$

3.160 $$\begin{aligned}&\quad + 2\varepsilon \left[ 2 f+ xD(5+\varepsilon ) + 4\varepsilon x \right] . \end{aligned}$$

Now (3.159) corresponds to the second term of $a_1$ in (3.131) and (3.160) corresponds to the last three terms of $a_2$ in (3.132). It remains to check the formula for $b_2$. To this end, we now group (3.149)–(3.153) into $\varepsilon$ term and $\varepsilon ^2$ terms:

3.161 $$\begin{aligned}&\hbox {(3.149)}+\cdots +\hbox {(3.153)}\end{aligned}$$

3.162 $$\begin{aligned}&= \Big [ 2\varepsilon (1+\varepsilon ) x^2 D^2 + 4\varepsilon D^3 x^2 + 4\varepsilon ^2\tfrac{1+\varepsilon }{1-\varepsilon } x^2 D(1+ D) + \tfrac{8\varepsilon ^2}{1-\varepsilon } x^2 D^2 (1+D) \end{aligned}$$

3.163 $$\begin{aligned}&\quad + 4\varepsilon x^2 D^2 (1 + D) + \tfrac{8\varepsilon ^2}{1-\varepsilon }x^2 D(1 + D)^2 + 2\varepsilon ^2 x^2 (D+ \varepsilon ) \Big ]\end{aligned}$$

3.164 $$\begin{aligned}&\quad - \Big [ 6\varepsilon x^2 D^3 +6\varepsilon ^2 x^2 D^2 + \tfrac{8\varepsilon ^2}{1-\varepsilon }x^2 D^2 (1+D)+ \tfrac{8\varepsilon ^3}{1-\varepsilon }x^2 D(1+D) \end{aligned}$$

3.165 $$\begin{aligned}&\quad +2\varepsilon (1-\varepsilon ) x^2 D+4\varepsilon ^2 x^2 (1+D) \Big ] \end{aligned}$$

3.166 $$\begin{aligned}&= \big [8\varepsilon x^2 D^3 + 2\varepsilon x^2D^2 ( 3+\varepsilon )\big ] -\big [6\varepsilon x^2 D^3 + 2\varepsilon (1-\varepsilon ) x^2 D\big ] \nonumber \\&\quad + \big [ 4\varepsilon ^2\tfrac{1+\varepsilon }{1-\varepsilon } x^2 D(1+ D) + \tfrac{8\varepsilon ^2}{1-\varepsilon }x^2 D^3 + \tfrac{16 \varepsilon ^2}{1-\varepsilon } x^2 D^2 + 2\varepsilon ^2 (\tfrac{4}{1-\varepsilon } +1)x^2D+2\varepsilon ^3 x^2 \big ]\nonumber \\&\quad - \big [ (6\varepsilon ^2 + \tfrac{8\varepsilon ^3}{1-\varepsilon } )x^2 D^2 + (\tfrac{8\varepsilon ^3}{1-\varepsilon } + 4\varepsilon ^2) x^2 D+ 4\varepsilon ^2 x^2 \big ]\nonumber \\&= 2\varepsilon x^2D\left[ D^2 + (3+\varepsilon ) D- (1-\varepsilon ) \right] \end{aligned}$$

3.167 $$\begin{aligned}&\quad + 2\varepsilon ^2x^2\left[ \tfrac{4}{1-\varepsilon } D^3+ \tfrac{2(1+\varepsilon )}{1-\varepsilon } D(1+D) + \tfrac{5-\varepsilon }{1-\varepsilon } D^2 + 3 D- 2+\varepsilon \right] . \end{aligned}$$

This completes the proof. $\square$

#### Corollary 3.8

Let $(D,W)$ be a smooth solution to the selfsimilar Einstein-Euler system (2.35)–(2.36) on some interval $I\subset (0,\infty )$. Then for any $x_1<x$, $x_1,x\in I$, we have the formula

3.168 $$\begin{aligned} f(x) =&f(x_1) e^{-\int _{x_1}^x a[z;D,W]\,dz} \nonumber \\&+e^{-\int _{x_1}^x a[z;D,W]\,dz} \int _{x_1}^x \left( b_1[z;D,W] + \varepsilon b_2[z;D,W]\right) e^{\int _{x_1}^z a[s;D,W]\,ds}\,dz. \end{aligned}$$

#### Proof

The proof follows by applying the integrating factor method to (3.130). $\square$

#### Lemma 3.9

(Sign properties of *b*) Assume that $J-xW>0$ and $B[x;D,W]>0$. Then there exists an $\varepsilon _0>0$ sufficiently small such that for all $0<\varepsilon \le \varepsilon _0$ the following statements hold:

*(a)*: For any $$D>0$$
  3.169 $$\begin{aligned} b_1[x;D,W]<0. \end{aligned}$$
*(b)*: Furthermore,
  3.170 $$\begin{aligned} b_2[x;D,W] <0 \quad \text { for all } \; D>\frac{1}{3}. \end{aligned}$$

#### Proof

The assumptions of the lemma and the decomposition (3.123) imply that $Z>0$ and $H+xW>0$.

*Proof of part (a).* The negativity of $b_1$ is obvious from (3.133) and the obvious bound $2D^2-2D+1-\varepsilon >0$ for $\varepsilon$ sufficiently small.

*Proof of part (b).* Let $\varphi _0$ be the larger of the two roots of the quadratic polynomial $D\mapsto D^2+(3+\varepsilon )D-(1-\varepsilon )$, which is given by

$$\begin{aligned} \varphi _0{:}{=}\frac{-(3+\varepsilon ) +\sqrt{(3+\varepsilon )^2+ 4(1-\varepsilon )}}{2}. \end{aligned}$$

It is easily checked that there exists an $\varepsilon _0>0$ such that $0<\varphi _0<\frac{1}{3}$ for all $0<\varepsilon \le \varepsilon _0$ and in particular

$$\begin{aligned}D\left[ D^2+(3+\varepsilon )D-(1-\varepsilon )\right] >0\end{aligned}$$

for $D>\frac{1}{3} (>\varphi _0)$. On the other hand,

$$\begin{aligned} \frac{4}{1-\varepsilon }D^3+\frac{2(1+\varepsilon )}{1-\varepsilon }D(1+D) + \frac{5-\varepsilon }{1-\varepsilon }D^2+3D- 2+\varepsilon> 4 D^3 + 6D^2 +5D-2>0 \end{aligned}$$

for $D>\frac{1}{3}$ and the claim follows from (3.134). $\square$

## The Sonic Point Analysis

It turns out that for purposes of homogeneity, it is more convenient to work with rescaled unknowns where the sonic point is pulled-back to a fixed value 1. Namely, we introduce the change of variables:

4.171 $$\begin{aligned} z= \frac{x}{x_*}, \quad \mathcal {W}(z)=W(x), \quad \mathcal {R}(z) = D(x). \end{aligned}$$

so that the sonic point $x_*$ is mapped to $z=1$. It is then easily checked from (2.35)–(2.36) that $(\mathcal {R},\mathcal {W})$ solves

4.172 $$\begin{aligned} \frac{d \mathcal {R}}{dz}&= - \frac{ 2x_*^2 z(1-\varepsilon ) \mathcal {R}( \mathcal {W}+\varepsilon ) ( \mathcal {R}- \mathcal {W}) }{B} , \end{aligned}$$

4.173 $$\begin{aligned} \frac{d \mathcal {W}}{dz}&= \frac{(1-3\mathcal {W})}{z} + \frac{2x_*^2 z(1+\varepsilon ) \mathcal {W}( \mathcal {W}+ \varepsilon ) ( \mathcal {R}- \mathcal {W})}{B}, \end{aligned}$$

where

4.174 $$\begin{aligned} B = \mathcal {R}^{-\eta } -\left[ ( \mathcal {W}+ \varepsilon )^2 - \varepsilon ( \mathcal {W}- 1)^2 + 4\varepsilon \mathcal {R}\mathcal {W}\right] x_*^2z^2. \end{aligned}$$

We introduce

4.175 $$\begin{aligned} \delta z{:}{=}\, z-1. \end{aligned}$$

we look for solutions $\mathcal {R},\mathcal {W}$ to (4.172)-(4.173) of the form

4.176 $$\begin{aligned} \mathcal {R}= \sum _{N=0}^\infty \mathcal {R}_N (\delta z)^N, \quad \mathcal {W}= \sum _{N=0}^\infty \mathcal {W}_N (\delta z)^N. \end{aligned}$$

We observe that there is a simple relation between the formal Taylor coefficients of $(D,W)$ and $(\mathcal {R},\mathcal {W})$:

4.177 $$\begin{aligned} \mathcal {R}_j = D_j x_*^j, \quad \mathcal {W}_j = W_j x_*^j, \quad j=0,1,2,\dots . \end{aligned}$$

### Sonic Point Conditions

#### Lemma 4.1

(Sonic conditions) There exists a small $\varepsilon _0>0$ such that for all $|\varepsilon |\le \varepsilon _0$ and all $x_*\in [\frac{3}{2}, \frac{7}{2}]$ there exists a continuously differentiable curve $[-\varepsilon _0,\varepsilon _0]\ni \varepsilon \mapsto (\mathcal {R}_0(\varepsilon ),\mathcal {W}_0(\varepsilon ))=(\mathcal {R}_0(\varepsilon ;x_*),\mathcal {W}_0(\varepsilon ;x_*))$ such that

1. $$\mathcal {R}_0(\varepsilon )=\mathcal {W}_0(\varepsilon )>0$$.
2. $$B[\mathcal {R}_0(\varepsilon ),\mathcal {W}_0(\varepsilon )]=0$$.
3. $$\mathcal {R}_0(0)=\mathcal {W}_0(0) = \frac{1}{x_*}$$.
4. $$-\infty<\partial _\varepsilon \mathcal {R}_0(0)=\partial _\varepsilon \mathcal {W}_0(0)<0$$.

#### Proof

Fix an $x_*\in [\frac{3}{2},\frac{7}{2}]$. Consider a small neighbourhood of $(\varepsilon ,\mathcal {W})=(0,\frac{1}{x_*})$, open rectangle $(\varepsilon ,\mathcal {W})\in (-l,l)\times (w_1,w_2)$, and a continuously differentiable function $h:(-l,l)\times (w_1,w_2) \rightarrow \mathbb R$ defined by

4.178 $$\begin{aligned} h(\varepsilon ,\mathcal {W}){:}{=} \left[ (1+3\varepsilon ) \mathcal {W}^2 + 4\varepsilon \mathcal {W}+\varepsilon (\varepsilon -1)\right] \mathcal {W}^{\eta } - \frac{1}{x_*^2}, \end{aligned}$$

where we recall $\eta =\eta (\varepsilon )=\frac{2\varepsilon }{1-\varepsilon }$. Then the sonic point conditions $\mathcal {R}=\mathcal {W}$ and $B=0$ with $z=1$ reduce to $h(\varepsilon ,\mathcal {W})=0$ and moreover we have $h(0,\frac{1}{x_*})=0$. Clearly *h* is continuously differentiable in all arguments. Observe that

4.179 $$\begin{aligned} \frac{\partial h}{\partial \mathcal {W}} = \left[ 2(1+3\varepsilon ) \mathcal {W}^2 + 4\varepsilon \mathcal {W}+\eta \left( (1+3\varepsilon ) \mathcal {W}^2 + 4\varepsilon \mathcal {W}+ \varepsilon (\varepsilon -1) \right) \right] \mathcal {W}^{\eta -1} \end{aligned}$$

from which we have

4.180 $$\begin{aligned} \frac{\partial h}{\partial \mathcal {W}} \Big |_{(\varepsilon ,\mathcal {W})=(0,\frac{1}{x_*})} = \frac{2}{x_*} >0 . \end{aligned}$$

Therefore, by the implicit function theorem, we deduce that there exists an open interval $(-l_0,l_0)$ of $\varepsilon =0$ and a unique continuously differential function $g:(-l_0,l_0) \rightarrow (w_1,w_2)$ such that $g(0)=\frac{1}{x_*}$ and $h(\varepsilon , g(\varepsilon )) =0$ for all $\varepsilon \in (-l_0,l_0)$. Moreover, we have

4.181 $$\begin{aligned} \frac{\partial g}{\partial \varepsilon } = - \frac{\frac{\partial h}{\partial \varepsilon }}{\frac{\partial h}{\partial \mathcal {W}}}, \quad \varepsilon \in (-l_0,l_0) \end{aligned}$$

where

4.182 $$\begin{aligned} \frac{\partial h}{\partial \varepsilon }= \left[ 3 \mathcal {W}^2 + 4\mathcal {W}+ 2\varepsilon -1+ \left( (1+3\varepsilon ) \mathcal {W}^2 + 4\varepsilon \mathcal {W}+\varepsilon (\varepsilon -1)\right) \frac{2\ln \mathcal {W}}{(1-\varepsilon )^2} \right] \mathcal {W}^{\eta }. \end{aligned}$$

When $$\varepsilon =0$$,

4.183 $$\begin{aligned} \begin{aligned} \frac{\partial g}{\partial \varepsilon }(0) = - \frac{\frac{\partial h}{\partial \varepsilon }|_{(0,\frac{1}{x_*})}}{\frac{\partial h}{\partial \mathcal {W}}|_{(0,\frac{1}{x_*})}}&=- \frac{\frac{3}{x_*^2} + \frac{4}{x_*} -1 -\frac{2\ln x_*}{x_*^2}}{\frac{2}{x_*}} = \frac{x_*^2 - 4x_*+2\log x_*-3}{2x_*}. \end{aligned} \end{aligned}$$

The derivative of the map $x_*\rightarrow x_*^2 - 4x_*+2\log x_*-3$ is $2\frac{(x_*-1)^2}{x_*}$ and the function is therefore strictly increasing for $x_*\ne 1$. It is easy to check that the value at $x_*=\frac{7}{2}$ is negative, and therefore there exists a constant $\kappa >0$ such that $\frac{\partial g}{\partial \varepsilon }(0)<-\kappa$ for all $x_*\in [\frac{3}{2},\frac{7}{2}]$. In particular, there exists a $0<\varepsilon _0\ll 1$ sufficiently small and a constant $c_*>0$ such that

$$\begin{aligned} \frac{1}{x_*}-c_*\varepsilon< g(\varepsilon ;x_*)< \frac{1}{x_*}, \quad \varepsilon \in (0,\varepsilon _0], \quad x_*\in \left[ \frac{3}{2},\frac{7}{2}\right] . \end{aligned}$$

We let

4.184 $$\begin{aligned} \mathcal {R}_0{:}{=} \mathcal {R}(\varepsilon )=g(\varepsilon ), \quad \mathcal {W}_0{:}{=} \mathcal {W}_0(\varepsilon ) = g(\varepsilon ). \end{aligned}$$

$$\square $$

#### Remark 4.2

(The map $x_*\mapsto \mathcal {W}_0(\varepsilon ;x_*)$ is decreasing) In order to examine the behaviour of $\mathcal {W}_0(\varepsilon ; x_*)=g(\varepsilon ;x_*)$ as a function of $x_*$ for any fixed $\varepsilon$, we rewrite the relation $h(\varepsilon ,\mathcal {W}_0(\varepsilon ))=0$ in the form

$$\begin{aligned} h(\varepsilon ,g(\varepsilon ;x_*);x_*)=0. \end{aligned}$$

Upon taking the $\frac{\partial }{\partial x_*}$ derivative of the above, we easily see that $\partial _{x_*}g(\varepsilon ;x_*)<0$. In fact, we have $\partial _{x_*}g = - \frac{\partial _{x_*} h}{\partial _{\mathcal {W}} h}$ where $\partial _{x_*} h = \frac{2}{x_*^3}>0$ from (4.178), and $\partial _{\mathcal {W}} h>0$ is given in (4.179).

For a given function *f*, we write $(f)_M$, $M\in \mathbb N$, to denote the *M*-th Taylor coefficient in the expansion of *f* around the sonic point $z = 1$. In particular,

$$\begin{aligned} (\mathcal {R}( \mathcal {W}+ \varepsilon ) ( \mathcal {R}- \mathcal {W}))_M&= \sum _{l+m+n=M} \mathcal {R}_l ( \mathcal {W}_m + \varepsilon \delta _{m}^{0}) ( \mathcal {R}_n- \mathcal {W}_n), \\ (\mathcal {W}( \mathcal {W}+ \varepsilon )( \mathcal {R}- \mathcal {W}))_M&= \sum _{l+m+n=M} \mathcal {W}_l ( \mathcal {W}_m + \varepsilon \delta _{m}^{0}) ( \mathcal {R}_n- \mathcal {W}_n),\\ (\mathcal {W}^2)_M&= \sum _{l+m=M} \mathcal {W}_l \mathcal {W}_m, \\ (\mathcal {R}\mathcal {W})_M&= \sum _{l+m=M} \mathcal {R}_l \mathcal {W}_m. \end{aligned}$$

We set $(f)_M=0$ for $M<0$.

*Formula of Faa Di Bruno.* Given two functions *f*, *g* with formula power series expansions

4.185 $$\begin{aligned} f(x)=\sum _{n=0}^\infty {f_n} x^n, \quad g(x)=\sum _{n=1}^\infty {g_n} x^n, \end{aligned}$$

we can compute the formal Taylor series expansion of the composition $h=f \circ g$ via

4.186 $$\begin{aligned} h(x) =\sum _{n=0}^\infty {h_n} x^n \end{aligned}$$

where

4.187 $$\begin{aligned} h_n = \sum _{m=1}^n \sum _{\pi (n,m)} \frac{m !}{\lambda _1 !\dots \lambda _n !} f_m \left( {g_1} \right) ^{\lambda _1}\dots \left( {g_n}\right) ^{\lambda _n}, \quad h_0= f_0 \end{aligned}$$

and

4.188 $$\begin{aligned} \pi (n,m)= \left\{ (\lambda _1, \dots ,\lambda _n): \lambda _i \in \mathbb Z_{\ge 0}, \sum _{i=1}^n \lambda _i =m, \sum _{i=1}^n i \lambda _i =n \right\} . \end{aligned}$$

An element of $\pi (n,m)$ encodes the partitions of the first *n* numbers into $\lambda _i$ classes of cardinality *i* for $i\in \{1,\dots ,m\}$. Observe that by necessity

$$\begin{aligned} \lambda _j=0 \; \text { for } \ n-m+2\le j\le n. \end{aligned}$$

To see this, suppose $\lambda _j = p\ge 1$ for some $n-m+2\le j\le n$. Then $m - p=\sum _{i\ne j} \lambda _i \le \sum _{i\ne j} i\lambda _i = n-j p \le n- (n-m+2) p$, which leads to $(n-m+1)p \le n-m$. But this is impossible if $p\ge 1$.

Now

4.189 $$\begin{aligned} \begin{aligned} \mathcal {R}^{-\eta }&= \mathcal {R}_0^{-\eta } \left( 1+ \sum _{i=1}^\infty \frac{\mathcal {R}_i}{\mathcal {R}_0} (\delta z)^i \right) ^{-\eta }= \mathcal {R}_0^{-\eta }+ \sum _{j=1}^\infty (\mathcal {R}^{-\eta } )_j (\delta z)^j \end{aligned} \end{aligned}$$

where

4.190 $$\begin{aligned} (\mathcal {R}^{-\eta } )_j =\mathcal {R}_0^{-\eta } \sum _{m=1}^j \frac{1}{\mathcal {R}_0^m}\sum _{\pi (j,m)} (- \eta )_m \frac{1}{\lambda _1 ! \dots \lambda _j !} {\mathcal {R}_1}^{\lambda _1}\dots {\mathcal {R}_j}^{\lambda _j}, \quad j \ge 1. \end{aligned}$$

Here $(-\eta )_m = (-\eta )(-\eta -1) \cdots (-\eta -m+1)$. Then we may write *B* as

4.191 $$\begin{aligned} B&= \mathcal {R}^{-\eta } -\left[ (1-\varepsilon )\mathcal {W}^2 + 4\varepsilon \mathcal {W}+ 4\varepsilon \mathcal {R}\mathcal {W}+ \varepsilon ^2-\varepsilon \right] x_*^2z^2 \end{aligned}$$

4.192 $$\begin{aligned}&=: \mathcal {R}^{-\eta } - x_*^2 Hz^2\end{aligned}$$

4.193 $$\begin{aligned}&= \sum _{l=0}^\infty (\mathcal {R}^{-\eta })_l (\delta z)^l - x_*^2 \sum _{l=0}^\infty H_l (\delta z)^l(1+ 2 \delta z + (\delta z)^2) \end{aligned}$$

so that

4.194 $$\begin{aligned} H_l = (1-\varepsilon )(\mathcal {W}^2)_l + 4\varepsilon \mathcal {W}_l+ 4\varepsilon ( \mathcal {R}\mathcal {W})_l+ (\varepsilon ^2-\varepsilon )\delta ^0_l . \end{aligned}$$

#### Lemma 4.3

For any $N\ge 0$, the following formulas hold:

4.195 $$\begin{aligned} \begin{aligned}&\sum _{l+m=N} (m+1) \mathcal {R}_{m+1} (\mathcal {R}^{-\eta })_l \\&- x_*^2 \left( \sum _{l+m=N} (m+1) \mathcal {R}_{m+1} H_l + 2\sum _{l+m=N-1} (m+1) \mathcal {R}_{m+1}H_l +\sum _{l+m=N-2} (m+1) \mathcal {R}_{m+1}H_l \right) \\&+ 2x_*^2(1-\varepsilon )\left( ( \mathcal {R}( \mathcal {W}+ \varepsilon ) ( \mathcal {R}- \mathcal {W}) )_N +( \mathcal {R}( \mathcal {W}+ \varepsilon ) ( \mathcal {R}- \mathcal {W}) )_{N-1} \right) =0 \end{aligned} \end{aligned}$$

and

4.196 $$\begin{aligned} \begin{aligned}&\sum _{l+m=N} (m+1) \mathcal {W}_{m+1} (\mathcal {R}^{-\eta })_l \\&- x_*^2 \left( \sum _{l+m=N} (m+1) \mathcal {W}_{m+1} H_l + 2\sum _{l+m=N-1} (m+1) \mathcal {W}_{m+1}H_l +\sum _{l+m=N-2} (m+1) \mathcal {W}_{m+1}H_l \right) \\&- \sum _{l+m=N} (\mathcal {R}^{-\eta })_l (-1)^m + 3\sum _{l+m+n=N} \mathcal {W}_n (\mathcal {R}^{-\eta })_l (-1)^m \\&+ x_*^2 \left( \sum _{l+m=N} H_l (-1)^m +2 \sum _{l+m=N-1} H_l (-1)^m + \sum _{l+m=N-2}H_l (-1)^m \right) \\&-3x_*^2 \left( \sum _{l+m+n=N} \mathcal {W}_n H_l (-1)^m +2 \sum _{l+m+n=N-1} \mathcal {W}_nH_l (-1)^m + \sum _{l+m+n=N-2}\mathcal {W}_n H_l (-1)^m \right) \\&- 2x_*^2(1+\varepsilon )\left( ( \mathcal {W}( \mathcal {W}+ \varepsilon ) ( \mathcal {R}- \mathcal {W}) )_N +( \mathcal {W}( \mathcal {W}+ \varepsilon ) ( \mathcal {R}- \mathcal {W}) )_{N-1} \right) =0 \end{aligned} \end{aligned}$$

where we recall $(f)_M=0$ for $M<0$.

#### Proof

*Proof of* (4.195). We now plug (4.176) into

4.197 $$\begin{aligned} B \mathcal {R}'&+ 2x_*^2(1-\varepsilon ) (1+\delta z) \mathcal {R}( \mathcal {W}+ \varepsilon ) ( \mathcal {R}- \mathcal {W}) =0, \end{aligned}$$

4.198 $$\begin{aligned} B \mathcal {W}'&- \frac{(1 -3 \mathcal {W}) B}{1+\delta z} - 2x_*^2 (1+\varepsilon ) (1+\delta z) \mathcal {W}( \mathcal {W}+ \varepsilon ) ( \mathcal {R}- \mathcal {W})=0. \end{aligned}$$

(4.197) reads as

$$\begin{aligned} 0&= \left[ \sum _{l=0}^\infty (\mathcal {R}^{-\eta })_l (\delta z)^l - x_*^2 \sum _{l=0}^\infty H_l (\delta z)^l(1+ 2 \delta z + (\delta z)^2)\right] \left[ \sum _{m=0}^\infty (m+1) \mathcal {R}_{m+1} (\delta z)^m \right] \\&\quad + 2x_*^2(1-\varepsilon ) \sum _{l=0}^\infty ( \mathcal {R}( \mathcal {W}+ \varepsilon ) ( \mathcal {R}- \mathcal {W}) )_l (\delta z)^l (1+\delta z) \\&= \sum _{N=0}^\infty \sum _{l+m=N} (m+1) \mathcal {R}_{m+1} (\mathcal {R}^{-\eta })_l (\delta z)^N \\&\quad - x_*^2 \sum _{N=0}^\infty \left( \sum _{l+m=N} (m+1) \mathcal {R}_{m+1} H_l \right. \\&\quad \left. + 2\sum _{l+m=N-1} (m+1) \mathcal {R}_{m+1}H_l +\sum _{l+m=N-2} (m+1) \mathcal {R}_{m+1}H_l \right) (\delta z)^N\\&\quad + 2x_*^2(1-\varepsilon ) \sum _{N=0}^\infty \left( ( \mathcal {R}( \mathcal {W}+ \varepsilon ) ( \mathcal {R}- \mathcal {W}) )_N +( \mathcal {R}( \mathcal {W}+ \varepsilon ) ( \mathcal {R}- \mathcal {W}) )_{N-1} \right) (\delta z)^N . \end{aligned}$$

Comparing the coefficients, we obtain (4.195).

*Proof of* (4.196). Since $\frac{1}{1+\delta z} = \sum _{m=0}^\infty (-1)^m (\delta z)^m$, we can expand $\frac{(1 -3 \mathcal {W}) B}{1+\delta z}$ as

$$\begin{aligned}&\frac{(1 -3 \mathcal {W}) B}{1+\delta z} \\&\quad = \left( 1 -3\sum _{n=0}^\infty \mathcal {W}_n (\delta z)^n \right) \left( \sum _{l=0}^\infty (\mathcal {R}^{-\eta })_l (\delta z)^l - x_*^2 \sum _{l=0}^\infty H_l(\delta z)^l (1+ 2 \delta z + (\delta z)^2) \right) \\&\quad \sum _{m=0}^\infty (-1)^m (\delta z)^m\\&\quad = \sum _{N=0}^\infty \sum _{l+m=N} (\mathcal {R}^{-\eta })_l (-1)^m (\delta z)^N - 3 \sum _{N=0}^\infty \sum _{l+m+n=N} \mathcal {W}_n (\mathcal {R}^{-\eta })_l (-1)^m (\delta z)^N \\&\quad - x_*^2 \sum _{N=0}^\infty \left( \sum _{l+m=N} H_l (-1)^m +2 \sum _{l+m=N-1} H_l (-1)^m + \sum _{l+m=N-2}H_l (-1)^m \right) (\delta z)^N\\&\quad +3x_*^2 \sum _{N=0}^\infty \left( \sum _{l+m+n=N} \mathcal {W}_n H_l (-1)^m +2 \sum _{l+m+n=N-1} \mathcal {W}_nH_l (-1)^m + \sum _{l+m+n=N-2}\mathcal {W}_n H_l (-1)^m \right) (\delta z)^N. \end{aligned}$$

We plug (2.43) into (4.198)

$$\begin{aligned} 0&= \left[ \sum _{l=0}^\infty (\mathcal {R}^{-\eta })_l (\delta z)^l - x_*^2 \sum _{l=0}^\infty H_l (\delta z)^l(1+ 2 \delta z + (\delta z)^2)\right] \left[ \sum _{m=0}^\infty (m+1) \mathcal {W}_{m+1} (\delta z)^m \right] \\&\quad - \sum _{N=0}^\infty \left( \frac{(1 -3 \mathcal {W}) B}{1+\delta z} \right) _N (\delta z)^N - 2x_*^2(1+\varepsilon ) \sum _{l=0}^\infty ( \mathcal {W}( \mathcal {W}+ \varepsilon ) ( \mathcal {R}- \mathcal {W}) )_l (\delta z)^l (1+\delta z) \\&= \sum _{N=0}^\infty \sum _{l+m=N} (m+1) \mathcal {W}_{m+1} (\mathcal {R}^{-\eta })_l (\delta z)^N \\&\quad - x_*^2 \sum _{N=0}^\infty \left( \sum _{l+m=N} (m+1) \mathcal {W}_{m+1} H_l \right. \\&\quad \left. + 2\sum _{l+m=N-1} (m+1) \mathcal {W}_{m+1}H_l +\sum _{l+m=N-2} (m+1) \mathcal {W}_{m+1}H_l \right) (\delta z)^N\\&\quad - \sum _{N=0}^\infty \left( \frac{(1 -3 \mathcal {W}) B}{1+\delta z} \right) _N (\delta z)^N \\&\quad - 2x_*^2(1+\varepsilon ) \sum _{N=0}^\infty \left( ( \mathcal {W}( \mathcal {W}+ \varepsilon ) ( \mathcal {R}- \mathcal {W}) )_N +( \mathcal {W}( \mathcal {W}+ \varepsilon ) ( \mathcal {R}- \mathcal {W}) )_{N-1} \right) (\delta z)^N \end{aligned}$$

which leads to (4.196). $\square$

#### Remark 4.4

$(\mathcal {R}_0,\mathcal {W}_0)$ obtained in Lemma 4.1 satisfy the sonic conditions:

4.199 $$\begin{aligned} \mathcal {R}_0=\mathcal {W}_0, \quad \mathcal {R}_0^{-\eta }=x_*^2 H_0 \end{aligned}$$

and it is easy to verify that for such choice of $(\mathcal {R}_0,\mathcal {W}_0)$, the relations (4.195)$_{N=0}$ and (4.196)$_{N=0}$ trivially hold. Moreover, with (4.199), there are no $(\mathcal {R}_{N+1}, \mathcal {W}_{N+1})$ in (4.195) and (4.196).

#### Remark 4.5

To determine $R_1, \mathcal {W}_1$, we record (4.195)$_{N=1}$ and (4.196)$_{N=1}$:

4.200 $$\begin{aligned} \begin{aligned} (\mathcal {R}^{- \eta })_1 \mathcal {R}_1&- x_*^2 H_1 \mathcal {R}_1 - 2x_*^2 H_0 \mathcal {R}_1+ 2x_*^2 (1-\varepsilon ) \mathcal {R}_0(\mathcal {W}_0 + \varepsilon )(\mathcal {R}_1 -\mathcal {W}_1) =0 \end{aligned} \end{aligned}$$

and

4.201 $$\begin{aligned} \begin{aligned}&(\mathcal {R}^{-\eta })_1 \mathcal {W}_1 - x_*^2 (\mathcal {W}_1 H_1+ 2\mathcal {W}_1 H_0) - [ (\mathcal {R}^{-\eta })_1- \mathcal {R}_0^{-\eta }] \\&+ 3 [ \mathcal {W}_1\mathcal {R}_0^{-\eta } + \mathcal {W}_0 (\mathcal {R}^{-\eta })_1 - \mathcal {W}_0 \mathcal {R}_0^{-\eta }] + x_*^2 [H_1+H_0] \\&- 3x_*^2 [\mathcal {W}_1 H_0 + \mathcal {W}_0 H_1 + \mathcal {W}_0 H_0] - 2x_*^2 (1+\varepsilon ) \mathcal {W}_0(\mathcal {W}_0 + \varepsilon )(\mathcal {R}_1 -\mathcal {W}_1) =0, \end{aligned} \end{aligned}$$

where we have used the sonic conditions (4.199). Recalling from (4.190) and (4.194)

4.202 $$\begin{aligned} (\mathcal {R}^{-\eta })_1&= -\eta \mathcal {R}_0^{-\eta -1} \mathcal {R}_1,\end{aligned}$$

4.203 $$\begin{aligned} H_1&= 2\left[ (1+\varepsilon )\mathcal {W}_0 + 2\varepsilon \right] \mathcal {W}_1 + 4\varepsilon \mathcal {W}_0 \mathcal {R}_1, \end{aligned}$$

we see that for general nonzero $\varepsilon$, (4.200) is linear in $\mathcal {W}_1$ and quadratic in $\mathcal {R}_1$ and (4.201) is linear in $\mathcal {R}_1$ and quadratic in $\mathcal {W}_1$. On the other hand, when $\varepsilon =0$, since $(\mathcal {R}^{-\eta })_1 =0$, $H_1= 2\mathcal {W}_0\mathcal {W}_1$, $H_0=\mathcal {W}_0^2$, (4.200) becomes

$$\begin{aligned} \begin{aligned} -2x_*^2 \mathcal {W}_0\mathcal {W}_1 \mathcal {R}_1 - 2x_*^2\mathcal {W}_0^2 \mathcal {R}_1 + 2x_*^2 \mathcal {R}_0\mathcal {W}_0 (\mathcal {R}_1-\mathcal {W}_1)=0, \end{aligned} \end{aligned}$$

and (4.201) becomes

$$\begin{aligned} \begin{aligned} -x_*^2(2\mathcal {W}_0\mathcal {W}_1^2 + 2 \mathcal {W}_1\mathcal {W}_0^2) + 1 + 3[\mathcal {W}_1-\mathcal {W}_0] + x_*^2[2\mathcal {W}_0\mathcal {W}_1+\mathcal {W}_0^2] \\ - 3x_*^2 [\mathcal {W}_1\mathcal {W}_0^2+2\mathcal {W}_0^2\mathcal {W}_1 + \mathcal {W}_0^3] - 2x_*^2\mathcal {W}_0^2 (\mathcal {R}_1-\mathcal {W}_1) =0. \end{aligned} \end{aligned}$$

Thus by using $\mathcal {R}_0\big |_{\varepsilon =0}=\mathcal {W}_0\big |_{\varepsilon =0}=\frac{1}{x_*}$, we see that (4.200) and (4.201) are reduced to

4.204 $$\begin{aligned} -2\mathcal {W}_1 \left( x_*{\mathcal {R}_1} +1 \right) =0, \end{aligned}$$

4.205 $$\begin{aligned} -2 \left( x_*\mathcal {W}_1^2 - x_*\mathcal {W}_1 + 3\mathcal {W}_1 + \mathcal {R}_1 + \frac{3}{x_*} -1\right) =0, \end{aligned}$$

which are the the sonic point conditions satisfied in the Newtonian limit, see [15]. In general, there are two pairs of solutions to (4.204)-(4.205), one is of Larson-Penston type given by $(\mathcal {R}_1,\mathcal {W}_1)=(-\frac{1}{x_*}, 1-\frac{2}{x_*})$ and the other one is of Hunter type given by $(\mathcal {R}_1,\mathcal {W}_1)=(1-\frac{3}{x_*},0)$.

In the following, we will show that there exists a continuously differentiable curve $(\mathcal {R}_1(\varepsilon ),\mathcal {W}_1(\varepsilon ))$ satisfying (4.200)–(4.201), which at $\varepsilon =0$ agrees precisely with the values of $(\mathcal {R}_1,\mathcal {W}_1)$ associated with the (Newtonian) Larson-Penston solution, namely $(\mathcal {R}_1(0),\mathcal {W}_1(0))=(-\frac{1}{x_*}, 1-\frac{2}{x_*})$ for $x_*>2$.

#### Lemma 4.6

(RLP conditions) Let $x_*\in (2, \frac{7}{2})$ be fixed and let $(\mathcal {R}_0,\mathcal {W}_0)$ be as obtained in Lemma 4.1. Then there exists an $\varepsilon _1>0$ such that there exists a continuously differentiable curve $(-\varepsilon _1,\varepsilon _1)\ni \varepsilon \mapsto (\mathcal {R}_1(\varepsilon )$, $\mathcal {W}_1(\varepsilon ))$ such that

1. The relations (4.195)$_{N=1}$ and (4.196)$_{N=1}$ hold.
2. $$\mathcal {R}_1(0)= -\frac{1}{x_*}$$ and $$\mathcal {W}_1(0)= 1-\frac{2}{x_*}$$.

#### Proof

As in Lemma 4.1 we will use the implicit function theorem. To this end, consider a small neighborhood $(-l,l)\times (r_1,r_2)\times (w_1,w_2)$ of $(0, -\frac{1}{x_*}, 1-\frac{2}{x_*})$ and introduce $\mathcal {F}:(-l,l)\times (r_1,r_2)\times (w_1,w_2)\rightarrow \mathbb R^2$ as

4.206 $$\begin{aligned} \mathcal {F}(\varepsilon , \mathcal {R}_1,\mathcal {W}_1) = \left( \mathcal {F}_1 (\varepsilon , \mathcal {R}_1,\mathcal {W}_1),\mathcal {F}_2(\varepsilon , \mathcal {R}_1,\mathcal {W}_1) \right) ^T \end{aligned}$$

where4.207so that (4.200) and (4.201) are equivalent to $\mathcal {F}(\varepsilon , \mathcal {R}_1,\mathcal {W}_1) =0$. It is clear that $\mathcal {F}$ is continuously differentiable in all arguments and $\mathcal {F} (0, -\frac{1}{x_*}, 1-\frac{2}{x_*})=0$. We next compute the Jacobi matrix $\frac{\partial \mathcal {F}}{\partial [\mathcal {R}_1,\mathcal {W}_1]}$

4.208 $$\begin{aligned} \begin{aligned} \frac{\partial \mathcal {F}}{\partial [\mathcal {R}_1,\mathcal {W}_1]}&= \left( \begin{array}{cc} \frac{\partial \mathcal {F}_1}{\partial \mathcal {R}_1} &{} \frac{\partial \mathcal {F}_1}{\partial \mathcal {W}_1} \\ \frac{\partial \mathcal {F}_2}{\partial \mathcal {R}_1} &{} \frac{\partial \mathcal {F}_2}{\partial \mathcal {W}_1} \end{array} \right) \end{aligned} \end{aligned}$$

where

$$\begin{aligned} \frac{\partial \mathcal {F}_1}{\partial \mathcal {R}_1}&= -2\eta \mathcal {R}_0^{-\eta -1} \mathcal {R}_1 - x_*^2 H_1 - x_*^2 \mathcal {R}_1 \tfrac{\partial H_1}{\partial \mathcal {R}_1} - 2x_*^2 H_0 + 2x_*^2 (1-\varepsilon ) \mathcal {R}_0 (\mathcal {W}_0 +\varepsilon ) , \\ \frac{\partial \mathcal {F}_1}{\partial \mathcal {W}_1}&= - x_*^2 \mathcal {R}_1 \tfrac{\partial H_1}{\partial \mathcal {W}_1} - 2x_*^2 (1-\varepsilon ) \mathcal {R}_0 (\mathcal {W}_0 +\varepsilon ) , \\ \frac{\partial \mathcal {F}_2}{\partial \mathcal {R}_1}&=- \eta \mathcal {R}_0^{-\eta -1} \mathcal {W}_1 - x_*^2 \mathcal {W}_1 \tfrac{\partial H_1}{\partial \mathcal {R}_1} + \eta \mathcal {R}_0^{-\eta -1} - 3\eta \mathcal {W}_0\mathcal {R}_0^{-\eta -1} \\&\quad + x_*^2\tfrac{\partial H_1}{\partial \mathcal {R}_1} - 3x_*^2 \mathcal {W}_0 \tfrac{\partial H_1}{\partial \mathcal {R}_1} - 2x_*^2 (1+\varepsilon ) \mathcal {W}_0 (\mathcal {W}_0 +\varepsilon ), \\ \frac{\partial \mathcal {F}_2}{\partial \mathcal {W}_1}&= - \eta \mathcal {R}_0^{-\eta -1} \mathcal {R}_1 - x_*^2 (H_1+2H_0)- x_*^2 \mathcal {W}_1 \tfrac{\partial H_1}{\partial \mathcal {W}_1} + 3 \mathcal {R}_0^{-\eta } \\&\quad + x_*^2\tfrac{\partial H_1}{\partial \mathcal {W}_1}- 3x_*^2 H_0 - 3x_*^2 \mathcal {W}_0 \tfrac{\partial H_1}{\partial \mathcal {W}_1} + 2x_*^2 (1+\varepsilon ) \mathcal {W}_0 (\mathcal {W}_0 +\varepsilon ), \end{aligned}$$

and

$$\begin{aligned} \tfrac{\partial H_1}{\partial \mathcal {R}_1} = 4\varepsilon \mathcal {W}_0, \quad \tfrac{\partial H_1}{\partial \mathcal {W}_1} = 2[(1+\varepsilon )\mathcal {W}_0 + 2\varepsilon ]. \end{aligned}$$

Using $H_1(0)= 2\mathcal {W}_0(0)\mathcal {W}_1(0)$, $\tfrac{\partial H_1}{\partial \mathcal {R}_1} (0)=0$, $\tfrac{\partial H_1}{\partial \mathcal {W}_1}(0)= 2\mathcal {W}_0(0)$, $H_0(0)=\mathcal {W}_0^2(0)$, $\mathcal {W}_0(0)=\mathcal {R}_0(0) =\frac{1}{x_*}$, $\mathcal {W}_1(0)=\frac{x_*-2}{x_*}$ and $\mathcal {R}_1(0)=-\frac{1}{x_*}$, we evaluate the above at $(\varepsilon ,\mathcal {R}_1,\mathcal {W}_1)= (0,-\frac{1}{x_*}, 1-\frac{2}{x_*})$

$$\begin{aligned} \begin{aligned} \frac{\partial \mathcal {F}_1}{\partial \mathcal {R}_1}\Big |_{(k,\mathcal {R}_1,\mathcal {W}_1)= (0,-\frac{1}{x_*}, 1-\frac{2}{x_*})}&= - x_*^2H_1(0) - 2x_*^2 H_0 (0) + 2x_*^2 \mathcal {R}_0(0) \mathcal {W}_{0}(0) = - 2(x_*-2) , \\ \frac{\partial \mathcal {F}_1}{\partial \mathcal {W}_1}\Big |_{(k,\mathcal {R}_1,\mathcal {W}_1)= (0,-\frac{1}{x_*}, 1-\frac{2}{x_*})}&=- 2x_*^2 \mathcal {R}_1(0) \mathcal {W}_0(0) - 2x_*^2 \mathcal {R}_0(0) \mathcal {W}_0(0)= 0, \\ \frac{\partial \mathcal {F}_2}{\partial \mathcal {R}_1}\Big |_{(k,\mathcal {R}_1,\mathcal {W}_1)= (0,-\frac{1}{x_*}, 1-\frac{2}{x_*})}&=- 2x_*^2 \mathcal {W}_0^2(0)= -2, \\ \frac{\partial \mathcal {F}_2}{\partial \mathcal {W}_1}\Big |_{(k,\mathcal {R}_1,\mathcal {W}_1)= (0,-\frac{1}{x_*}, 1-\frac{2}{x_*})}&=- x_*^2 (H_1(0) +2H_0(0)) - 2x_*^2 \mathcal {W}_1(0)\mathcal {W}_0(0) + 3 \\&\quad + 2x_*^2 \mathcal {W}_0(0) - 3x_*^2 H_0(0) - 6x_*^2 \mathcal {W}_0^2(0)+ 2x_*^2 \mathcal {W}_0^2(0) \\&= - 4x_*^2 \mathcal {W}_0(0)\mathcal {W}_1(0) + 2 x_*- 6 = - 4 (x_*-2) + 2 x_*- 6 \\&= -2 (x_*-1). \end{aligned} \end{aligned}$$

The Jacobian at $(\varepsilon ,\mathcal {R}_1,\mathcal {W}_1)= (0,-\frac{1}{x_*}, 1-\frac{2}{x_*})$ is

4.209 $$\begin{aligned} \begin{aligned} \left| \frac{\partial \mathcal {F}}{\partial [\mathcal {R}_1,\mathcal {W}_1]} \right| _{(\varepsilon ,\mathcal {R}_1,\mathcal {W}_1)= (0,-\frac{1}{x_*}, 1-\frac{2}{x_*})}&= 4 (x_*-2) (x_*-1)>0 \quad \text {if } \ x_*>2 \end{aligned} \end{aligned}$$

and thus, $\frac{\partial \mathcal {F}}{\partial [\mathcal {R}_1,\mathcal {W}_1]}$ is invertible in sufficiently small neighborhood of $(0,-\frac{1}{x_*}, 1-\frac{2}{x_*})$ for any fixed $x_*>2$. Now by the implicit function theorem, we deduce that there exists an open interval $(-l_0,l_0)$ of $\varepsilon =0$ and unique continuously differential functions $g_1:(-l_0,l_0) \rightarrow (r_1,r_2)$, $g_2:(-l_0,l_0) \rightarrow (w_1,w_2)$ such that $g_1(0)=-\frac{1}{x_*}$, $g_2(0)= 1-\frac{2}{x_*}$ and $\mathcal {F}(\varepsilon , g_1(\varepsilon ), g_2(\varepsilon )) =0$ for all $\varepsilon \in (-l_0,l_0)$. $\square$

It is evident that $\mathcal {R}_1$ and $\mathcal {W}_1$ become degenerate at $x_*=2$. In particular, when $\varepsilon =0$, the corresponding $\mathcal {R}_1$ and $\mathcal {W}_1$ for the LP-type and the Hunter-type solutions coincide precisely at $x_*=2$, while they are well-defined for further values of $x_*$ below 2, see [15]. Interestingly this feature does not persist for $\varepsilon >0$. In fact, we will show that $\mathcal {R}_1$ and $\mathcal {W}_1$ cease to exist as real numbers before $x_*$ reaches 2 from above. To this end, we first derive the algebraic equation satisfied by $\mathcal {W}_1$.

#### Lemma 4.7

(The cubic equation for $\mathcal {W}_1$) The Taylor coefficient $\mathcal {W}_1$ is a root of the cubic polynomial

4.210 $$\begin{aligned} P(X) {:}{=} X^3 + \frac{a_2}{a_3} X^2+ \frac{a_1}{a_3} X + \frac{a_0}{a_3}, \end{aligned}$$

where $a_j$, $j=0,1,2,3$ are real-valued continuous functions of $\mathcal {W}_0$ given in (4.221)–(4.224) below. Moreover, there exists a constant $c>0$ and $0<\varepsilon _0\ll 1$ such that for all $0<\varepsilon \ll \varepsilon _0$ and any $\frac{1}{3}<\mathcal {W}_0<1$, we have $c<a_3<\frac{1}{c}$, $c<\frac{a_0}{\varepsilon (3\mathcal {W}_0-1)}<\frac{1}{c}$. In particular any root of $X\mapsto P(X)$ is not zero for $0<\varepsilon \ll \varepsilon _0$.

#### Proof

Note that (4.201) can be rearranged as

4.211 $$\begin{aligned} \begin{aligned} -x_*^2H_1 [\mathcal {W}_1+ 3\mathcal {W}_0 -1] - x_*^2 H_0 [\eta \tfrac{\mathcal {R}_1}{\mathcal {W}_0} +2] [\mathcal {W}_1+ 3\mathcal {W}_0 -1] \\ - 2x_*^2 (1+\varepsilon ) \mathcal {W}_0(\mathcal {W}_0 + \varepsilon )(\mathcal {R}_1 -\mathcal {W}_1) =0 . \end{aligned} \end{aligned}$$

Rewrite (4.200) as

4.212 $$\begin{aligned} \begin{aligned}&\left[ {(1-\varepsilon ) \mathcal {W}_0(\mathcal {W}_0+\varepsilon ) -H_0 - [ \tfrac{\eta }{2} \tfrac{H_0}{\mathcal {W}_0} + 2\varepsilon \mathcal {W}_0]\mathcal {R}_1}\right] \mathcal {R}_1\\&\quad -\left[ {(1-\varepsilon )\mathcal {W}_0(\mathcal {W}_0+\varepsilon ) +[(1+\varepsilon )\mathcal {W}_0+2\varepsilon ]\mathcal {R}_1} \right] \mathcal {W}_1= 0, \end{aligned} \end{aligned}$$

and (4.211) as

4.213 $$\begin{aligned} \begin{aligned} \mathcal {R}_1= - \frac{ [(1+\varepsilon )\mathcal {W}_0+2\varepsilon ]\mathcal {W}_1^2 + [(3+5\varepsilon )\mathcal {W}_0^2- (1-8\varepsilon +\varepsilon ^2)\mathcal {W}_0- (3\varepsilon -\varepsilon ^2)] \mathcal {W}_1 + (3\mathcal {W}_0-1) H_0}{(1+\varepsilon )\mathcal {W}_0(\mathcal {W}_0+\varepsilon ) +[\frac{\eta }{2}\frac{H_0}{\mathcal {W}_0} + 2\varepsilon \mathcal {W}_0] (3\mathcal {W}_0-1) +[\frac{\eta }{2}\frac{H_0}{\mathcal {W}_0} + 2\varepsilon \mathcal {W}_0]\mathcal {W}_1}. \end{aligned} \end{aligned}$$

We would like to derive an algebraic equation for $\mathcal {W}_1$. To this end, write (4.213) and (4.212) as

4.214 $$\begin{aligned} \mathcal {R}_1 = - \frac{d\mathcal {W}_1^2 + e\mathcal {W}_1 + cH_0}{h+ b\mathcal {W}_1}, \end{aligned}$$

where

4.215 $$\begin{aligned}&d{:}{=}(1+\varepsilon )\mathcal {W}_0+2\varepsilon , \ \ e{:}{=}(3+5\varepsilon )\mathcal {W}_0^2- (1-8\varepsilon +\varepsilon ^2)\mathcal {W}_0- (3\varepsilon -\varepsilon ^2), \ \ c{:}{=}3\mathcal {W}_0-1, \end{aligned}$$

4.216 $$\begin{aligned}&h{:}{=} (1+\varepsilon )\mathcal {W}_0(\mathcal {W}_0+\varepsilon ) +[\frac{\eta }{2}\frac{H_0}{\mathcal {W}_0} + 2\varepsilon \mathcal {W}_0] (3\mathcal {W}_0-1) , \ \ b{:}{=} \frac{\eta }{2}\frac{H_0}{\mathcal {W}_0} + 2\varepsilon \mathcal {W}_0. \end{aligned}$$

We observe that we may write $h=(1+\varepsilon )a+ bc$, where

4.217 $$\begin{aligned} a{:}{=}\mathcal {W}_0(\mathcal {W}_0+\varepsilon ). \end{aligned}$$

If we let

4.218 $$\begin{aligned} f{:}{=}(1-\varepsilon )a -H_0, \end{aligned}$$

then (4.212) can be rewritten in the form

4.219 $$\begin{aligned}{}[f- b\mathcal {R}_1]\mathcal {R}_1 - [(1-\varepsilon )a + d \mathcal {R}_1]\mathcal {W}_1=0, \end{aligned}$$

with *f*, .*b*, *a*, *d* as above. Now replace $\mathcal {R}_1$ in (4.219) using the relation  (4.214):

$$\begin{aligned}&-\left[ f+ b \frac{d\mathcal {W}_1^2 + e\mathcal {W}_1 + cH_0}{h+ b\mathcal {W}_1} \right] \frac{d\mathcal {W}_1^2 + e\mathcal {W}_1 + cH_0}{h+ b\mathcal {W}_1}\\&\quad - \left[ (1-\varepsilon )a - d \frac{d\mathcal {W}_1^2 + e\mathcal {W}_1 + cH_0}{h+ b\mathcal {W}_1} \right] \mathcal {W}_1=0. \end{aligned}$$

Multiply $$(h+ b\mathcal {W}_1)^2$$:

$$\begin{aligned}&- \left[ f(h+ b\mathcal {W}_1)+ b (d\mathcal {W}_1^2 + e\mathcal {W}_1 + cH_0) \right] (d\mathcal {W}_1^2 + e\mathcal {W}_1 + cH_0) \\&- \left[ (1-\varepsilon )a (h+ b\mathcal {W}_1)- d(d\mathcal {W}_1^2 + e\mathcal {W}_1 + cH_0) \right] (h+ b\mathcal {W}_1) \mathcal {W}_1=0. \end{aligned}$$

Note that the highest order term $\mathcal {W}_1^4$ is not present. Rearrange similar terms to conclude the identity

4.220 $$\begin{aligned} a_3 \mathcal {W}_1^3 + a_2 \mathcal {W}_1^2 + a_1\mathcal {W}_1 + a_0 = 0, \end{aligned}$$

where

4.221 $$\begin{aligned} a_3&{:}{=} d ( d h - be )- b (fd +(1-\varepsilon ) ab), \end{aligned}$$

4.222 $$\begin{aligned} a_2&{:}{=}-\Big [bcdH_0 -e ( d h - be ) +h (fd+(1-\varepsilon )ab) + b ( fe + (1-\varepsilon ) a h) \Big ],\end{aligned}$$

4.223 $$\begin{aligned} a_1&{:}{=}-\Big [ bceH_0 -cH_0 ( d h - be ) + bcf H_0 + h ( fe + (1-\varepsilon ) a h ) \Big ],\end{aligned}$$

4.224 $$\begin{aligned} a_0&{:}{=}- cH_0 ( bcH_0 + fh). \end{aligned}$$

By (4.224), the sign of $a_0$ is the same as the sign of $bcH_0 + fh$. We now use

$$\begin{aligned}&bcH_0 + fh = \left[ \frac{\eta }{2}\frac{H_0}{\mathcal {W}_0} + 2\varepsilon \mathcal {W}_0 \right] \left[ 3\mathcal {W}_0-1 \right] H_0\\&\quad + \left[ (1-\varepsilon ) \mathcal {W}_0(\mathcal {W}_0+\varepsilon ) -H_0 \right] \left[ (1+\varepsilon )\mathcal {W}_0(\mathcal {W}_0+\varepsilon ) +[\tfrac{\eta }{2}\tfrac{H_0}{\mathcal {W}_0} + 2\varepsilon \mathcal {W}_0] (3\mathcal {W}_0-1) \right] \\&= \left[ (1-\varepsilon ) \mathcal {W}_0(\mathcal {W}_0+\varepsilon ) -H_0 \right] (1+\varepsilon )\mathcal {W}_0(\mathcal {W}_0+\varepsilon ) \\&\quad + (1-\varepsilon ) \mathcal {W}_0(\mathcal {W}_0+\varepsilon ) [\tfrac{\eta }{2}\tfrac{H_0}{\mathcal {W}_0} + 2\varepsilon \mathcal {W}_0] (3\mathcal {W}_0-1)\\&= \mathcal {W}_0(\mathcal {W}_0+\varepsilon )\underbrace{ \Big \{(1+\varepsilon )\underbrace{\left[ (1-\varepsilon ) \mathcal {W}_0(\mathcal {W}_0+\varepsilon ) -H_0 \right] }_{(I)} + \varepsilon \underbrace{[\tfrac{H_0}{\mathcal {W}_0} + 2(1-\varepsilon )\mathcal {W}_0]}_{(II)} (3\mathcal {W}_0-1) \Big \}}_{(III)}, \end{aligned}$$

where we have used $\frac{\eta }{2}=\frac{\varepsilon }{1-\varepsilon }$ in the last line. Now

$$\begin{aligned} (I)&= (1-\varepsilon ) \mathcal {W}_0(\mathcal {W}_0+\varepsilon ) - (1+3\varepsilon )\mathcal {W}_0^2 - 4\varepsilon \mathcal {W}_0 +\varepsilon -\varepsilon ^2\\&=\varepsilon \left[ -4 \mathcal {W}_0^2 - (3+\varepsilon ) \mathcal {W}_0 +1-\varepsilon \right] \end{aligned}$$

and

$$\begin{aligned} (II)&= (1+3\varepsilon )\mathcal {W}_0 + 4\varepsilon - \tfrac{\varepsilon -\varepsilon ^2}{\mathcal {W}_0} + 2(1-\varepsilon ) \mathcal {W}_0\\&= (3+\varepsilon )\mathcal {W}_0 + 4\varepsilon - \tfrac{\varepsilon -\varepsilon ^2}{\mathcal {W}_0}. \end{aligned}$$

Hence

$$\begin{aligned} (III)&=\varepsilon \Big \{ -4(1+\varepsilon )\mathcal {W}_0^2 - (3+\varepsilon )(1+\varepsilon )\mathcal {W}_0 + 1-\varepsilon ^2 + [ (3+\varepsilon )\mathcal {W}_0+ 4\varepsilon - \tfrac{\varepsilon -\varepsilon ^2}{\mathcal {W}_0} ](3\mathcal {W}_0-1) \Big \}\\&= \varepsilon \Big \{ (5-\varepsilon )\mathcal {W}_0^2 - (6-7\varepsilon +\varepsilon ^2) \mathcal {W}_0 + (1-7\varepsilon +2\varepsilon ^2) + \tfrac{\varepsilon -\varepsilon ^2}{\mathcal {W}_0}\Big \}\\&= \varepsilon \Big \{ 5\mathcal {W}_0^2 - 6\mathcal {W}_0 + 1+ O(\varepsilon ) \Big \}. \end{aligned}$$

Since $$5\mathcal {W}_0^2 - 6\mathcal {W}_0 + 1= (5\mathcal {W}_0-1) (\mathcal {W}_0-1) <0$$ for $$\frac{1}{5}<\mathcal {W}_0<1$$,

4.225 $$\begin{aligned} a_0&= - cH_0 ( bcH_0 + fh) \nonumber \\&= - \varepsilon cH_0 \mathcal {W}_0(\mathcal {W}_0+\varepsilon )\Big \{ 5\mathcal {W}_0^2 - 6\mathcal {W}_0 + 1+ O(\varepsilon ) \Big \} \nonumber \\&>0 \ \text { for } \ \frac{1}{3}< \mathcal {W}_0<1 \text { and } 0<\varepsilon \ll 1. \end{aligned}$$

From (4.215)–(4.217) it is easy to see that

4.226 $$\begin{aligned} a&= \mathcal {W}_0^2 + O(\varepsilon ), \ \ b= O(\varepsilon ), \ \ c= 3\mathcal {W}_0-1, \ \ d= \mathcal {W}_0 +O(\varepsilon ), \end{aligned}$$

4.227 $$\begin{aligned} e&= 3\mathcal {W}_0^2-\mathcal {W}_0 +O(\varepsilon ), \ \ f= (1-\varepsilon ) \mathcal {W}_0(\mathcal {W}_0+\varepsilon ) -H_0= O(\varepsilon ) , \end{aligned}$$

4.228 $$\begin{aligned} h&= (1+\varepsilon )\mathcal {W}_0(\mathcal {W}_0+\varepsilon ) +[\tfrac{\varepsilon }{1-\varepsilon }\tfrac{H_0}{\mathcal {W}_0} + 2\varepsilon \mathcal {W}_0] (3\mathcal {W}_0-1)= \mathcal {W}_0^2 +O(\varepsilon ). \end{aligned}$$

This then implies

4.229 $$\begin{aligned} a_3=&\mathcal {W}_0^4 +O(\varepsilon ), \ \ a_2=\mathcal {W}_0^4(3\mathcal {W}_0-1) +O(\varepsilon ), \nonumber \\ a_1=&\mathcal {W}_0^5(2\mathcal {W}_0-1)+O(\varepsilon ), \ \ \frac{a_0}{\varepsilon (3\mathcal {W}_0-1)} \sim 1 \end{aligned}$$

which shows the uniform positivity and boundedness of $a_3$ for sufficiently small $\varepsilon$. Upon dividing (4.220) by $a_3$ we finally conclude the proof of the lemma. $\square$

#### Remark 4.8

In the formal Newtonian limit $\varepsilon =0$, the cubic equation $P(X)=0$ reduces to

4.230 $$\begin{aligned} X^3 + (3\mathcal {W}_0-1) X^2 + (2\mathcal {W}_0-1)\mathcal {W}_0 X = X (X +\mathcal {W}_0)(X+ 2\mathcal {W}_0 -1 ) =0. \end{aligned}$$

The root $X=0$ corresponds to the Hunter-type, $X=1-2\mathcal {W}_0$ is of the LP-type, and $X=-\mathcal {W}_0$ is the Newtonian ghost solution, see [15].

### Relativistic Larson-Penston-Type Solutions

By Lemma 4.7 we know that there are in general three complex roots of (4.210) giving possible values for $\mathcal {W}_1$. One of those roots is a “ghost root" and is discarded as unphysical solution of (4.210). More precisely, in the Newtonian limit $\varepsilon \rightarrow 0$ such a spurious root corresponds to the value $\mathcal {W}_1(0)=-\frac{1}{x_*}$. Our goal is to first mod out such a solution by using the implicit function theorem to construct a curve $\varepsilon \rightarrow \mathcal {W}^{\text {gh}}_1(\varepsilon )$ of spurious solutions agreeing with $-\frac{1}{x_*}$ when $\varepsilon =0$.

#### Lemma 4.9

(Ghost root) The cubic polynomial *P* introduced in Lemma 4.7 can be factorised in the form

4.231 $$\begin{aligned} P(X) = (X-\mathcal {W}_1^{\text {gh}}(\varepsilon )) Q(X), \end{aligned}$$

where $\mathcal {W}_1^{\text {gh}}(\varepsilon )$ is a real valued function of $\mathcal {W}_0(\varepsilon )$ and *Q* is a quadratic polynomial given by

4.232 $$\begin{aligned} Q(X) = X^2 + \left[ \frac{a_2}{a_3} +\mathcal {W}_1^{\text {gh}} \right] X - \frac{a_0}{a_3 \mathcal {W}_1^{\text {gh}}} . \end{aligned}$$

#### Proof

We differentiate (4.210) to obtain

4.233 $$\begin{aligned} \begin{aligned} \frac{\partial P}{\partial X}\Big |_{(\varepsilon ,X)=(0,-\frac{1}{x_*})}&= \left( 3X^2 + 2\frac{a_2}{a_3} X+ \frac{a_1}{a_3}\right) \Big |_{(\varepsilon ,X)=(0,-\frac{1}{x_*})} \\&= 3\frac{1}{x_*^2} - 2\frac{3-x_*}{x_*}\frac{1}{x_*} + \frac{2-x_*}{x_*^2}= \frac{x_*-1}{x_*^2} >0. \end{aligned} \end{aligned}$$

The implicit function theorem implies the existence of a unique $\varepsilon$-parametrised curve

4.234 $$\begin{aligned} \mathcal {W}_1^{\text {gh}}(\varepsilon )= -\frac{1}{x_*} + O(\varepsilon ) \end{aligned}$$

satisfying $P(\mathcal {W}_1^{\text {gh}}(\varepsilon ))=0$. Identity (4.231) can now be checked directly, keeping in mind (4.232).

The discriminant of *Q* is given by

$$\begin{aligned} \Delta _Q= \left( \frac{a_2}{a_3} +\mathcal {W}_1^{\text {gh}} \right) ^2 + \frac{4a_0}{a_3 \mathcal {W}_1^{\text {gh}}}. \end{aligned}$$

$$\square $$

#### Lemma 4.10

(Definition of $x_{\text {crit}}(\varepsilon )$) There exists a continuous curve $(0,\varepsilon _0]\ni \varepsilon \mapsto x_{\text {crit}}(\varepsilon )\in (\frac{1}{2},\frac{7}{2})$ such that

4.235 $$\begin{aligned} \Delta _Q(x_{\text {crit}}(\varepsilon ))&=0, \end{aligned}$$

4.236 $$\begin{aligned} \Delta _Q(x_*)&>0, \ \ x_*\in (x_{\text {crit}}(\varepsilon ),x_{\text {max}}(\varepsilon )], \end{aligned}$$

4.237 $$\begin{aligned} x_{\text {crit}}(\varepsilon )&= 2+ O(\sqrt{\varepsilon })>2. \end{aligned}$$

Moreover, for any $x_*\in (x_{\text {crit}}(\varepsilon ),\frac{7}{2}]$ there exists a constant $C_*=C_*(x_*)>0$ such that for all $\varepsilon \in (0,\varepsilon _0]$

4.238 $$\begin{aligned} \left| \mathcal {W}_1^{\text {{RLP}}} (\varepsilon ; x_*) - \frac{x_*-2}{x_*} \right| \le C_*\varepsilon . \end{aligned}$$

When $x_*=x_{\text {crit}}(\varepsilon )$ the rate of convergence changes and there exists a constant $\bar{C}>0$ such that

4.239 $$\begin{aligned} \left| \mathcal {W}_1^{\text {{RLP}}} (\varepsilon ; x_{\text {crit}}(\varepsilon )) - \frac{x_{\text {crit}}(\varepsilon )-2}{x_{\text {crit}}(\varepsilon )} \right| \le \bar{C} \sqrt{\varepsilon }. \end{aligned}$$

#### Proof

*Step 1. Existence of* $x_{\text {crit}}$. From the asymptotic formulas (4.229) and (4.234) it is clear that for all $\varepsilon \in (0,\varepsilon _0]$ we have

4.240 $$\begin{aligned} - \left[ \frac{a_2}{a_3} +\mathcal {W}_1^{\text {gh}} \right] = \frac{x_*-2}{x_*}+O(\varepsilon ), \end{aligned}$$

and therefore

$$\begin{aligned} \Delta _Q(\varepsilon ,x_*) = \left( \frac{x_*-2}{x_*}+O(\varepsilon )\right) ^2 + \frac{4a_0}{a_3 \mathcal {W}_1^{\text {gh}}} . \end{aligned}$$

However, by Lemma 4.7 we know that $\frac{4a_0}{a_3 \mathcal {W}_1^{\text {gh}}} \le - \alpha (3\mathcal {W}_0-1) \varepsilon$ for some positive constant $\alpha >0$. Choosing $x_*<2$ such that $2-x_*=O(\varepsilon )$, we see that $\Delta _Q(\varepsilon ,x_*)<0$. On the other hand, for any $x_*\ge 2+\delta$ for some small, but fixed $\delta >0$, we see that $\Delta _Q(\varepsilon ,x_*)>0$. Therefore, by the intermediate value property, there exists an $x_*\in (2-O(\varepsilon ),2+\delta )$ such that $\Delta _Q(x_*)=0$. Let $x_{\text {crit}}$ be the largest such $x_*$ - it is clear from the construction that the properties (4.235)–(4.236) are satisfied.

*Step 2. Asymptotic behaviour of* $x_{\text {crit}}(\varepsilon )$. From (4.240), the identity

4.241 $$\begin{aligned} - \left[ \frac{a_2}{a_3} +\mathcal {W}_1^{\text {gh}} \right] \Big \vert _{x_*=x_{\text {crit}}(\varepsilon )} =\sqrt{ - \frac{4a_0}{a_3 \mathcal {W}_1^{\text {gh}}} }\Big \vert _{x_*=x_{\text {crit}}(\varepsilon )}, \end{aligned}$$

and Lemma 4.7 we easily conclude (4.237).

*Step 3. Continuity properties of the map* $\varepsilon \mapsto \mathcal {W}_1^{\text {{RLP}}}(\varepsilon ,x_*)$. Fix an $x_*\in (x_{\text {crit}},x_{\text {max}}]$. Then

4.242 $$\begin{aligned} \mathcal {W}_1^{\text {RLP}} (\varepsilon ; x_*) - \frac{x_*-2}{x_*}&= \frac{- \left[ \frac{a_2}{a_3} +\mathcal {W}_1^{\text {gh}} \right] + \sqrt{( \frac{a_2}{a_3} +\mathcal {W}_1^{\text {gh}} )^2 + \frac{4a_0}{a_3 \mathcal {W}_1^{\text {gh}}}} }{2} - \frac{x_*-2}{x_*} \nonumber \\&=\frac{ - \left[ \frac{a_2}{a_3} +\mathcal {W}_1^{\text {gh}} \right] - \frac{x_*-2}{x_*}}{2} + \frac{\sqrt{( \frac{a_2}{a_3} +\mathcal {W}_1^{\text {gh}} )^2 + \frac{4a_0}{a_3 \mathcal {W}_1^{\text {gh}}}} - \frac{x_*-2}{x_*} }{2} \nonumber \\&=: (i) + (ii). \end{aligned}$$

By (4.240) we have $(i)=O(\varepsilon )$. For (*ii*),

4.243 $$\begin{aligned} (ii)= \frac{( \frac{a_2}{a_3} +\mathcal {W}_1^{\text {gh}} )^2 - (\frac{x_*-2}{x_*})^2 + \frac{4a_0}{a_3 \mathcal {W}_1^{\text {gh}} }}{ 2 \left[ \sqrt{( \frac{a_2}{a_3} +\mathcal {W}_1^{\text {gh}} )^2 + \frac{4a_0}{a_3 \mathcal {W}_1^{\text {gh}}}} + \frac{x_*-2}{x_*}\right] } = \frac{O(\varepsilon )}{ \sqrt{(x_*-2)^2 + O(\varepsilon ) } +(x_*-2) } \ \ \text { as }\quad \varepsilon \rightarrow 0^+, \end{aligned}$$

by (4.229) and (4.240). Therefore, the bound (4.238) follows.

If we now let $x_*=x_{\text {crit}}(\varepsilon )$, since by definition $\Delta _Q(x_{\text {crit}}(\varepsilon ))=0$, we have

4.244 $$\begin{aligned} \mathcal {W}_1^{\text {RLP}} (\varepsilon ; x_{\text {crit}}(\varepsilon )) - \frac{x_{\text {crit}}(\varepsilon )-2}{x_{\text {crit}}(\varepsilon )}&= -\frac{ \frac{a_2}{a_3} +\mathcal {W}_1^{\text {gh}}}{2} - \frac{x_{\text {crit}}(\varepsilon )-2}{x_{\text {crit}}(\varepsilon )} \nonumber \\&= - \sqrt{-\frac{a_0}{a_3\mathcal {W}_1^{\text {gh}}}}- \frac{x_{\text {crit}}(\varepsilon )-2}{x_{\text {crit}}(\varepsilon )} \nonumber \\&= O_{\varepsilon \rightarrow 0^+}(\sqrt{\varepsilon }), \end{aligned}$$

where we have used (4.237) and Lemma 4.7. This proves (4.239). $\square$

Of special importance in our analysis is the Friedmann solution, which has the property that for any $\varepsilon >0$ $\mathcal {W}_0=\mathcal {R}_0=\frac{1}{3}$. By Lemma 4.1 there exists a continuously differentiable curve $\varepsilon \rightarrow x_{\text {max}}(\varepsilon )$ defined through the property

4.245 $$\begin{aligned} \mathcal {W}_0(\varepsilon ;x_{\text {max}}(\varepsilon )) = \frac{1}{3}, \ \ \varepsilon \in [0,\varepsilon _0]. \end{aligned}$$

Since $\mathcal {W}_0(0;3)=\frac{1}{3}$, we conclude from Lemma 4.1 that

4.246 $$\begin{aligned} x_{\text {max}}(\varepsilon )<3 \ \ \text { and } \ 3-x_{\text {max}}(\varepsilon )=O(\varepsilon ), \ \ \varepsilon \in [0,\varepsilon _0]. \end{aligned}$$

With above preparations in place, we are now ready to define what we mean by a solution of the *relativistic Larson-Penston type*.

#### Definition 4.11

(Solutions of RLP-type) Let $0<\varepsilon _0\ll 1$ be given by Lemma 4.7 and let $x_*\in [x_{\text {crit}},x_{\text {max}}]$. We say that the sequence $(\mathcal {R}_N,\mathcal {W}_N)_{N\in \mathbb N}$ is of relativistic Larson-Penston (RLP)-type if for all $\varepsilon \in (0,\varepsilon _0]$

4.247 $$\begin{aligned} \mathcal {R}_0&= \mathcal {W}_0, \end{aligned}$$

the coefficients $(\mathcal {R}_N,\mathcal {W}_N)_{N\in \mathbb N}$ satisfy the recursive relations (4.195)–(4.196), $\mathcal {W}_1$ is the root of the quadratic polynomial *Q* (4.232) given by

4.248 $$\begin{aligned} \mathcal {W}_1 =\mathcal {W}_1^{\text {{RLP}}} = \frac{- \left[ \frac{a_2}{a_3} +\mathcal {W}_1^{\text {gh}} \right] + \sqrt{( \frac{a_2}{a_3} +\mathcal {W}_1^{\text {gh}} )^2 + \frac{4a_0}{a_3 \mathcal {W}_1^{\text {gh}}}} }{2}, \end{aligned}$$

and $\mathcal {R}_1$ is given as a function of $\mathcal {W}_0$ and $\mathcal {W}_1$ via (4.213).

#### Remark 4.12

For all $\varepsilon \in (0,\varepsilon _0]$ it is clear from the proof of the lemma that the following bound holds in the interval $x_*\in [x_{\text {crit}}(\varepsilon ),x_{\text {max}}(\varepsilon )]$:

4.249 $$\begin{aligned} \mathcal {W}_1^{\text {RLP}} (\varepsilon ; x_*) - \frac{x_*-2}{x_*} = \frac{O(\varepsilon )}{ \sqrt{(x_*-2)^2 + O(\varepsilon ) } +(x_*-2) }. \end{aligned}$$

### High-Order Taylor Coefficients

We now consider (4.195)$_{N\ge 2}$ and (4.196)$_{N\ge 2}$.

#### Lemma 4.13

Let $N\ge 2$. Then the following holds

4.250 $$\begin{aligned} \mathcal {A}_N (\mathcal {W}_0, \mathcal {W}_1, \mathcal {R}_1) \left( \begin{array}{c} \mathcal {R}_N \\ \mathcal {W}_N \end{array} \right) = \left( \begin{array}{c} \mathcal {S}_N \\ \mathcal {V}_N \end{array} \right) . \end{aligned}$$

Here

4.251 $$\begin{aligned} \mathcal {A}_N (\mathcal {W}_0, \mathcal {W}_1, \mathcal {R}_1) = \left( \begin{array}{cc} A_{11} \ &{} A_{12} \\ A_{21} \ &{} A_{22} \end{array} \right) \end{aligned}$$

where

$$\begin{aligned} A_{11}&=-2x_*^2 H_0 N + 2x_*^2 (1-\varepsilon ) \mathcal {R}_0 (\mathcal {W}_0+\varepsilon ) -x_*^2 H_1 N - \eta \mathcal {R}_0^{-\eta -1}\mathcal {R}_1(N+1) - 4\varepsilon x_*^2 \mathcal {W}_0\mathcal {R}_1, \\ A_{12}&= -x_*^2 \mathcal {R}_1 (2(1-\varepsilon ) \mathcal {W}_0 + 4\varepsilon + 4\varepsilon \mathcal {R}_0) - 2x_*^2 (1-\varepsilon ) \mathcal {R}_0 (\mathcal {W}_0+\varepsilon ), \\ A_{21}&= - 2x_*^2 (1+\varepsilon ) \mathcal {W}_0 (\mathcal {W}_0+\varepsilon ) - (\eta \mathcal {R}_0^{-\eta -1} +4\varepsilon \mathcal {W}_0x_*^2 ) (\mathcal {W}_1 +3\mathcal {W}_0 -1), \\ A_{22}&= - 2x_*^2 H_0 N - 3x_*^2 H_0 + 3\mathcal {R}_0^{-\eta } + 2x_*^2 (1+\varepsilon ) \mathcal {W}_0 (\mathcal {W}_0+\varepsilon )\\&\quad -x_*^2 ( \mathcal {W}_1 + 3\mathcal {W}_0-1) (2(1-\varepsilon ) \mathcal {W}_0 + 4\varepsilon + 4\varepsilon \mathcal {R}_0) -x_*^2 H_1N - \eta \mathcal {R}_0^{-\eta -1} \mathcal {R}_1N , \end{aligned}$$

and

$$\begin{aligned} \mathcal {S}_N= \mathcal {S}_N[\mathcal {R}_0,\mathcal {W}_0; R_1,\mathcal {W}_1;\cdots , \mathcal {R}_{N-1}, \mathcal {W}_{N-1}], \\ \mathcal {V}_N= \mathcal {V}_N[\mathcal {R}_0,\mathcal {W}_0; R_1,\mathcal {W}_1;\cdots , \mathcal {R}_{N-1}, \mathcal {W}_{N-1}], \end{aligned}$$

are given in (4.255) and (4.258).

#### Proof

We first observe that there are no terms involving $(\mathcal {R}_{N+1}, \mathcal {W}_{N+1})$ in (4.195) and (4.196) due to the sonic conditions (4.199) (cf. Remark 4.4 and see the cancelation below). To prove the lemma, we isolate all the coefficients in $(\mathcal {R}^{-\eta })_N$ and $H_N$ that contain the top order coefficients $\mathcal {R}_{N}, \mathcal {W}_{N}$. For $(\mathcal {R}^{-\eta })_N$, from (4.190) we have

4.252 $$\begin{aligned} \begin{aligned} (\mathcal {R}^{-\eta })_N&= -\eta \mathcal {R}_0^{-\eta -1}\mathcal {R}_N \\&\quad + \mathcal {R}_0^{-\eta } \sum _{m=2}^N \frac{1}{\mathcal {R}_0^m}\sum _{\pi (N,m)} (- \eta )_m \frac{1}{\lambda _1 ! \dots \lambda _N !} {\mathcal {R}_1}^{\lambda _1}\dots {\mathcal {R}_N}^{\lambda _N} \end{aligned} \end{aligned}$$

where we recall $\lambda _j=0$ for $N-m+2\le j\le N$, in particular $\lambda _N=0$ and therefore there are no terms involving $\mathcal {R}_N$ in the summation term. For $H_N$, from (4.194), we have

4.253 $$\begin{aligned} \begin{aligned} H_N&= 2(1-\varepsilon )\mathcal {W}_0 \mathcal {W}_N + 4\varepsilon \mathcal {W}_N + 4\varepsilon (\mathcal {R}_0 \mathcal {W}_N+ \mathcal {R}_N \mathcal {W}_0)\\&\quad + (1-\varepsilon ) \sum _{\begin{array}{c} \ell +m =N\\ 1\le m\le N-1 \end{array}} \mathcal {W}_\ell \mathcal {W}_m+ \sum _{\begin{array}{c} \ell +m =N\\ 1\le m\le N-1 \end{array}} 4\varepsilon \mathcal {R}_\ell \mathcal {W}_m . \end{aligned} \end{aligned}$$

Using (4.252) and (4.253), we can isolate all the coefficients in (4.195) that contain contributions from $(R_{N}, W_{N})$ as follows:4.254where

4.255 $$\begin{aligned} \begin{aligned} \mathcal {S}_N = - \mathcal {R}_1\mathcal {R}_0^{-\eta } \sum _{m=2}^N \frac{1}{\mathcal {R}_0^m}\sum _{\pi (N,m)} (- \eta )_m \frac{1}{\lambda _1 ! \dots \lambda _N !} {\mathcal {R}_1}^{\lambda _1}\dots {\mathcal {R}_N}^{\lambda _N} \\ +x_*^2 \mathcal {R}_1 \Big [ (1-\varepsilon ) \sum _{\begin{array}{c} \ell +m =N\\ 1\le m\le N-1 \end{array}} \mathcal {W}_\ell \mathcal {W}_m+ \sum _{\begin{array}{c} \ell +m =N\\ 1\le m\le N-1 \end{array}} 4\varepsilon \mathcal {R}_\ell \mathcal {W}_m\Big ] +\tilde{\mathcal {S}}_N, \end{aligned} \end{aligned}$$

and

4.256 $$\begin{aligned} \begin{aligned}&\tilde{\mathcal {S}}_N = - \sum _{\begin{array}{c} \ell +m=N \\ 1\le m\le N-2 \end{array}} (m+1) \mathcal {R}_{m+1} (\mathcal {R}^{-\eta })_\ell \\&+x_*^2 \left( \sum _{\begin{array}{c} \ell +m=N\\ 1\le m\le N-2 \end{array}} (m+1) \mathcal {R}_{m+1} H_\ell + 2\sum _{\begin{array}{c} \ell +m=N-1 \\ m\le N-2 \end{array}} (m+1) \mathcal {R}_{m+1}H_\ell +\sum _{\ell +m=N-2} (m+1) \mathcal {R}_{m+1}H_\ell \right) \\&- 2x_*^2(1-\varepsilon )\left( \sum _{\begin{array}{c} \ell +m+n=N\\ 1\le n\le N-1 \end{array}} \mathcal {R}_\ell ( \mathcal {W}+ \varepsilon )_m ( \mathcal {R}- \mathcal {W})_n +( \mathcal {R}( \mathcal {W}+ \varepsilon ) ( \mathcal {R}- \mathcal {W}) )_{N-1} \right) . \end{aligned} \end{aligned}$$

Here we recall the definitions of $(\mathcal {R}^{-\eta })_\ell$ in (4.190) and $H_1$ in (4.203) as well as the sonic conditions in (4.199). Note that we have also used (4.202) above.

Following the same procedure, we now isolate all the coefficients in (4.196) that contain contributions from $(\mathcal {R}_{N}, \mathcal {W}_{N})$.4.257where

4.258 $$\begin{aligned} \begin{aligned} \mathcal {V}_N = - (\mathcal {W}_1-1 +3\mathcal {W}_0)\mathcal {R}_0^{-\eta } \sum _{m=2}^N \frac{1}{\mathcal {R}_0^m}\sum _{\pi (N,m)} (- \eta )_m \frac{1}{\lambda _1 ! \dots \lambda _N !} {\mathcal {R}_1}^{\lambda _1}\dots {\mathcal {R}_N}^{\lambda _N} \\ +x_*^2 ( \mathcal {W}_1 -1 + 3\mathcal {W}_0) \Big [ (1-\varepsilon ) \sum _{\begin{array}{c} \ell +m =N\\ 1\le m\le N-1 \end{array}} \mathcal {W}_\ell \mathcal {W}_m+ \sum _{\begin{array}{c} \ell +m =N\\ 1\le m\le N-1 \end{array}} 4\varepsilon \mathcal {R}_\ell \mathcal {W}_m\Big ] +\tilde{\mathcal {V}}_N \end{aligned} \end{aligned}$$

and

4.259 $$\begin{aligned} \tilde{\mathcal {V}}_N&= - \sum _{\begin{array}{c} \ell +m=N \\ 1\le m\le N-2 \end{array}} (m+1) \mathcal {W}_{m+1} (\mathcal {R}^{-\eta })_\ell \nonumber \\&\quad +x_*^2 \left( \sum _{\begin{array}{c} \ell +m=N\\ 1\le m\le N-2 \end{array}} (m+1) \mathcal {W}_{m+1} H_\ell + 2\sum _{\begin{array}{c} \ell +m=N-1 \\ m\le N-2 \end{array}} (m+1) \mathcal {W}_{m+1}H_\ell +\sum _{\ell +m=N-2} (m+1) \mathcal {W}_{m+1}H_\ell \right) \nonumber \\&\quad + \sum _{\begin{array}{c} \ell +m=N \\ 1\le m\le N \end{array}} (\mathcal {R}^{-\eta })_\ell (-1)^m - 3\sum _{\begin{array}{c} \ell +m+n=N \\ 1\le m\le N \end{array}} \mathcal {W}_n (\mathcal {R}^{-\eta })_\ell (-1)^m \nonumber \\&\quad - x_*^2 \left( \sum _{\begin{array}{c} \ell +m=N \\ 1\le m\le N \end{array}} H_\ell (-1)^m +2 \sum _{\ell +m=N-1} H_\ell (-1)^m + \sum _{\ell +m=N-2}H_\ell (-1)^m \right) \nonumber \\&\quad + 3x_*^2 \left( \sum _{\begin{array}{c} \ell +n=N\\ 1\le n\le N-1 \end{array}} \mathcal {W}_n H_\ell \!+\! \sum _{\begin{array}{c} \ell +m+n=N\\ 1\le m\le N \end{array}} \mathcal {W}_n H_\ell (-1)^m \!+\!2 \sum _{\ell +m+n=N-1} W_nH_\ell (-1)^m \!+\! \sum _{\ell +m+n=N-2}W_n H_\ell (-1)^m \right) \nonumber \\&\quad + 2x_*^2(1+\varepsilon )\left( \sum _{\begin{array}{c} \ell +m+n=N\\ 1\le n\le N-1 \end{array}} \mathcal {W}_\ell ( \mathcal {W}+ \varepsilon )_m ( \mathcal {R}- \mathcal {W})_n +( \mathcal {W}( \mathcal {W}+ \varepsilon ) ( \mathcal {R}- \mathcal {W}) )_{N-1} \right) . \end{aligned}$$

This completes the proof. $\square$

Let $\kappa >0$ be a sufficiently small number independent of $\varepsilon$ to be fixed later in Section 4.5.

#### Lemma 4.14

Let $x_*\in [x_{\text {crit}}+\kappa ,x_{\text {max}}]$ be given. Then the components $A_{ij}$ of the matrix $\mathcal {A}_N$ satisfy the following

4.260 $$\begin{aligned} A_{11}&= -2 N\left( x_*-1 + O(\varepsilon )\right) + 2 +O(\varepsilon ), \end{aligned}$$

4.261 $$\begin{aligned} A_{12}&=O(\varepsilon ), \end{aligned}$$

4.262 $$\begin{aligned} A_{21}&= -2+O(\varepsilon ), \end{aligned}$$

4.263 $$\begin{aligned} A_{22}&= -2N\left( x_*-1+O(\varepsilon ) \right) +O(\varepsilon ). \end{aligned}$$

In particular, the matrix $\mathcal {A}_N$ is invertible for all sufficiently small $\varepsilon$ and $\det \mathcal {A}_N = O(N^2)$. Moreover, the Taylor coefficients $(\mathcal {R}_N,\mathcal {W}_N)$, $N\ge 2$, satisfy the recursive relationship

4.264 $$\begin{aligned} \mathcal {R}_N = \frac{A_{22}}{\det A_N} \mathcal {S}_N - \frac{A_{12}}{\det A_N } \mathcal {V}_N, \end{aligned}$$

4.265 $$\begin{aligned} \mathcal {W}_N = \frac{ A_{11}}{\det A_N} \mathcal {V}_N- \frac{A_{21}}{\det A_N} \mathcal {S}_N, \end{aligned}$$

where the source terms $\mathcal {V}_N,\mathcal {S}_N$, $N\ge 2$, are given by Lemma 4.13.

#### Proof

We rearrange terms in $A_{ij}$ of Lemma 4.13 as

$$\begin{aligned} A_{11}&=-2N \left( x_*^2 H_0 + \tfrac{1}{2} x_*^2 H_1 + \frac{\eta }{2}\mathcal {R}_0^{-\eta -1}\mathcal {R}_1\right) \\&\quad + 2x_*^2 \mathcal {R}_0\mathcal {W}_0 + 2\varepsilon x_*^2 \mathcal {R}_0 (1-\varepsilon -\mathcal {W}_0) - \eta \mathcal {R}_0^{-\eta -1}\mathcal {R}_1 - 4\varepsilon x_*^2 \mathcal {W}_0\mathcal {R}_1,\\ A_{12}&= - 2x_*^2 \mathcal {R}_1 \mathcal {W}_0 - 2x_*^2 \mathcal {R}_0 \mathcal {W}_0 - 2\varepsilon x_*^2 \mathcal {R}_1 ( 2 + 2\mathcal {R}_0- \mathcal {W}_0) - 2\varepsilon x_*^2 \mathcal {R}_0 (1-\varepsilon -\mathcal {W}_0),\\ A_{21}&= - 2x_*^2\mathcal {W}_0^2 - 2\varepsilon x_*^2 \mathcal {W}_0 (\mathcal {W}_0+1+\varepsilon ) - 2\varepsilon (\tfrac{1}{1-\varepsilon }\mathcal {R}_0^{-\eta -1} +2 \mathcal {W}_0x_*^2 ) (\mathcal {W}_1 +3\mathcal {W}_0 -1),\\ A_{22}&=-2N \left( x_*^2 H_0 + \tfrac{1}{2} x_*^2 H_1 + \frac{\eta }{2}\mathcal {R}_0^{-\eta -1}\mathcal {R}_1 \right) \\&\quad - 3x_*^2 H_0 + 3\mathcal {R}_0^{-\eta } + 2x_*^2 \mathcal {W}_0^2 -2x_*^2\mathcal {W}_0 ( \mathcal {W}_1 + 3\mathcal {W}_0-1) \\&\quad + 2\varepsilon x_*^2 \mathcal {W}_0 (\mathcal {W}_0+1+\varepsilon ) -2\varepsilon x_*^2 ( \mathcal {W}_1 + 3\mathcal {W}_0-1) (2 +2\mathcal {R}_0- \mathcal {W}_0) . \end{aligned}$$

From (4.199), equations (4.202)–(4.203) and Lemmas 4.1, 4.6, we have the relations

$$\begin{aligned} H_0 = \frac{1}{x_*^2} + O(\varepsilon ), \ \ H_1= 2\mathcal {W}_0(0)\mathcal {W}_1(0) + O(\varepsilon ) = \frac{2}{x_*}\left( 1-\frac{2}{x_*}\right) + O(\varepsilon ), \end{aligned}$$

and therefore

$$\begin{aligned} A_{11}&= -2 N\left( 1+ \tfrac{\mathcal {W}_1(0)}{\mathcal {W}_0(0)} + O(\varepsilon )\right) + 2 +O(\varepsilon ), \\ A_{12}&= -2\tfrac{\mathcal {R}_1(0)}{\mathcal {W}_0(0)} - 2+O(\varepsilon ) ,\\ A_{21}&= -2+O(\varepsilon ), \\ A_{22}&= -2N\left( 1+ \tfrac{\mathcal {W}_1(0)}{\mathcal {W}_0(0)}+O(\varepsilon ) \right) -4 +2\tfrac{1}{\mathcal {W}_0(0)}-2\tfrac{\mathcal {W}_1(0)}{\mathcal {W}_0(0)} +O(\varepsilon ). \end{aligned}$$

Since $\mathcal {R}_0(0)=\mathcal {W}_0(0)=\frac{1}{x_*}$, $\frac{\mathcal {W}_1(0)}{\mathcal {W}_0(0)} = x_*-2$ and $\mathcal {R}_1(0)=-\frac{1}{x_*}$, the claimed behavior of $A_{ij}$ follows.

Since $N\ge 2$ and $x_*\ge x_{\text {crit}}+\kappa >2$, the determinant of $\mathcal {A}_N$ has a lower bound

$$\begin{aligned} \tfrac{1}{4}\det \mathcal {A}_N&= \left( N(x_*-1+O(\varepsilon )) +O(\varepsilon ) \right) \left( N (x_*-1+O(\varepsilon )) -1 +O(\varepsilon )\right) +O(\varepsilon ) \\&\ge \left( N(x_*-1-C\varepsilon _0))-C\varepsilon _0 \right) \left( N (x_*-1-C\varepsilon _0) -1-C\varepsilon _0\right) - C\varepsilon _0 \end{aligned}$$

for some universal constant $C>0$. For a sufficiently small $\varepsilon _0>0$ we have $N(x_*-1-C\varepsilon _0))-C\varepsilon _0 \ge \frac{3}{2}$ for $N\ge 2$ and $x_*\ge x_{\text {crit}}+\kappa >2$. We see that $\det \mathcal {A}_N >0$ and hence $\mathcal {A}_N$ is invertible for all $0<\varepsilon \le \varepsilon _0$ with $\varepsilon _0$ chosen sufficiently small. It immediately follows that $\det \mathcal {A}_N = O(N^2)$. Since $\mathcal {A}_N$ is invertible, relations (4.264)–(4.265) follow by multiplying (4.250) by $\mathcal {A}_N^{-1}$ from the left. $\square$

#### Remark 4.15

A simple consequence of the previous lemma is the existence of a universal constant $\beta _0>0$ such that for any $x_*\in [x_{\text {crit}}+\kappa ,x_{\text {max}}]$ and sufficiently small $0<\varepsilon \le \varepsilon _0$ the following bounds hold:

4.266 $$\begin{aligned} | \mathcal {R}_N|\le \frac{\beta _0}{N} \left( |\mathcal {S}_N| + \frac{\varepsilon }{N} |\mathcal {V}_N| \right) , \end{aligned}$$

4.267 $$\begin{aligned} |\mathcal {W}_N| \le \frac{\beta _0}{N} \left( |\mathcal {V}_N| + \frac{1}{N} |\mathcal {S}_N| \right) . \end{aligned}$$

#### Remark 4.16

It is a routine to check

4.268 $$\begin{aligned} \mathcal {R}_2&= \frac{-x_*^2 + 6x_*-7}{ 2x_*(2x_*-3)} + O({\varepsilon }), \end{aligned}$$

4.269 $$\begin{aligned} \mathcal {W}_2&= \frac{-5x_*^2 + 19 x_*- 17}{ 2x_*(2x_*-3)} + O({\varepsilon }), \end{aligned}$$

for any $$x_*\in [x_{\text {cr}}+\kappa , x_{\max }]$$.

#### Remark 4.17

In Lemma 4.14, the lower bound $x_{\text {crit}}+\kappa$ for the $x_*$-interval has been chosen for convenience to ensure $O(\varepsilon )$ disturbance of the coefficients to the corresponding ones to LP type solutions. It can be relaxed to $x_{\text {crit}}$ by replacing $O(\varepsilon )$ by $O(\sqrt{\varepsilon })$. See the change of the distance of $\mathcal {W}_1$ near $x_{\text {crit}}$ in Lemma 4.10.

### Series Convergence and Local Existence Around a Sonic Point

#### Theorem 4.18

(Local existence around the sonic point) There exist an $\varepsilon _0>0$ and $r>0$ sufficiently small such that for all $0<\varepsilon \le \varepsilon _0$ and all $x_*\in [x_{\text {crit}}(\varepsilon )+\kappa ,x_{\text {max}}(\varepsilon )]$ the sequence $\{\mathcal {R}_N, \mathcal {W}_N\}_{N\in \mathbb Z_{\ge 0}}$ of RLP-type (see Definition 4.11) has the following property: the formal power series

4.270 $$\begin{aligned} \mathcal {R}(z){:}{=} \sum _{N=0}^\infty \mathcal {R}_N (\delta z)^N, \quad \mathcal {W}(z) {:}{=} \sum _{N=0}^\infty \mathcal {W}_N (\delta z)^N \end{aligned}$$

converge for all $z\in (1-r,1+r)$ and functions $z\mapsto \mathcal {R}(z)$ and $z\mapsto \mathcal {W}(z)$ are real analytic inside $|z-1|<r$. We can differentiate the infinite sums term by term, the pair $z\mapsto (\mathcal {R}(z), \mathcal {W}(z))$ solves (4.172)–(4.173) for $|z-1|<r$, and $\mathcal R(z)$ is strictly positive for $|z-1|<r$.

#### Proof

The proof is analogous to Theorem 2.10 of [15]. By Lemma A.9, using $\alpha \in (1,2)$, there exists $C>1$ such that

$$\begin{aligned} \sum _{N=1}^\infty |\mathcal {R}_N||\delta z|^N + \sum _{N=1}^\infty |\mathcal {W}_N||\delta z|^N \le 2 \sum _{N=1}^\infty \frac{|C\delta z|^N}{CN^3} <\infty \end{aligned}$$

when $|\delta z|<\frac{1}{C}{=}{:} r$. The claim follows by the comparison test. The real analyticity and differentiability statements are clear. Recalling (4.193), we may rewrite *B* as

4.271 $$\begin{aligned} \begin{aligned} B&=\sum _{N=0}^\infty (\mathcal {R}^{-\eta })_N (\delta z)^N - x_*^2 \sum _{N=0}^\infty H_N (\delta z)^N(1+ 2 \delta z + (\delta z)^2) \\&= \left[ (\mathcal {R}^{-\eta })_1 - x_*^2 (H_1 + 2 H_0) \right] \delta z + \sum _{N=2}^\infty \left[ (\mathcal {R}^{-\eta })_N - x_*^2 (H_N + 2H_{N-1} + H_{N-2}) \right] (\delta z)^N. \end{aligned} \end{aligned}$$

By (4.202)–(4.203)

4.272 $$\begin{aligned} \begin{aligned}&(\mathcal {R}^{-\eta })_1 - x_*^2 (H_1 + 2 H_0) \\&= -\eta \mathcal {R}_0^{-\eta -1} \mathcal {R}_1 - x_*^2 \Big ( 2\left[ (1+\varepsilon )\mathcal {W}_0 + 2\varepsilon \right] \mathcal {W}_1 + 4\varepsilon \mathcal {W}_0 \mathcal {R}_1 \\&\qquad \qquad \qquad \qquad \qquad \quad +2\left[ (1-\varepsilon )\mathcal {W}_0^2 + 4\varepsilon \mathcal {W}_0+ 4\varepsilon \mathcal {R}_0\mathcal {W}_0+\varepsilon ^2-\varepsilon \right] \Big ) \\&= - 2x_*^2 \mathcal {W}_0 (\mathcal {W}_1+ \mathcal {W}_0) -2\varepsilon \mathcal {P} \ne 0, \end{aligned} \end{aligned}$$

for all sufficiently small $\varepsilon$, where

$$\begin{aligned} \mathcal {P} = \tfrac{1}{1-\varepsilon } \mathcal {R}_0^{-\eta -1} \mathcal {R}_1 +x_*^2 \left( \mathcal {W}_0\mathcal {W}_1+2\mathcal {W}_1 + 2\mathcal {W}_0\mathcal {R}_1 +4 \mathcal {W}_0 + 3\mathcal {W}_0^2 +\varepsilon -1 \right) . \end{aligned}$$

Therefore, it is now easy to see that for all sufficiently small $\varepsilon$ and for $r>0$ sufficiently small, the function $B \ne 0$ for all $|z-1|<r$ and $z\ne 1$. As a consequence, $\mathcal {R}(z)$ and $\mathcal {W}(z)$ are indeed the solutions as can be seen by plugging the infinite series (4.270) into (4.197) and (4.198). $\square$

#### Lemma 4.19

There exist an $\varepsilon _0>0$ and $r>0$ sufficiently small such that for any $\alpha \in (1,2)$ and for all $x_*\in [x_{\text {crit}}(\varepsilon )+\kappa ,x_{\text {max}}(\varepsilon )]$ the sequence $\{\mathcal {R}_N, \mathcal {W}_N\}_{N\in \mathbb N_{\ge 0}}$ of RLP-type (see Definition 4.11) has the following property: There exists a constant $C=C(x_*,\alpha )>0$ such that for all $0<\varepsilon \le \varepsilon _0$ the bounds $|\partial _{x_*}\mathcal {R}_0|$, $|\partial _{x_*}\mathcal {W}_0|$, $|\partial _{x_*}\mathcal {R}_1|$, $|\partial _{x_*}\mathcal {W}_1|\le C$ hold and

4.273 $$\begin{aligned} |\partial _{x_*}\mathcal {R}_N|\le \frac{C^{N-\alpha }}{N^3}, \ \ N\ge 2, \end{aligned}$$

4.274 $$\begin{aligned} |\partial _{x_*}\mathcal {W}_N|\le \frac{C^{N-\alpha }}{N^3}, \ \ N\ge 2. \end{aligned}$$

In particular, the formal power series

$$\begin{aligned} \sum _{N=0}^\infty \partial _{x_*} \mathcal {R}_N (\delta z)^N, \quad \sum _{N=0}^\infty \partial _{x_*} \mathcal {W}_N (\delta z)^N \end{aligned}$$

converge for all *z* satisfying $|z-1|<r$. Moreover, the function $x_*\in (x_{\text {crit}}+\kappa ,x_{\text {max}})\rightarrow ( \mathcal {R}(z; x_*), \mathcal {W}(z;x_*) )$ is $C^1$ and the derivatives $\partial _{x_*} \mathcal {R}$ and $\partial _{x_*} \mathcal {W}$ are given by the infinite series above.

#### Proof

From Lemma 4.1 and Lemma 4.6, $\partial _{x_*} \mathcal {R}_0(0) =\partial _{x_*} \mathcal {W}_0(0)= -\frac{1}{x_*^2}$ and $\partial _{x_*}\mathcal {R}_1(0)= \frac{1}{x_*^2}$, $\partial _{x_*}\mathcal {W}_1(0)= \frac{2}{x_*^2}$, and it is clear that $|\partial _{x_*}\mathcal {R}_0|$, $|\partial _{x_*}\mathcal {W}_0|$, $|\partial _{x_*}\mathcal {R}_1|$, $|\partial _{x_*}\mathcal {W}_1|\le C$ for $x_*\in [x_{\text {crit}}+\kappa ,x_{\text {max}}]$ and for all sufficiently small $\varepsilon$. For $N\ge 2$, $\partial _{x_*} \mathcal {R}_N$ and $\partial _{x_*} \mathcal {W}_N$ are recursively given by differentiating the expression in (4.264) and (4.265):

4.275 $$\begin{aligned} \partial _{x_*} \mathcal {R}_N =\partial _{x_*} \left( \frac{A_{22}}{\det A_N}\right) \mathcal {S}_N+ \frac{A_{22}}{\det A_N} \partial _{x_*}\mathcal {S}_N - \partial _{x_*}\left( \frac{A_{12}}{\det A_N }\right) \mathcal {V}_N- \frac{A_{12}}{\det A_N } \partial _{x_*}\mathcal {V}_N, \end{aligned}$$

4.276 $$\begin{aligned} \partial _{x_*} \mathcal {W}_N =\partial _{x_*} \left( \frac{ A_{11}}{\det A_N} \right) \mathcal {V}_N + \frac{ A_{11}}{\det A_N} \partial _{x_*}\mathcal {V}_N -\partial _{x_*} \left( \frac{A_{21}}{\det A_N}\right) \mathcal {S}_N - \frac{A_{21}}{\det A_N} \partial _{x_*}\mathcal {S}_N . \end{aligned}$$

When $N=2$, the claim immediately follows. For $N\ge 3$, we will apply the same induction argument used for $\mathcal {R}_N, \mathcal {W}_N$ bounds. To this end, we first observe that from Lemma 4.13 and Lemma 4.14

4.277 $$\begin{aligned}&\partial _{x_*} A_{11} = - 2N (1+O(\varepsilon )) + O(\varepsilon ), \quad \partial _{x_*} A_{12} = O(\varepsilon ), \end{aligned}$$

4.278 $$\begin{aligned}&\partial _{x_*} A_{21} =O(\varepsilon ), \quad \partial _{x_*} A_{22} = - 2N (1+O(\varepsilon )) + O(\varepsilon ), \end{aligned}$$

4.279 $$\begin{aligned}&\partial _{x_*} \det \mathcal {A}_N = 4N(1+O(\varepsilon ))( 2N (( x_*-1) +O(\varepsilon )) -1) + O(\varepsilon ) , \end{aligned}$$

leading to

$$\begin{aligned} \left| \partial _{x_*} \left( \frac{A_{22}}{\det A_N}\right) \right| , \ \left| \partial _{x_*} \left( \frac{A_{11}}{\det A_N}\right) \right| \lesssim \frac{1}{N},\ \ \left| \partial _{x_*} \left( \frac{A_{12}}{\det A_N}\right) \right| \lesssim \frac{\varepsilon }{N^2}, \ \left| \partial _{x_*} \left( \frac{A_{21}}{\det A_N}\right) \right| \lesssim \frac{1}{N^2}. \end{aligned}$$

Hence using Lemmas A.8 and A.9,

4.280 $$\begin{aligned} \begin{aligned} \left| \partial _{x_*} \mathcal {R}_N \right|&\lesssim \frac{1}{N} |\mathcal {S}_N|+\frac{1}{N} |\partial _{x_*}\mathcal {S}_N| +\frac{\varepsilon }{N^2} |\mathcal {V}_N| +\frac{\varepsilon }{N^2} |\partial _{x_*}\mathcal {V}_N| \\&\lesssim \frac{ C^{N-\alpha }}{N^3} +\frac{1}{N} |\partial _{x_*}\mathcal {S}_N| +\frac{\varepsilon }{N^2} |\partial _{x_*}\mathcal {V}_N|, \\ \left| \partial _{x_*} \mathcal {W}_N \right|&\lesssim \frac{1}{N}| \mathcal {V}_N |+\frac{1}{N} |\partial _{x_*}\mathcal {V}_N | +\frac{1}{N^2} |\mathcal {S}_N| +\frac{1}{N^2} |\partial _{x_*}\mathcal {S}_N | \\&\lesssim \frac{ C^{N-\alpha }}{N^3} +\frac{1}{N} |\partial _{x_*}\mathcal {V}_N | + \frac{1}{N^2} |\partial _{x_*}\mathcal {S}_N |. \end{aligned} \end{aligned}$$

We now recall that $\mathcal {S}_N$ and $\mathcal {V}_N$ consist of sum and product of polynomials in $\mathcal {R}_0,\mathcal {W}_0,\dots ,\mathcal {R}_{N-1},\mathcal {W}_{N-1}$ and power functions of $\mathcal {R}_0$. When we differentiate with respect to $x_*$, at most one term indexed by $\mathcal {R}_i$ or $\mathcal {W}_i$, $0\le i\le N-1$ is differentiated. In particular, the same combinatorial structure in the problem is maintained and the same inductive proof relying on the already established bounds (1.828) and (1.829) gives (4.273) and (4.274). The remaining conclusions now follow easily. $\square$

### The Sonic Window and $x_{\text {min}}$

The goal of this subsection is to define $x_{\text {min}}$ and to define the sonic window, which serves as the basic interval in our shooting method in the next section. We begin with the following lemma.

#### Lemma 4.20

Consider the RLP type solution constructed in Theorem 4.18. There exist a small constant $\delta _0>0$ independent of $\varepsilon$ and $0<z_0=z_0(\delta _0) <1$ such that for $x_*= 2+\delta _0 > x_{\text {crit}}$

$$\begin{aligned} \mathcal {W}(z_0) >\frac{1}{2-2\eta } \end{aligned}$$

for all sufficiently small $\varepsilon >0$. Here we recall $\eta = \frac{2\varepsilon }{1-\varepsilon }$.

#### Proof

For any $x_*\in (x_{\text {crit}}, x_{\max })$, we Taylor-expand $\mathcal {W}$ in *z* around $z=1$:

4.281 $$\begin{aligned} \mathcal {W}(z) = \mathcal {W}(1) + \mathcal {W}'(1) (z-1) + \frac{\mathcal {W}''(\tilde{z})}{2} (z-1)^2 \end{aligned}$$

for some $\tilde{z} \in [z,1]$. We note that $\mathcal {W}(1)= \mathcal {W}_0$ and $\mathcal {W}'(1)= \mathcal {W}_1$. Let $\delta >0$ be a small constant independent of $\varepsilon$ to be fixed and set $x_*= 2+\delta$. Then we have

4.282 $$\begin{aligned} \mathcal {W}_0 = \frac{1}{2+\delta } + O(\varepsilon ), \quad \mathcal {W}_1= \frac{\delta }{2+\delta } + O(\varepsilon ), \end{aligned}$$

see the end of Remark 4.5. On the other hand, from (4.269) we have

4.283 $$\begin{aligned} \mathcal {W}''(1)= 2 \mathcal {W}_2 = \frac{1-\delta - 5\delta ^2}{(2+\delta ) (1+2\delta )} + O({\varepsilon }). \end{aligned}$$

Now let

$$\begin{aligned} z_0(\delta ,\varepsilon ) = \min \{ z_1 : \mathcal {W}''(z) \ge \frac{1}{4} \ \text {for all} \ z\in [z_1,1] \}. \end{aligned}$$

Observe that $z_0<1$ by (4.283) for $\varepsilon ,\delta$ sufficiently small. We now claim that there exists a small enough $\delta _0>0$ such that $z_0(\delta _0,\varepsilon )\le 1-\delta _0^{1/4}$ for all sufficiently small $\varepsilon$. Suppose not. Then for all $\delta >0$ and for some $\varepsilon _0>0$, $z_0(\delta ,\varepsilon _0) > 1-\delta ^{1/4}$. Thus there exists $1-\delta ^{1/4}<z_1=z_1(\delta ,\varepsilon _0)<1$ so that $\mathcal {W}''(z_1) <\frac{1}{4}$, but this is impossible because of (4.283) and the continuity of $\mathcal {W}''$. Now the Taylor expansion (4.281) at $z=1-\delta _0^{1/4}$ gives rise to

4.284 $$\begin{aligned} \begin{aligned} \mathcal {W}(1-\delta _0^{1/4})&= \frac{1}{2+\delta _0} - \frac{\delta _0}{2+\delta _0} \delta _0^{1/4} + \frac{\mathcal {W}''(\tilde{z})}{2} \delta _0^{1/2} + O(\varepsilon )\\&\ge \frac{1}{2+\delta _0} - \frac{\delta _0}{2+\delta _0} \delta _0^{1/4} + \frac{1}{8} \delta _0^{1/2} + O(\varepsilon ) \end{aligned} \end{aligned}$$

from which we deduce $\mathcal {W}(1-\delta _0^{1/4}) > \frac{1}{2-2\eta }$ for all sufficiently small $\varepsilon$. $\square$

We now define

4.285 $$\begin{aligned} x_{\text {min}}{:}{=} 2+\delta _0 \end{aligned}$$

where $\delta _0$ is given in Lemma 4.20. We observe that by construction $x_{\text {min}}$ is independent of $\varepsilon$. We are ready to introduce the sonic window:

#### Definition 4.21

(The sonic window) For any $0\le \varepsilon \le \varepsilon _0$ we refer to the interval $[x_{\text {min}},x_{\text {max}}(\varepsilon )]$ as the sonic window. We often drop the $\varepsilon$-dependence when the $\varepsilon$ is fixed.

#### Remark 4.22

Observe that by construction, the sonic window $[x_{\text {min}},x_{\text {max}}(\varepsilon )]$ is a strict subset of the interval [2, 3], while the interval $[x_{\text {crit}}(\varepsilon ), x_{\text {max}}(\varepsilon )]$ coincides with the interval [2, 3] when $\varepsilon =0$. The latter is precisely the range of possible sonic points within which we found the Newtonian Larson-Penston solution in [15] and our new sonic window $[x_{\text {min}},x_{\text {max}}(\varepsilon )]$ shows that the lower bound $x_*=2$ can be improved to $2+\delta _0$ even for the Newtonian problem.

For future use in Sections 5 and 6, we analyse the behaviour of $J-xW$ and $J-xD$ near the sonic point $x_*$.

#### Lemma 4.23

(Initialisation) Let $\varepsilon \in (0,\varepsilon _0]$, where $\varepsilon _0>0$ is a sufficiently small constant given by Theorem 4.18. There exist a $\delta >0$ and $c_0>0$ such that $c_0\varepsilon _0<\delta$ and for any $x_*\in [x_{\text {min}},x_{\text {max}}]$, the unique local RLP-type solution associated to $x_*$ given by Theorem 4.18, satisfies the bounds

*(a)*: 4.286 $$\begin{aligned} J[x;D]&<xW, \ \ x\in (x_*,x_*+\delta ), \end{aligned}$$
  4.287 $$\begin{aligned} J[x;D]&>xW, \ \ x\in (x_*-\delta ,x_*). \end{aligned}$$
*(b)*: 4.288 $$\begin{aligned} J[x;D]&>xD, \ \ x\in (x_*-2\delta , x_*-c_0\varepsilon _0), \end{aligned}$$
  4.289 $$\begin{aligned} J[x;D]&>xD, \ \ x\in (x_*+ c_0\varepsilon _0, x_*+2\delta ). \end{aligned}$$
*(c)*: Moreover, the following bound holds
  4.290 $$\begin{aligned} \left[ x(1-W(x))\right] \Big |_{x=x_*+\delta }\ge \frac{1}{2}, \ \ \text { for all} \ \varepsilon \in (0,\varepsilon _0]. \end{aligned}$$

#### Proof

*Proof of part (a).* Let $g(x){:}{=} xW - J$. Since *g* is a smooth function of $D$ and *W*, by Theorem 4.18, *g* is smooth near the sonic point. Note that $g(x_*)=0$ from Lemma 4.1. Since $g'= W + x W' - J'$, using Lemma 4.1, Lemma 4.6 and (3.127), we deduce that

$$\begin{aligned} g'(x_*) = 1- \frac{1}{x_*} + O(\varepsilon ) >\frac{1}{3} \ \text {for all} \ x_*\in [x_{\text {min}},x_{\text {max}}] \end{aligned}$$

for all sufficiently small $\varepsilon >0$. Therefore, *g* is locally strictly increasing and (4.286) and (4.287) hold for some $\delta >0$.

*Proof of part (b).* Since $J-xD=0$ at $x=x_*$, we use this and the formula (3.127) to conclude

4.291 $$\begin{aligned} (J' - (xD)')\Big |_{x=x_*} =&\varepsilon \frac{-2 (1+D_0)x_*D_0+(1-\varepsilon )x_*-\left( 2x_*^2 D_0 D_1+\frac{1}{1-\varepsilon }D_0^{-\eta -1}D_1\right) }{(1-\varepsilon )x_*D_0 + 2\varepsilon x_*(1+D_0)}\nonumber \\&- x_*D_1- D_0, \end{aligned}$$

where we recall (2.43). By Remark 4.5 and (4.177), we have $D_0=W_0=\frac{1}{x_*}+O(\varepsilon )$ and $D_1=-\frac{1}{x_*^2}+O(\varepsilon )$. Plugging this into the above expression, we conclude that

$$\begin{aligned} f'(x_*)=(J' - (xD)')\Big |_{x=x_*} = O(\varepsilon ). \end{aligned}$$

Similarly, $f'' = J'' - 2 D' - x D''$ and thus using $D''(x_*) = \frac{2}{x_*^2} \mathcal {R}_2= \frac{2}{x_*^2} \frac{-x_*^2 + 6x_*-7}{2x_*(2x_*-3)} +O(\varepsilon )$ (see (4.268)) and $J''(x_*) = O(\varepsilon )$, we have

$$\begin{aligned} f''(x_*) = \frac{2}{x_*^2} - x_*\frac{2}{x_*^2} \left( \frac{-x_*^2 + 6x_*-7}{2x_*(2x_*-3)}\right) + O(\varepsilon )= \frac{x_*^2 - 2x_*+1 }{x_*^2(2x_*-3 )}+ O(\varepsilon ) \end{aligned}$$

and hence $f''(x_*)>\frac{1}{9}$ for all $x_*\in [x_{\text {min}},x_{\text {max}}]$. Since $f''$ is uniformly continuous, there exists a $\delta >0$ such that

4.292 $$\begin{aligned} f''(x)> \frac{1}{18} \ \text {for} \ x\in (x_*-\delta ,x_*+\delta ) \end{aligned}$$

for all $$x_*\in [x_{\text {min}},x_{\text {max}}]$$.

We now Taylor-expand *f* at $x=x_*$ to obtain

$$\begin{aligned} f(x) = O(\varepsilon ) (x-x_*) + \frac{f''(\tilde{x})}{2} (x-x_*)^2, \quad x\in (x_*- \delta , x_*+\delta ) \end{aligned}$$

for some $\tilde{x}$ between $x_*$ and *x*. For $x>x_*$ and for some $c_1>0$ we have

$$\begin{aligned} f(x) > - c_1 \varepsilon (x-x_*) + \frac{1}{36} (x-x_*)^2 =\frac{1}{36} (x-x_*) \left( x-x_*- 36 c_1\varepsilon \right) . \end{aligned}$$

Therefore we deduce (4.289) with $c_0=36c_1$. An analogous argument gives (4.288).

*Proof of part (c).* Bound (4.290) follows trivially from Theorem 4.18 and the asymptotic behaviour as $\varepsilon \rightarrow 0$ in Lemma 4.1. $\square$

### Singularity at the Origin $x=0$

By analogy to the previous section, we Taylor-expand the solution at the origin $z = 0$ in order to prove a local existence theorem starting from the origin to the right. An immediate consistency condition follows from the presence of $\frac{1 -3W}{z}$ in (4.173): $\mathcal {W}(0) =\frac{1}{3}$ and $\mathcal {R}(0)= \tilde{\mathcal {R}}_0>0$ is a free parameter. Denote the solution from the left by $\mathcal {R}_-$ and $\mathcal {W}_-$ and assume that locally around $z=0$

4.293 $$\begin{aligned} \mathcal {R}_-(z;\tilde{\mathcal {R}}_0) {:}{=} \sum _{N=0}^\infty \tilde{\mathcal {R}}_N z^N, \quad \mathcal {W}_-(z;\tilde{\mathcal {R}}_0) {:}{=} \sum _{N=0}^\infty \tilde{\mathcal {W}}_N z^N \end{aligned}$$

where $\tilde{\mathcal {R}}_0>0$ is a free parameter and $\tilde{\mathcal {W}}_0 = \frac{1}{3}$. The following theorem asserts that the formal power series converge and hence $(\mathcal {R}_-,\mathcal {W}_-)$ are real analytic in a small neighbourhood of $z=0$.

#### Theorem 4.24

Let $\tilde{\mathcal {R}}_0>0$ be given. There exists an $0<\tilde{r}<1$ such that the formal power series (4.293) converge for all $z\in [0,\tilde{r})$. In particular, $\mathcal {R}_-$ and $\mathcal {W}_-$ are real analytic on $[0,\tilde{r})$. We can differentiate the infinite sums term by term and the functions $\mathcal {R}_-(z,\tilde{\mathcal {R}}_0)$ and $\mathcal {W}_-(z,\tilde{\mathcal {R}}_0)$ solve (4.172) and (4.173) with the initial conditions $\mathcal {R}_-(0;\tilde{\mathcal {R}}_0)= \tilde{\mathcal {R}}_0$, $\mathcal {W}_-(0;\tilde{\mathcal {R}}_0) = \frac{1}{3}$.

#### Proof

Around the origin $z=0$ we write out the formal expansion of *B*:

4.294 $$\begin{aligned} B= \mathcal {R}^{-\eta } -x_*^2Hz^2 = \sum _{j=0}^\infty (\mathcal {R}^{-\eta })_j z^j - x_*^2 \sum _{j=0}^\infty H_j z^{j+2} . \end{aligned}$$

By the Faa di Bruno formula (4.186)–(4.188)

4.295 $$\begin{aligned} (\mathcal {R}^{-\eta } )_j =\tilde{\mathcal {R}}_0^{-\eta } \sum _{m=1}^j \frac{1}{\tilde{\mathcal {R}}_0^m}\sum _{\pi (j,m)} (- \eta )_m \frac{1}{\lambda _1 ! \dots \lambda _j !} {\tilde{\mathcal {R}}_1}^{\lambda _1}\dots {\tilde{\mathcal {R}}_j}^{\lambda _j}, \quad j \ge 1 \end{aligned}$$

and $(\mathcal {R}^{-\eta } )_0 = \tilde{\mathcal {R}}_0^{-\eta }$. Plugging (4.293) into (4.172), we obtain the formal relation

4.296 $$\begin{aligned} \begin{aligned} 0&=\left( \sum _{\ell =0}^\infty (\mathcal {R}^{-\eta })_\ell z^\ell - x_*^2 \sum _{\ell =0}^\infty H_\ell z^{\ell +2} \right) \left( \sum _{m=0}^\infty (m+1) \tilde{\mathcal {R}}_{m+1} z^{m} \right) \\&\quad + 2x_*^2(1-\varepsilon ) \sum _{\ell =0}^\infty (\mathcal {R}( \mathcal {W}+\varepsilon ) ( \mathcal {R}- \mathcal {W}))_\ell z^{\ell +1} \\&=\sum _{N=0}^\infty \sum _{\ell +m=N}(m+1) \tilde{\mathcal {R}}_{m+1} (\mathcal {R}^{-\eta })_\ell z^N -x_*^2 \sum _{N=0}^\infty \sum _{\ell +m=N-2}(m+1) \tilde{\mathcal {R}}_{m+1} H_\ell z^N \\&\quad + 2x_*^2(1-\varepsilon ) \sum _{N=0}^\infty (\mathcal {R}( \mathcal {W}+ \varepsilon ) ( \mathcal {R}- \mathcal {W}))_{N-1} z^{N}. \end{aligned} \end{aligned}$$

Comparing the coefficients, we obtain

4.297 $$\begin{aligned} \begin{aligned} \sum _{\ell +m=N} (m+1) \tilde{\mathcal {R}}_{m+1} (\mathcal {R}^{-\eta })_\ell - x_*^2 \sum _{\ell +m=N-2} (m+1) \tilde{\mathcal {R}}_{m+1}H_\ell \\ \quad + 2x_*^2(1-\varepsilon ) ( \mathcal {R}( \mathcal {W}+\varepsilon ) ( \mathcal {R}- \mathcal {W}) )_{N-1} =0. \end{aligned} \end{aligned}$$

Similarly, plugging (4.293) into (4.173), we obtain

4.298 $$\begin{aligned} \begin{aligned} 0&=\left( \sum _{\ell =0}^\infty (R^{-\eta })_\ell z^\ell - x_*^2 \sum _{\ell =0}^\infty H_\ell z^{\ell +2} \right) \left( \sum _{m=0}^\infty (m+1) \tilde{\mathcal {W}}_{m+1} z^{m} + 3\sum _{m=0}^\infty \tilde{\mathcal {W}}_{m+1} z^m \right) \\&\quad - 2x_*^2(1+\varepsilon ) \sum _{\ell =0}^\infty (\mathcal {W}( \mathcal {W}+\varepsilon ) ( \mathcal {R}- \mathcal {W}))_\ell z^{\ell +1} \\&=\sum _{N=0}^\infty \sum _{\ell +m=N}(m+4) \tilde{\mathcal {W}}_{m+1} (\mathcal {R}^{-\eta })_\ell z^N -x_*^2 \sum _{N=0}^\infty \sum _{\ell +m=N-2}(m+4) \tilde{\mathcal {W}}_{m+1} H_\ell z^N \\&\quad -2x_*^2(1+\varepsilon ) \sum _{N=0}^\infty (\mathcal {W}( \mathcal {W}+ \varepsilon ) ( \mathcal {R}- \mathcal {W}))_{N-1} z^{N}. \end{aligned} \end{aligned}$$

Comparing the coefficients, we obtain

4.299 $$\begin{aligned} \begin{aligned} \sum _{\ell +m=N} (m+4) \tilde{\mathcal {W}}_{m+1} (\mathcal {R}^{-\eta })_\ell - x_*^2 \sum _{\ell +m=N-2} (m+4) \tilde{\mathcal {W}}_{m+1}H_\ell \\ \quad - 2x_*^2(1+\varepsilon ) ( \mathcal {R}( \mathcal {W}+ \varepsilon ) ( \mathcal {R}- \mathcal {W}) )_{N-1} =0. \end{aligned} \end{aligned}$$

Identities (4.297) and (4.299) give the recursive relationships

4.300 $$\begin{aligned} \tilde{\mathcal {R}}_{N+1} = \frac{1}{N+1} \tilde{\mathcal {S}}_{N+1}, \quad N\ge 0, \end{aligned}$$

4.301 $$\begin{aligned} \tilde{\mathcal {W}}_{N+1} = \frac{1}{N+4} \tilde{\mathcal {V}}_{N+1}, \quad N\ge 0, \end{aligned}$$

where

4.302 $$\begin{aligned} \tilde{\mathcal {S}}_{N+1}&=\tilde{\mathcal {R}}_0^{\eta } \Big [ - \sum _{\begin{array}{c} \ell +m=N\\ m\le N-1 \end{array}} (m+1) \tilde{\mathcal {R}}_{m+1} (\mathcal {R}^{-\eta })_\ell +x_*^2 \sum _{\ell +m=N-2} (m+1) \tilde{\mathcal {R}}_{m+1}H_\ell \end{aligned}$$

4.303 $$\begin{aligned}&\qquad \qquad - 2x_*^2(1-\varepsilon ) ( \mathcal {R}( \mathcal {W}+ \varepsilon ) ( \mathcal {R}- \mathcal {W}) )_{N-1}\Big ] ,\nonumber \\ \tilde{\mathcal {V}}_{N+1}&=\tilde{\mathcal {R}}_0^{\eta } \Big [ - \sum _{\begin{array}{c} \ell +m=N\\ m\le N-1 \end{array}} (m+4) \tilde{\mathcal {W}}_{m+1} (\mathcal {R}^{-\eta })_\ell +x_*^2 \sum _{\ell +m=N-2} (m+4) \tilde{\mathcal {W}}_{m+1}H_\ell \nonumber \\&\qquad \qquad + 2x_*^2(1+\varepsilon ) ( \mathcal {W}( \mathcal {W}+ \varepsilon ) ( \mathcal {R}- \mathcal {W}) )_{N-1}\Big ], \end{aligned}$$

where $\tilde{\mathcal {S}}_{N+1}$ and $\tilde{\mathcal {V}}_{N+1}$ depend only on $(\tilde{\mathcal {R}}_i, \tilde{\mathcal {W}}_i)$ for $0\le i\le N$. The rest of the proof is now entirely analogous to the proof of Theorem 4.18 and we omit the details. $\square$

#### Remark 4.25

We may repeat the same procedure as in Lemma 4.19 to deduce that $\partial _{\tilde{\mathcal {R}}_0} \mathcal {R}_-(z; \tilde{\mathcal {R}}_0)$ and $\partial _{\tilde{\mathcal {R}}_0} \mathcal {W}_-(z; \tilde{\mathcal {R}}_0)$ have the convergent power series near the origin and the function $\tilde{\mathcal {R}}_0\in (0,\infty ) \rightarrow (\mathcal {R}_-(z;\tilde{\mathcal {R}}_0), \mathcal {W}_-(z;\tilde{\mathcal {R}}_0))$ is $C^1$. And the derivatives $\partial _{\tilde{\mathcal {R}}_0} \mathcal {R}_-$ and $\partial _{\tilde{\mathcal {R}}_0}\mathcal {W}_-$ satisfy the system of ODEs obtained by the differentiating (4.172) and (4.173) with initial conditions $\partial _{\tilde{\mathcal {R}}_0} \mathcal {R}_-(0; \tilde{\mathcal {R}}_0)=1$ and $\partial _{\tilde{\mathcal {R}}_0}\mathcal {W}_-(0;\tilde{\mathcal {R}}_0 )=0$.

## The Friedmann Connection

### Sonic Time, A Priori Bounds, and the Key Continuity Properties of the Flow

We denote $x_*r$ by $\tilde{r}$, where *r* is the analyticity radius given by Theorem 4.18.

#### Definition 5.1

(Sonic time) For any $x_*\in [x_{\text {min}},x_{\text {max}}]$ consider the unique local solution on the interval $[x_*-\tilde{r},x_*+\tilde{r}]$ given by Theorem 4.18. The *sonic time* $s(x_*)$ is given by

5.304 $$\begin{aligned} s(x_*) : =&\inf _{x\in (0,x_*]} \Big \{ (D(\cdot ,x_*), W(\cdot ;x_*))\text { is a solution to~(2.35)--(2.36) on} \quad [x,x_*)\text { and } \nonumber \\&\ \ \ B(x';D,W)>0 \text { for all } \ x'\in [x,x_*)\Big \}, \end{aligned}$$

where we recall the definition (3.123) of *B*.

#### Lemma 5.2

Let $\tilde{r}>0$ be as above. Then

5.305 $$\begin{aligned} \frac{1}{3}<\frac{J[x;\frac{1}{3}]}{x} \ \ \text { for all } \ x\in [0,x_{\text {max}}-\tilde{r}]. \end{aligned}$$

#### Proof

By (3.124), bound (5.305) is equivalent to showing

5.306 $$\begin{aligned} \frac{1}{9}+\frac{2}{3}\eta (1+\frac{1}{3}) < \varepsilon + \frac{(\frac{1}{3})^{-\eta }}{(1-\varepsilon )x^2}. \end{aligned}$$

Since $x\le x_{\text {max}}-\tilde{r}=3+O(\varepsilon )-\tilde{r}$ the right-hand side above is larger than $\varepsilon +\frac{(\frac{1}{3})^{-\eta }}{(1-\varepsilon )(3+O(\varepsilon )-r)^2}$, which converges to $\frac{1}{(3-r)^2}$ as $\varepsilon \rightarrow 0^+$. The left-hand side on the other hand converges to $\frac{1}{9}$ and thus the claim follows. $\square$

#### Lemma 5.3

For any $x_*\in [x_{\text {min}},x_{\text {max}}]$ consider the unique local solution on the interval $[x_*-\tilde{r},x_*+\tilde{r}]$ given by Theorem 4.18. Then for any $x\in (s(x_*),x_*]$ the following bounds hold:

5.307 $$\begin{aligned} W(x)&< \frac{J(x)}{x}, \end{aligned}$$

5.308 $$\begin{aligned} |W(x)|&< \frac{J(x)}{x} + 2\eta (1+D), \end{aligned}$$

5.309 $$\begin{aligned} 0&<D(x), \end{aligned}$$

where $J$ is defined in (3.124). Moreover, for any $x\in (s(x_*),x_*-\delta ]$ such that $D(x)\ge \frac{1}{3}$ we have the upper bound

5.310 $$\begin{aligned} D(x)&< \frac{J(x)}{x}, \end{aligned}$$

where $0<\delta \ll 1$ is an $\varepsilon$-independent constant from Lemma 4.23.

#### Proof

Let $x_*\in [x_{\text {min}},x_{\text {max}}]$ and let $\mathring{x}\in (s(x_*),x_*)$. By Definition 5.1 there exists a $\kappa >0$ such that $B(x)>\kappa$ for all $x\in [\mathring{x},x_*-\tilde{r})$, which according to (3.123) is equivalent to the bound

$$\begin{aligned} \left( J-xW\right) \left( J+ 2\eta x(1+D) + xW\right) >\frac{\kappa }{1-\varepsilon }. \end{aligned}$$

If $W(x)>0$ then from the strict positivity of $J$ and the above bound we immediately have $W(x)<\frac{J(x)}{x}$. If on the other hand $W(x)\le 0$ then $J-xW>0$ and therefore from the above bound again $|xW| = - xW < J+ 2\eta x(1+D).$ The two bounds together imply

5.311 $$\begin{aligned} |W(x)| \le \frac{J(x)}{x} + 2\eta (1+D), \ \ x\in (s(x_*), x_*), \end{aligned}$$

which shows (5.308). The strict positivity of $D$ on $(s(x_*),x_*]$ follows by rewriting the equation (2.35) in the form

5.312 $$\begin{aligned} \frac{d}{dx}\left( \log D\right)&= - \frac{ 2x(1-\varepsilon ) (W + \varepsilon ) (D-W) }{B}. \end{aligned}$$

Finally, to prove (5.310), we observe that it suffices to show $f>0$ where we recall the formula $f=J-xD$. If $D(x)=\frac{1}{3}$, by Lemma 5.2, we are done. If $D(x)>\frac{1}{3}$, we consider two cases. First suppose $D>\frac{1}{3}$ on $[x,x_*-\delta ]$. Then $b<0$ on $[x,x_*-\delta ]$ by Lemma 3.9 and (5.307), and $f(x_*-\delta ) >0$ by Lemma 4.23. Hence, by using Corollary 3.8, we have

$$\begin{aligned} f(x)> f(x_*-\delta ) e^{\int _{x}^{x_*-\delta } a[z;D,W] \,dz} >0. \end{aligned}$$

If $$D\ngtr \frac{1}{3}$$ on $$[x,x_*-\delta ]$$, there should exist $$x_1\in (x,x_*-\delta ]$$ such that $$D>\frac{1}{3}$$ on $$[x,x_1)$$ and $$D(x_1)=\frac{1}{3}$$. Note that $$b<0$$ on $$[x,x_1)$$ and $$f(x_1)>0$$ by Lemma 5.2. By using Corollary 3.8 again, we obtain

5.313 $$\begin{aligned} f(x)> f(x_1) e^{\int _{x}^{x_1} a[z;D,W]\,dz} >0, \end{aligned}$$

which proves the claim. $\square$

#### Lemma 5.4

Let $\varepsilon \in (0,\varepsilon _0]$, where $\varepsilon _0>0$ is a small constant given by Theorem 4.18. For any $x_*\in [x_{\text {min}},x_{\text {max}}]$ consider the unique local RLP-type solution associated to $x_*$ given by Theorem 4.18. If $D(x)\ge \frac{1}{3}$ for some $x\in (s(x_*),x_*-\delta )$, then

5.314 $$\begin{aligned} D(x) < \frac{1}{x^{1-\varepsilon }}. \end{aligned}$$

#### Proof

Since $D(x)\ge \frac{1}{3}$, by Lemma 5.3, we have $\frac{D}{J[x;D]} <\frac{1}{x}$. Using the definition of $J[\cdot ;D]$ (3.124) it is easy to see that the inequality $\frac{D}{J[x;D]} <\frac{1}{x}$ is equivalent to

$$\begin{aligned} \sqrt{ \eta ^2 (1+D)^2 x^2 + \varepsilon x^2 + \tfrac{D^{-\eta }}{1-\varepsilon }} < \frac{ \varepsilon x^2 + \frac{D^{-\eta }}{1-\varepsilon } }{Dx} - \eta (1+D) x. \end{aligned}$$

This in turn implies

$$\begin{aligned} \varepsilon x^2 + \tfrac{D^{-\eta }}{1-\varepsilon }&< \frac{( \varepsilon x^2 + \frac{D^{-\eta }}{1-\varepsilon })^2 }{D^2 x^2} - 2\eta \tfrac{ (1+D)}{D} ( \varepsilon x^2 + \tfrac{D^{-\eta }}{1-\varepsilon })\\&\Longleftrightarrow 1< \frac{( \varepsilon x^2 + \frac{D^{-\eta }}{1-\varepsilon }) }{D^2 x^2} - 2\eta \tfrac{ (1+D)}{D} \\&\Longleftrightarrow \left[ (1+3\varepsilon )D^2 + 4\varepsilon D-\varepsilon (1-\varepsilon ) \right] D^{\eta } < \frac{1}{x^2}. \end{aligned}$$

Now we note that

$$\begin{aligned} (1+3\varepsilon )D^2 + 4\varepsilon D-\varepsilon (1-\varepsilon ) = D^2 + \varepsilon \left[ 3D^2 + 4D- (1-\varepsilon )\right] \ge D^2 \end{aligned}$$

where we have used $D\ge \frac{1}{3}$. This implies (5.314). $\square$

#### Remark 5.5

It is a priori possible that the solution blows-up at a point at which *B* remains strictly positive, for example through blow-up of *W*. It is trivial to see that this cannot happen in the Newtonian setting, but in the relativistic case it requires a careful argument, which is given in the next lemma.

#### Lemma 5.6

(No blow up before the sonic point) For any $x_*\in [x_{\text {min}},x_{\text {max}}]$ consider the unique RLP-type solution on the interval $(s(x_*),x_*]$. If $s(x_*)>0$, then

5.315 $$\begin{aligned} \liminf _{x\rightarrow s(x_*)^+}B(x)=0. \end{aligned}$$

#### Proof

Assume the opposite. In that case there exists a constant $\kappa >0$ such that $B(x)\ge \kappa$ for all $x\in (s(x_*),x_*-\tilde{r}]$. Our goal is to show that $|D(x)|+|W(x)|<\infty$ on $(s(x_*), x_*]$, which would lead to the contradiction.

*Step 1. Boundedness of* $D$. If $D(x)\ge \frac{1}{3}$ the bound (5.310) gives $D+\eta (1+D)<\sqrt{\eta ^2 (1+D)^2+\varepsilon +\frac{D^{-\eta }}{(1-\varepsilon )x^2}}$, which upon taking a square and using $2\eta D(1+D)>0$ leads to

$$\begin{aligned} D^2< \varepsilon + \frac{D^{-\eta }}{(1-\varepsilon )x^2}< \varepsilon + \frac{D^{-\eta }}{(1-\varepsilon )s(x_*)^2}. \end{aligned}$$

Since $0<\eta \ll 1$, this gives a uniform upper bound on $D$ on $(s(x_*),x_*-\tilde{r}]$

5.316 $$\begin{aligned} D(x)\le M, \quad x\in (s(x_*), x_*]. \end{aligned}$$

*Step 2. Boundedness of* |*W*|. It follows from (2.35)–(2.36) that there exists a sufficiently large value $N=N(s(x_*),x_*)>0$ such that $W'<0$, $D'>0$ if $W>N$ and $W'>0$, $D'>0$ if $W<-N$, where we use the already shown upper bound on $D$. In both cases, the two regions are dynamically trapped and we denote the union of the two regions by $I_N$. For any $x\in I_N$ we multiply (2.35) by $\frac{1+\varepsilon }{(1-\varepsilon )D}$, (2.36) by $\frac{1}{W}$, and sum them to obtain

5.317 $$\begin{aligned} \left( \log \left( D^{2(1+\eta )}W^2\right) \right) ' = \frac{2}{x}\left( \frac{1}{W}-3\right) <0 \; \text { for } \ |W|>N, \end{aligned}$$

with *N* sufficiently large. In particular, for any $s(x_*)<x_1<x_2<x_*$, where $x_1,x_2$ both belong to the invariant region $I_N$ above, we obtain

5.318 $$\begin{aligned} D(x_1)^{2(1+\eta )}W(x_1)^2> D(x_2)^{2(1+\eta )}W(x_2)^2 \end{aligned}$$

On the other hand, since $B\ge \kappa$, $|W|>N$, and $D<M$ there exists a universal constant constant $C=C(s(x_*),x_*)$ such that for a sufficiently large choice of *N*, rom (2.37) we have

5.319 $$\begin{aligned} D^{-\eta }>C W^2, \quad x\in I_N. \end{aligned}$$

We apply this to (5.318) to conclude that

$$\begin{aligned} \frac{1}{C} D(x_1)^{2+\eta } > D(x_2)^{2(1+\eta )}W(x_2)^2, \;\text { for any } \ x_1\in (s(x_*),x_2). \end{aligned}$$

This gives a lower bound for $D$ and therefore an upper bound for |*W*| via (5.319) in the region $I_N$. In particular $\limsup _{x\rightarrow s(x_*)^+}|W(x)|<\infty$ and the claim follows. $\square$

Essentially a consequence of the previous lemma and a standard ODE argument is the statement that as long as the sonic denominator *B* is bounded below by some constant $\delta >0$ for all $x\ge \bar{x}>s(x_*)$, we can extend the solution to the left to some interval $[\bar{x}-t,x_*]$, where $t>0$ depends only on $\delta$ and $\bar{x}$. The statement and the proof are analogous to Lemma 4.3 in [15] and we state it without proof.

#### Lemma 5.7

Let $x_*\in [x_{\text {min}},x_{\text {max}}]$ be given and consider the unique RLP-type solution $(D(\cdot ;x_*),W(\cdot ;x_*))$ to the left of $x=x_*$, given by Theorem 4.18. Assume that for some $\bar{x}\in (0,x_*-\tilde{r})$ and $\delta >0$ we have $\bar{x}>s(x_*)$ and the conditions

5.320 $$\begin{aligned} B(x)>\delta , \quad D(x)>0, \quad x\in [\bar{x},x_*-\tilde{r}], \end{aligned}$$

hold. Then there exists a $t=t(\delta ,\bar{x})>0$ such that the solution can be continued to the interval $[\bar{x}- t,x_*]$ so that

$$\begin{aligned} B(x)>0, \quad D(x)>0, \quad x\in [\bar{x}- t,x_*-\tilde{r}]. \end{aligned}$$

#### Proposition 5.8

Let $x_*\in [x_{\text {min}},x_{\text {max}}]$ be given and consider the unique RLP-type solution $(D(\cdot ;x_*),W(\cdot ;x_*))$ to the left of $x=x_*$.

*(a)*: (Upper semi-continuity of the sonic time). Then
  $$\begin{aligned} \limsup _{\tilde{x}_*\rightarrow x_*} s(\tilde{x}_*) \le s(x_*), \end{aligned}$$
  i.e. the map $x_*\rightarrow s(x_*)$ is upper semi-continuous. In particular, if $s(x_*)=0$ then the map $s(\cdot )$ is continuous at $x_*$.
*(b)*: ([Continuity of the flow away from the sonic time]) Let $\{x^n_*\}_{n\in \mathbb N}\subset [x_{\text {min}},x_{\text {max}}]$ and $x_*\subset [ x_{\text {min}},x_{\text {max}}]$ satisfy $\lim _{n\rightarrow \infty }x^n_*= x_*$. Let $x_*-\tilde{r}>z>\max \{s(x_*),\sup _{n\in \mathbb N}s(x_*^n)\}$. Then
  $$\begin{aligned} \lim _{n\rightarrow \infty }W(x;x_*^n) = W(x;x_*), \quad \lim _{n\rightarrow \infty }D(x;x_*^n) = D(x;x_*). \end{aligned}$$
*(c)*: Let $\{x^n_*\}_{n\in \mathbb N}\subset [x_{\text {min}},x_{\text {max}}]$ and $x_*\subset [x_{\text {min}},x_{\text {max}}]$ satisfy $\lim _{n\rightarrow \infty }x^n_*= x_*$. Assume that there exist $0<\hat{x}<x_*-\tilde{r}$ and $\kappa >0$ such that $s(x_*^n)<\hat{x}$ for all $n\in \mathbb N$ and the following uniform bound holds:
  5.321 $$\begin{aligned} B[x;x_*^n,W,D]>\kappa , \quad n\in \mathbb N, \quad x\in [\hat{x} ,x_*-\tilde{r}]. \end{aligned}$$
  Then there exists a $T=T(\kappa ,\hat{x})>0$ such that
  5.322 $$\begin{aligned} s(x_*)<\hat{x}-T, \quad s(x_*^n)<\hat{x}-T, \quad n\in \mathbb N. \end{aligned}$$

### The Friedmann Shooting Argument

The basic idea of this section is inspired by a related proof in our earlier work on the existence of Newtonian Larson-Penston solutions [15]. We start by recalling Definition 2.2.

#### Lemma 5.9

($\mathcal {X}_{>\frac{1}{3}}$ and $\mathcal {X}_{\frac{1}{3}}$ are nonempty) There exists an $0<\varepsilon _0\ll 1$ sufficiently small so that the following statements hold for any $0<\varepsilon \le \varepsilon _0$:

*(a)*: There exists a $\kappa =\kappa (\varepsilon )>0$ such that $(x_{\text {max}}(\varepsilon )-\kappa ,x_{\text {max}}(\varepsilon )]\subset X \subset \mathcal {X}_{\frac{1}{3}}$.
*(b)*: Moreover, $$x_\text {min} \in \mathcal {X}_{>\frac{1}{3}}$$.

#### Proof

*Proof of part (a).* We use the mean value theorem to write

$$\begin{aligned} W(x;x_*) = W_0 + W'(\bar{x}; x_*) (x-x_*), \ x\in (s(x_*), x_*) \end{aligned}$$

for some $\bar{x}\in (x,x_*)$. Note that $W'(x_*; x_*) =\frac{1}{x_*} \mathcal {W}_1 = \frac{1}{x_*}\left( 1-\frac{2}{x_*}+O(\varepsilon )\right) =\frac{1}{x_*}-\frac{2}{x_*^2}+O(\varepsilon )$. By (4.245), it follows that $W'(x_{\text {max}}(\varepsilon );x_{\text {max}}(\varepsilon )) = \frac{1}{9}+O(\varepsilon )$. By Theorem 4.18 there exist small enough $r>0$ and $\delta _1>0$ such that $W'(x; x_*)>\frac{1}{18}$ for all $x\in (x_*-r,x_*]$ and $x_*\in [x_{\text {max}}(\varepsilon )-\delta _1,x_{\text {max}}(\varepsilon )]$. For such $x_*$ and *x*, we have

$$\begin{aligned} W(x;x_*) \le \frac{1}{x_*} +\frac{1}{18} (x-x_*), \end{aligned}$$

where we have used $W_0=\mathcal {W}_0 <\frac{1}{x_*}$ for $\varepsilon \in (0,\varepsilon _0]$, see Lemma 4.1. Note $\frac{1}{x_*} + \frac{1}{18} (x-x_*) = \frac{1}{3}$ when $x=\tilde{x}(x_*)= x_*- \frac{6(3-x_*)}{x_*}$. Therefore for all $x_*\in (x_{\text {max}}(\varepsilon )-\kappa , x_{\text {max}}(\varepsilon )]\subset (3-\kappa ,3)$ with $\kappa = \min \{ \delta _1, x_{\text {max}}(\varepsilon )-3+\frac{3r}{6+r}\}$, there exists an $x\ge \tilde{x}(x_*)$ such that $W(x;x_*)=\frac{1}{3}$, which shows the claim. Here we have used (4.246) and the smallness of $0<\varepsilon _0\ll 1$.

*Proof of part (b).* We rewrite (2.36) in the form

5.323 $$\begin{aligned} xW' = 1- 2W - W \frac{B - 2 x^2(1+\varepsilon )(W + \varepsilon )(D- W)}{B}. \end{aligned}$$

We now recall Lemma 3.6 and express the numerator above in the form

$$\begin{aligned}&B - 2x^2(1+\varepsilon ) (W + \varepsilon )(D- W) \\&= (1-\varepsilon ) (J[D]-x W)(H[D] + x W) - 2(1+\varepsilon )x ( W + \varepsilon ) ( x D- J[D] + J[D] - x W) \\&= 2(1+\varepsilon )x( W + \varepsilon ) f+ (J[D]- x W ) \left\{ (1-\varepsilon ) (H[D] + x W) - 2(1+\varepsilon ) x( W + \varepsilon )\right\} . \end{aligned}$$

where we recall $f(x) = J[x;D]-xD$. Using (3.125),

$$\begin{aligned}&(1-\varepsilon ) (H[D] + x W) - 2(1+\varepsilon ) x( W + \varepsilon )\\&\quad = (1-\varepsilon ) ( J[D] +2\eta (1+D) x+ x W) - 2(1+\varepsilon )x ( W + \varepsilon ) \\&\quad =(1-\varepsilon ) ( J[D] - x W) + 4\varepsilon ( x D- x W) + 2\varepsilon (1-\varepsilon ) x \\&\quad = (1+3\varepsilon ) ( J[D] - x W) -4\varepsilon f + 2\varepsilon (1-\varepsilon ) x. \end{aligned}$$

Putting these together, and by (3.123) and $W>0$, (5.323) reads as

5.324 $$\begin{aligned} x W'&= 1- 2 W + 2\eta \frac{f}{H[D] + x W} W \nonumber \\&\quad - W \frac{ (1+3\varepsilon )( J[D] - x W)^2 +2(1+\varepsilon ) x( W + \varepsilon )(J[D] - x D) + 2\varepsilon (1-\varepsilon )( J[D] - x W)x }{B} \nonumber \\&\le 1- (2-2\eta ) W, \end{aligned}$$

where we have used $0<f=J[D] - x D< H[D] + x W$ and $B>0$ for $x\in (s(x_*),x_*)$. Hence the set

5.325 $$\begin{aligned} \left\{ x\in (s(x_*),x_*))\, \big | W(x;x_*)>\frac{1}{2-2\eta }\right\} \end{aligned}$$

is an invariant set.

We now use Lemma 4.20 and the definition of $x_{\text {min}}$ to obtain $W(x_0;x_{\text {min}}) >\frac{1}{2-2\eta }$ for some $x_0\in (s(x_*),x_*))$. Together with the invariance of (5.325) we conclude that $x_{\min } \in \mathcal {X}_{>\frac{1}{3}}$. $\square$

#### Remark 5.10

A corollary of Definition 2.2 and Lemma 5.9 is that the fundamental set *X* is a simply connected subset of $(x_{\text {min}},x_{\text {max}}]$ which contains the point $x_{\text {max}}$. The set *X* is in particular an interval.

Of fundamental importance in our analysis are the following two quantities.

#### Definition 5.11

(Critical point and Friedmann time) Let $\varepsilon _0>0$ be a small constant given by Theorem 4.18. For any $\varepsilon \in (0,\varepsilon _0]$ we introduce the critical point

5.326 $$\begin{aligned} \bar{x}_*: = \inf _{x_*\in X } x_*, \end{aligned}$$

and the Friedmann time

5.327 $$\begin{aligned} x_F&= x_F(x_*) : = \inf \left\{ x \in (s(x_*), x_*) \, \big | \quad W(\tau ;x_*)>\frac{1}{3} \ \text { for } \ \tau \in (x,x_*) \right\} . \end{aligned}$$

We will show later, the unique local RLP-type solution associated with the sonic point $\bar{x}_*$ is in fact *global* and extends all the way to the origin $x=0$. The Friedmann time introduced above plays a crucial role in the proof.

The sets $\mathcal {X}_{\frac{1}{3}}$ and $\mathcal {X}_{<\frac{1}{3}}$ enjoy several important properties which we prove in the next lemma.

#### Lemma 5.12

Let $\varepsilon _0>0$ be a small constant given by Theorem 4.18. Then for any $\varepsilon \in (0,\varepsilon _0]$ the following statements hold.

*(a)*: For any $x_*\in \mathcal {X}_{\frac{1}{3}}\cup \mathcal {X}_{<\frac{1}{3}}$ we have
  5.328 $$\begin{aligned} W(x;x_*)&<D(x;x_*), \quad x\in (s(x_*), x_*), \end{aligned}$$
  5.329 $$\begin{aligned} W(x;y_*)&<\frac{1}{3}, \quad x\in (s(x_*), x_{F}(x_*)), \end{aligned}$$
  where (5.329) is considered trivially true in the case $s(x_*)=x_{F}(x_*)$.
*(b)*: For any $x_*\in \mathcal {X}_{\frac{1}{3}}$ we have $W'(x_{F}(x_*);x_*)>0$. Moreover, the set $\mathcal {X}_{\frac{1}{3}}$ is relatively open in $[x_{\text {min}},x_{\text {max}}]$.

#### Proof

*Proof of Part (a).* Let $x_*\in \mathcal {X}_{\frac{1}{3}}\cup \mathcal {X}_{<\frac{1}{3}}$. Then from Lemma 4.6 we know that $W(x) <D(x)$ for all $x\in [ (1-\bar{r}) x_*, x_*)$ for some $\bar{r}\le r$ where *r* is given by Theorem 4.18. We first prove (5.328). By way of contradiction, assume that there exists $x_c\in (s(x_*), x_*)$ such that

$$\begin{aligned} W(x_c) = D(x_c) \ \text { and } \ W(x)< D(x), \ x \in (x_c, x_*). \end{aligned}$$

We distinguish three cases.

*Case 1:* $x_c\in (x_F, x_*)$. In this case, from (2.35) and (2.36), we have $W'(x_c) <0$ and $D'(x_c)=0$. In particular, $(D-W)'(x_c)>0$ and locally strictly to the left of $x_c$ we have

$$\begin{aligned} W' <0, \ W - D>0, \ D'>0, \ W>\frac{1}{3} . \end{aligned}$$

We observe that these conditions are dynamically trapped and since $W'<0$, we deduce that *W* stays strictly bounded away from $\frac{1}{3}$ from above for all $x\in (x_F, x_*)$, which is a contradiction to the assumption $x_*\in \mathcal {X}_{\frac{1}{3}}\cup \mathcal {X}_{<\frac{1}{3}}$.

*Case 2:* $x_c=x_F$. In this case, $x_*\in \mathcal {X}_{\frac{1}{3}}$ necessarily and $W(x_c) = D(x_c)=\frac{1}{3}$. On the other hand, since $D-W>0$ for $x\in (x_F, x_*)$, it follows $D'<0$ on $(x_F, x_*)$ from (2.35). Hence, $D(x_c) > D(x_*)=\mathcal {W}_0 \ge \frac{1}{3}$ since $x_*\in [x_{\text {min}},x_{\text {max}}]$. This is a contradiction.

*Case 3:* $x_c\in (s(x_*), x_F)$. In this case, $x_*\in \mathcal {X}_{\frac{1}{3}}$ necessarily. Since $x_c<x_F$ we know that $D-W>0$ locally around $x_F$. Therefore, we have

$$\begin{aligned} W'>0, \ D-W >0, \ W<\frac{1}{3} \ \text { on } (x_F-\delta , x_F) \end{aligned}$$

for a sufficiently small $\delta >0$. These conditions are dynamically trapped and we conclude that $D-W>0$ on $(s(x_*),x_F)$. This is a contradiction, and hence completing the proof of (5.328).

Inequality (5.329) follows by a similar argument, since the property $W'>0, \ D-W >0, \ W<\frac{1}{3}$ is dynamically preserved and all three conditions are easily checked to hold locally to the left of $x_F(x_*)$.

*Proof of Part (b).* For any $x_*\in \mathcal {X}_{\frac{1}{3}}$ by part (a) and (2.36) we have $W'( x_F(x_*); x_*) >0$. Therefore there exists a sufficiently small $\delta >0$ so that $W(x; x_*)<\frac{1}{3}$ for all $x\in (x_F(x_*)-\delta , x_F(x_*))$. By Proposition 5.8, there exists a small neighborhood of $x_*$ such that $W(x;x_*)<\frac{1}{3}$ for some $x\in (x_F(x_*)-\delta , x_F(x_*))$. Therefore $\mathcal {X}_{\frac{1}{3}}$ is open. $\square$

#### Lemma 5.13

Let $\varepsilon _0>0$ be a small constant given by Theorem 4.18 and for any $x_*\in [x_{\text {min}},x_{\text {max}}]$ consider the unique local RLP-type solution given by Theorem 4.18. If $x_F(x_*)=s(x_*)>0$ then necessarily

5.330 $$\begin{aligned} W(x_F(x_*);x_*)>\frac{1}{3}. \end{aligned}$$

In particular if $x_*\in X$, then necessarily $s(x_*)<x_F(x_*)$.

#### Proof

Assume the claim is not true, in other words $W(x_F(x_*);x_*)=\frac{1}{3}$. Since $D$ is decreasing on $(x_F,x_*-\delta ]$ by Lemma 5.12 and (2.35) and since $D(x_*) \ge 1/3$ it follows that $\lim _{x\rightarrow x_F}D(x)>\frac{1}{3}$. In particular, since $J\ge xD$ on $(s(x_*),x_*-\delta )$ by (5.310), it follows from (3.123) that $B(x;x_*) \ge (1-\varepsilon )x(D-W)(H(x)+xW)$. Therefore

$$\begin{aligned} \liminf _{x\rightarrow x_F} B(x)>0, \end{aligned}$$

a contradiction to the assumption $x_F(x_*)=s(x_*)$ due to Lemma 5.6. The second claim is a consequence of the just proven claim and the definition of the set *X*. $\square$

#### Lemma 5.14

Let $\varepsilon _0>0$ be a small constant given by Theorem 4.18 and for any $x_*\in \mathcal {X}_{>\frac{1}{3}}\subset [x_{\text {min}},x_{\text {max}}]$ consider the unique local RLP-type solution given by Theorem 4.18. Then there exists a monotonically increasing continuous function

$$\begin{aligned} m:(0,x_*-\tilde{r}]\rightarrow (0,\infty ), \end{aligned}$$

such that for any $X\in (0,x_*-\tilde{r}]$

5.331 $$\begin{aligned} B(x;x_*)>m (X)>0 \; \text { for all } \;x\in (\max (X,s(x_*)),x_*-\tilde{r}]. \end{aligned}$$

#### Proof

Since $x_*\in \mathcal {X}_{\frac{1}{3}}$, by Lemma 5.12$J-xW\ge J-xD= f$ on $(s(x_*),x_*-\tilde{r})$. Since $H+xW\ge J-xW\ge f$ we conclude from (3.123) that $B\ge (1-\varepsilon )f^2$. Since $W(x;x_*)\ge \frac{1}{3}$ for all $x\in [x_F(x_*), x_*-\tilde{r}]$, it follows from Lemma 5.12 that $D>\frac{1}{3}$ on the same interval. Therefore, by Lemma 3.9 we conclude that $b[x;D,W]<0$ on $[x_F(x_*), x_*-\tilde{r}]$. By Corollary 3.8 we then conclude

5.332 $$\begin{aligned} f(x)\ge f(x_*-\tilde{r}) e^{\int _{x}^{x_*-\tilde{r}} a[z;D,W]\,dz}, \quad x\in [x_F(x_*), x_*-\tilde{r}]. \end{aligned}$$

From (3.132) it is clear that on $[x_F(x_*), x_*-\tilde{r}]$,

$$\begin{aligned} a_2&= 2\varepsilon \left[ \left( J-xW\right) \left( D-1\right) + 2f + 4\varepsilon x + xD\left( 5+\varepsilon \right) \right] Z^{-1} \\&= 2\varepsilon \left[ \left( J-xW\right) \left( D+1\right) + 2xW + 4\varepsilon x + xD\left( 3+\varepsilon \right) \right] Z^{-1}>0. \end{aligned}$$

Similarly, the first line of (3.131) is strictly positive on the same interval, and we conclude from (5.332) and (3.131) that

5.333 $$\begin{aligned} f(x)\ge f(x_*-\tilde{r}) e^{ -2\varepsilon \int _{x}^{x_*-\tilde{r}} \left( \frac{1}{1-\varepsilon }\frac{ D^{-\eta }}{z} + (1-\varepsilon )z + (D+\varepsilon ) f \right) Z^{-1} \,dz}, \ \ x\in [x_F(x_*), x_*-\tilde{r}]. \end{aligned}$$

From (2.46), $W\ge \frac{1}{3}$ on $[x_F(x_*), x_*-\tilde{r}]$, and $H\ge J$ we have $Z\ge \max \{ \frac{2}{3}\varepsilon z^2, \frac{1}{3} Jz\}$ on $[x_F(x_*), x_*-\tilde{r}]$. Similarly,

$$\begin{aligned} \begin{aligned}&2\varepsilon \left( \frac{1}{1-\varepsilon }\frac{ D^{-\eta }}{z} + (1-\varepsilon )z+ (D+\varepsilon ) f \right) Z^{-1} \\&\le 2\varepsilon \left( \frac{3^\eta }{1-\varepsilon } \frac{1}{z} + (1-\varepsilon ) x_*\right) \frac{3}{2\varepsilon z^2} + 2\varepsilon \frac{(M+\varepsilon ) J}{ \frac{1}{3} Jz}\le C_1 \left( \frac{1}{z^3}+\frac{1}{z^2}+ \frac{1}{z}\right) \le C_2\frac{1}{z^3}, \end{aligned} \end{aligned}$$

for and some $C_1,C_2>0$ where we recall the upper bound *M* of $D$ in (5.316). Combining the two bounds and plugging it back into (5.333) we get

5.334 $$\begin{aligned} f(x) \ge f(x_*-\tilde{r}) e^{- C_2\int _x^{x_*-\tilde{r}}\frac{1}{z^3}\,dz} \ge f(x_*-\tilde{r}) e^{-\frac{C_2}{2} x^{-2}}. \end{aligned}$$

Together with the established lower bound $B\ge (1-\varepsilon )f^2$ we conclude the proof. $\square$

#### Proposition 5.15

The unique RLP-type solution associated with $\bar{x}_*\in (x_{\text {min}},x_{\text {max}})$ exists globally to the left, i.e. $s(\bar{x}_*)=0.$

#### Proof

*Case 1.* We assume $x_F(\bar{x}_*)=0$. Since $0\le s(\bar{x}_*) \le x_F(\bar{x}_*)$ we are done.

*Case 2.* We assume $x_F(\bar{x}_*)>s(\bar{x}_*)>0$. In this case $\bar{x}_*\in X$ which is impossible, since *X* is relatively open in $[x_{\text {min}},x_{\text {max}}]$ by Lemma 5.12 and $x_{\text {min}}\notin X$ by Lemma 5.9.

*Case 3.* We now assume that $x_F(\bar{x}_*)=s(\bar{x}_*)>0$. By Lemma 5.13 this in particular implies that

5.335 $$\begin{aligned} \bar{x}_*\in [x_{\text {min}},x_{\text {max}}]\setminus \mathcal {X}_{\frac{1}{3}}\end{aligned}$$

and

$$\begin{aligned} W(x_F(\bar{x}_*);\bar{x}_*)>\frac{1}{3}. \end{aligned}$$

Consider now a sequence $\{x_*^n\}_{n\in \mathbb N}\subset X$ such that $\lim _{n\rightarrow \infty }x_*^n = \bar{x}_*$. We consider

$$\begin{aligned} \bar{x}_F : = \limsup _{n\rightarrow \infty } x_F(x_*^n) \end{aligned}$$

and after possibly passing to a subsequence, we assume without loss that $\lim _{n\rightarrow \infty } x_F(x_*^n)=\bar{x}_F$. We now consider two possibilities.

*Case 3 a).* Assume that $\bar{x}_F>0$. Since $\{x_*^n\}_{n\in \mathbb N}\subset X$, by Lemma 5.13 necessarily $s(x_*^n)<x_F(x_*^n)$, $n\in \mathbb N$. Upon possibly passing to a further subsequence, we can ascertain that there exists some $\delta >0$ such that $\bar{x}_F>x_F(x_*^n)>\bar{x}_F-\delta >0$ for all $n\in \mathbb N$. By Lemma 5.14 we conclude in particular that

5.336 $$\begin{aligned} B(x;x_*^n)>m(\bar{x}_F-\delta )>0 \ \ \text { for all } \ \ x\in [x_F(x_*^n),x_*-\tilde{r}], \ \ n\in \mathbb N. \end{aligned}$$

Therefore, by part (c) of Proposition 5.8, there exists $T=T(m(\bar{x}_F-\delta ),\bar{x}_F)$ such that

5.337 $$\begin{aligned} s(\bar{x}_*)<\bar{x}_F-T, \ \ s(x_*^n)<\bar{x}_F - T, \ \ n\in \mathbb N. \end{aligned}$$

Fix an $x\in (\bar{x}_F-T,\bar{x}_F)$ and observe that

5.338 $$\begin{aligned} W(x;\bar{x}_*)=\lim _{n\rightarrow \infty }W(x,x_*^n)\le \frac{1}{3}, \end{aligned}$$

where we have used Lemma 5.12. This implies $\bar{x}_*\in \mathcal {X}_{\frac{1}{3}}$, a contradiction to (5.335).

*Case 3 b).* Assume that $\bar{x}_F=0$. For any fixed $\hat{x}>0$ we can apply the argument from *Case 3 a)* to conclude that $s(\bar{x}_*)<\hat{x}$. Therefore $s(\bar{x}_*)=0$ in this case. $\square$

#### Lemma 5.16

(Continuity of $\mathcal {X}_{\frac{1}{3}}\ni x_*\mapsto x_{F}(x_*)$) The map

$$\begin{aligned} \mathcal {X}_{\frac{1}{3}}\ni x_*\mapsto x_{F}(x_*) \end{aligned}$$

is continuous and

5.339 $$\begin{aligned} \lim _{X \ni x\rightarrow \bar{x}_*} x_{F}(x) = 0 = x_{F}(\bar{x}_*). \end{aligned}$$

#### Proof

The proof is nearly identical to the proof of Lemma 4.13 in [15]. $\square$

### The Solution from the Origin $x=0$ to the Right

In this section we consider the solutions $(D_-,W_-)$ to (2.35)–(2.36) generated by the data imposed at $x=0$ and satisfying $W_-(0)=\frac{1}{3}$. Upon specifying the value $D_0=D_-(0)>0$, the problem is well-posed by Theorem 4.24 on some interval [0, *r*].

#### Definition 5.17

We introduce the sonic time from the left

5.340 $$\begin{aligned} s_-(D_0){:}{=} \sup _{x\ge 0}\{x\ \big | \ J[x;D_-(x;D_0)] - x W_- (x;D_0)>0 \}. \end{aligned}$$

#### Lemma 5.18

Let $D_-(0)=D_0>\frac{1}{3}$, $W_-(0)=\frac{1}{3}$ and $x_*\in [x_{\text {min}},x_{\text {max}}]$. The solution $(D_-(x;D_0), W_-(x;D_0))$ to (2.35)–(2.36) with the initial data

5.341 $$\begin{aligned} D_-(0,D_0)=D_0\ge \frac{1}{3}, \quad W_-(0;D_0)= \frac{1}{3}, \end{aligned}$$

exists on the interval $[0, s_-(D_0))$ and satisfies the following bounds: for $x\in (0, s_-(D_0))$

5.342 $$\begin{aligned} W_-&> \frac{1}{3} , \end{aligned}$$

5.343 $$\begin{aligned} \frac{ D_-}{1-\varepsilon }+ \frac{ W_-}{1+\varepsilon }&<\frac{D_0}{1-\varepsilon }+ \frac{1}{3(1+\varepsilon )}, \end{aligned}$$

5.344 $$\begin{aligned} D_-^{1+\varepsilon } W_-^{1-\varepsilon }&< \frac{ D_-^{1+\varepsilon } }{ 3^{1-\varepsilon }}, \end{aligned}$$

5.345 $$\begin{aligned} D_-&> W_- , \end{aligned}$$

5.346 $$\begin{aligned} D_-'&<0. \end{aligned}$$

#### Proof

The proof is analogous to the proof of Lemma 4.14 from [15]. $\square$

In the following lemma we identify a spatial scale $x_0\sim \frac{1}{D_0^{1+O(\varepsilon )}}$ over which we obtain quantitative lower bounds on the density $D_-$ over $[0,x_0]$.

#### Lemma 5.19

(Quantitative lower bounds on $D_-$) Let $D_0>\frac{1}{3}$ and $x_*\in [x_{\text {min}},x_{\text {max}}]$ be given and consider the unique solution $(D_-(\cdot ;D_0), W_-(\cdot ;D_0))$ to the initial-value problem (2.35)–(2.36), (5.341). For any $D_0>\frac{1}{3}$ let

5.347 $$\begin{aligned} x_0=x_0(D_0): = {\left\{ \begin{array}{ll} \frac{3^{\frac{1-\varepsilon }{2}}}{2^\frac{1}{2} (1+8\varepsilon )^\frac{1}{2}D_0^{\frac{1}{1-\varepsilon } } }, &{} D_0> 1; \\ \frac{3^{\frac{1-\varepsilon }{2}}}{2^\frac{1}{2} (1+8\varepsilon )^\frac{1}{2} }, &{} \frac{1}{3} < D_0\le 1. \end{array}\right. } \end{aligned}$$

Then $s_-(D_0)>x_0$ for all $D_0>\frac{1}{3}$ and

5.348 $$\begin{aligned} D_-(x;D_0) \ge {\left\{ \begin{array}{ll} D_0 \exp \left( - D_0^{-1+\varepsilon }\right) , &{} D_0>1;\\ D_0 \exp \left( -1\right) , &{} \frac{1}{3} < D_0\le 1, \end{array}\right. } \ \ x\in [0, x_0]. \end{aligned}$$

Moreover, for all sufficiently small $\varepsilon >0$, there exists a $\hat{D}>1$ such that for all $D_0>\hat{D}$ we have

5.349 $$\begin{aligned} D_-(x_0;D_0)&>\frac{1}{x_0^{1-\varepsilon }}. \end{aligned}$$

#### Proof

Equation (2.35) is equivalent to

5.350 $$\begin{aligned} D_-(x) = D_0 \exp \left( -\int _0^x\frac{2 \tau (1-\varepsilon )( W_-+\varepsilon )(D_-- W_-)}{B}\,d\tau \right) . \end{aligned}$$

Since $W_- =( W_-^{1+\varepsilon } W_-^{1-\varepsilon })^\frac{1}{2}< (D_-^{1+\varepsilon } W_-^{1-\varepsilon })^\frac{1}{2}$ and $D_- W_- = (D_-^{1+\varepsilon } W_-^{1-\varepsilon })^{\frac{1}{1+\varepsilon }} W_-^{\frac{2\varepsilon }{1+\varepsilon }}$, by Lemma 5.18, we have the following bounds on the interval $(0, s_-(D_0))$

5.351 $$\begin{aligned} W_-<D_-&<D_0, \end{aligned}$$

5.352 $$\begin{aligned} \frac{1}{3}< W_-&<\left( \frac{D_0^{1+\varepsilon }}{3^{1-\varepsilon }}\right) ^\frac{1}{2}, \end{aligned}$$

5.353 $$\begin{aligned} D_- W_-&< \frac{D_0^{1+\varepsilon }}{3^{1-\varepsilon }}. \end{aligned}$$

Now from the definition (2.37) of *B*, for any $0\le x\le x_0$ using (5.351)-(5.353) and the definition of $x_0$ in (5.347), we have

5.354 $$\begin{aligned} D_-^{\frac{2\varepsilon }{1-\varepsilon }} B&=1 -D_-^{\frac{2\varepsilon }{1-\varepsilon }} \left[ (1-\varepsilon ) W_-^2 + 4\varepsilon D_- W_- + 4\varepsilon W_-+ \varepsilon ^2-\varepsilon \right] x^2\nonumber \\&\ge 1 -D_0^{\frac{2\varepsilon }{1-\varepsilon }}\left[ (1+3\varepsilon )\frac{D_0^{1+\varepsilon }}{3^{1-\varepsilon }} + 4\varepsilon \left( \frac{D_0^{1+\varepsilon }}{3^{1-\varepsilon }}\right) ^\frac{1}{2}+ \varepsilon ^2-\varepsilon \right] x^2\nonumber \\&\ge 1 -D_0^{\frac{2\varepsilon }{1-\varepsilon }} (1+8\varepsilon ) \frac{D_0^{1+\varepsilon }}{3^{1-\varepsilon }} x^2\nonumber \\&\ge {\left\{ \begin{array}{ll} 1- \frac{1}{2D_0^{1-\varepsilon }} \ge \frac{1}{2} ,&{} D_0>1; \\ 1- \frac{D_0^{1+\varepsilon +\frac{2\varepsilon }{1-\varepsilon }}}{2} \ge \frac{1}{2}, &{} \frac{1}{3}<D_0\le 1. \end{array}\right. } \end{aligned}$$

Note from the second line to the third line we have used the upper bound of $[\cdot ]$ term

5.355 $$\begin{aligned} \frac{D_0^{1+\varepsilon }}{3^{1-\varepsilon }} \le (1+3\varepsilon )\frac{D_0^{1+\varepsilon }}{3^{1-\varepsilon }} + 4\varepsilon \left( \frac{D_0^{1+\varepsilon }}{3^{1-\varepsilon }}\right) ^\frac{1}{2}+ \varepsilon ^2-\varepsilon \le (1+8\varepsilon ) \frac{D_0^{1+\varepsilon }}{3^{1-\varepsilon }}, \end{aligned}$$

which holds true for $D_0>\frac{1}{3}$ and sufficiently small $0<\varepsilon$. Therefore, $s_-(D_0)>x_0$ for all $D_0>\frac{1}{3}$.

From (5.351),  (5.353), and (5.354) for any $x\in [0,x_0]$ we obtain

$$\begin{aligned}&\int _0^x\frac{2 \tau (1-\varepsilon ) ( W_-+\varepsilon )(D_-- W_-)}{ B}\,d\tau \le 4 (1-\varepsilon )D_0^{\frac{2\varepsilon }{1-\varepsilon }} ( \frac{D_0^{1+\varepsilon }}{3^{1-\varepsilon }} + \varepsilon D_0) \int _0^x\tau \,d\tau \\&\le 2x_0^2 (1-\varepsilon ) D_0^{\frac{2\varepsilon }{1-\varepsilon }} ( \frac{D_0^{1+\varepsilon }}{3^{1-\varepsilon }} + \varepsilon D_0) = {\left\{ \begin{array}{ll} \frac{1}{D_0^{1-\varepsilon }}\frac{(1-\varepsilon )( \frac{D_0^{1+\varepsilon }}{3^{1-\varepsilon }} + \varepsilon D_0)}{ (1+8\varepsilon ) \frac{D_0^{1+\varepsilon }}{3^{1-\varepsilon }} }, &{} D_0>1;\\ D_0 \frac{(1-\varepsilon ) D_0^\frac{2\varepsilon }{1-\varepsilon }( D_0^{\varepsilon } + \varepsilon 3^{1-\varepsilon } )}{ (1+8\varepsilon ) } ,&{} \frac{1}{3}<D_0\le 1 . \end{array}\right. } \end{aligned}$$

Now comparing the denominator and numerator of the last fractions, we see that

$$\begin{aligned} \begin{aligned}&(1+8\varepsilon ) \frac{D_0^{1+\varepsilon }}{3^{1-\varepsilon }} - (1-\varepsilon )( \frac{D_0^{1+\varepsilon }}{3^{1-\varepsilon }} + \varepsilon D_0) = \varepsilon \left[ 9\frac{D_0^{1+\varepsilon }}{3^{1-\varepsilon }} - (1-\varepsilon )D_0 \right] \ge 0 \ \text { for all } \ D_0 > 1 \end{aligned} \end{aligned}$$

and

$$\begin{aligned} \begin{aligned} 1+8\varepsilon - (1-\varepsilon ) D_0^\frac{2\varepsilon }{1-\varepsilon }( D_0^{\varepsilon } + \varepsilon 3^{1-\varepsilon } )&\ge 1+8\varepsilon - (1-\varepsilon ) (1+\varepsilon 3^{1-\varepsilon }) \\&= \varepsilon (9-(1-\varepsilon ) 3^{1-\varepsilon }) \ge 0 \ \text { for all } \ \frac{1}{3}<D_0\le 1 \end{aligned} \end{aligned}$$

and hence we deduce that for any $x\in [0,x_0]$

$$\begin{aligned} \int _0^x\frac{2\tau (1-\varepsilon ) ( W_-+\varepsilon )(D_-- W_-)}{ B}\,d\tau \le {\left\{ \begin{array}{ll} \frac{1}{D_0^{1-\varepsilon }}, &{} D_0>1;\\ 1, &{} \frac{1}{3}<D_0\le 1 . \end{array}\right. } \end{aligned}$$

Plugging this bound in (5.350) we obtain (5.348).

To show (5.349), we first rewrite (5.347) for $D_0>1$ as

5.356 $$\begin{aligned} D_0 = \frac{1}{x_0^{1-\varepsilon }} \left( \frac{3^{1-\varepsilon }}{2(1+8\varepsilon )} \right) ^{\frac{1-\varepsilon }{2}}. \end{aligned}$$

Using this in (5.348)

5.357 $$\begin{aligned} \begin{aligned} D_-(x_0; D_0)&\ge D_0 e^{-\frac{1}{D_0^{1-\varepsilon }}} = \frac{1}{x_0^{1-\varepsilon }} \left( \frac{3^{1-\varepsilon }}{2(1+8\varepsilon )} \right) ^{\frac{1-\varepsilon }{2}} e^{-\frac{1}{D_0^{1-\varepsilon }}}. \end{aligned} \end{aligned}$$

The bound (5.349) follows if $\left( \frac{3^{1-\varepsilon }}{2(1+8\varepsilon )} \right) ^{\frac{1-\varepsilon }{2}} e^{-\frac{1}{D_0^{1-\varepsilon }}}>1$ which is clearly true for sufficiently large $D_0$ and sufficiently small $\varepsilon$. $\square$

#### Remark 5.20

The specific choice of $x_0$ in (5.347) has been made to ensure the lower bound in (5.349) to be compared with the bound (5.314) satisfied by the solutions emanating from the sonic point. In fact, better bounds are obtained by choosing different $x_0$. For instance, if $x_0= O(\frac{1}{D_0^{1+\alpha }})$ for $\frac{\varepsilon }{1-\varepsilon }+\frac{\varepsilon }{2}+\frac{1}{2}<1+\alpha \le \frac{1}{1-\varepsilon }$ in (5.347), then we may deduce that for such $x_0$, $D_-(x_0;D_0) > \frac{1}{x_0^{\frac{1}{1+\alpha }} } \ge \frac{1}{ x_0^{1-\varepsilon }}$ with the equality being valid for $\alpha =\frac{\varepsilon }{1-\varepsilon }$ for all sufficiently large $D_0$ and small $\varepsilon$.

#### Remark 5.21

Since the mapping $D_0\mapsto x_0(D_0)$ from (5.347) is nonincreasing, it follows that for any fixed $D_0>\frac{1}{3}$ we have the uniform bound on the sonic time:

$$\begin{aligned} s_-(\tilde{D}_0)>x_0(\tilde{D}_0)\ge x_0(D_0), \ \ \text { for all }\ \ \frac{1}{3} <\tilde{D}_0\le D_0. \end{aligned}$$

The following lemma shows the crucial monotonicity property of $D_-(\cdot ;D_0)$ with respect to $D_0$ on a time-scale of order $\sim D_0^{-(\frac{3}{4}+O(\varepsilon ))}$.

#### Lemma 5.22

Let $x_*\in [x_{\text {min}},x_{\text {max}}]$. There exists a sufficiently small $\kappa >0$ such that for all $D_0\ge \frac{1}{3}$

$$\begin{aligned} \partial _{D_0}D_-(x;D_0)>0 \ \ \text { for all } \ x\in [0, \kappa D_0^{-b}], \ \text { where } \ b= \tfrac{3+\varepsilon +2\eta }{4} \end{aligned}$$

#### Proof

We introduce the short-hand notation $\partial D_- = \partial _{ D_0} D_-$ and $\partial W_- = \partial _{ D_0} W_-$, where we note that the map $D_0\mapsto (D_-,W_-)$ is $C^1$ by Remark 4.25. It is then easy to check that $(\partial D_-,\partial W_-)$ solve

5.358 $$\begin{aligned} \partial W_-'&= - \frac{3}{x} \partial W_- - \frac{2 x (1+\varepsilon ) W_- ( W_- +\varepsilon )}{B}\partial W_- \nonumber \\&\ \ \ \ + \frac{2 x(1+\varepsilon )( D_- - W_-)}{B} \left( 2 W_- +\varepsilon - \tfrac{\mathfrak D_ W B}{B} W_-( W_-+\varepsilon )\right) \partial W_- \end{aligned}$$

5.359 $$\begin{aligned}&\ \ \ \ + \frac{2 x (1+\varepsilon ) W_- ( W_- +\varepsilon )}{B} \left( 1- \tfrac{\mathfrak D_{ D} B}{B} ( D_-- W_-) \right) \partial D_- , \nonumber \\ \partial D_-'&= - \frac{2 x(1-\varepsilon )( W_-+\varepsilon )}{B} \left( 2 D_- - W_- -\tfrac{\mathfrak D_{ D} B}{B} D_- ( D_-- W_-) \right) \partial D_-\nonumber \\&\ \ \ \ -\frac{2 x(1-\varepsilon ) D_-}{B} \left( D_- -2 W_--\varepsilon -\tfrac{\mathfrak D_ W B}{B} ( W_-+\varepsilon )( D_-- W_-) \right) \partial W_-, \end{aligned}$$

where

$$\begin{aligned} \mathfrak D_ W B = - [2(1-\varepsilon ) W + 4\varepsilon (1+ D)] x^2, \quad \mathfrak D_ DB = -\tfrac{2\varepsilon }{1-\varepsilon } D^{-\frac{2\varepsilon }{1-\varepsilon }-1} - 4\varepsilon W x^2. \end{aligned}$$

At $x=0$ we have the initial values

5.360 $$\begin{aligned} \partial D_-(0) = 1, \ \ \partial W_-(0)=0. \end{aligned}$$

We multiply (5.358) by $\partial W_-$ and integrate over the region [0, *x*]. By (5.360) we obtain

5.361 $$\begin{aligned}&\frac{1}{2}\partial W_-^2(x) + \int _0^x \left( \frac{3}{\tau }+\frac{2 \tau (1+\varepsilon ) W_- ( W_- +\varepsilon )}{B} \right) \partial W_-^2 \,d\tau \nonumber \\&= \int _0^x \frac{2 \tau (1+\varepsilon )( D_- - W_-)}{B} \left( 2 W_- +\varepsilon - \tfrac{\mathfrak D_ W B}{B} W_-( W_-+\varepsilon )\right) \partial W_-^2\,d\tau \nonumber \\&\ \ \ \ + \int _0^x \frac{2 \tau (1+\varepsilon ) W_- ( W_- +\varepsilon )}{B} \left( 1- \tfrac{\mathfrak D_{ D} B}{B} ( D_-- W_-) \right) \partial D_- \partial W_-\,d\tau . \end{aligned}$$

Just like in (5.354), by using (5.351)–(5.353), and (5.355) we have

$$\begin{aligned} \begin{aligned} D_-^{\frac{2\varepsilon }{1-\varepsilon }} B&\ge 1 - D_0^{\frac{2\varepsilon }{1-\varepsilon }}\left[ (1+3\varepsilon )\frac{ D_0^{1+\varepsilon }}{3^{1-\varepsilon }} + 4\varepsilon \left( \frac{ D_0^{1+\varepsilon }}{3^{1-\varepsilon }}\right) ^\frac{1}{2}+ \varepsilon ^2-\varepsilon \right] \tau ^2 \\&\ge 1- (1+8\varepsilon ) D_0^{\frac{2\varepsilon }{1-\varepsilon }} \frac{ D_0^{1+\varepsilon }}{3^{1-\varepsilon }} \tau ^2 . \end{aligned} \end{aligned}$$

Therefore

5.362 $$\begin{aligned} D_-^{\frac{2\varepsilon }{1-\varepsilon }} B \ge \frac{1}{2}, \text { for any } \ \tau \in [0, ( 8 D_0^{1+\varepsilon +\frac{2\varepsilon }{1-\varepsilon }} )^{-\frac{1}{2}}] \end{aligned}$$

where we have used $2(1+8\varepsilon ) 3^{\varepsilon -1} < 8$ for all $x_*\le x_{\text {max}}$ and sufficiently small $\varepsilon$.

Using the bounds $x_*\le x_{\text {max}}$, (5.351)–(5.353), (5.362), we obtain from (5.361)

5.363 $$\begin{aligned}&\frac{1}{2}\partial W_-^2(x) + \int _0^x \left( \frac{3}{\tau }+\frac{2 \tau (1+\varepsilon ) W_- ( W_- +\varepsilon )}{B} \right) \partial W_-^2 \,d\tau \nonumber \\&\le C \int _0^x D_0^{1+\varepsilon +\frac{2\varepsilon }{1-\varepsilon }} \tau \partial W_-^2\,d\tau + C D_0^{1+\varepsilon +\frac{2\varepsilon }{1-\varepsilon }} \int _0^x \tau |\partial D_-| |\partial W_-| \, d\tau , \ \ x\le ( 8 D_0^{1+\varepsilon +\frac{2\varepsilon }{1-\varepsilon }} )^{-\frac{1}{2}} \end{aligned}$$

for all sufficiently small $\varepsilon$. Here we have used $-\tfrac{\mathfrak D_DB}{B} (D_--W_-) >0$ and $\tfrac{\mathfrak D_DB}{B} W_- <0$, and

$$\begin{aligned} \begin{aligned} 0<- \tfrac{\mathfrak D_{ D} B}{B} ( D_-- W_- )&<- \tfrac{\mathfrak D_{ D} B}{B} D_- = - \tfrac{\mathfrak D_{ D} B}{D_-^{\eta }B} D_-^{1+\eta } \\&\le C_1\varepsilon ( 1 + D_-^{1+\eta } W_- \tau ^2 ) \le C_1\varepsilon (1+ \frac{D_0^{1+\varepsilon + \eta }}{3^{1-\varepsilon }}\tau ^2)\le C_2 \varepsilon \end{aligned} \end{aligned}$$

for any $\tau \in [0, ( 8 D_0^{1+\varepsilon +\frac{2\varepsilon }{1-\varepsilon }} )^{-\frac{1}{2}}]$ so that $\left( 1- \tfrac{\mathfrak D_{ D} B}{B} ( D_-- W_-) \right)$ is bounded by a constant.

Let $$\hat{X} = \kappa D_0^{-b}$$ where $$b=\frac{3+\varepsilon +\frac{4\varepsilon }{1-\varepsilon }}{4}$$ with a sufficiently small $$\kappa >0$$ to be specified later. Note that $$\hat{X}< ( 8 D_0^{1+\varepsilon +\frac{2\varepsilon }{1-\varepsilon }} )^{-\frac{1}{2}}$$ for all $$ D_0\ge \frac{1}{3}$$ and $$\kappa $$ chosen sufficiently small and independent of $$ D_0$$. For any $$\tau \in [0,\hat{X}]$$ we have $$ D_0 \le \kappa ^{\frac{1}{b}} \tau ^{-\frac{1}{b}}$$. Therefore $$ D_0^{1+\varepsilon +\frac{2\varepsilon }{1-\varepsilon }} \tau \le \kappa ^{\frac{1}{b}( 1+\varepsilon +\frac{2\varepsilon }{1-\varepsilon }) } \tau ^{-\frac{1}{b}+1}$$. From these estimates and (5.363) we conclude for $$ x\in [0,\hat{X}]$$,

5.364 $$\begin{aligned}&\frac{1}{2}\partial W_-^2(x) + \int _0^x \left( \frac{3}{\tau }+\frac{2 \tau (1+\varepsilon ) W_- ( W_- +\varepsilon )}{B} \right) \partial W_-^2 \,d\tau \nonumber \\&\quad \le C \int _0^x \kappa ^{\frac{1}{b}( 1+\varepsilon +\frac{2\varepsilon }{1-\varepsilon }) } \tau ^{-\frac{1}{b}+1}\partial W_-^2\,d\tau + \frac{C}{\sqrt{3}} D_0^{1+\varepsilon +\eta } \left( \int _0^x\frac{3}{\tau }\partial W_-^2\,d\tau \right) ^{\frac{1}{2}} \left( \int _0^x \tau ^3 \partial D_-^2 \, d\tau \right) ^{\frac{1}{2}} \nonumber \\&\quad \le C \int _0^x \kappa ^{\frac{1}{b}( 1+\varepsilon +\frac{2\varepsilon }{1-\varepsilon }) } \tau ^{-\frac{1}{b}+1}\partial W_-^2\,d\tau + \frac{1}{2} \int _0^x\frac{3}{\tau }\partial W_-^2\,d\tau + \frac{C^2}{6} D_0^{2+2\varepsilon +2\eta } \Vert \partial D_-\Vert _{\infty }^2 \int _0^x \tau ^3\,d\tau . \end{aligned}$$

Since $$-\frac{1}{b}+1>-1$$, with $$\kappa $$ chosen sufficiently small, but independent of $$ D_0$$, we can absorb the first two integrals on the right-most side into the term $$\int _0^x \frac{3}{\tau }\partial W_-^2\,d\tau $$ on the left-hand side. We then conclude

5.365 $$\begin{aligned} \left| \partial W_-(x)\right| \le C D_0^{1+\varepsilon +\eta } x^2 \Vert \partial D_-\Vert _{\infty }, \ \ x\in [0,\hat{X}]. \end{aligned}$$

We now integrate (5.359), use (5.351)–(5.353), (5.362), and conclude from (5.360)

5.366 $$\begin{aligned} \left| \partial D_-(x) - 1\right|&\le C D_0^{1+\varepsilon +\frac{2\varepsilon }{1-\varepsilon }} \Vert \partial D_-\Vert _{\infty } \int _0^x \tau \,d\tau \nonumber \\&\ \ \ \ + C \int _0^x \left( D_0^{2+\eta } \tau + \left[ D_0^{2+2\varepsilon +2\eta } + \varepsilon D_0^{3+\varepsilon +2\eta }\right] \tau ^3\right) \left| \partial W_-(\tau )\right| \,d\tau \nonumber \\&\le C\left( D_0^{1+\varepsilon +\eta } x^2 + D_0^{3+\varepsilon +2\eta } x^4 + ( D_0^{3+3\varepsilon +3\eta } + \varepsilon D_0^{4+2\varepsilon +3\eta } ) x^6 \right) \Vert \partial D_-\Vert _{\infty } \nonumber \\&\le C\kappa ^2 \Vert \partial D_-\Vert _{\infty }, \ \ x\in [0,\hat{X}], \end{aligned}$$

for all sufficiently small $\varepsilon$, where we have used (5.365) and $0\le x\le \kappa D_0^{-b}$. Therefore,

$$\begin{aligned} \Vert \partial D_-\Vert _{\infty } \le 1 + C\kappa ^2 \Vert \partial D_-\Vert _{\infty } \end{aligned}$$

and thus, for $\kappa$ sufficiently small so that $C\kappa ^2<\frac{1}{3}$, we have $\Vert \partial D_-\Vert _\infty \le \frac{3}{2}$. From here we infer

$$\begin{aligned} \partial D_-(x) \ge 1 - \frac{3}{2}C\kappa ^2>\frac{1}{2}>0, \ \ x\in [0,\hat{X}]. \end{aligned}$$

$$\square $$

### Upper and Lower Solutions

#### Definition 5.23

(Upper and lower solution) For any $x_*\in [x_{\text {min}},x_{\text {max}}]$ we say that $(D(\cdot ;x_*),W(\cdot ;x_*))$ is an *upper* (resp. *lower*) solution at $x_0\in (0,x_*)$ if there exists $D_0>0$ such that

$$\begin{aligned} D(x_0;x_*) = D_-(x_0;D_0) \end{aligned}$$

and

$$\begin{aligned} W(x_0;x_*)> \ (\text {resp. } <) \ W_-(x_0;D_0). \end{aligned}$$

#### Lemma 5.24

(Existence of a lower solution) There exists a $$\kappa >0$$ such that for any $$x_0<\kappa $$ there exists an $$x_{**}\in [\bar{x}_*,x_{\text {max}}]$$ such that $$(D(\cdot ;x_{**}),W(\cdot ;x_{**}))$$ is a lower solution at $$x_0$$. Moreover, there exists a universal constant *C* such that $$D_1<\frac{C}{x_0^{1-\varepsilon }}$$, where $$D_-(x_0;D_1)=D(x_0;x_{**})$$.

#### Proof

For any $x_*\in X$ we consider the function

5.367 $$\begin{aligned} S(x_*) : = \sup _{\tilde{x}_*\in [\bar{x}_*, x_*]} \left\{ x_{F}(\tilde{x}_*)\right\} . \end{aligned}$$

The function $$x_*\mapsto S(x_*)$$ is clearly increasing, continuous, and by Lemma 5.16, $$\lim _{x_*\rightarrow \bar{x}_*}S(x_*)=0$$. Therefore, the range of *S* is of the form $$[0,\kappa ]$$ for some $$\kappa >0$$. For any $$x_*\in X $$, by Lemma 5.16, the supremum in (5.367) is attained, i.e. there exists $$x_{**}\in [\bar{x}_*, x_*]$$ such that $$S(x_*) = x_{F}(x_{**}){=}{:}x_0$$. Therefore, for any $$\bar{x}_*< x_*< x_{**}$$ we have

$$\begin{aligned} s(x_*)<x_{F}(x_*)\le x_0. \end{aligned}$$

By Lemma 5.12 we have the bound $$D(x_0;x_{**})>W(x_0;x_{**})=\frac{1}{3}$$. By Lemma 5.19 choosing $$D_0 = D_0(x_0) = \frac{1}{x_0^{1-\varepsilon }} \left( \frac{3^{1-\varepsilon }}{2(1+8\varepsilon )} \right) ^{\frac{1-\varepsilon }{2}}$$ and using Lemmas 5.4 and 5.3 we have

$$\begin{aligned} D_-(x_0;D_0)>\frac{1}{x_0^{1-\varepsilon }}> \frac{J[x_0;D]}{x_0}>D(x_0;x_{**}), \end{aligned}$$

where we have assumed $$x_0$$ to be sufficiently small. On the other hand $$D_-(x_0;\frac{1}{3})=\frac{1}{3}<D(x_0;x_{**})$$ (where we recall that $$D_-(\cdot ;\frac{1}{3})$$ is the Friedmann solution, see (2.40)). Using Remark 5.21 and the Intermediate Value Theorem, there exists a $$D_1\in (\frac{1}{3},D_0)$$ such that

$$\begin{aligned} D(x_0;x_{**}) = D_-(x_0;D_1). \end{aligned}$$

By (5.342) $$W_-(x_0;D_1)>\frac{1}{3} = W(x_0;x_{**})$$ and therefore $$(D(\cdot ,x_{**}),W(\cdot ;x_{**}))$$ is a lower solution at $$x_0$$. The upper bound on $$D_1$$ follows from our choice of $$D_0$$ above. $$\square $$

#### Remark 5.25

The proof of Lemma 5.24 follows closely the analogous proof in the Newtonian case (Lemma 4.20 in [15]).

#### Lemma 5.26

Let $x_*\in \mathcal {X}_{>\frac{1}{3}}$ (see (2.44) for the definition of $\mathcal {X}_{>\frac{1}{3}}$) and assume that $s(x_*)=0$. Then

- $$\begin{aligned} D(x;x_*)>W(x;x_*), \ \ x\in (0,x_*); \end{aligned}$$
- $$\begin{aligned} \limsup _{x\rightarrow 0} x^{1-\varepsilon } W(x;x_*) >0. \end{aligned}$$

#### Proof

*Proof of Part (a).* If not let

$$\begin{aligned} x_c : = \sup _{x\in (0,x_*)} \left\{ D(\tau ;x_*)-W(\tau ;x_*)>0, \ \tau \in (x,x_*) , \ \ D(x;x_*)=W(x;x_*)\ \right\} >0. \end{aligned}$$

At $x_c$ we have from (2.35)–(2.36) $W'(x_c;x_*) = \frac{1-3W}{x_c}<0$ since $W>\frac{1}{3}$ for $x_*\in \mathcal {X}_{>\frac{1}{3}}$, and $D'(x_c;x_*)=0$. Therefore there exists a neighbourhood strictly to the left of $x_c$ such that $W'<0$, $D<W$, and $D'>0$. It is easily checked that this property is dynamically trapped and we conclude

5.368 $$\begin{aligned} W'(x;x_*) \le \frac{1-3W(x;x_*)}{x} , \ \ x\le x_c. \end{aligned}$$

Integrating the above equation over $[x,x_c]$ we conclude

5.369 $$\begin{aligned} W(x;x_*) x^3 \ge \omega (x_c;x_*) x_c^3 - \frac{1}{3} x_c^3 = \left( W(x_c;x_*)-\frac{1}{3}\right) x_c^3 {=}{:}c>0. \end{aligned}$$

We now recall (5.317). From (5.369), this implies that $\left( \log \left( D^{2(1+\eta )}W^2\right) \right) ' <0$ since $x_*\in \mathcal {X}_{>\frac{1}{3}}$. In particular we obtain the lower bound

$$\begin{aligned} D(x)^{2(1+\eta )}W(x)^2> D(x_c)^{2(1+\eta )}W(x_c)^2 = W(x_c)^{4+2\eta }>3^{-(4+2\eta )}, \ \ x\le x_c. \end{aligned}$$

It follows that

5.370 $$\begin{aligned} D^{-\eta } < 3^{\frac{\eta (2+\eta )}{1+\eta }}W^{\frac{\eta }{1+\eta }}, \ \ x\le x_c. \end{aligned}$$

On the other hand, bound (5.369) implies

5.371 $$\begin{aligned} x^2 > c^{\frac{2}{3}} W^{-\frac{2}{3}}, \ \ x\le x_c. \end{aligned}$$

From (2.37), (5.370)–(5.371) we therefore have

5.372 $$\begin{aligned} B < 3^{\frac{\eta (2+\eta )}{1+\eta }}W^{\frac{\eta }{1+\eta }} - C W^{\frac{4}{3}}, \ \ x\le x_c. \end{aligned}$$

for some universal constant $C>0$. Since *W* grows to infinity as $x\rightarrow 0$, this implies that the right-hand side above necessarily becomes negative, i.e. $s(x_*)>0$. A contradiction.

*Proof of Part (b).* Since $D>W$ by part (a) and $W>\frac{1}{3}$ (since $x_*\in \mathcal {X}_{>\frac{1}{3}}$), from Lemmas 5.3 and 5.4

5.373 $$\begin{aligned} D\le \frac{1}{x^{1-\varepsilon }}. \end{aligned}$$

By way of contradiction we assume that $\limsup _{x\rightarrow 0}x^{1-\varepsilon } W(x;x_*)=0$. For any $\tilde{\varepsilon }>0$ choose $\delta >0$ so small that

5.374 $$\begin{aligned} x^{1-\varepsilon } W(x;x_*)<\tilde{\varepsilon }, \ \ x\in (0,\delta ). \end{aligned}$$

Note that in particular, for any $x\in (0,\delta )$ we have

5.375 $$\begin{aligned} x^2 W D^{1+\eta } = x^{1-\varepsilon }W x^{1+\varepsilon }D^{1+\eta } \le \tilde{\varepsilon }, \ \ x\in (0,\delta ), \end{aligned}$$

where we have used (5.373) and (5.374). Similarly,

5.376 $$\begin{aligned} x^2 W D^{\eta } = x^{1-\varepsilon } W x^{2\varepsilon } D^\eta x^{1-\varepsilon } \le \tilde{\varepsilon } \delta ^{1-\varepsilon } , \ \ x\in (0,\delta ). \end{aligned}$$

We next claim that there exists a universal constant $\bar{C}$ such that

5.377 $$\begin{aligned} 2(1+\varepsilon )x^2(W+\varepsilon )(D-W) \le \bar{C} B. \end{aligned}$$

Keeping in mind (2.37), this is equivalent to the estimate

5.378 $$\begin{aligned}&x^2 W^2D^\eta \left( \bar{C}(1-\varepsilon )-2(1+\varepsilon )\right) + x^2WD^\eta \left( -2\varepsilon (1+\varepsilon )+2(1+\varepsilon )D+4\varepsilon \bar{C}(1+D)\right) \nonumber \\&+ x^2D^\eta \left( 2\varepsilon (1+\varepsilon )D+ \bar{C}(\varepsilon ^2-\varepsilon )\right) \le \bar{C}. \end{aligned}$$

For any $0<\bar{C}<2$ the first term on the left-hand side of (5.378) is strictly negative, and so are $-2\varepsilon (1+\varepsilon )x^2WD^\eta$ and $\bar{C}(\varepsilon ^2-\varepsilon ) x^2D^\eta$. On the other hand

5.379 $$\begin{aligned} x^2WD^\eta \left( 2(1+\varepsilon )D+4\varepsilon \bar{C}(1+D)\right)&= x^2WD^{1+\eta } \left( 2(1+\varepsilon )+4\varepsilon \bar{C}\right) +4\varepsilon \bar{Cx}^2WD^\eta \nonumber \\&\le \tilde{\varepsilon } \left( 2(1+\varepsilon )+4\varepsilon \bar{C}\right) + 4\varepsilon \bar{C} \tilde{\varepsilon } \delta ^{1-\varepsilon }, \end{aligned}$$

where we have used (5.375) and (5.376). Similarly,

5.380 $$\begin{aligned} 2\varepsilon (1+\varepsilon ) x^2D^{1+\eta } \le 2\varepsilon (1+\varepsilon ) x^{1-\varepsilon } \le 2\varepsilon (1+\varepsilon ) \delta ^{1-\varepsilon }, \end{aligned}$$

where we have used (5.373). It is thus clear that we can choose $\bar{C}$ of the form $\bar{C} = \tilde{C} (\tilde{\varepsilon } + \delta ^{1-\varepsilon })$ for some universal constant $\tilde{C}>0$ and $\tilde{\varepsilon },\delta$ sufficiently small, so that (5.377) is true. It then follows from (2.36) that

5.381 $$\begin{aligned} W' \le \frac{1-3W}{x} + \frac{\tilde{C}(\tilde{\varepsilon } +\delta ^{1-\varepsilon })W}{x} = \frac{1-(3-\tilde{C}(\tilde{\varepsilon } +\delta ^{1-\varepsilon }))W}{x}. \end{aligned}$$

As a consequence of (5.381), for sufficiently small $\tilde{\varepsilon },\delta >0$ and $x\in (0,\delta )$ we have

$$\begin{aligned} W' \le -\frac{2W}{x}, \end{aligned}$$

which in turn implies $W(x;x_*)\ge C x^{-2}$ for some $C>0$ and sufficiently small *x*, a contradiction. $\square$

#### Lemma 5.27

(Existence of an upper solution) If

$$\begin{aligned} \lim _{x\rightarrow 0} W(x;\bar{x}_*) \ne \frac{1}{3}, \end{aligned}$$

then there exists a universal constant $C>0$ and an arbitrarily small $x_0>0$ such that $(D(\cdot ;\bar{x}_*),W(\cdot ;\bar{x}_*))$ is an upper solution at $x_0$ and $D_1<\frac{C}{x_0}$, where $D_-(x_0;D_1)=D(x_0;\bar{x}_*)$.

#### Proof

It is clear that $\liminf _{x\rightarrow 0}W(x;\bar{x}_*)\ge \frac{1}{3}$ as otherwise we would have $\bar{x}_*\in X$, a contradiction to the definition (5.326) of $\bar{x}_*$ and the openness of *X*. We distinguish three cases.

*Case 1.*

$$\begin{aligned} \liminf _{x\rightarrow 0} W(x;\bar{x}_*) >\frac{1}{3}. \end{aligned}$$

In this case $\bar{x}_*\in \mathcal {X}_{>\frac{1}{3}}$ and by Proposition 5.15 we have $s(\bar{x}_*)=0$. By part (b) of Lemma 5.26 there exists a constant $C>0$ and a sequence $\{x_n\}_{n\in \mathbb N}\subset (0,1)$ such that $\lim _{n\rightarrow \infty }x_n=0$ and

5.382 $$\begin{aligned} W(x_n;\bar{x}_*)>\frac{C}{x_n^{1-\varepsilon }} \end{aligned}$$

where *C* is independent of *n*. For any such $x_n$ we have by part (a) of Lemma 5.26 and Lemma 5.4

5.383 $$\begin{aligned} D(x_n;\bar{x}_*)>W(x_n;\bar{x}_*)>\frac{C}{x_n^{1-\varepsilon }} > C D(x_n;\bar{x}_*) \end{aligned}$$

where we have used the assumption $\bar{x}_*\in \mathcal {X}_{>\frac{1}{3}}$ and part (a) of Lemma 5.26 to conclude $D(\cdot ;\bar{x}_*)>\frac{1}{3}$, which is necessary for the application of Lemma 5.4.

For any $0<x_n\ll 1$ sufficiently small consider $(D_-(\cdot ;D_{0,n}),W_-(\cdot ;D_{0,n}))$ with $D_{0,n}=D_0(x_n)= \frac{1}{x_n^{1-\varepsilon }} \left( \frac{3^{1-\varepsilon }}{2(1+8\varepsilon )} \right) ^{\frac{1-\varepsilon }{2}}>1$ for *n* large enough. By Lemmas 5.19 and 5.26

$$\begin{aligned} D_-(x_n;D_{0,n})> \frac{1}{x_n^{1-\varepsilon }}>D(x_n;\bar{x}_*)>W(x_n;\bar{x}_*)>\frac{1}{3}. \end{aligned}$$

On the other hand,

$$\begin{aligned} D(x_n;\bar{x}_*)>\frac{1}{3} = D_-(x_n;\frac{1}{3}), \end{aligned}$$

where we recall that $D_-(\cdot ;\frac{1}{3})\equiv \frac{1}{3}$ is the Friedmann solution. Moreover, by Remark 5.21$[0,x_n]\subset [0,s_-(\tilde{D}_0))$ for all $\tilde{D}_0\subset [\frac{1}{3},D_{0,n}]$. By the continuity of the map $[\frac{1}{3},D_{0,n}]\ni \tilde{D}_0\mapsto D_-(x_n;\tilde{D}_0)$ the Intermediate Value Theorem implies that there exists $D_{n,1}\in (\frac{1}{3},D_{0,n})$ such that

5.384 $$\begin{aligned} D_-(x_n;D_1^n) = D(x_n;\bar{x}_*) \ \ \text { for all sufficiently large }\quad n\in \mathbb N. \end{aligned}$$

Let

$$\begin{aligned} c_{1,n}: = {\left\{ \begin{array}{ll} \exp \left( - \left( D_{n,1}\right) ^{-1+\varepsilon }\right) , &{} \text { if }\ D_{n,1}> 1; \\ \exp \left( -1 \right) , &{} \text { if }\ \frac{1}{3} <D_{n,1} \le 1. \end{array}\right. } \end{aligned}$$

Clearly $c_{1,n}\ge e^{-1}{=}{:}c_1$ for all $n\in \mathbb N$. By Lemma 5.19$D_-(x_n;D_{n,1})\ge c_1D_{n,1}$. Using (5.344)–(5.345) we also have $W_-(x_n;D_{n,1})< D_{n,1}^{\frac{1+\varepsilon }{2}} 3^{-\frac{1-\varepsilon }{2}}$. Together with (5.383) and (5.384) we conclude that for all *n* sufficiently large

5.385 $$\begin{aligned} W_-(x_n;D_{n,1})&<D_{n,1}^{\frac{1+\varepsilon }{2}} 3^{-\frac{1-\varepsilon }{2}}<\left( \frac{D_-(x_n;D_{n,1})}{c_1}\right) ^{\frac{1+\varepsilon }{2}}3^{-\frac{1-\varepsilon }{2}} = \left( \frac{D(x_n;\bar{x}_*)}{c_1}\right) ^{\frac{1+\varepsilon }{2}}3^{-\frac{1-\varepsilon }{2}} \nonumber \\&< 3^{-\frac{1-\varepsilon }{2}} (c_1 C)^{-\frac{1+\varepsilon }{2}} W(x_n;\bar{x}_*)^{\frac{1+\varepsilon }{2}}. \end{aligned}$$

By (5.383) $W(x_n;\bar{x}_*)$ grows to positive infinity as $x_n$ approaches zero. Therefore, we may choose a sufficiently large $N\in \mathbb N$ and set $x_0=x_N\ll 1$, $D_0=D_{0,N}$, $D_1=D_{1,N}$ so that the right-hand side of (5.385) is bounded from above by $W(x_0;\bar{x}_*)$. This gives

$$\begin{aligned} W_-(x_0;D_1) <W(x_0;\bar{x}_*). \end{aligned}$$

We conclude that $(D(\cdot ;\bar{x}_*), W(\cdot ;\bar{x}_*))$ is an upper solution (see Definition 5.23) at $x_0$ and the upper bound on $D_1$ follows from our choice of $D_0$.

*Case 2.*

5.386 $$\begin{aligned} \frac{1}{3}<\limsup _{x\rightarrow 0} W(x;\bar{x}_*) <\infty , \ \ \liminf _{x\rightarrow 0}W(x;\bar{x}_*)=\frac{1}{3}. \end{aligned}$$

In particular $\bar{x}_*\in \mathcal {X}_{<\frac{1}{3}}$ (see (2.46)) and by Lemma 5.12$D(x;\bar{x}_*)>W(x;\bar{x}_*)$. Assumption (5.386) also implies that there exists a constant $c>0$ independent of *x* such that

5.387 $$\begin{aligned} W(x;\bar{x}_*)<c, \ \ x\in (0,\bar{x}_*]. \end{aligned}$$

We now claim that there exists a constant *C* such that

5.388 $$\begin{aligned} D'\ge - \frac{C D}{x^\varepsilon }, \end{aligned}$$

for all sufficiently small *x*. To prove (5.388) we note that by (2.35) this is equivalent to the bound $x^{1+\varepsilon }(W+\varepsilon )(D-W)\le C B,$ which, by (2.37) is equivalent to the bound

5.389 $$\begin{aligned}&x^2W^2D^\eta \left( -\frac{1}{x^{1-\varepsilon }}+C(1-\varepsilon )\right) + x^2WD^{1+\eta } \left( \frac{1}{x^{1-\varepsilon }}+4\varepsilon C)\right) \nonumber \\&\quad + x^2 W D^\eta \left( -\frac{\varepsilon }{x^{1-\varepsilon }}+4\varepsilon C\right) + x^2 D^\eta \left( \frac{\varepsilon }{x^{1-\varepsilon }}+C(\varepsilon ^2-\varepsilon )\right) \le C. \end{aligned}$$

Using (5.387) and Lemma 5.4, we have

$$\begin{aligned} x^2W^2D^\eta \le c^2 x^{2-2\varepsilon }, \ \ x^2WD^{1+\eta } \le c x^{1-\varepsilon }, \ \ x^2WD^\eta \le c x^{2-2\varepsilon }, \ \ x^2D^\eta \le x^{2-2\varepsilon }. \end{aligned}$$

Choosing $\varepsilon >0$ sufficiently small, *x* so small that $-\frac{1}{x^{1-\varepsilon }}+(1-\varepsilon )<0$, and $C>1$ sufficiently large, but independent of $\varepsilon$, we use the above bounds to conclude the claim.

Bound (5.388) gives $\left( \log D+ \frac{C}{1-\varepsilon }x^{1-\varepsilon }\right) '\ge 0$, which in turn gives the bound

5.390 $$\begin{aligned} D(x) \le D(\bar{x}) e^{ \frac{C}{1-\varepsilon }\bar{x}^{1-\varepsilon } - \frac{C}{1-\varepsilon } x^{1-\varepsilon }}, \ \ x\in (0,\bar{x}], \end{aligned}$$

for some $\bar{x}$ sufficiently small. This immediately implies the uniform boundedness of $D(\cdot ;\bar{x}_*)$, i.e.

5.391 $$\begin{aligned} D(x;\bar{x}_*)<c, \ \ x\in (0,x_*], \end{aligned}$$

where we have (possibly) enlarged *c* so that (5.387) and (5.391) are both true. There exists an $\delta >0$ and a sequence $\left\{ x_n\right\} _{n\in \mathbb N}$ such that $\lim _{n\rightarrow \infty }x_n=0$ and

$$\begin{aligned} \frac{1}{3} + \delta < W(x_n;\bar{x}_*), \ \ \text { and } \ \ \lim _{n\rightarrow \infty } W(x_n;\bar{x}_*) = \limsup _{x\rightarrow 0} W(x;\bar{x}_*). \end{aligned}$$

Since $\{ D(x_n;\bar{x}_*)\}_{n\in \mathbb N}$ is bounded, by Lemma 5.19 we can choose an $D_0>1$ such that $D_-(x_n;D_0) > D(x_n;\bar{x}_*)$ for all $n\in \mathbb N$. On the other hand $D(x_n;\bar{x}_*)>\frac{1}{3}=D_-(x_n;\frac{1}{3})$. By the intermediate value theorem there exists a sequence $\{D_{0,n}\}_{n\in \mathbb N}\subset (\frac{1}{3},D_0)$ such that

$$\begin{aligned} D_-(x_n;D_{0,n}) = D(x_n;\bar{x}_*). \end{aligned}$$

Since $W_-(x;D_{0,n})^2\le \frac{D_{0,n}^{1+\varepsilon }}{3^{1-\varepsilon }}<\frac{D_0^{1+\varepsilon }}{3^{1-\varepsilon }}$ and $D_-(x;D_{0,n})<D_{0,n}<D_0$ (Lemma 5.18) we conclude from (2.35)–(2.36) and Theorem 4.24 that $\left| D_-'(x_n;D_{0,n}) \right|$ and $\left| W_-'(x_n;D_{0,n})\right|$ are bounded uniformly-in-*n*, by some constant $\tilde{C}>0$. Therefore

$$\begin{aligned} W_-(x_n;D_{0,n}) \le \frac{1}{3} + \tilde{C} x_n. \end{aligned}$$

We thus conclude that for a fixed *n* sufficiently large $W_-(x_n;D_{0,n})\le \frac{1}{3} + \delta <W(x_n;\bar{x}_*)$. Therefore, $W(\cdot ;\bar{x}_*)$ is an upper solution (see Definition 5.23) at $x_0{:}{=}x_n$ with $D_1=D_{0,n}$. The claimed upper bound on $D_1$ is clear.

*Case 3.*

$$\begin{aligned} \frac{1}{3}<\limsup _{x\rightarrow 0} W(x;\bar{x}_*) =\infty , \ \ \liminf _{x\rightarrow 0}W(x;\bar{x}_*)=\frac{1}{3}. \end{aligned}$$

As $W(\cdot ;\bar{x}_*)$ must oscillate between $\frac{1}{3}$ and $\infty$ we can use the mean value theorem to conclude that there exists a sequence $\left\{ x_n\right\} _{n\in \mathbb N}$ such that $\lim _{n\rightarrow \infty }x_n=0$ and

5.392 $$\begin{aligned} W(x_n;\bar{x}_*)>n, \ \ \text { and } \ \ W'(x_n;\bar{x}_*)=0. \end{aligned}$$

We claim that there exist $N_0>0$ and $0<\eta \ll 1$ such that

5.393 $$\begin{aligned} W(x_n;\bar{x}_*)\ge \frac{1}{2x_n^{1-\varepsilon }}, \ \ n\ge N_0. \end{aligned}$$

To prove this, assume that (5.393) is not true. Then there exists a subsequence of $\{x_n\}_{n\in \mathbb N}$ such that $W(x_n;\bar{x}_*)< \frac{1}{2x_n^{1-\varepsilon }}$. We now rewrite (2.36) in the form

5.394 $$\begin{aligned} W' = \frac{W}{x}\left( \frac{1}{W} - (3-\gamma ) +\frac{-\gamma B + 2(1+\varepsilon )x^2(W+\varepsilon )(D-W)}{B}\right) , \end{aligned}$$

where $\gamma \in (0,3)$ is a control parameter to be chosen below. We use (2.37) to evaluate

5.395 $$\begin{aligned}&-\gamma B + 2x^2(1+\varepsilon )(W+\varepsilon )(D-W) \nonumber \\&\quad =x^2\left[ \gamma \left( ( W + \varepsilon )^2 - \varepsilon (W - 1)^2 + 4\varepsilon DW\right) - 2(1+\varepsilon )(W+\varepsilon )(W-D)\right] -\gamma D^{-\eta }\nonumber \\&\quad = x^2\Big \{ \left[ \gamma (1-\varepsilon )-2(1+\varepsilon )\right] W^2 + \left[ 4\gamma \varepsilon - 2(1+\varepsilon )\varepsilon +\left( 4\gamma \varepsilon +2(1+\varepsilon )\right) D\right] W \nonumber \\&\ \ \ \ \qquad + \gamma (\varepsilon ^2-\varepsilon ) + 2 \varepsilon (1+\varepsilon )D\Big \} - \gamma D^{-\eta }. \end{aligned}$$

Since $D\ge \frac{1}{3}$, by Lemma 5.4 we also have the bound $D(x)\le \frac{1}{x^{1-\varepsilon }}$. We may therefore estimate

5.396 $$\begin{aligned}&D^\eta \left( -\gamma B + 2x^2(1+\varepsilon )(W+\varepsilon )(D-W)\right) \nonumber \\&\quad \le x^2 D^\eta \left\{ \left[ \gamma (1-\varepsilon )-2(1+\varepsilon )\right] W^2 + 4\gamma \varepsilon W\right\} + \left[ \left( 4\gamma \varepsilon +2(1+\varepsilon )\right) \right] x^2WD^{1+\eta } \nonumber \\&\qquad + 2 \varepsilon (1+\varepsilon )x^2D^{1+\eta } - \gamma . \end{aligned}$$

By (5.392), for any $\gamma \in (0,\frac{3}{2})$ we have $\left[ \gamma (1-\varepsilon )-2(1+\varepsilon )\right] W(x_n;\bar{x}_*)^2 + 4\gamma \varepsilon W(x_n;\bar{x}_*)<0$ for all sufficiently large *n*. We use the upper bounds on $(D,W)$ along the sequence $\{x_n\}_{n\in \mathbb N}$ to obtain

$$\begin{aligned} x^2WD^{1+\eta } \le \frac{1}{2} \ \text { and } \ x^2 D^{1+\eta } \le x^{1-\varepsilon }, \ \ \end{aligned}$$

and therefore

5.397 $$\begin{aligned}&D^\eta \left( -\gamma B + 2x^2(1+\varepsilon )(W+\varepsilon )(D-W)\right) \Big |_{x=x_n} \le 2\gamma \varepsilon + 1 + \varepsilon + 2\varepsilon (1+\varepsilon )x_n^{1-\varepsilon }-\gamma <0 \nonumber \\ \end{aligned}$$

for some $\gamma \in (1,\frac{3}{2})$ and all sufficiently small $\varepsilon$. Feeding this back into (5.394), we conclude

5.398 $$\begin{aligned} W'(x_n;\bar{x}_*)&\le \frac{W(x_n;\bar{x}_*)}{x_n} \left( \frac{1}{W(x_n;\bar{x}_*)} - (3-\gamma )\right) <0, \end{aligned}$$

for all sufficiently large *n*, where we use the first bound in (5.392), which contradicts the second claim in (5.392). We can therefore repeat the same argument following (5.382) to conclude that $W(\cdot ;\bar{x}_*)$ is an upper solution at $x_0{:}{=}x_n$, for some *n* sufficiently large. The upper bound on $D_1$ follows in the same way. $\square$

We next show that $W(\cdot ;\bar{x}_*)$ takes on value $\frac{1}{3}$ at $x=0$.

#### Proposition 5.28

The limit $\lim _{x\rightarrow 0}W(x;\bar{x}_*)$ exists and

$$\begin{aligned} \lim _{x\rightarrow 0}W(x;\bar{x}_*) = \frac{1}{3}. \end{aligned}$$

#### Proof

Assume that the claim is not true. By Lemmas 5.24 and 5.27 we can find a $$0<x_0\ll 1$$ and $$x_{**}\in X $$ so that $$(D(\cdot ;x_{**}),W(\cdot ;x_{**}))$$ and $$(D(\cdot ;\bar{x}_*),W(\cdot ;\bar{x}_*))$$ are respectively a lower and an upper solution at $$x_0$$. Without loss of generality let

$$\begin{aligned} A: =D(x_0;x_{**})< D(x_0;\bar{x}_*){=}{:}B. \end{aligned}$$

By Lemmas 5.24 and 5.27 there exist $D_A,D_B>\frac{1}{3}$ such that $A=D_-(x_0;D_A)$, $B=D_-(x_0;D_B)$, and $D_A,D_B\in (\frac{1}{3},D_0)$, where $D_0\gg 1$ and $x_0 \le C\frac{1}{D_0}$. Therefore by Lemma 5.22, $\partial _{D_0}D_-(x_0;\tilde{D}_0)>0$ for all $\tilde{D}_0\in [\frac{1}{3},D_0]$, since $D_0^{- \tfrac{3+\varepsilon +2\eta }{4} }\gg D_0^{-1}$ for $D_0$ large and all $\varepsilon \le \varepsilon _0$ with $\varepsilon _0$ sufficiently small. By the inverse function theorem, there exists a continuous function $\tau \mapsto g(\tau )$ such that

$$\begin{aligned} D_-(x_0;g(\tau ))&= \tau , \ \ \tau \in [A,B], \\ g(D_A)&= A. \end{aligned}$$

By strict monotonicity of $\tilde{D}_0\mapsto D_-(x_0;\tilde{D}_0)$ on $(0, D_0]$ the inverse *g* is in fact injective and therefore $g( D_B)=B$. We consider the map

$$\begin{aligned}{}[\bar{x}_*,x_{**}] \ni x_*\mapsto W(x_0;x_*) - W_-(x_0;g( D(x_0;x_*))) = : h(x_*). \end{aligned}$$

By the above discussion *h* is continuous, $$h(A)<0$$ and $$h(B)>0$$. Therefore, by the Intermediate Value Theorem there exists an $$x_s\in (\bar{x}_*,x_{**})$$ such that $$h(x_s)=0$$. The solution $$( D(\cdot ;x_s),W(\cdot ;x_s))$$ exists on $$[0,x_s]$$, satisfies $$W(0)=\frac{1}{3}$$ and belongs to *X*. This is a contradiction to the definition of *X*. $$\square $$

In the next proposition we will prove that the solution $(D(\cdot ;x_*), W(\cdot ;x_*))$ is analytic in a left neighbourhood of $x=0$, by showing that it coincides with a solution emanating from the origin.

#### Proposition 5.29

There exists a constant $C_*>0$ so that

$$\begin{aligned} \left| D(x;\bar{x}_*)\right| + \left| W(x;\bar{x}_*)\right| + \left| \frac{W(x;\bar{x}_*)-\frac{1}{3}}{x^2}\right| \le C_*, \ \ x\in (0,\bar{x}_*]. \end{aligned}$$

The solution $D(\cdot ;\bar{x}_*):(0,\bar{x}_*]\rightarrow \mathbb R_{>0}$ extends continuously to $x=0$ and $D_*{:}{=}D(0;\bar{x}_*)<\infty$. Moreover, the solution $(D(\cdot ;\bar{x}_*), W(\cdot ;\bar{x}_*))$ coincides with $(D_-(\cdot ;D_*), W_-(\cdot ;D_*))$ and it is therefore analytic at $x=0$ by Theorem 4.24.

#### Proof

By Proposition 5.28 it is clear that $W(\cdot ;\bar{x}_*)$ is bounded on $[0,\bar{x}_*]$. Moreover, $D\ge W\ge \frac{1}{3}$ on $[0,\bar{x}_*]$ and therefore by Lemma 5.4$D\le \frac{1}{x^{1-\varepsilon }}$. We may now apply the exact same argument as in the proof of (5.391) to conclude that $D$ is uniformly bounded on $[0,\bar{x}_*]$. Since $B>0$ and $D>W$ on $[0,\bar{x}_*]$, by (2.35) $D'\le 0$ and therefore the limit $D_*{:}{=}\lim _{x\rightarrow 0}D(x;\bar{x}_*)$ exists and it is finite. It is also clear that there exists a constant $c_0>0$ such that

5.399 $$\begin{aligned} B(x)>c_0, \ \ x\in [0,\bar{x}_*-\tilde{r}]. \end{aligned}$$

Let

$$\begin{aligned} \zeta = W-\frac{1}{3}\ge 0. \end{aligned}$$

It is then easy to check from (2.36)

$$\begin{aligned} \left( \zeta x^3\right) ' = x^3 \frac{2(1+\varepsilon ) x W(W+\varepsilon )(D- W)}{B} \end{aligned}$$

and therefore, by (5.399) and the uniform boundedness of *W* and $D$, for any $0<x_1<x$ we obtain

$$\begin{aligned} \zeta (x) x^3 - \zeta (x_1) x_1^3 \le C \int _{x_1}^{x}\tau ^4\,d\tau = \frac{C}{5}\left( x^5-x_1^5\right) , \ \ x\in (0,\bar{x}_*-\tilde{r}]. \end{aligned}$$

We now let $x_1\rightarrow 0^+$ and conclude

5.400 $$\begin{aligned} \zeta (x)\lesssim x^2. \end{aligned}$$

Consider now $\bar{D}(x){:}{=}D(x;\bar{x}_*)-D_-(x;D_*)$ and $\bar{W}(x){:}{=} W(x;\bar{x}_*)- W_-(x;D_*)$, both are defined in a (right) neighbourhood of $x=0$ and satisfy

$$\begin{aligned} \bar{D}'&= O(1)\bar{D} + O(1)\bar{W}, \\ \bar{W}'&= - 3\frac{\bar{W}}{x} + O(1)\bar{D} + O(1)\bar{W}, \end{aligned}$$

where $\bar{D}(0)=\bar{W}(0)=0$. Here we have used the already proven boundedness of $(D(\cdot ;\bar{x}_*), W(\cdot ;\bar{x}_*))$ and the boundedness of $(D_-(\cdot ;D_*), W_-(\cdot ;D_*))$, see Lemma 5.18. We multiply the first equation by $\bar{D}$, the second by $\bar{W}$, integrate over [0, *x*] and use Cauchy-Schwarz to get

5.401 $$\begin{aligned} \bar{D}(x)^2+\bar{W}(x)^2 + 3\int _0^x\frac{\bar{W}(\tau )^2}{\tau }\,d\tau \le C \int _0^x \left( \bar{D}(\tau )^2+\bar{W}(\tau )^2\right) \,d\tau . \end{aligned}$$

We note that $$\int _0^x\frac{\bar{W}(\tau )^2}{\tau }\,d\tau $$ is well-defined, since $$\bar{W} = \zeta - \zeta _-$$, where $$\zeta _-= W_--\frac{1}{3}$$; we use (5.400) and observe $$\zeta _-\lesssim x^2$$ in the vicinity of $$x=0$$ by the analyticity of $$ W_-$$, see Theorem 4.24. Therefore $$\bar{D}(x)^2+\bar{W}(x)^2=0$$ by (5.401). The analyticity claim now follows from Theorem 4.24. $$\square $$

#### Remark 5.30

Propositions 5.28 and 5.29 follow closely the arguments in the Newtonian case (Propositions 4.22 and 4.23 in [15]). However, an important difference is the use of (5.391) in the proof of Proposition 5.29.

*Proof of Theorem* 2.3. Theorem 2.3 is now a simple corollary of Propositions 5.15, 5.28, and 5.29. $\square$

## The Far Field Connection

The goal of this section is to construct a global-to-the-right solution of the dynamical system (2.35)–(2.36) for all $x_*$ in the sonic window $[x_{\text {min}},x_{\text {max}}]$. We refer to Section 2.4 for a detailed overview.

### A Priori Bounds

#### Remark 6.1

The condition $J>xD$ is equivalent to the inequality

6.402 $$\begin{aligned} D^{-\eta } > x^2\left( (1-\varepsilon )D^2-\varepsilon (1-\varepsilon )+4\varepsilon D(1+D)\right) , \end{aligned}$$

which can be seen directly from the definition (3.124) of $J[x;D]$. Similarly, the inequality $xW>J$ is equivalent to

6.403 $$\begin{aligned} D^{-\eta } < x^2\left( (1-\varepsilon )W^2- \varepsilon (1-\varepsilon ) +4\varepsilon W(1+D) \right) . \end{aligned}$$

#### Lemma 6.2

(A priori bounds to the right) Let $x_*\in [x_{\text {min}},x_{\text {max}}]$. Let $X>x_*$ be the maximal time of existence to the right of the associated RLP-type solution on which we have

6.404 $$\begin{aligned} xW(x)&>J[x;D], \ \ x\in (x_*+\delta ,X), \end{aligned}$$

6.405 $$\begin{aligned} J[x;D]&>x D(x), \ \ x\in (x_*+\delta ,X), \end{aligned}$$

where $0<\delta \ll 1$ is an $\varepsilon$-independent constant from Lemma 4.23. Then the following claims hold

*(a)*: 6.406 $$\begin{aligned} D'&<0, \ \ x\in (x_*+\delta ,X) , \end{aligned}$$
  6.407 $$\begin{aligned} W&>\frac{1}{6}, \ \ x\in (x_*+\delta ,X) , \end{aligned}$$
  6.408 $$\begin{aligned} D(x)W(x)&> D_\delta ^2 \left( \frac{x_*+\delta }{x}\right) ^3, \ \ x\in (x_*+\delta ,X), \end{aligned}$$
  where
  $$\begin{aligned} D_\delta {:}{=}D(x_*+\delta ). \end{aligned}$$
*(b)*: There exists a constant $\tilde{C}$ *independent* of *X* and $\varepsilon _0>0$ such that for all $0<\varepsilon \le \varepsilon _0$
  6.409 $$\begin{aligned} W(x)\le \tilde{C}, \ \ x\in (x_*+\delta ,X). \end{aligned}$$
(c): There exist constants $C>0$, $0<\varepsilon _0\ll 1$ such that for all $\varepsilon \in (0,\varepsilon _0)$
  6.410 $$\begin{aligned} \frac{D'x}{D} \le - \gamma , \ \ x\in (x_*+\delta ,X), \ \ \gamma {:}{=} 1-C\varepsilon >0 \end{aligned}$$
  and therefore
  6.411 $$\begin{aligned} D(x) \le D_\delta \left( \frac{x}{x_*+\delta }\right) ^{-\gamma }. \end{aligned}$$
  In particular, with $0<\varepsilon _0\ll 1$ sufficiently small, we may choose $\gamma = 1- C\varepsilon _0$ uniform for all $\varepsilon \in (0,\varepsilon _0)$.

#### Proof

We note that by Lemma 4.23 there exists a $\delta >0$ such that $X>x_*+2\delta$.

*Proof of part (a).* Notice that by assumptions (6.404)–(6.405) we have

6.412 $$\begin{aligned} W&>D, \ \ x\in (x_*+\delta ,X). \end{aligned}$$

Bound (6.406) follows from (2.35), (3.123), (6.404), and (6.412).

By (2.36) and (6.404)–(6.405) we conclude $W'\ge \frac{1-3W}{x}$ or equivalently $(Wx^{3})'\ge x^{2}$. We conclude that for any $x\in (x_*+\delta ,X)$ we have

6.413 $$\begin{aligned} W(x) \ge W_\delta \left( \frac{x_*+\delta }{x}\right) ^{3} + \frac{1}{3}\left( 1-\left( \frac{x_*+\delta }{x}\right) ^{3}\right) \ge \frac{1}{6}, \end{aligned}$$

since $W_\delta {:}{=}W(x_*+\delta ) =W_0(\varepsilon ,x_*)-O(\delta )\ge W_0(\varepsilon ;x_{\text {max}}(\varepsilon ))-O(\delta )=\frac{1}{3}-O(\delta )$, for $0<\delta \ll 1$ sufficiently small and independent of $\varepsilon$. Here we have also used Remark 4.2 and the definition of $x_{\text {max}}$ in (4.245).

From (2.35)–(2.36) we have

6.414 $$\begin{aligned} x^{1-\beta }\left( WDx^{\beta }\right) '&= D' W x + DW' x + \beta DW \nonumber \\&= D+ (\beta -3) DW + \frac{4\varepsilon x^2 DW (W + \varepsilon ) (D-W)}{B}. \end{aligned}$$

We may let $\beta =3$ and conclude from (6.404), (6.412) that $\frac{d}{dx}\left( WDx^{\beta }\right) >0$ on $(x_*+\delta ,X)$. In particular

6.415 $$\begin{aligned} D(x) W(x) > D_\delta ^2 \left( \frac{x_*+\delta }{x}\right) ^3, \ \ x\in (x_*+\delta ,X), \end{aligned}$$

which is (6.408).

*Proof of part (b).* Let now $\gamma \in (0,3)$ and rewrite (2.36) in the following way:

6.416 $$\begin{aligned} W' = \frac{1-(3-\gamma )W}{x} + \frac{W}{xB}\left( -\gamma B + 2x^2(1+\varepsilon )(W+\varepsilon )(D-W)\right) . \end{aligned}$$

We use (2.37) to evaluate

6.417 $$\begin{aligned}&-\gamma B + 2x^2(1+\varepsilon )(W+\varepsilon )(D-W) \nonumber \\&=x^2\left[ \gamma \left( ( W + \varepsilon )^2 - \varepsilon (W - 1)^2 + 4\varepsilon DW\right) - 2(1+\varepsilon )(W+\varepsilon )(W-D)\right] -\gamma D^{-\eta }\nonumber \\&= x^2\Big \{ \left[ \gamma (1-\varepsilon )-2(1+\varepsilon )\right] W^2 + \left[ 4\gamma \varepsilon - 2(1+\varepsilon )\varepsilon +\left( 4\gamma \varepsilon +2(1+\varepsilon )\right) D\right] W \nonumber \\&\ \ \ \ \qquad + \gamma (\varepsilon ^2-\varepsilon ) + 2 \varepsilon (1+\varepsilon )D\Big \} - \gamma D^{-\eta } \nonumber \\&{=}{:} G_\gamma [x;D,W]. \end{aligned}$$

By (6.408) we know that for any $x\in (x_*+\delta ,X)$, $D(x) > D_\delta ^2 (x_*+\delta )^3 x^{-3}W(x)^{-1},$ and therefore

6.418 $$\begin{aligned} D^{-\eta } <D_\delta ^{-2\eta } (x_*+\delta )^{-3\eta } x^{3\eta }W(x)^{\eta }, \ \ x\in (x_*+\delta , X). \end{aligned}$$

Fix now any $\gamma \in (\frac{2(1+\varepsilon )}{1-\varepsilon },3)$, which is possible since $\varepsilon$ can be chosen small. The expression $G_\gamma [x;D,W]$ in (6.417) can be bounded from below with the help of (6.418) by

6.419 $$\begin{aligned} g[x,W]: = x^2(1-\varepsilon )\left( \gamma - \frac{2(1+\varepsilon )}{1-\varepsilon } \right) W^2 + x^2\chi _1[D] W + x^2\chi _2[D] - C_0(x_*) x^{3\eta }W(x)^{\eta }, \end{aligned}$$

where

$$\begin{aligned} \chi _1[D]&: = 4\gamma \varepsilon - 2(1+\varepsilon )\varepsilon +\left( 4\gamma \varepsilon +2(1+\varepsilon )\right) D, \\ \chi _2[D]&: = \gamma (\varepsilon ^2-\varepsilon ) + 2 \varepsilon (1+\varepsilon )D, \\ C_0(x_*)&: = \gamma D_\delta ^{-2\eta } (x_*+\delta )^{-3\eta } . \end{aligned}$$

By the already proven bound (6.406) it follows that $D(x)\le D_\delta$. Therefore, from the above formulas, there exists a universal constant *C* such that

$$\begin{aligned} |\chi _1[D]| + |\chi _2[D]| + |C_0(x_*)| \le C, \ \ x\in (x_*+\delta , X), \ \ x_*\in [x_{\text {min}},x_{\text {max}}]. \end{aligned}$$

Since $x>x_*+\delta \ge 1$ and for sufficiently small $\varepsilon$ we have $3\eta <2$ (recall (2.38)), it follows that there exists a constant $\bar{C}=\bar{C}(\gamma )$ *independent* of *X* and $\varepsilon$ such that

6.420 $$\begin{aligned} g[x,W]>1, \ \ \text { if } \ W(x)>\bar{C}. \end{aligned}$$

Let now $\tilde{C} = \max \{\bar{C}, \frac{2}{3-\gamma }\}$. It then follows that both summands on the right-hand side of (6.416) are strictly negative when $W\ge \tilde{C}$; the first term is negative due to $1-(3-\gamma ) C<0$ and the second one due to (6.420) and the negativity of *B*. Therefore the region $W\ge \tilde{C}$ is dynamically inaccessible. This completes the proof of (6.409).

*Proof of part (c).* For any $\gamma \in \mathbb R$ it follows from (2.35) that

6.421 $$\begin{aligned} \frac{D'x}{D} + \gamma = \frac{\gamma B - 2(1-\varepsilon )x^2(W+\varepsilon )(D-W)}{B}. \end{aligned}$$

We focus on the numerator of the right-hand side above. By (2.37) we have

6.422 $$\begin{aligned}&\gamma B - 2(1-\varepsilon )x^2(W+\varepsilon )(D-W) \nonumber \\&= \gamma D^{-\eta } -\gamma \left[ ( W + \varepsilon )^2 - \varepsilon (W - 1)^2 + 4\varepsilon DW \right] x^2- 2(1-\varepsilon )x^2(W+\varepsilon )(D-W) \nonumber \\&\ge \gamma x^2\left( (1-\varepsilon )D^2-\varepsilon (1-\varepsilon )+4\varepsilon D(1+D)\right) \nonumber \\&\ \ \ \ -\gamma \left[ ( W + \varepsilon )^2 - \varepsilon (W - 1)^2 + 4\varepsilon DW \right] x^2- 2(1-\varepsilon )x^2(W+\varepsilon )(D-W) \nonumber \\&= x^2 (W-D) \left[ \gamma (W-D) + (2-2\gamma ) W - \varepsilon \left( 3\gamma D+ W(2-\gamma ) + 4\gamma +2\varepsilon -2\right) \right] . \end{aligned}$$

In the third line above we crucially used the bound (6.402), which by Remark 6.1 is equivalent to the assumption (6.405). Since $W>D$, $\tilde{C}>W\ge \frac{1}{6}$, and $0<D<D_\delta$ on $(x_*+\delta ,X)$, we may choose $\gamma =1-C\varepsilon$ with *C* sufficiently large, but $\varepsilon$-independent, so that the above expression is strictly positive, since in this case $(2-2\gamma ) W\ge \frac{C\varepsilon }{3}$. Therefore, for sufficiently small $\varepsilon$ and $\gamma =1-C\varepsilon$, the right-hand side of (6.421) is negative, since $B<0$ on $(x_*,X)$. This proves (6.410), which after an integration gives (6.411). $\square$

### Monotonicity and Global Existence

#### Lemma 6.3

(Monotonicity properties of the flow) Let $x_*\in [x_{\text {min}},x_{\text {max}}]$. Let $X>x_*$ be the maximal time of existence to the right of the associated RLP-type solution on which we have

6.423 $$\begin{aligned} xW(x)&>J[x;D], \ \ x\in (x_*+\delta ,X). \end{aligned}$$

Then there exists an $0<\varepsilon _0\ll 1$ such that for all $\varepsilon \in (0,\varepsilon _0]$ the associated RLP-type solution satisfies,

6.424 $$\begin{aligned} J[x;D]>x D(x), \ \ x\in (x_*+\delta ,X). \end{aligned}$$

If in addition $X<\infty$, then there exists a constant $\bar{\kappa } = \bar{\kappa }(X)>0$ such that $f(x)=J[x;D]-xD(x)>\bar{\kappa }$ for all $x\in (x_*+\delta , X)$.

#### Proof

We observe that $b_1[x;D,W]$ is strictly positive on $(x_*+\delta ,X)$ by our assumption. Moreover, from the definition of (3.134), it is clear that $b_2[x;D,W]\ge \tilde{b}_2[x;D,W]$, where

6.425 $$\begin{aligned} \tilde{b}_2[x;D,W] =&- 2 x^2D\Big \{D^2+(3+\varepsilon )D-(1-\varepsilon ) \nonumber \\&\ \ \ \ +\varepsilon \left[ \frac{4}{1-\varepsilon }D^2+\frac{2(1+\varepsilon )}{1-\varepsilon }(1+D) + \frac{5-\varepsilon }{1-\varepsilon }D+3\right] \Big \}Z^{-1}; \end{aligned}$$

It is clear that for any $D\le \frac{1}{4}$ and $\varepsilon _0$ sufficiently small the expression $\tilde{b}_2[x;D,W]$ is strictly positive for all $0<\varepsilon \le \varepsilon _0$. If there exists an $\tilde{x}>x_*+\delta$ such that $D(\tilde{x})\le \frac{1}{4}$, then by the monotonicity of $x\mapsto D(x)$, $D(x)\le \frac{1}{4}$ for all $x\ge \tilde{x}$. Thus $\tilde{b}_2[x;D,W]$ and therefore $b[x;D,W]$ remain positive. It follows that if $f(\tilde{x})>0$, then by Corollary 3.8$f(x)>0$ for all $x\in [\tilde{x}, X)$.

We next show that for a sufficiently small $\varepsilon _0$ the value of $D$ always drops below $\frac{1}{4}$ at some $\tilde{x}$ for all $\varepsilon \in (0,\varepsilon _0]$ so that *f* remains strictly positive on $(x_*+\delta ,\tilde{x}]$. By the bound (6.411) and $D_\delta \le 1$ it follows that $D(x)\le \frac{1}{4}$ as long as $x\ge x_*4^{\frac{1}{\gamma }}$. Since $\gamma = 1+O(\varepsilon )$ and $x_*\le 3$, then with

6.426 $$\begin{aligned} \tilde{x} = 3 \times 4^{\frac{1}{1-\frac{1}{2}}} = 48 \end{aligned}$$

it follows that if $X>\tilde{x}$ and $f(x)>0$ on $(x_*+\delta , \tilde{x}]$, then $f(x)>0$ for all $x\in (x_*+\delta , X)$, with $\varepsilon _0$ chosen sufficiently small.

It remains to show that for all $\varepsilon \in (0,\varepsilon _0]$ with $\varepsilon _0$ chosen sufficiently small, we indeed have $f>0$ on $(x_*+\delta , \min \{\tilde{x},X\})$. Let $(x_*+\delta , X_1)\subset (x_*+\delta , X)$ be the maximal subinterval of $(x_*+\delta , X)$, such that $f>0$ on $(x_*+\delta , X_1)$. By Lemma 4.23 we have $X_1\ge x_*+2\delta$. Notice that on the interval $(x_*+\delta ,X_1)$, by the strict negativity of *B* the factor $a_1[x;D,W]$ (3.131) is strictly negative. Since $b_1$ is positive and $b_2\ge \tilde{b}_2$ we have from Lemma 3.7

$$\begin{aligned} f' + a_2 f\ge \varepsilon \tilde{b}_2. \end{aligned}$$

For any $x_*+\delta<x_1<x<X_1$ this immediately yields

6.427 $$\begin{aligned} f(x) \ge f(x_1) e^{-\int _{x_1}^x a_2[z;D,W]\,dz} + \varepsilon e^{-\int _{x_1}^x a_2[z;D,W]\,dz} \int _{x_1}^x \tilde{b}_2[z;D,W] e^{\int _{x_1}^z a_2[s;D,W]\,ds}\,dz. \end{aligned}$$

Observe that

6.428 $$\begin{aligned} Z^{-1} \le \frac{1}{x^2DW}\le \frac{6}{x^2 D}, \end{aligned}$$

where we have used $H+xW\ge xW$, $(1-\varepsilon )J+ 2\varepsilon x \left( 1+D\right) \ge (1-\varepsilon )xD+ 2\varepsilon x \left( 1+D\right) \ge xD$, and finally $W\ge \frac{1}{6}$. Therefore

6.429 $$\begin{aligned} \left| \tilde{b}_2[x;D,W]\right|&\le \frac{2x^2D}{Z} \Big (\left| \left[ D^2+(3+\varepsilon )D-(1-\varepsilon )\right] \right| \nonumber \\&\ \ \ \ + 2\varepsilon \left| \left[ \frac{4}{1-\varepsilon }D^2+\frac{2(1+\varepsilon )}{1-\varepsilon }(1+D) + \frac{5-\varepsilon }{1-\varepsilon }D+3\right] \right| \Big ) \nonumber \\&\le C, \end{aligned}$$

for some universal constant *C*, which follows from (6.428) and the a priori bound $D\le D_\delta \le 1$.

We note that by (2.46) and (3.125),

6.430 $$\begin{aligned} 2\varepsilon \left| \left( J-xW\right) \left( D-1\right) Z^{-1}\right|&= 2\varepsilon \left| \frac{ \left( J-xW\right) \left( D-1\right) }{\left[ (1-\varepsilon )J+ 2\varepsilon x \left( 1+D\right) \right] \left[ H+xW\right] } \right| \nonumber \\&= 2\varepsilon \left| \frac{ \left( J-xW\right) \left( D-1\right) }{\left[ (1-\varepsilon )J+ 2\varepsilon x \left( 1+D\right) \right] \left[ J[D] +\frac{4\varepsilon }{1-\varepsilon }(1+D)x+xW\right] } \right| \nonumber \\&\le 2\varepsilon \frac{ \left| J+xW\right| \left( 1-D_\delta \right) }{\left[ (1-\varepsilon )J+ 2\varepsilon x \left( 1+D\right) \right] \left[ J+\frac{4\varepsilon }{1-\varepsilon }(1+D)x+xW\right] } \nonumber \\&\le \frac{1-D_\delta }{ x} \le 1, \end{aligned}$$

where we have used $[(1-\varepsilon )J+ 2\varepsilon x \left( 1+D\right) \ge 2\varepsilon x$, $J+\frac{4\varepsilon }{1-\varepsilon }(1+D)x+xW\ge J+xW$, and $x\ge x_*\ge 1$. Similarly,

6.431 $$\begin{aligned} 2\varepsilon \left| \frac{f}{Z}\right|&\le \frac{J+xD}{\left[ (1-\varepsilon )J+ 2\varepsilon x \left( 1+D\right) \right] \left[ H+xW\right] } \nonumber \\&\le 2\varepsilon \frac{J+xW}{\left[ (1-\varepsilon )xD+ 2\varepsilon x \left( 1+D\right) \right] \left[ H+xW\right] } \nonumber \\&\le 2\varepsilon \frac{1}{2\varepsilon x} = \frac{1}{x}\le 1, \end{aligned}$$

where we have used $f = J-xD\le J+xD$ in the first line, $D\le W$ and $J>xD$ in the second, and finally $J+xW\le H+xW$, $(1-\varepsilon )xD+ 2\varepsilon x \left( 1+D\right) \ge 2\varepsilon x$, and $x\ge x_*\ge 1$ in the last line. Also, since $H+xW\ge xW\ge \frac{1}{6} x$ and $(1-\varepsilon )xD+ 2\varepsilon x \left( 1+D\right) \ge 2\varepsilon x$, we have the following simple bound

6.432 $$\begin{aligned} Z^{-1} \le \frac{3}{\varepsilon x^2}. \end{aligned}$$

Since $D\le D_\delta \le 1$, we can use (6.430), (6.431), (6.428), (6.432), and the definition (3.132) of $a_2[x;D,W]$ to conclude that

6.433 $$\begin{aligned} \left| a_2[x;D,W]\right|&\le 2\varepsilon \left| \left( J-xW\right) \left( D-1\right) Z^{-1}\right| + 4\varepsilon \left| \frac{f}{Z}\right| + 2\varepsilon Z^{-1} \left( 4\varepsilon x + xD\left( 5+\varepsilon \right) \right) \nonumber \\&\le C. \end{aligned}$$

We now feed the bounds (6.429) and (6.433) into (6.427) to conclude

6.434 $$\begin{aligned}&e^{C(x-x_1)}f(x)\ge f(x)e^{\int _{x_1}^x a_2[z;D,W]\,dz} \nonumber \\&\quad \ge f(x_1) - \varepsilon C e^{C(x-x_1)}(x-x_1), \ \ x\in (x_1, X), \ \ x_1\in [x_*+\delta , X_1). \end{aligned}$$

Let $x_1 = x_*+\delta$ so that by Lemma 4.23$f(x_1)>\bar{c}$ for some $\bar{c}>0$ and all $\varepsilon \in (0,\varepsilon _0]$ and all $x_*\in [x_{\text {min}},x_{\text {max}}]$. From (6.434) we conclude that we can choose $\varepsilon _0$ so that $f(x)>c>0$ for all $x\in (x_*+\delta , \min \{\tilde{x},X_1\})$, where we recall $\tilde{x} = 48$. By the definition of $X_1$, this concludes the proof of (6.424).

To prove the remaining claim, by (6.434) we need only to address the case when $X>\tilde{x}=48$. In this case $\tilde{b}_2$ is strictly positive for $x>\tilde{x}$ by the argument following (6.425) and therefore by (6.427) and (6.433)

6.435 $$\begin{aligned} f(x) \ge f(\tilde{x}) e^{-\int _{\tilde{x}}^x a_2[z;D,W]\,dz} \ge f(\tilde{x}) e^{-C(x-\tilde{x})}, \end{aligned}$$

and thus the claim follows. $\square$

We are now ready to prove Theorem 2.4, which asserts global-to-the-right existence of RLP-type solutions.

*Proof of Theorem* 2.4. Let $$[x_*,X)$$ be the maximal time of existence on which

6.436 $$\begin{aligned} xW>J, \ \ x\in (x_*,X). \end{aligned}$$

By Lemma 6.3 we conclude that $J>xD$ on $(x_*+\delta , X)$ for all $\varepsilon \le \varepsilon _0$ sufficiently small and therefore all the conclusions of Lemma 6.2 apply. We argue by contradiction and assume that $X<\infty$. By Lemma 6.3 we know that there exists a constant $\bar{\kappa }= \bar{\kappa }(X)$ such that $f(x)>\bar{\kappa }$ for all $x\in (x_*+\delta , X)$. In particular $\limsup _{x\rightarrow X^-}(W(x)-D(x))>0$. It follows that

6.437 $$\begin{aligned} \limsup _{x\rightarrow X^-}\left( (xW)' - J'\right) \le 0. \end{aligned}$$

By (2.36) and (3.127), we have

6.438 $$\begin{aligned} (xW)' - J'&= 1-2W(x) + \frac{2x^2(1+\varepsilon ) W (W + \varepsilon ) (D-W)}{B} \nonumber \\&\ \ \ \ + \frac{ 2\varepsilon J(1+D) - \varepsilon (1-\varepsilon )x}{(1-\varepsilon ) J+ 2\varepsilon x (1+D)} +\frac{ (2\varepsilon x J+ \frac{\varepsilon }{1-\varepsilon } D^{-\eta -1}) D' }{(1-\varepsilon ) J+ 2\varepsilon x (1+D) } \nonumber \\&= 1-2W(x) + \frac{ 2\varepsilon J(1+D) - \varepsilon (1-\varepsilon )x}{(1-\varepsilon ) J+ 2\varepsilon x (1+D)} \nonumber \\&\ \ \ \ + \left( - \frac{x(1+\varepsilon )W}{(1-\varepsilon )D} + \varepsilon \frac{ 2 x J+ \frac{1}{1-\varepsilon } D^{-\eta -1} }{(1-\varepsilon ) J+ 2\varepsilon x (1+D)} \right) D' . \end{aligned}$$

By the uniform bounds (6.407) and (6.409) we have $\frac{1}{6}\le W \le \tilde{C}$. By (6.406) and (6.408) we have

6.439 $$\begin{aligned} D_\delta \ge D(x) \ge D_\delta ^2 \frac{(x_*+\delta )^3}{x^3 \tilde{C}} \ge D_\delta ^2 \frac{(x_*+\delta )^3}{X^3 \tilde{C}}. \end{aligned}$$

Therefore the first line of the right-most side of (6.438) is bounded. Since $\lim _{x\rightarrow X^-}\left( xW - J\right) =0$ it follows that $\lim _{x\rightarrow X^-}B = 0$ and therefore $\limsup _{x\rightarrow X^-} D'(x) = -\infty$ by (2.35). On the other hand, the second line of the right-most side of (6.438) is of the form $g(x) D'$, where

6.440 $$\begin{aligned} g(x)&= - \frac{x(1+\varepsilon )W}{(1-\varepsilon )D} + \varepsilon \frac{ 2 x J+ \frac{1}{1-\varepsilon } D^{-\eta -1} }{(1-\varepsilon ) J+ 2\varepsilon x (1+D)} \nonumber \\&= \frac{-xW(1+\varepsilon )\left[ (1-\varepsilon ) J+ 2\varepsilon x (1+D)\right] + \left[ 2 \varepsilon x J+ \frac{\varepsilon }{1-\varepsilon } D^{-\eta -1}\right] (1-\varepsilon )D}{(1-\varepsilon )D\left[ (1-\varepsilon ) J+ 2\varepsilon x (1+D)\right] } \nonumber \\&= \frac{-(1-\varepsilon ^2)xWJ- 2\varepsilon (1+\varepsilon )x^2 W(1+D)+2\varepsilon (1-\varepsilon )xDJ+ \varepsilon D^{-\eta }}{(1-\varepsilon )D\left[ (1-\varepsilon ) J+ 2\varepsilon x (1+D)\right] }. \end{aligned}$$

We now use (6.403), which for any $x<X$ allows us to estimate the numerator of (6.440) from above:

6.441 $$\begin{aligned}&-(1-\varepsilon ^2)xWJ- 2\varepsilon (1+\varepsilon )x^2 W(1+D)+2\varepsilon (1-\varepsilon )xDJ+ \varepsilon D^{-\eta } \nonumber \\&< -(1-\varepsilon ^2)xWJ- 2\varepsilon (1+\varepsilon )x^2 W(1+D)+2\varepsilon (1-\varepsilon )xDJ\nonumber \\&\ \ \ \ + \varepsilon x^2\left( (1-\varepsilon )W^2- \varepsilon (1-\varepsilon ) +4\varepsilon W(1+D) \right) \nonumber \\&= x^2\left[ -(1-\varepsilon ^2)W\frac{J}{x} + 2\varepsilon (1-\varepsilon )D\frac{J}{x} +\varepsilon (1-\varepsilon )W^2 \right] \nonumber \\&\ \ \ \ - (2\varepsilon -2\varepsilon ^2) x^2 W(1+D) - x^2 \varepsilon ^2(1-\varepsilon ). \end{aligned}$$

As $x\rightarrow X$, $\frac{J}{x}$ approaches *W* and therefore the above expression is negative for $\varepsilon$ sufficiently small (and independent of *X*). Therefore by (6.440) $g(x)<0$ as $x\rightarrow X^-$, which implies that the right-most side of (6.438) blows up to $+\infty$ as $x\rightarrow X^-$, which is a contradiction to (6.437). This concludes the proof of the theorem. $\square$

### Asymptotic Behaviour as $x\rightarrow \infty$

We introduce the unknown $\Pi (x)=\Sigma (y)$, where $\Sigma$ is the energy density introduced in (2.19). Recalling (2.34) and (2.26), we have

6.442 $$\begin{aligned} \Pi = D^{1+\eta } = D^{\frac{1+\varepsilon }{1-\varepsilon }}. \end{aligned}$$

It is straightforward to check that the system (2.35)–(2.36) can be reformulated as follows

6.443 $$\begin{aligned} \Pi '&= - \frac{2x(1+\varepsilon )\Pi (W+\varepsilon )(D-W)}{B}, \end{aligned}$$

6.444 $$\begin{aligned} W'&= \frac{(1 -3W )}{x} + \frac{2x(1+\varepsilon ) W (W + \varepsilon ) (D-W)}{B}. \end{aligned}$$

#### Lemma 6.4

There exist an $x_0>x_*$, a constant *C* and $\varepsilon _0>0$ sufficiently small so that for all $x>x_0$

6.445 $$\begin{aligned} \frac{\Pi '(x)x}{\Pi (x)}+2 = \varepsilon \alpha (x) (W-1) + \beta (x), \ \ x\in (x_*,\infty ), \end{aligned}$$

where

6.446 $$\begin{aligned} |\alpha (x)|&\le C \ \ x\ge x_0, \end{aligned}$$

6.447 $$\begin{aligned} |\beta (x)|&\le C x^{-\frac{3}{2}}, \ \ x\ge x_0. \end{aligned}$$

#### Proof

From (6.443) and the definition (3.123) of *B* it follows that

6.448 $$\begin{aligned} \frac{\Pi ' x}{\Pi } + 2&= \frac{2B - 2x^2(1+\varepsilon )(W+\varepsilon )(D-W)}{B} \nonumber \\&= 2\frac{D^{-\eta } - x^2\left[ (W+\varepsilon )^2-\varepsilon (W-1)^2+4\varepsilon DW + (1+\varepsilon )(W+\varepsilon )(D-W)\right] }{D^{-\eta }-x^2\left( (W+\varepsilon )^2-\varepsilon (W-1)^2+4\varepsilon DW\right) }. \end{aligned}$$

We rewrite the rectangular brackets in the numerator above in the form

6.449 $$\begin{aligned}&-\left[ (W+\varepsilon )^2-\varepsilon (W-1)^2+4\varepsilon DW + (1+\varepsilon )(W+\varepsilon )(D-W)\right] \nonumber \\&= \varepsilon (W-1)\left[ 2W+\varepsilon -1\right] - D\left( 5\varepsilon W +W+\varepsilon (1+\varepsilon )\right) . \end{aligned}$$

We feed this back into (6.448), divide the numerator and the denominator by $x^2$, and obtain

6.450 $$\begin{aligned} \frac{\Pi ' x}{\Pi } + 2&= 2\frac{ \varepsilon (W-1)\left[ 2W+\varepsilon -1\right] - D\left( 5\varepsilon W +W+\varepsilon (1+\varepsilon )\right) +D^{-\eta }x^{-2}}{-\left( (W+\varepsilon )^2-\varepsilon (W-1)^2+4\varepsilon DW\right) +D^{-\eta }x^{-2}} \nonumber \\&= \varepsilon \alpha (x) (W-1) + \beta (x), \end{aligned}$$

where

6.451 $$\begin{aligned} \alpha (x){:}{=} \frac{2\left( 2W+\varepsilon -1\right) }{-\left( (W+\varepsilon )^2-\varepsilon (W-1)^2+4\varepsilon DW\right) +D^{-\eta }x^{-2}} , \end{aligned}$$

6.452 $$\begin{aligned} \beta (x){:}{=}\frac{-2D\left( 5\varepsilon W +W+\varepsilon (1+\varepsilon )\right) +2D^{-\eta }x^{-2}}{-\left( (W+\varepsilon )^2-\varepsilon (W-1)^2+4\varepsilon DW\right) +D^{-\eta }x^{-2}}. \end{aligned}$$

By (6.408) and $W\le \tilde{C}$, it follows that $D(x)\ge c_1 x^{-3}$ for some universal constant $c_1>0$. Together with (6.411) we conclude

6.453 $$\begin{aligned} c_1 x^{-3} \le D(x) \le c_2 x^{-\gamma }, \end{aligned}$$

where $\gamma =1-C\varepsilon _0$ for some $\varepsilon _0\ll 1$. We conclude that $D^{-\eta }x^{-2}\le c_1^{-\eta }x^{3\eta -2}$. Since $\frac{1}{6}\le W\le \tilde{C}$ by Lemma 6.2, for $\varepsilon \le \varepsilon _0$ sufficiently small we conclude that there exist a constant *C* and $x_0>x_*$ such that for all $x>x_0$

$$\begin{aligned} \frac{1}{C}\le \left| -\left( (W+\varepsilon )^2-\varepsilon (W-1)^2+4\varepsilon DW\right) +D^{-\eta }x^{-2}\right| \le C. \end{aligned}$$

This immediately yields (6.446). Note that the leading order behaviour in the rectangular brackets in the very last line of (6.422) is of the form $\gamma (W-D) + (2-2\gamma ) W+O(\varepsilon )$. We use the decay bound (6.411), namely $D(x)\lesssim x^{-1+C\varepsilon }$, and we see that the right-most side of (6.422) becomes positive with $\gamma =\frac{3}{2}$, with *x* sufficiently large and $\varepsilon$ sufficiently small. The identity (6.421) yields (6.447). $\square$

#### Lemma 6.5

Let $x_*\in [x_{\text {min}},x_{\text {max}}]$ and let $(W,D)$ be the unique RLP-type solution defined globally to the right for all $\varepsilon \in (0,\varepsilon _0]$. Then for any $\varepsilon \in (0,\varepsilon _0]$ there exists a constant $d_2>0$ such that

6.454 $$\begin{aligned} \lim _{x\rightarrow \infty }W(x)&= 1, \end{aligned}$$

6.455 $$\begin{aligned} \lim _{x\rightarrow \infty }\Pi (x)x^2&= d_2 . \end{aligned}$$

Claim (6.455) equivalently reads

6.456 $$\begin{aligned} \lim _{x\rightarrow \infty }D(x)x^{2\frac{1-\varepsilon }{1+\varepsilon }} = d_2^{\frac{1-\varepsilon }{1+\varepsilon }}. \end{aligned}$$

#### Proof

Note that by (6.444)

6.457 $$\begin{aligned} \left( (W-1)x\right) '&= W'x + W-1 = -2W + \frac{2x^2(1+\varepsilon ) W (W + \varepsilon ) (D-W)}{B} \nonumber \\&= -W \frac{2B - 2x^2(1+\varepsilon )(W+\varepsilon )(D-W)}{B}. \end{aligned}$$

From this and the first line of (6.448) we conclude

6.458 $$\begin{aligned} \left( (W-1)x\right) ' =- W \left( \frac{\Pi ' x}{\Pi } + 2\right) . \end{aligned}$$

Let now

6.459 $$\begin{aligned} \vartheta (x): = (1-W)x. \end{aligned}$$

Equation (6.458) and Lemma 6.4 now give

6.460 $$\begin{aligned} \vartheta '(x) = -\varepsilon \frac{W(x)\alpha (x)}{x} \vartheta (x) + W(x)\beta (x). \end{aligned}$$

We now use the integrating factor to solve for $\vartheta$. For any $x_0>x_*$ we have

6.461 $$\begin{aligned} \vartheta (x) e^{\varepsilon \int _{x_0}^x \frac{W(s)\alpha (s)}{s}\,ds} = \vartheta (x_0) + \int _{x_0}^x W(s)\beta (s) e^{\varepsilon \int _{x_0}^s \frac{W(s')\alpha (s')}{s'}\,ds'} \,ds. \end{aligned}$$

By Lemma 6.4 and the bound $\frac{1}{6}\le W\le \tilde{C}$ from Lemma 6.2 we have $|\alpha W|\le C$ and therefore

6.462 $$\begin{aligned} \left( \frac{x}{x_0}\right) ^{-C\varepsilon }\le e^{\varepsilon \int _{x_0}^x \frac{W(s)\alpha (s)}{s}\,ds} \le \left( \frac{x}{x_0}\right) ^{C\varepsilon }. \end{aligned}$$

We use this bound in (6.461) and together with (6.447) we conclude that

6.463 $$\begin{aligned} \left( \frac{x}{x_0}\right) ^{-C\varepsilon } |\vartheta (x)|&\le |\vartheta (x_0)| + C \int _{x_0}^x s^{-\frac{3}{2}+C\varepsilon }\,ds \le C. \end{aligned}$$

We now recall the definition (6.459) of $\vartheta$ and conclude from the above bound that

6.464 $$\begin{aligned} |1-W| \le C x^{-1+C\varepsilon }, \text { for all }\ \ \varepsilon \in (0,\varepsilon _0]. \end{aligned}$$

where we recall (6.410). The claim (6.454) follows.

From Lemma 6.4 we conclude

6.465 $$\begin{aligned} \log (\Pi (x)x^2)&= \log (\Pi (x_0)x_0^2) + \int _{x_0}^x \left( \varepsilon \frac{\alpha (s)}{s} (W(s)-1) + \frac{\beta (s)}{s} \right) \,ds, \end{aligned}$$

and thus

6.466 $$\begin{aligned} \Pi (x) x^2 = \Pi (x_0) x_0^2 \, e^{\int _{x_0}^x \left( \varepsilon \frac{\alpha (s)}{s} (W(s)-1) + \frac{\beta (s)}{s} \right) \,ds}. \end{aligned}$$

We now use (6.464) and Lemma 6.4 to conclude that $\left| \varepsilon \frac{\alpha (s)}{s} (W(s)-1) + \frac{\beta (s)}{s}\right| \lesssim s^{-2+O(\varepsilon )}$ and is therefore integrable. Letting $x\rightarrow \infty$ we conclude (6.455). That (6.456) is equivalent to (6.455) follows from (6.442). $\square$

#### Remark 6.6

Stronger versions of the claims (6.454) and (6.455) are contained in (6.464) and (6.466) respectively. Formula (6.466) can be used to give a quantitative decay bound for

$$\begin{aligned} \left| \Pi (x)x^2-d_2\right| , \ \ \text { as } \ x\rightarrow \infty . \end{aligned}$$

In the following lemma we establish the strict upper bound $W<1$ for the RLP-type solutions and provide the crucial $\varepsilon$-independent bounds at spacelike hypersurface $x=+\infty$, which will play an important role in the construction of the maximal selfsimilar extension of the RLP spacetime.

#### Lemma 6.7

(*W* stays below 1) Let $x_*\in [x_{\text {min}},x_{\text {max}}]$ and let $(W,D)$ be the unique RLP-type solution defined globally to the right for all $\varepsilon \in (0,\varepsilon _0]$. Then there exist a sufficiently small $0<\varepsilon _0\ll 1$ and constants $c_2>c_1>0$ such that

6.467 $$\begin{aligned} W(x)<1, \ \ x\in [x_*,\infty ), \ \ \text { for all } \ \varepsilon \in (0,\varepsilon _0], \end{aligned}$$

and

6.468 $$\begin{aligned} 1+ c_2\ge d_2 \ge 1+c_1 \ \ \text { for all } \ \varepsilon \in (0,\varepsilon _0], \end{aligned}$$

where $$d_2 = \lim _{x\rightarrow \infty } \Pi (x)W(x)x^2 = \lim _{x\rightarrow \infty } \Pi (x)x^2$$.

#### Proof

We first observe that by (6.443)–(6.444)

6.469 $$\begin{aligned} (\Pi W x^2)' = \Pi x (1-W) = \Pi \vartheta , \end{aligned}$$

where we have used (6.459) in the second equality. By way of contradiction, assume that there exists an $\bar{x}\in (x_*,\infty )$ such that $W(\bar{x})=1$ and $W(x)<1$ for all $x\in (x_*,\bar{x})$. Integrating (6.469) over $[x_*,\bar{x}]$ we conclude that

6.470 $$\begin{aligned} \Pi (\bar{x}) \bar{x}^2&=\Pi (\bar{x}) W(\bar{x}) \bar{x}^2 \nonumber \\&= \Pi (x_*) W(x_*)x_*^2 +\int _{x_*}^{\bar{x}} \Pi (s) \vartheta (s)\,ds \nonumber \\&= W_0^{2+\eta }x_*^2 +\int _{x_*}^{\bar{x}} \Pi (s) \vartheta (s)\,ds, \end{aligned}$$

where we recall (6.442). On the other hand, clearly $W'(\bar{x})\ge 0$ and therefore, by (6.444), at $\bar{x}$ we have

6.471 $$\begin{aligned} 0\le W'(\bar{x})&= -\frac{2}{\bar{x}} + \frac{2\bar{x}(1+\varepsilon )^2(D-1)}{D^{-\eta }-\left( (1+\varepsilon )^2+4\varepsilon D\right) \bar{x}^2} \nonumber \\&= \frac{-2\left( D^{-\eta }-\left( (1+\varepsilon )^2+4\varepsilon D\right) \bar{x}^2\right) +2\bar{x}^2(1+\varepsilon )^2(D-1)}{\bar{x}\left( D^{-\eta }-\left( (1+\varepsilon )^2+4\varepsilon D\right) \bar{x}^2\right) } \nonumber \\&= \frac{-2 D^{-\eta } +2D\bar{x}^2 \left( 4\varepsilon + (1+\varepsilon )^2\right) }{\bar{x}\left( D^{-\eta }-\left( (1+\varepsilon )^2+4\varepsilon D\right) \bar{x}^2\right) } \nonumber \\&= - 2 D^{-\eta } \frac{1- \Pi \bar{x}^2\left( 4\varepsilon + (1+\varepsilon )^2\right) }{\bar{x}\left( D^{-\eta }-\left( (1+\varepsilon )^2+4\varepsilon D\right) \bar{x}^2\right) }. \end{aligned}$$

Since the denominator is strictly negative, it follows that

6.472 $$\begin{aligned} \Pi (\bar{x}) \bar{x}^2 \le \frac{1}{4\varepsilon + (1+\varepsilon )^2}. \end{aligned}$$

Combining (6.470) and (6.472) we conclude that

6.473 $$\begin{aligned} \frac{1}{4\varepsilon + (1+\varepsilon )^2} \ge W_0^{2+\eta }x_*^2 + \int _{x_*}^{\bar{x}} \Pi (s) \vartheta (s)\,ds. \end{aligned}$$

Our goal is to provide a lower bound for $\vartheta (x)$ which is uniform-in-$\varepsilon$. Using (6.461),(6.462), and (6.447), we conclude that for any $\bar{x}>x>x_0>x_*$

6.474 $$\begin{aligned} \left( \frac{x}{x_0}\right) ^{C\varepsilon } |\vartheta (x)|&\ge \vartheta (x_0) - C \int _{x_0}^x s^{-\frac{3}{2}+C\varepsilon }\,ds. \end{aligned}$$

Therefore

6.475 $$\begin{aligned} |\vartheta (x)| \ge \vartheta (x_0)\left( \frac{x}{x_0}\right) ^{-C\varepsilon } - C\int _{x_0}^x s^{-\frac{3}{2}+C\varepsilon }\,ds \left( \frac{x}{x_0}\right) ^{-C\varepsilon }. \end{aligned}$$

Now choose $x_0=x_*+\delta$ with $\delta$ as in Lemma 4.23 so that, by Lemma 4.23 we can ascertain the *uniform-in*-$\varepsilon$ bound

6.476 $$\begin{aligned} \vartheta (x_0)\ge \frac{1}{2}, \ \ \text { for all} \ \varepsilon \in (0,\varepsilon _0]. \end{aligned}$$

This bound together with the lower bound (6.475) yields

6.477 $$\begin{aligned} |\vartheta (x)| \ge \frac{1}{2}\left( \frac{x}{x_0}\right) ^{-C\varepsilon } - C\int _{x_0}^x s^{-\frac{3}{2}+C\varepsilon }\,ds \left( \frac{x}{x_0}\right) ^{-C\varepsilon }, \ \ \text { for all} \ \varepsilon \in (0,\varepsilon _0]. \end{aligned}$$

The right-hand side of (6.477) is a continuous function in *x* which converges to $\frac{1}{2}$ as $x\rightarrow x_0^-$. Therefore, there exists an $\varepsilon$-independent real number $\tilde{x}>x_0$ such that

6.478 $$\begin{aligned} \vartheta (x) \ge \frac{1}{4}, \ \ \text { for all } \ x\in [x_0,\tilde{x}] \ \ \text { and all } \ \varepsilon \in (0,\varepsilon _0]. \end{aligned}$$

Observe that the bound (6.478) also ascertains that $\tilde{x}<\bar{x}$, see (6.459).

It is easily checked from (6.443)–(6.444) that $(\Pi W x^3)'=\Pi x^2\ge 0$. Since $|W|\le \tilde{C}$ by Lemma 6.2 it then follows that

6.479 $$\begin{aligned} \Pi (x) \ge \frac{\Pi (x_*)W(x_*)x_*^3}{\tilde{C} x^3} = \frac{W_0^{2+\eta }x_*^3}{\tilde{C} x^3} \ge C x^{-3}, \ \ x>x_*, \end{aligned}$$

where, as usual, the constant *C* is independent of $\varepsilon$.

We now use the bounds (6.478) and (6.479) in (6.473) to conclude

6.480 $$\begin{aligned} \frac{1}{4\varepsilon + (1+\varepsilon )^2}&\ge W_0^{2+\eta }x_*^2 + \int _{x_*}^{\bar{x}} \Pi (s) \vartheta (s)\,ds \ge W_0^{2+\eta }x_*^2 + \int _{x_0}^{\tilde{x}} \Pi (s) \vartheta (s)\,ds \nonumber \\&\ge W_0^{2+\eta }x_*^2 + C \int _{x_0}^{\tilde{x}} s^{-3} \,ds \ge W_0^{2+\eta }x_*^2 + C \left( \frac{1}{x_0^2}-\frac{1}{\tilde{x}^2}\right) . \end{aligned}$$

Observe that we have used the non-negativity of $\vartheta$ on $[x_*,\bar{x}]$ in the second bound above.

By the $\varepsilon$-asymptotic behaviour of $W_0$ from Lemma 4.1 we have

$$\begin{aligned} W_0^{2+\eta }x_*^2 = 1+O(\varepsilon ), \end{aligned}$$

and clearly $\frac{1}{4\varepsilon + (1+\varepsilon )^2} = 1+O(\varepsilon )$. Since however the term $C \left( \frac{1}{x_0^2}-\frac{1}{\tilde{x}^2}\right)$ is bounded from below by some $\varepsilon$-independent positive real number $\tilde{\delta }>0$, we obtain contradiction by choosing $\varepsilon _0$ sufficiently small. This completes the proof of (6.467).

By Lemma 6.5 we may integrate (6.469) over the whole interval $[x_*,\infty )$ to conclude

6.481 $$\begin{aligned} \lim _{x\rightarrow \infty }\Pi (x) W(x) x^2&= \Pi (x_*) W(x_*)x_*^2 + \int _{x_*}^\infty \Pi (s) \vartheta (s)\,ds \nonumber \\&\ge \Pi (x_*) W(x_*)x_*^2 + \int _{x_0}^{\tilde{x}} \Pi (s) \vartheta (s)\,ds \nonumber \\&\ge W_0^{2+\eta }x_*^2 + C \left( \frac{1}{x_0^2}-\frac{1}{\tilde{x}^2}\right) \nonumber \\&\ge 1 + O(\varepsilon ) + \tilde{\delta } > 1, \end{aligned}$$

for $\varepsilon \le \varepsilon _0$ sufficiently small. This proves the lower bound stated in (6.468).

To prove the upper bound on $\Pi (x)W(x) x^2$, we rewrite (6.469) in the form $\log (\Pi W x^2)' = \frac{1-W}{xW}$ and integrate to obtain the identity

6.482 $$\begin{aligned} \log (\Pi (x) W(x) x^2) = \log (\Pi (x_0) W(x_0) x_0^2) + \int _{x_0}^x \frac{1-W(s)}{sW(s)}\,ds, \ \ x_*\le x_0 < x. \end{aligned}$$

Using (6.464) and (6.407) we conclude from (6.482) that

6.483 $$\begin{aligned} \log (\Pi (x) W(x) x^2) \le \log (\Pi (x_0) W(x_0) x_0^2) + 6C \int _{x_0}^x s^{-2+C\varepsilon }\,ds, \end{aligned}$$

where the constant *C* does not depend on $\varepsilon$. We let $x_0=x_*$, so that for sufficiently small $\varepsilon \le \varepsilon _0$ the $\varepsilon$-dependent quantity $\Pi (x_*) W(x_*)$ is bounded uniformly-in-$\varepsilon$. On the other hand

$$\begin{aligned} 6C \int _{x_*}^x s^{-2+C\varepsilon }\,ds \le 6C \int _{x_*}^\infty s^{-2+C\varepsilon }\,ds = \frac{6C}{1-C\varepsilon } x_*^{-1+C\varepsilon }, \end{aligned}$$

which, since $x_*\in [x_{\text {min}},x_{\text {max}}]$, is also bounded uniformly-in-$\varepsilon$. This completes the proof of the lemma. $\square$

In the following proposition, we establish the sharp asymptotic behaviour of the variable *W* as $x\rightarrow \infty$ - this will play an important role in constructing the unique extension of the solution in Section 7.

#### Proposition 6.8

(Precise asymptotic behaviour for *W*) Let $x_*\in [x_{\text {min}},x_{\text {max}}]$ and let $(W,D)$ be the unique RLP-type solution defined globally to the right for all $\varepsilon \in (0,\varepsilon _0]$. After choosing a possibly smaller $\varepsilon _0>0$, there exists a constant $\tilde{c}$ such that for any $\varepsilon \in (0,\varepsilon _0]$ there exists a constant $w_1<0$ such that

6.484 $$\begin{aligned} 1-W(x) = -w_1 x^{-\frac{1-\varepsilon }{1+\varepsilon }} + o_{x\rightarrow \infty }(x^{\frac{1-\varepsilon }{1+\varepsilon }}), \ \ x\ge x_*, \end{aligned}$$

where $w_1< - \tilde{c}<0$ for all $\varepsilon \in (0,\varepsilon _0]$.

#### Proof

We may now derive more precise asymptotics for the functions $\alpha (x)$ and $\beta (x)$ from (6.451)–(6.452). Using (6.456) and (6.454) we see that

6.485 $$\begin{aligned} \lim _{x\rightarrow \infty }\frac{\beta (x)}{D(x)}&= \frac{2}{(1+\varepsilon )^2} \left( 1+\varepsilon (6+\varepsilon ) - \lim _{x\rightarrow \infty }\left( D^{-\frac{1+\varepsilon }{1-\varepsilon }}x^{-2}\right) \right) \nonumber \\&= \frac{2}{(1+\varepsilon )^2} \left( 1+\varepsilon (6+\varepsilon ) -\frac{1}{c}\right) , \end{aligned}$$

where $1+c_2>c= \lim _{x\rightarrow \infty }\left( D^{\frac{1+\varepsilon }{1-\varepsilon }}x^{2}\right) >1+c_1$ for all $\varepsilon \in (0,\varepsilon _0]$ by Lemma 6.7. We conclude that there exists a constant $\tilde{c}>0$ such that for all $\varepsilon \in (0,\varepsilon _0]$,

6.486 $$\begin{aligned} \frac{1}{\tilde{c}}>\lim _{x\rightarrow \infty } \frac{\beta (x)}{x^{-2\frac{1-\varepsilon }{1+\varepsilon }}} >\tilde{c}. \end{aligned}$$

Using (6.454) and (6.456), it is easy to see that

6.487 $$\begin{aligned} \alpha (x) \asymp _{x\rightarrow \infty } -\frac{2}{1+\varepsilon }. \end{aligned}$$

Recall now $\vartheta$ from (6.459). Letting $W = 1 + (W-1) = 1- \frac{\vartheta }{x}$, we may rewrite (6.460) in the form

6.488 $$\begin{aligned} \vartheta '(x) = \frac{2\varepsilon }{1+\varepsilon } \frac{\vartheta (x)}{x} + \frac{\varepsilon \vartheta (x)^2}{x^2} \alpha (x) -\varepsilon \tilde{\alpha }(x)\frac{\vartheta (x)}{x} + W(x)\beta (x), \ \ \alpha = -\frac{2}{1+\varepsilon } + \tilde{\alpha }, \end{aligned}$$

with $\lim _{x\rightarrow \infty }\tilde{\alpha }(x)=0$. This yields the identity

6.489 $$\begin{aligned} \left( \vartheta x^{-\frac{2\varepsilon }{1+\varepsilon }}\right) ' = x^{-\frac{2\varepsilon }{1+\varepsilon }} \left( \frac{\varepsilon \vartheta (x)^2}{x^2} \alpha (x) -\varepsilon \tilde{\alpha }(x)\frac{\vartheta (x)}{x} + W(x)\beta (x)\right) . \end{aligned}$$

By the bound (6.464) and by (6.486)–(6.487), it follows that the right-hand side of of the above identity is integrable on $[x_*,\infty )$ and therefore (6.484) holds for some $v_1\le 0$ (note that $v_1$ cannot be positive by Lemma 6.7). To prove the $\varepsilon$-uniform upper bound on $v_1$, we must first estimate the rate of decay of $\tilde{\alpha }(x)$ as $x\rightarrow \infty$. From (6.488) and (6.451), we directly check

6.490 $$\begin{aligned} \tilde{\alpha }(x) = - \frac{2\left( (1-\varepsilon )(1-W)^2+4\varepsilon DW-D^{-\eta }x^{-2}\right) }{(1+\varepsilon )\left( D^{-\eta }x^{-2}-\left( (W+\varepsilon )^2-\varepsilon (W-1)^2+4\varepsilon DW\right) \right) }. \end{aligned}$$

Therefore, for sufficiently large $x\gg 1$ we have the upper bound

6.491 $$\begin{aligned} |\tilde{\alpha }(x)| \le K x^{-2+O(\varepsilon )}, \end{aligned}$$

for some $K>0$ independent of $\varepsilon$. Here we have used (6.464), the bound (6.456), and Lemma 6.5. Keeping in mind (6.459), the bound $1-W>0$ (from Lemma 6.7) and (6.486) we conclude that for any $x\ge x_*$ sufficiently large we have

6.492 $$\begin{aligned}&-w_1=\lim _{x\rightarrow \infty }\left( (1-W(x)) x^{\frac{1-\varepsilon }{1+\varepsilon }}\right) = \lim _{x\rightarrow \infty }\left( \vartheta (x)x^{-\frac{2\varepsilon }{1+\varepsilon }}\right) \nonumber \\&\quad = \left( 1-W(x_0)\right) x_0^{\frac{1-\varepsilon }{1+\varepsilon }} + \int _{x_0}^\infty \varepsilon x^{-\frac{2\varepsilon }{1+\varepsilon }} \left( \frac{ \vartheta (x)^2}{x^2} \alpha (x) - \tilde{\alpha }(x)\frac{\vartheta (x)}{x}\right) + x^{-\frac{2\varepsilon }{1+\varepsilon }} W(x)\beta (x) \, dx \nonumber \\&\quad \ge \varepsilon \int _{x_0}^\infty x^{-\frac{2\varepsilon }{1+\varepsilon }} \left( \frac{ \vartheta (x)^2}{x^2} \alpha (x) - \tilde{\alpha }(x)\frac{\vartheta (x)}{x}\right) \, dx + C_1 \int _{x_0}^\infty x^{-\frac{2\varepsilon }{1+\varepsilon }} x^{-2\frac{1-\varepsilon }{1+\varepsilon }}\,dx \nonumber \\&\quad \ge C_1 \int _{x_0}^\infty x^{-\frac{2}{1+\varepsilon }}\,dx - C_2 \varepsilon \int _{x_0}^\infty x^{-3+C\varepsilon }\,dx, \end{aligned}$$

for some constants $C_1,C_2>0$ and independent of $\varepsilon$. Note that we have used $W\ge \frac{1}{6}$ in the third line and (6.491) in the last. This implies the claim for $\varepsilon$ sufficiently small. $\square$

## Maximal Analytic Extension

The main results of this section are the description of the local and the global extension of the flow across the coordinate singularity at $y=\infty$, see Theorems 7.4 and 2.5 respectively. For a detailed overview, we refer to Section 2.5.

### Adapted Comoving Coordinates

As explained in Section 2.5 the metric *g* given by (2.7) becomes singular as $y\rightarrow \infty$. To show that this is merely a coordinate singularity, we introduced the adapted comoving chart (2.54), and the new selfsimilar variable *Y* given by (2.55)–(2.56). A simple manipulation of (2.54) gives the relation

7.493 $$\begin{aligned} \tau = \tau (\tilde{\tau },R) = Y^\eta \tilde{\tau }, \ \ \tau <0, \ R>0. \end{aligned}$$

Recall the new unknowns $\chi (Y)$, $d(Y)$, and *w*(*Y*) from (2.57). In the new chart the spacetime metric (2.7) by (2.19)–(2.24), (7.493), and (2.57) takes the form

$$\begin{aligned} g = - e^{2\tilde{\mu }(Y)}\,d\tilde{\tau }^2 - \frac{4\sqrt{\varepsilon }}{1+\varepsilon } Y e^{2\tilde{\mu }(Y)}\,d\tilde{\tau }\,dR + \left( e^{2\tilde{\lambda }(Y)}-\frac{4\varepsilon }{(1+\varepsilon )^2} Y^2 e^{2\tilde{\mu }(Y)} \right) \,dR^2 + R^2 \chi (Y)^2\, \gamma , \end{aligned}$$

where we recall (2.68)–(2.69). Note that by (2.52) and (2.68), we have

7.494 $$\begin{aligned} e^{2\tilde{\mu }(Y)} = \frac{1}{(1-\varepsilon )^2} \left( dY^{-2}\right) ^{-\eta }, \ \ Y>0. \end{aligned}$$

It is clear that in the limit $y\rightarrow \infty$, i.e. $Y\rightarrow 0^+$ the metric coefficient $e^{2\tilde{\mu }(Y)}$ approaches a positive constant due to (2.53) and (2.56).

#### Lemma 7.1

Let the triple $(\tilde{r},\textbf{d},\textbf{w})$ be a smooth solution to (3.117)–(3.118). Then the new unknowns $(\chi ,d,w)$ defined in (2.57) solve the system (2.58)–(2.61).

#### Proof

The Lagrangian system (3.117)–(3.118) now takes the form

7.495 $$\begin{aligned} \textbf{d}'&= - \frac{\textbf{w}+\varepsilon }{1+\varepsilon } \ \frac{2(1-\varepsilon )\tilde{r}^2}{y} \frac{ \textbf{d}(\textbf{w}+\varepsilon )(\textbf{d}-\textbf{w})}{\mathcal {B}} , \end{aligned}$$

7.496 $$\begin{aligned} \textbf{w}'&= \frac{\textbf{w}+\varepsilon }{1+\varepsilon }\left( \frac{1-3\textbf{w}}{y} +\frac{2(1+\varepsilon )\tilde{r}^2}{y} \frac{\textbf{w}(\textbf{w}+\varepsilon )(\textbf{d}-\textbf{w})}{\mathcal {B}}\right) , \end{aligned}$$

7.497 $$\begin{aligned} \tilde{r}'&= \frac{\tilde{r}}{y} \ \frac{\textbf{w}+\varepsilon }{1+\varepsilon }, \end{aligned}$$

where

7.498 $$\begin{aligned} \mathcal {B} = \textbf{d}^{-\eta } - \tilde{r}^2 \left[ (\textbf{w}+ \varepsilon )^2-\varepsilon (\textbf{w}-1)^2 + 4\varepsilon \textbf{w}\textbf{d}\right] , \end{aligned}$$

and $'$ refers to differentiation with respect to *y*. Using (2.57) it is then straightforward to check that (2.58)–(2.61) hold. $\square$

#### Remark 7.2

From (7.497) and the leading order behaviour of $\textbf{w}(y)=w(Y)$ at $y=\infty$ it is easy to see that

7.499 $$\begin{aligned}&\tilde{r}(y) = a y + o_{y\rightarrow +\infty }(y) = a \frac{1}{Y^{1+\eta }} + o_{Y\rightarrow 0^+}(\frac{1}{Y^{1+\eta }}), \ Y>0, \end{aligned}$$

7.500 $$\begin{aligned}&\lim _{Y\rightarrow 0^+}\chi (Y) = a, \end{aligned}$$

for some constant $a>0$. Here the constant *a* corresponds to the labelling gauge freedom and we set without loss of generality

7.501 $$\begin{aligned} a=1. \end{aligned}$$

We note that the unknown $d$ corresponds to the modified density $\textbf{d}$ defined in (2.57). By Lemma 6.5 and Proposition 6.8, and the asymptotic behaviour (7.499) with $a=1$, we have the leading order asymptotic behaviour

7.502 $$\begin{aligned} d(Y)&= d_2 Y^2 + o_{Y\rightarrow 0^+}\left( Y^2 \right) , \end{aligned}$$

7.503 $$\begin{aligned} w(Y)&= 1 + w_1 Y + o_{Y\rightarrow 0^+}\left( Y\right) , \end{aligned}$$

where $$1+c_2>d_2>1+c_1$$ and

7.504 $$\begin{aligned} w_1<-\tilde{c}<0 \end{aligned}$$

by Lemma 6.7 and Proposition 6.8. We note that

7.505 $$\begin{aligned} \lim _{Y\rightarrow 0^+} \frac{\chi (Y)^2}{Y^{2+2\eta }} w(Y)d^{1+\eta }(Y) = \lim _{x\rightarrow \infty } \Pi (x)W(x)x^2 = d_2 >1 \end{aligned}$$

by Lemma 6.7.

### Local Extension

To prove the existence of a solution to (2.58)–(2.60) with suitable boundary conditions (see (7.528)–(7.530)), we formally Taylor-expand the unknowns $d,w,\chi$ around $Y=0$ and prove the convergence of the series. Assume that the following expansions hold

7.506 $$\begin{aligned} d(Y)&= d_2 Y^2 + \sum _{N=3}^\infty d_N Y^N, \end{aligned}$$

7.507 $$\begin{aligned} w(Y)&= 1 + \sum _{N=1}^\infty w_N Y^N , \end{aligned}$$

7.508 $$\begin{aligned} \chi (Y)&= \chi _0 + \sum _{N=1}^\infty \chi _N Y^N, \end{aligned}$$

where by (7.501) $\chi _0=1$. From this and (2.61) we have the formal expansion

7.509 $$\begin{aligned} \mathcal {C} = \sum _{N=0}^\infty \mathcal {C}_N Y^N, \end{aligned}$$

where for any $N\ge 0$

7.510 $$\begin{aligned} \mathcal {C}_N = \left( (dY^{-2})^{-\eta }\right) _{N-2} - \sum _{k+\ell =N} (\chi ^2)_k\left[ (1-\varepsilon )(w^2)_\ell + 4\varepsilon w_\ell +(\varepsilon ^2-\varepsilon )\delta _{0\ell }+4\varepsilon (wd)_\ell \right] . \end{aligned}$$

Here we employ the convention that $(f)_j=0$ if $j<0$. We may single out the leading order contribution on the right-hand side of (7.510):

7.511 $$\begin{aligned} \mathcal {C}_N&= - 4\varepsilon \chi _0^2 d_N - 2(1+\varepsilon )\chi _0^2 w_N - 2(1+\varepsilon )^2\chi _0 \chi _N + \mathcal {C}_{N,1}, \end{aligned}$$

where

7.512 $$\begin{aligned} \mathcal {C}_{N,1}{:}{=}&\left( (dY^{-2})^{-\eta }\right) _{N-2} - \sum _{\begin{array}{c} k+\ell =N \\ k,\ell \le N-1 \end{array}} (\chi ^2)_k\left[ (1-\varepsilon )(w^2)_\ell + 4\varepsilon w_\ell +(\varepsilon ^2-\varepsilon )\delta _{0\ell }+4\varepsilon (wd)_\ell \right] \nonumber \\&- \chi _0^2 \left[ (1-\varepsilon )\sum _{\begin{array}{c} k+\ell =N \\ k,\ell \le N-1 \end{array}}w_k w_\ell + 4\varepsilon \sum _{\begin{array}{c} k+\ell =N \\ \ell \le N-1 \end{array}}w_k d_\ell \right] . \end{aligned}$$

#### Lemma 7.3

For any $N\ge 1$, $N\in \mathbb N$, the Taylor coefficients $(d_N,w_N,\chi _N)$ satisfy the following recursive relations

7.513 $$\begin{aligned} (N-2)\chi _0^2(1+\varepsilon )^2d_N&= \mathcal {U}_N, \end{aligned}$$

7.514 $$\begin{aligned} (1-\varepsilon )(1+\varepsilon )^2 (N-1)\chi _0^2 w_N - 2(1+\varepsilon )\left( (1+\varepsilon )^2+4\varepsilon \right) \chi _0^2 d_N&= \mathcal {V}_N, \end{aligned}$$

7.515 $$\begin{aligned} \chi _0 w_N + (1-\varepsilon ) N \chi _N&=- \sum _{\begin{array}{c} k+\ell =N \\ k,\ell \le N-1 \end{array}}\chi _k w_\ell , \end{aligned}$$

where

7.516 $$\begin{aligned} \mathcal {U}_N&= \sum _{\begin{array}{c} k+j=N \\ k\le N-1 \end{array}} k d_k \mathcal {C}_j -2\sum _{\begin{array}{c} k+\ell +m+n=N \\ \ell \le N-1 \end{array}} (\chi ^2)_k d_\ell (d_m-w_m)\left( (w^2)_n+2\varepsilon w_n + \varepsilon ^2\delta _{0n}\right) , \ \ N\ge 3, \end{aligned}$$

7.517 $$\begin{aligned} \mathcal {V}_N&= (1-\varepsilon )\sum _{\begin{array}{c} k+j=N \\ k\le N-1 \end{array}} k w_k \mathcal {C}_j \nonumber \\&\ \ \ \ - \sum _{\begin{array}{c} k+\ell =N \\ k,\ell \le N-1 \end{array}} \left( 3(w^2)_k-(1-3\varepsilon )w_k\right) \mathcal {C}_\ell + 2(1+\varepsilon )\mathcal {C}_{N,1} + 3(1+\varepsilon )^2 \chi _0^2 \sum _{\begin{array}{c} k+\ell =N \\ k,\ell \le N-1 \end{array}} w_k w_\ell \nonumber \\&\ \ \ \ +2(1+\varepsilon )\sum _{\begin{array}{c} k+m+n=N \\ k,m,n\le N-1 \end{array}} (\chi ^2)_k (d_m-w_m)\left( (w^3)_n+2\varepsilon (w^2)_n + \varepsilon ^2w_n\right) \nonumber \\&- 2(1+\varepsilon )\chi _0^2 \left( \sum _{\begin{array}{c} k+\ell +n=N\\ k,\ell ,n\le N-1 \end{array}} w_kw_\ell w_n + 2\varepsilon \sum _{\begin{array}{c} k+\ell =N \\ k,\ell \le N-1 \end{array}} w_kw_\ell \right) - 2(1+\varepsilon )^3 \sum _{\begin{array}{c} k+\ell =N \\ k,\ell \le N-1 \end{array}} \chi _k \chi _\ell . \end{aligned}$$

#### Proof

*Proof of* (7.513). We multiply (2.58) by $Y\mathcal {C}$ and plug in the formal expansions (7.506)–(7.510). We formally obtain that the *N*-th Taylor coefficient in the expansion of $d' Y \mathcal {C}$ is given by

7.518 $$\begin{aligned} \sum _{k+j=N} k d_k \mathcal {C}_j. \end{aligned}$$

On the other hand, the *N*-th Taylor coefficient in the expansion of $2\chi ^2d(w+\varepsilon )^2(d-w)=2\chi ^2d(w^2+2\varepsilon w+\varepsilon ^2)(d-w)$ is easily checked to be

7.519 $$\begin{aligned} 2\sum _{k+\ell +m+n=N} (\chi ^2)_k d_\ell (d_m-w_m)\left( (w^2)_n+2\varepsilon w_n + \varepsilon ^2\delta _{0n}\right) . \end{aligned}$$

We now extract the leading order terms in (7.518) and (7.519) - these are the factors containing either $d_N$ or $w_N$. We see that

7.520 $$\begin{aligned} \sum _{k+j=N} k d_k \mathcal {C}_j&= N d_N \mathcal {C}_0 + \sum _{\begin{array}{c} k+j=N \\ k\le N-1 \end{array}} k d_k \mathcal {C}_j \nonumber \\&= - N\chi _0^2(1+\varepsilon )^2 d_N + \sum _{\begin{array}{c} k+j=N \\ k\le N-1 \end{array}} k d_k \mathcal {C}_j, \end{aligned}$$

where we have used $\mathcal {C}_0 = - \chi _0^2 (1+\varepsilon )^2$. Note that the term $\mathcal {C}_N$ does not contribute a copy of $d_N$ on the right-hand side above since $k=0$ when $j=N$. Similarly, the expression (7.519) can be split into

7.521 $$\begin{aligned} -2 \chi _0^2 (1+\varepsilon )^2d_N+ 2\sum _{\begin{array}{c} k+\ell +m+n=N \\ \ell \le N-1 \end{array}} (\chi ^2)_k d_\ell (d_m-w_m)\left( (w^2)_n+2\varepsilon w_n + \varepsilon ^2\delta _{0n}\right) . \end{aligned}$$

Note that we have repeatedly used the assumption $d_0=d_1 = 0$. Equating (7.520) and (7.521) we obtain the recursive relation (7.513).

*Proof of* (7.514). We multiply (2.59) by $(1-\varepsilon )Y\mathcal {C}$ and expand the two sides of the equation by analogy to the above. We obtain formally

7.522 $$\begin{aligned} (1-\varepsilon )w' Y\mathcal {C} = (1-\varepsilon )\sum _{N=0}^\infty \left( \sum _{k+j=N} k w_k \mathcal {C}_j\right) Y^N. \end{aligned}$$

Upon extracting the leading order term in the *N*-th Taylor coefficient on the right-hand side of (7.522), by analogy to (7.520) we obtain

7.523 $$\begin{aligned} (1-\varepsilon )\sum _{k+j=N} k w_k \mathcal {C}_j= - (1-\varepsilon )N\chi _0^2(1+\varepsilon )^2 w_N + (1-\varepsilon )\sum _{\begin{array}{c} k+j=N \\ k\le N-1 \end{array}} k w_k \mathcal {C}_j. \end{aligned}$$

We now turn our attention to the right-hand side. Observe that formally the *N*-th Taylor coefficient in the expansion of $-(w+\varepsilon )(1-3w)\mathcal {C} = \left( 3w^2-(1-3\varepsilon )w-\varepsilon \right) \mathcal {C}$ equals

7.524 $$\begin{aligned} \sum _{k+\ell =N} \left( 3(w^2)_k\mathcal {C}_\ell -(1-3\varepsilon )w_k\mathcal {C}_\ell -\varepsilon \delta _{0k}\mathcal {C}_\ell \right) . \end{aligned}$$

To single out the leading order contribution we use (7.511) and thus (7.524) can be rewritten in the form

7.525 $$\begin{aligned}&2(1+\varepsilon ) \mathcal {C}_N + \left( 3(w^2)_N-(1-3\varepsilon )w_N\right) \mathcal {C}_0 + \sum _{\begin{array}{c} k+\ell =N \\ k,\ell \le N-1 \end{array}} \left( 3(w^2)_k-(1-3\varepsilon )w_k\right) \mathcal {C}_\ell \nonumber \\&= -8\varepsilon (1+\varepsilon )\chi _0^2 d_N - 3(1+\varepsilon )^2(3+\varepsilon )\chi _0^2 w_N - 4(1+\varepsilon )^3\chi _0 \chi _N \nonumber \\&\ \ \ \ + \sum _{\begin{array}{c} k+\ell =N \\ k,\ell \le N-1 \end{array}} \left( 3(w^2)_k-(1-3\varepsilon )w_k\right) \mathcal {C}_\ell + 2(1+\varepsilon )\mathcal {C}_{N,1} - 3(1+\varepsilon )^2 \chi _0^2 \sum _{\begin{array}{c} k+\ell =N \\ k,\ell \le N-1 \end{array}} w_k w_\ell . \end{aligned}$$

By analogy to (7.519) and (7.521) we also check that the *N*-the Taylor coefficient in the expansion of $-2(1+\varepsilon )\chi ^2w(w+\varepsilon )^2(d-w)$ formally corresponds to

7.526 $$\begin{aligned} -2(1+\varepsilon )\sum _{k+m+n=N} (\chi ^2)_k (d_m-w_m)\left( (w^3)_n+2\varepsilon (w^2)_n + \varepsilon ^2w_n\right) . \end{aligned}$$

After isolating the leading order coefficients, the above expression can be rewritten in the form

7.527 $$\begin{aligned}&- 2(1+\varepsilon )^3 \chi _0^2 d_N+4(1+\varepsilon )^2(2+\varepsilon )\chi _0^2 w_N + 4(1+\varepsilon )^3 \chi _0\chi _N \nonumber \\&-2(1+\varepsilon )\sum _{\begin{array}{c} k+m+n=N \\ k,m,n\le N-1 \end{array}} (\chi ^2)_k (d_m-w_m)\left( (w^3)_n+2\varepsilon (w^2)_n + \varepsilon ^2w_n\right) \nonumber \\&+ 2(1+\varepsilon )\chi _0^2 \left( \sum _{\begin{array}{c} k+\ell +n=N\\ k,\ell ,n\le N-1 \end{array}} w_kw_\ell w_n + 2\varepsilon \sum _{\begin{array}{c} k+\ell =N \\ k,\ell \le N-1 \end{array}} w_kw_\ell \right) + 2(1+\varepsilon )^3 \sum _{\begin{array}{c} k+\ell =N \\ k,\ell \le N-1 \end{array}} \chi _k \chi _\ell . \end{aligned}$$

Claim (7.514) now follows from (7.523) (7.525), and (7.527).

*Proof of* (7.515). The claim follows by substituting the formal expansions for $\chi$ and $w$ into (2.60), comparing the coefficients and using $w_0=1$. $\square$

#### Theorem 7.4

(Local extension) There exists and $0<\varepsilon _0\ll 1$ and a $Y_0<0$ such that for any $\varepsilon \in (0,\varepsilon _0]$ there exists a unique analytic-in-*Y* solution to (2.58)–(2.60) on the interval $(-|Y_0|,|Y_0|)$ such that

7.528 $$\begin{aligned} \lim _{Y\rightarrow 0} \frac{d(Y)}{Y^2}&= d_2, \end{aligned}$$

7.529 $$\begin{aligned} w(0)&= 1, \ \ w'(0)=w_1, \end{aligned}$$

7.530 $$\begin{aligned} \chi (0)&=1, \end{aligned}$$

7.531 $$\begin{aligned} \mathcal {C}(Y)&<0, \ \ Y\in (-|Y_0|,|Y_0|), \end{aligned}$$

where $d_2, w_1$ are constants given by Lemma 6.7 and Proposition 6.8. Moreover,

*(a)*: There exists a $\delta >0$ such that
  7.532 $$\begin{aligned} d(Y_0)>\delta , \ \ \chi (Y_0)>\delta , \ \ \text { and } \ \ w(Y_0)<\frac{1}{\delta }\ \ \text { for all } \ \ \varepsilon \in (0,\varepsilon _0]. \end{aligned}$$
*(b)*: There exists a constant $c_0>0$ such that
  7.533 $$\begin{aligned} \chi ^2 w(dY^{-2})^{1+\eta }>1+c_0, \ \ \text { for all } \ Y\in [Y_0,0] \ \ \text { and all } \ \varepsilon \in (0,\varepsilon _0]. \end{aligned}$$
*(c)*: There exists a constant $\tilde{\delta }>0$ such that
  7.534 $$\begin{aligned} \frac{1}{100}>\frac{d}{w}\Big |_{Y=Y_0} > \tilde{\delta }, \ \ \varepsilon \in (0,\varepsilon _0]. \end{aligned}$$
*(d)*: There exists a constant $c_{w}>0$ such that
  7.535 $$\begin{aligned} w(Y_0)>1+ c_{w}, \ \ \text { for all } \ \ \varepsilon \in (0,\varepsilon _0]. \end{aligned}$$

#### Proof

The proof of existence of a local real-analytic solution is analogous to the proof of Theorem 4.18. We observe that the occurrence of the factors $(N-2)$ and $(N-1)$ in (7.513) and (7.514) respectively reflects the fact that $d_2$ and $w_1$ must be prescribed in order to consistently solve the ensuing recursive relations between the higher-order coefficients. We leave out the details, as they are similar to the ideas of the proof of Theorem 4.18.

*Proof of part (a).* Estimates (7.532) are a trivial consequence of the leading order Taylor expansions from Remark 7.2 the uniform-in-$\varepsilon$ positivity of $d_2$ and negativity of $w_1$.

*Proof of part (b).* Since there exists a constant $\tilde{c}_0$ such that $\lim _{Y\rightarrow 0}\left( \chi ^2 w(dY^{-2})^{1+\eta }\right) >1+\tilde{c}_0$ for all $\varepsilon \in (0,\varepsilon _0]$, by Taylor expansion around $Y=0$ we can ascertain that there is a constant $c_0>0$ and an interval $[-|Y_0|,|Y_0|]$, $Y_0<0$, such that (7.533) holds.

*Proof of part (c).* Observe that $\frac{d}{w}\Big |_{Y=0} =0$. Since $d$ is locally bounded from below and $w$ locally increases to the left, the bound follows from continuity.

*Proof of part (d).* By the Taylor expansion (7.507) around $$Y=0$$ and the uniform-in-$$\varepsilon $$ bound (7.504) we can guarantee that $$w'<0$$ on $$[Y_0,0)$$ and that $$c_{w}$$ can be chosen independently of $$\varepsilon $$. $$\square $$

#### Remark 7.5

Note that $Y_0$ is $\varepsilon$-independent, which plays an important role in our proof of the existence of outgoing null-geodesics from the scaling origin ${\mathcal {O}}$. The constants $d_2$ and $w_1$ act as initial conditions for the system (2.58)–(2.60) to extend the solution to the left.

It is a priori possible that the solution constructed by Theorem 7.4 does not coincide with the RLP solution constructed for $x\in (0,\infty )$. The overlapping region $(0,|Y_0|)$ expressed in the *x*-coordinate is given by $(X_0,\infty )$, where $X_0 = \left( \tilde{r}\left( |Y_0|^{-(1+\eta )}\right) \right) ^{-\frac{1}{1+\eta }}$. As it is less clear how to apply the standard uniqueness theorem for the problem phrased in the *Y*-coordinate, we shall revert to the coordinate

7.536 $$\begin{aligned} X = x^{-\frac{1}{1+\eta }}, \ \ x = X^{-1-\eta }, \end{aligned}$$

and reduce the question of uniqueness to the standard ODE theory.

#### Proposition 7.6

(Uniqueness of the RLP-extension) In the region $Y>0$, the solution constructed in Theorem 7.4 coincides with the RLP-type solution emanating from the sonic point $\bar{x}_*$ constructed in Section 6.

#### Proof

Introduce the unknowns

7.537 $$\begin{aligned} D(x)= X^2 \bar{D}(X), \ \ W(x) = 1+ X \bar{W}(X). \end{aligned}$$

A direct calculation gives

7.538 $$\begin{aligned} B(x)&= X^{-2-2\eta } \left( -(1+\varepsilon )^2 -2(1+\varepsilon )X\bar{W}+ X^2K_1(X;\bar{D},\bar{W})\right) , \end{aligned}$$

7.539 $$\begin{aligned} K_1(X;D,W)&{:}{=} \bar{D}^{-\eta } - (1-\varepsilon ) \bar{W}^2 - 4\varepsilon \bar{D} - 4\varepsilon X\bar{D}\bar{W}. \end{aligned}$$

Using (7.536) and (2.35)–(2.36), we may further compute

7.540 $$\begin{aligned} \frac{d}{dX}D&= D' \frac{dx}{dX} \nonumber \\&= 2X \frac{(1+\varepsilon )\bar{D}\left( 1+\varepsilon +X\bar{W}\right) \left( X^2\bar{D}-1-X\bar{W}\right) }{-(1+\varepsilon )^2 -2(1+\varepsilon )X\bar{W}+ X^2K_1(X;\bar{D},\bar{W})} \nonumber \\&= :2X\bar{D} + X^2 K_2(X,\bar{D},\bar{W}), \end{aligned}$$

where it is easy to check that the function $K_2(X,\bar{D},\bar{W})$ is Lipschitz. An analogous calculation based on (2.36) then gives

7.541 $$\begin{aligned} \frac{d}{dX}W&= W' \frac{dx}{dX} \nonumber \\&= (1+\eta )\left[ \frac{2}{X} +3\bar{W} + \frac{2}{X(1+\varepsilon )} \frac{\left( 1+X\bar{W}\right) \left( 1+\varepsilon +X\bar{W}\right) (X^2\bar{D}-1-X\bar{W})}{1 + \frac{2}{1+\varepsilon } X\bar{W} - \frac{X^2}{(1+\varepsilon )^2}K_1}\right] \nonumber \\&{=}{:} (1+\eta )\left[ \frac{2}{X} +3\bar{W} - \frac{2}{X} +\frac{1}{1+\eta }\bar{W} + X K_3(X;\bar{D},\bar{W}) \right] \nonumber \\&= \bar{W} + (1+\eta )X K_3(X;\bar{D},\bar{W}), \end{aligned}$$

where it is easy to check that the function $K_3(X,\bar{D},\bar{W})$ is Lipschitz. Recalling (7.537), we conclude that

7.542 $$\begin{aligned} \frac{d}{dX}\bar{D} = K_2(X;\bar{D},\bar{W}), \ \ \frac{d}{dX}\bar{W} =(1+\eta ) K_3(X;\bar{D},\bar{W}). \end{aligned}$$

Therefore, the dynamical system (7.542) is regular at $X=0$ and must coincide with the RLP-solution emanating from the sonic point $X_*= x_*^{-\frac{1}{1+\eta }}$. Since the mapping $X\rightarrow Y$ is smooth and invertible locally around $X=0$, the claimed uniqueness statement follows. $\square$

### Maximal Extension

By analogy to Lemma 3.6, we factorise the denominator $\mathcal {C}$ into

7.543 $$\begin{aligned} \mathcal {C} = (1-\varepsilon )\left( \mathcal J[Y;d,\chi ] - \chi w\right) \left( \mathcal {H}[Y;d,\chi ] + \chi w\right) , \end{aligned}$$

where

7.544 $$\begin{aligned} \mathcal J[Y;d,\chi ]= \mathcal J&: = -\eta (1 +d)\chi + \sqrt{\eta ^2(1 +d)^2 \chi ^2+ \varepsilon \chi ^2 + \frac{\left( dY^{-2}\right) ^{-\eta }Y^2}{1-\varepsilon }}, \end{aligned}$$

7.545 $$\begin{aligned} \mathcal {H}[Y;d,\chi ]= \mathcal {H}&: = \mathcal J+ 2\eta (1+d)\chi . \end{aligned}$$

Clearly, for any fixed *Y* and $d$ , $\mathcal J[Y, d]$ is the solution of the equation $\mathcal {C}=0$ viewed as a quadratic equation in $\chi w$. Just like in the proof of Lemma 3.6, it can be checked that

7.546 $$\begin{aligned} \mathcal J'&= - \frac{4\varepsilon \chi \mathcal J+\eta (dY^{-2})^{-\eta -1}}{2(1-\varepsilon )\mathcal J+4\varepsilon \chi (1+d)}d' \nonumber \\&\ \ \ \ + \frac{-4\varepsilon \chi '(1+d)\mathcal J-2(\varepsilon ^2-\varepsilon )\chi \chi '+\frac{2\eta }{Y}(dY^{-2})^{-\eta -1}d+2(dY^{-2})^{-\eta }Y}{2(1-\varepsilon )\mathcal J+4\varepsilon \chi (1+d)} . \end{aligned}$$

Our next goal is to prove a *global extension* result, which is shown later in Theorem 2.5. To that end define

7.547 $$\begin{aligned} Y^{\text {ms}}: = \inf \left\{ Y<0\, \Big | \exists \text { a smooth solution to~(2.58)--(2.60) and} \ w(Y)>1, \ \mathcal {C}(Y)<0, \ \chi (Y)>0\right\} . \end{aligned}$$

#### Lemma 7.7

Let $(\chi ,w,d)$ be a local-in-*Y* solution to (2.58)–(2.60).

*(a)*: Then for all $Y\in (Y^{\text {ms}},0]$ the following identities hold:
  7.548 $$\begin{aligned} (d^{1+\eta }w)'&= - d^{1+\eta } \frac{(w+\varepsilon )(1-3w)}{(1-\varepsilon )Y}, \end{aligned}$$
  7.549 $$\begin{aligned} (\chi ^2d^{1+\eta }w)'&= \frac{\chi ^2 d^{1+\eta }}{(1-\varepsilon )Y}\left( w^2 +w(1+3\varepsilon )-\varepsilon \right) . \end{aligned}$$
*(b)*: For all $Y\in (Y^{\text {ms}},0)$ we have the bounds
  7.550 $$\begin{aligned} 0<d<w. \end{aligned}$$

#### Proof

*Proof of part (a).* Dividing (2.58) by $d$ and (2.59) by $w$ and the summing the $(1+\eta )$-multiple of the first equation with the second, we obtain (7.548). Using (7.548) and (2.60) we then obtain

$$\begin{aligned} (\chi ^2d^{1+\eta }w)'&= \frac{\chi ^2 d^{1+\eta }}{(1-\varepsilon )Y} \left( 2w(1-w) -(w+\varepsilon )(1-3w)\right) \\&= \frac{\chi ^2 d^{1+\eta }}{(1-\varepsilon )Y}\left( w^2 +w(1+3\varepsilon )-\varepsilon \right) . \end{aligned}$$

*Proof of part (b).* The strict positivity of $d$ in a small open left neighbourhood of $Y=0$ follows from Theorem 7.4. The global positivity then follows by integrating (2.58), which can be rewritten as

$$\begin{aligned} \left( \log d\right) ' = \frac{2\chi ^2}{Y} \frac{(w+\varepsilon )^2(d-w)}{\mathcal {C}}. \end{aligned}$$

The upper bound $d<w$ clearly holds at $Y=0$ and in its small neighbourhood due to (7.502)–(7.503). Assume now by contradiction that there exists $Y^{\text {ms}}<Y_*<0$ so that $Y_*$ is the infimum over all values of $Y\in (Y^{\text {ms}},0]$ such that $d(Y)<w(Y)$. By continuity obviously $d(Y_*)=w(Y_*)$. However, by (2.58)–(2.59) $d'(Y_*) - w'(Y_*) = \frac{(w+\varepsilon )(1-3w)}{(1-\varepsilon )Y}>0$, since $w(Y_*)>1$ by (7.547). This is a contradiction, as this means that $d-w$ decays locally going to the left of $Y_*$. $\square$

#### Lemma 7.8

Let $(\chi ,w,d)$ be a local-in-*Y* solution to (2.58)–(2.60) and let

7.551 $$\begin{aligned} \Gamma _\delta (Y) : = (Y^2)^{1+\eta } - (1-\delta )\chi ^2 wd^{1+\eta }, \ \ \delta >0. \end{aligned}$$

Then there exist a $0<\delta <1$ and $0<\varepsilon _0\ll 1$ sufficiently small such that for all $\varepsilon \in (0,\varepsilon _0]$ we have

7.552 $$\begin{aligned} \Gamma _\delta (Y)< 0, \ \ Y\in (Y^{\text {ms}},0). \end{aligned}$$

#### Proof

We observe that by (7.549),

7.553 $$\begin{aligned} \Gamma _\delta '(Y)&= (2+2\eta )(Y^2)^\eta Y - (1-\delta )(\chi ^2 wd^{1+\eta })' \nonumber \\&= (2+2\eta )(Y^2)^\eta Y- (1-\delta ) \frac{\chi ^2 d^{1+\eta }}{(1-\varepsilon )Y}\left( w^2 +w(1+3\varepsilon )-\varepsilon \right) \nonumber \\&= \frac{1}{(1-\varepsilon )Y} \left[ 2(1+\varepsilon )\Gamma _\delta (Y)+ (1-\delta )\chi ^2 d^{1+\eta }\left( -w^2+ w- \varepsilon w+ \varepsilon \right) \right] \nonumber \\&= \frac{1}{(1-\varepsilon )Y} \left[ 2(1+\varepsilon )\Gamma _\delta (Y)- (1-\delta )\chi ^2 d^{1+\eta }(w-1)(w+\varepsilon ) \right] . \end{aligned}$$

Since $w>1$ by our assumptions, it follows that

$$\begin{aligned} (1-\delta )\chi ^2 d^{1+\eta }(w-1)(w+\varepsilon )>0. \end{aligned}$$

Since $$\Gamma _\delta (Y) = (Y^2)^{1+\eta } \left( 1- (1-\delta )\chi ^2 w(dY^{-2})^{1+\eta }\right) $$ it follows from Theorem 7.4, inequality (7.533) that we can choose $$\delta =\delta (c_0)>0$$ such that $$\Gamma _\delta <0$$ on $$[Y_0,0)$$. Using the standard integrating factor argument it then follows from (7.553) that

7.554 $$\begin{aligned} \Gamma _\delta (Y)<0, \ \ Y\in (Y^{\text {ms}},0). \end{aligned}$$

$$\square $$

#### Lemma 7.9

Let $(\chi ,w,d)$ be a local-in-*Y* solution to (2.58)–(2.60) with the radius of analyticity $(Y_0,-Y_0)$ given by Theorem 7.4. Then there exists a constant $\tilde{\delta }>0$ such that

7.555 $$\begin{aligned} \frac{d}{w}>\tilde{\delta } \ \ \text { for all } \ Y^{\text {ms}}<Y\le Y_0, \ \ 0<\varepsilon \le \varepsilon _0. \end{aligned}$$

#### Proof

By (2.58)–(2.59) we obtain

7.556 $$\begin{aligned} d'w- dw'&= \frac{2(2+\eta )\chi ^2dw(w+\varepsilon )^2(d-w)}{Y\mathcal {C}} + \frac{d(w+\varepsilon )(1-3w)}{(1-\varepsilon )Y} \nonumber \\&= \frac{d}{(1-\varepsilon )Y\mathcal {C}} \Big \{4\chi ^2 w(w+\varepsilon )^2(d-w) \nonumber \\&\ \ \ \ +(w+\varepsilon )(1-3w)\left( \left( dY^{-2}\right) ^{-\eta }Y^2 - \chi ^2 \left[ (w+ \varepsilon )^2-\varepsilon (w-1)^2 + 4\varepsilon wd\right] \right) \Big \} \nonumber \\&= : \frac{d}{(1-\varepsilon )Y\mathcal {C}} \bar{A}, \end{aligned}$$

where we used $2(2+\eta ) = \frac{4}{1-\varepsilon }$. We now rewrite $\bar{A}$ to obtain

7.557 $$\begin{aligned} \bar{A}&= - 4\chi ^2 w^2 (w+\varepsilon )^2 - (w+\varepsilon )(1-3w)\chi ^2 (w+\varepsilon )^2 \nonumber \\&\ \ \ \ +4\chi ^2 wd(w+\varepsilon )^2 - 4\varepsilon \chi ^2 wd(w+\varepsilon )(1-3w)\nonumber \\&\ \ \ \ + (w+\varepsilon )(1-3w)\left( dY^{-2}\right) ^{-\eta }Y^2 + \varepsilon \chi ^2 (w+\varepsilon )(1-3w)(w-1)^2 \nonumber \\&= \chi ^2 (w+\varepsilon )^2 \left( -w^2-w-\varepsilon + 3\varepsilon w\right) + 4\chi ^2 w^2d(w+\varepsilon )(1+3\varepsilon ) \nonumber \\&\ \ \ \ + (w+\varepsilon )(1-3w)\left( dY^{-2}\right) ^{-\eta }Y^2 + \varepsilon \chi ^2 (w+\varepsilon )(1-3w)(w-1)^2 \nonumber \\&= \chi ^2 (w+\varepsilon ) \left\{ 4 w^2 d(1+3\varepsilon ) - (w+\varepsilon )\left( w^2+w+\varepsilon (1-3w)\right) \right\} \nonumber \\&\ \ \ \ + (w+\varepsilon )(1-3w)\left( dY^{-2}\right) ^{-\eta }Y^2 + \varepsilon \chi ^2 (w+\varepsilon )(1-3w)(w-1)^2 \nonumber \\&= \chi ^2 (w+\varepsilon ) \left\{ w^2 \left[ 4(1+3\varepsilon )d-w-\varepsilon \right] - (w+\varepsilon )\left( w+\varepsilon (1-3w)\right) \right\} \nonumber \\&\ \ \ \ + (w+\varepsilon )(1-3w)\left( dY^{-2}\right) ^{-\eta }Y^2 + \varepsilon \chi ^2 (w+\varepsilon )(1-3w)(w-1)^2 \nonumber \\&<\chi ^2 (w+\varepsilon ) w^2\left[ 4(1+3\varepsilon )d-w\right] , \end{aligned}$$

where we have used $w>1$, which in turn gives the bounds $-(w+\varepsilon )\left( w+\varepsilon (1-3w)\right) <0$, $(w+\varepsilon )(1-3w)\left( dY^{-2}\right) ^{-\eta }Y^2<0$, $\varepsilon \chi ^2 (w+\varepsilon )(1-3w)(w-1)^2<0$. Recall that $\frac{d}{w}\Big |_{Y=0}=0$ by (7.528)–(7.529). From (7.556) and (7.557) it then follows that

7.558 $$\begin{aligned} \left( \frac{d}{w}\right) '<0 \ \ \text { if } \ \frac{d}{w}<\frac{1}{4(1+3\varepsilon )}. \end{aligned}$$

Therefore by Theorem 7.4, inequality (7.534) and (7.558), the ratio $\frac{d}{w}$ increases to the left of $Y=Y_0$ as long as $\frac{d}{w}<\frac{1}{4(1+3\varepsilon )}$. If the ratio ever exceeds $\frac{1}{4(1+3\varepsilon )}$ going to the left then it must stay larger than $\frac{1}{4(1+3\varepsilon )}$ as seen by a contradiction argument using (7.557). Therefore the claim follows. $\square$

The next lemma establishes the monotonicity of the function $\chi w$, and as a consequence the strict lower bound $w>1+c$ on $(Y^{\text {ms}},0]$ for some $c>0$. The former is a preparatory step to prove that the flow remains supersonic in Lemma 7.11.

#### Lemma 7.10

Let $(\chi ,w,d)$ be a local-in-*Y* solution to (2.58)–(2.60) analytic in $(Y_0,-Y_0)$ given by Theorem 7.4. Then there exists an $0<\varepsilon _0\ll 1$ sufficiently small so that

7.559 $$\begin{aligned} (\chi w)'<0, \ \ Y^{\text {ms}}<Y\le Y_0. \end{aligned}$$

Moreover, there exists a constant $c>0$ such that

7.560 $$\begin{aligned} w(Y)>1+c, \ \ \ Y^{\text {ms}}<Y\le Y_0, \ \ 0<\varepsilon \le \varepsilon _0. \end{aligned}$$

In particular, $$\inf _{(Y^{\text {ms}},0]}w(Y)>1$$.

#### Proof

By (2.59)–(2.60) we have

7.561 $$\begin{aligned} (\chi w)'&= \frac{(1-w)\chi w}{(1-\varepsilon )Y} - \frac{\chi (w+\varepsilon )(1-3w)}{(1-\varepsilon )Y} - \frac{2(1+\eta )\chi ^3 w(w+\varepsilon )^2(d-w)}{Y\mathcal {C}} \nonumber \\&= \frac{2\chi w^2-\varepsilon \chi +3\varepsilon w\chi }{(1-\varepsilon )Y} - \frac{2(1+\eta )\chi ^3 w(w+\varepsilon )^2(d-w)}{Y\mathcal {C}} \nonumber \\&= \frac{\chi }{(1-\varepsilon )Y\mathcal {C}} \left\{ \left( 2w^2-\varepsilon +3\varepsilon w\right) \mathcal {C} - 2(1+\varepsilon ) \chi ^2 w(w+\varepsilon )^2(d-w)\right\} \nonumber \\&=: \frac{\chi }{(1-\varepsilon )Y\mathcal {C}}\, A. \end{aligned}$$

From (2.61) we have

7.562 $$\begin{aligned} A&= \left( 2w^2-\varepsilon +3\varepsilon w\right) \left( \left( dY^{-2}\right) ^{-\eta }Y^2 - \chi ^2 \left[ (w+ \varepsilon )^2-\varepsilon (w-1)^2 + 4\varepsilon wd\right] \right) \nonumber \\&\ \ \ \ - 2(1+\varepsilon ) \chi ^2 wd(w+\varepsilon )^2 + 2(1+\varepsilon ) \chi ^2 w^2(w+\varepsilon )^2 \nonumber \\&= \chi ^2(w+\varepsilon )^2 \left[ -2w^2-3\varepsilon w+\varepsilon + 2(1+\varepsilon )w^2\right] + \varepsilon \chi ^2 \left( 2w^2-\varepsilon +3\varepsilon w\right) (w-1)^2 \nonumber \\&\ \ \ \ - 4\varepsilon \chi ^2 wd\left( 2w^2-\varepsilon +3\varepsilon w\right) - 2(1+\varepsilon ) \chi ^2 wd(w+ \varepsilon )^2\nonumber \\&\quad + \left( 2w^2-\varepsilon +3\varepsilon w\right) \left( dY^{-2}\right) ^{-\eta }Y^2 \nonumber \\&= \varepsilon \chi ^2(w+\varepsilon )^2 (w-1)(2w-1) + \varepsilon \chi ^2 \left( 2w^2-\varepsilon +3\varepsilon w\right) (w-1)^2 \nonumber \\&\ \ \ \ - 4\varepsilon \chi ^2 wd\left( 2w^2-\varepsilon +3\varepsilon w\right) - 2(1+\varepsilon ) \chi ^2 wd(w+ \varepsilon )^2\nonumber \\&\quad + \left( 2w^2-\varepsilon +3\varepsilon w\right) \left( dY^{-2}\right) ^{-\eta }Y^2 \end{aligned}$$

By Lemma 7.8 there exists a $\delta >0$ such that $\left( dY^{-2}\right) ^{-\eta }Y^2 < (1-\delta ) \chi ^2 wd$. Therefore

7.563 $$\begin{aligned}&- 4\varepsilon \chi ^2 wd\left( 2w^2-\varepsilon +3\varepsilon w\right) - 2(1+\varepsilon ) \chi ^2 wd(w+ \varepsilon )^2 + \left( 2w^2-\varepsilon +3\varepsilon w\right) \left( dY^{-2}\right) ^{-\eta }Y^2 \nonumber \\&< \chi ^2 wd\left( -4\varepsilon \left( 2w^2-\varepsilon +3\varepsilon w\right) - 2(1+\varepsilon ) (w+ \varepsilon )^2 +\left( 2w^2-\varepsilon +3\varepsilon w\right) (1-\delta ) \right) \nonumber \\&= \chi ^2 wd\left\{ (-2\delta -10\varepsilon )w^2 + \left( -12\varepsilon ^2-4\varepsilon (1+\varepsilon )+3\varepsilon (1-\delta )\right) w\right. \nonumber \\&\quad \left. + 4\varepsilon ^2-2\varepsilon ^2(1+\varepsilon )-\varepsilon (1-\delta )\right\} \nonumber \\&< -2\delta \chi ^2 w^3 d, \end{aligned}$$

for $\varepsilon \le \varepsilon _0$ sufficiently small. Therefore

7.564 $$\begin{aligned} A&< \varepsilon \chi ^2(w+\varepsilon )^2 (w-1)(2w-1) + \varepsilon \chi ^2 \left( 2w^2-\varepsilon +3\varepsilon w\right) (w-1)^2 -2\delta \chi ^2 w^3 d\nonumber \\&= \varepsilon \chi ^2 (w-1)\left( 4w^3 + (-3+7\varepsilon )w^2 + (2\varepsilon ^2-6\varepsilon )w+ \varepsilon -\varepsilon ^2\right) -2\delta \chi ^2 w^3 d\nonumber \\&< 4\varepsilon \chi ^2 w^4 -2\delta \chi ^2 w^3 d\nonumber \\&= 2\chi ^2 w^3\left( -\delta d+ 2\varepsilon w\right) , \end{aligned}$$

where we have used the bounds $w-1<w$ and $(-3+7\varepsilon )w^2 + (2\varepsilon ^2-6\varepsilon )w+ \varepsilon -\varepsilon ^2<0$, where the latter follows from the assumption $w>1$ on $(Y^{\text {ms}},0]$. Plugging (7.564) into (7.561) we obtain the estimate

7.565 $$\begin{aligned} (\chi w)'&< \frac{2\chi ^3 w^3 }{(1-\varepsilon )Y\mathcal {C}}\left( -\delta d+ 2\varepsilon w\right) = \frac{2\chi ^3 w^4 }{(1-\varepsilon )Y\mathcal {C}}\left( -\delta \frac{d}{w} + 2\varepsilon \right) \nonumber \\&< \frac{2\chi ^3 w^4 }{(1-\varepsilon )Y\mathcal {C}}\left( -\delta \tilde{\delta } + 2\varepsilon \right)< -\frac{\delta \tilde{\delta }\chi ^3 w^4 }{(1-\varepsilon )Y\mathcal {C}}<0, \end{aligned}$$

for $0<\varepsilon \le \varepsilon _0$ sufficiently small. Here we have crucially used Lemma 7.9.

To prove (7.560) we note that by (7.565)

7.566 $$\begin{aligned} \chi (Y)w(Y) > \chi (Y_0)w(Y_0), \ \ Y^{\text {ms}}<Y<Y_0 \end{aligned}$$

and therefore

7.567 $$\begin{aligned} w(Y)> \frac{\chi (Y_0)}{\chi (Y)}w(Y_0)\ge w(Y_0)>1+ c_{w}, \end{aligned}$$

where we have used part (d) of Theorem 7.4 in the last inequality. We have also used the bound $\chi (Y)\le \chi (Y_0)$ for $Y\le Y_0$ which follows from $\chi '>0$ on $(Y^{\text {ms}}, Y_0)$, which in turn follows from the bound $w>1$ and (2.60). $\square$

#### Lemma 7.11

Let $(\chi ,w,d)$ be a local-in-*Y* solution to (2.58)–(2.60) with the radius of analyticity $(Y_0,-Y_0)$ given by Theorem 7.4. Then

$$\begin{aligned} \limsup _{Y\rightarrow (Y^{\text {ms}})^-}\mathcal \, \mathcal {C}(Y)<0, \end{aligned}$$

in other words - the flow remains supersonic.

#### Proof

Using (7.546) and (7.559), and the bounds

$$\begin{aligned} - \frac{4\varepsilon \chi \mathcal J+\eta (dY^{-2})^{-\eta -1}}{2(1-\varepsilon )\mathcal J+4\varepsilon \chi (1+d)}d'>0, \ \ -2(\varepsilon ^2-\varepsilon )\chi \chi '>0, \end{aligned}$$

we get

7.568 $$\begin{aligned} \mathcal J' - (\chi w)' > \frac{\delta \tilde{\delta }\chi ^3 w^4 }{(1-\varepsilon )Y\mathcal {C}} + \frac{ -4\varepsilon \chi '(1+d)\mathcal J+(2\eta +2)(dY^{-2})^{-\eta } Y}{2(1-\varepsilon )\mathcal J+4\varepsilon \chi (1+d)}. \end{aligned}$$

Since $(dY^{-2})^{-\eta }Y^2 = (1-\varepsilon )\mathcal J^2 + 4\varepsilon \mathcal J(1+d)\chi - \varepsilon (1-\varepsilon ) \chi ^2$, it follows that

$$\begin{aligned} (dY^{-2})^{-\eta }Y^2< (1-\varepsilon )\mathcal J^2+ 4\varepsilon \mathcal J(1+d)\chi < \mathcal J\left( 2(1-\varepsilon )\mathcal J+ 4\varepsilon (1+d)\chi \right) . \end{aligned}$$

Therefore

7.569 $$\begin{aligned} \left| \frac{(2\eta +2)(dY^{-2})^{-\eta } Y}{2(1-\varepsilon )\mathcal J+4\varepsilon \chi (1+d)} \right| \le \frac{C\mathcal J}{|Y|}. \end{aligned}$$

From (2.60) and the bound $w>1$ we have the rough bound $|\chi '|\le C\frac{w\chi }{|Y|}$. Therefore

7.570 $$\begin{aligned} \left| \frac{-4\varepsilon \chi '(1+d)\mathcal J}{2(1-\varepsilon )\mathcal J+4\varepsilon \chi (1+d)} \right| \le \frac{C\varepsilon w\chi (1+d)\mathcal J}{|Y|(2(1-\varepsilon )\mathcal J+4\varepsilon \chi (1+d))} \le \frac{C w\mathcal J}{|Y|}. \end{aligned}$$

Using (7.569)–(7.570) in (7.568) we obtain

7.571 $$\begin{aligned} \mathcal J' - (\chi w)' > \frac{\delta \tilde{\delta }\chi ^3 w^4 }{(1-\varepsilon )Y\mathcal {C}} - C\frac{\mathcal J(1+ w)}{|Y|} = \chi w^2\left( \frac{\delta \tilde{\delta }\chi ^2w^2 }{(1-\varepsilon )Y\mathcal {C}}- C\frac{\frac{\mathcal J}{\chi w}(\frac{1}{w}+1)}{|Y|}\right) . \end{aligned}$$

Assume now that $\lim _{Y\rightarrow (Y^{\text {ms}})^-} (\mathcal J- \chi w)=0$. In that case $\lim _{Y\rightarrow (Y^{\text {ms}})^-}\mathcal {C} = 0$ and it is clear from (7.571) that $\mathcal J' - (\chi w)'$ is strictly positive in some right neighbourhood of $Y^{\text {ms}}$. Here we use the uniform positivity of $\chi w$ on $(Y^{\text {ms}},0]$, which follows from Lemma 7.10. A contradiction. $\square$

We have shown that the flow remains supersonic to the left of $Y=0$ and therefore the only obstruction to the global existence of the solution is the finite-time blow-up of the unknowns. We shall show that this is precisely the case. The intuition is that right-hand side of (2.59) will be, in a suitable sense, dominated by the first term on the right-hand side, which will lead to the blow-up of $w$ through a Riccati-type argument.

*Proof of Theorem* 2.5. Let $\alpha >0$ be a positive constant to be specified later. We rewrite (2.59) in the form

7.572 $$\begin{aligned} w' = -\frac{(w+\varepsilon )(1-(3-2\alpha )w)}{(1-\varepsilon )Y} +\frac{2(w+\varepsilon )}{(1-\varepsilon )Y\mathcal {C}}\left( \alpha w\mathcal {C}-(1+\varepsilon )\chi ^2w(w+\varepsilon )(d-w)\right) . \end{aligned}$$

We focus on the term

$$\begin{aligned} E: = \alpha w\mathcal {C}-(1+\varepsilon )\chi ^2w(w+\varepsilon )(d-w). \end{aligned}$$

By (2.61) we have

7.573 $$\begin{aligned} E&= \alpha wd^{-\eta }(Y^2)^{1+\eta } -\alpha w(1-\varepsilon )\chi ^2w^2 -4\varepsilon \alpha w^2 \chi ^2 \nonumber \\&\ \ \ \ + \alpha w\chi ^2(\varepsilon -\varepsilon ^2) - 4\varepsilon \alpha \chi ^2 w^2 d\nonumber \\&-(1+\varepsilon )\chi ^2 wd(w+\varepsilon ) +(1+\varepsilon )\chi ^2 w^2 (w+\varepsilon ). \end{aligned}$$

We let

7.574 $$\begin{aligned} \alpha = \frac{1+\bar{\delta }}{1-\varepsilon } \end{aligned}$$

where $\bar{\delta }>0$ is a constant to be specified later. After regrouping terms in *E* above we obtain

7.575 $$\begin{aligned} E&= \chi ^2 w^2\left( -(1+\bar{\delta }) w- 2\eta (1+\bar{\delta })+ (1+\varepsilon )(w+\varepsilon )+\frac{\varepsilon (1+\bar{\delta })}{w}\right) \nonumber \\&\ \ \ \ + \frac{1+\bar{\delta }}{1-\varepsilon } wd^{-\eta }(Y^2)^{1+\eta }- 2\eta (1+\bar{\delta }) \chi ^2 w^2 d-(1+\varepsilon )\chi ^2 wd(w+\varepsilon ) \nonumber \\&< \chi ^2 w^2\left( - (\bar{\delta }-\varepsilon ) w- 2\eta (1+\bar{\delta }) + \varepsilon + \varepsilon ^2+ \frac{\varepsilon (1+\bar{\delta })}{w} \right) \nonumber \\&\ \ \ \ + \chi ^2 w^2 d\left( \frac{1+\bar{\delta }}{1-\varepsilon }(1-\delta ) - 2\eta (1+\bar{\delta })-(1+\varepsilon ) \right) , \end{aligned}$$

where we have used Lemma 7.8 in the last inequality. It is now clear that with the choice

7.576 $$\begin{aligned} \bar{\delta } \le \frac{1}{2}\delta , \end{aligned}$$

there exists an $0<\varepsilon _0\ll 1$ sufficiently small, so that both expressions on the right-most side of (7.575) are strictly negative for all $0<\varepsilon \le \varepsilon _0$. Here we use $w>1$.

We conclude therefore from (7.572) and (7.574) that

7.577 $$\begin{aligned} w' \le -\frac{(w+\varepsilon )(1-\frac{1-3\varepsilon -2\bar{\delta }}{1-\varepsilon }w)}{(1-\varepsilon )Y} = -\frac{1-3\varepsilon -2\bar{\delta }}{1-\varepsilon } \ \frac{(w+\varepsilon )(\frac{1-\varepsilon }{1-3\varepsilon -2\bar{\delta }} - w)}{(1-\varepsilon )Y}. \end{aligned}$$

Now choose $\bar{\delta }$ sufficiently small (but independent of $\varepsilon$) so that $w(Y)>\frac{1-\varepsilon }{1-3\varepsilon -2\bar{\delta }}$ for all $Y\in (Y^{\text {ms}},Y_0)$, which is possible due to (7.560). Let now

$$\begin{aligned} K: = \frac{1-3\varepsilon -2\bar{\delta }}{1-\varepsilon }. \end{aligned}$$

We conclude from (7.577) that

7.578 $$\begin{aligned} w' \le - C \frac{(w+\varepsilon )(Kw-1)}{|Y|} \le - a \frac{w(Kw-1)}{|Y|} \ , \ \ Y^{\text {ms}}< Y < Y_0, \end{aligned}$$

for some universal constant $a>0$. Inequality (7.578) is a Riccati-type differential inequality and leads to finite *Y* blow-up of $w$. We provide here the standard argument for the sake of completeness. Upon multiplying $w$ by *K* and then redefining $a>0$, we may assume without loss of generality that $K=1$. Divide (7.578) (with $K=1$) by $w(w-1)$ and express both sides as an exact derivative to conclude

7.579 $$\begin{aligned} \log \left( \left( 1-\frac{1}{w}\right) |Y|^{-a}\right) ' \le 0, \ \ Y\le Y_0. \end{aligned}$$

Upon integration this yields the bound

7.580 $$\begin{aligned} w\ge \frac{1}{1 - C{|Y|^a}}, \ \ Y<Y_0, \end{aligned}$$

and therefore $w$ necessarily blows up as $Y\rightarrow \tilde{Y}^+$ for some $-\infty<\tilde{Y}<Y_0<0$. By Lemma 7.9 this also implies that *d* blows up as $Y\rightarrow \tilde{Y}^+$.

We next want to show that $\lim _{Y\rightarrow \tilde{Y}^+}\chi (Y)=0$ and therefore $\tilde{Y} = Y^{\text {ms}}$. We note that for any $Y\in (\tilde{Y},0]$ we necessarily have $\chi (Y)>0$, which follows from (2.60). Since $\chi '>0$ by (2.60) and the bound $w>1$ it follows that $\chi$ decreases to the left of $Y=0$ and the limit

7.581 $$\begin{aligned} \chi _*: = \lim _{Y\rightarrow \tilde{Y}^+}\chi (Y)\ge 0 \end{aligned}$$

exists. Assume by the way of contradiction that $\chi _*>0$. We look more closely at the leading order behaviour of the right-hand side of (2.59) on approach to the blow-up point $\tilde{Y}$. Since

7.582 $$\begin{aligned} 0<\tilde{\delta }< \frac{d}{w} <1 \end{aligned}$$

by Lemmas 7.7 and 7.9, and the assumption $\chi _*>0$ it is easily seen that

7.583 $$\begin{aligned} w' = \frac{3w^2}{(1-\varepsilon )Y} + \frac{2(1+\varepsilon )w^3(d-w)}{Y\left( (1-\varepsilon )w^2+4\varepsilon wd\right) } + \mathcal {R}, \end{aligned}$$

where $\mathcal {R}$ has the property

$$\begin{aligned} \lim _{Y\rightarrow \tilde{Y}^+}\frac{\mathcal {R}(Y)}{ \frac{2(1+\varepsilon )w^3(d-w)}{Y\left( (1-\varepsilon )w^2+4\varepsilon wd\right) } } = 0. \end{aligned}$$

From (7.582) and (7.583) it is now clear that there exist constants $0<\kappa _1<\kappa _2$ such that

7.584 $$\begin{aligned} \frac{\kappa _1}{Y}< \frac{w'}{w^2} < \frac{\kappa _2}{Y}, \ \ Y\in (\tilde{Y}, \tilde{Y}+\beta ), \end{aligned}$$

for some constant $\beta >0$. Integrating the above differential inequalities over an interval $[Y_0,Y]\subset (\tilde{Y}, \tilde{Y}+\beta )$ and letting $Y_0\rightarrow \tilde{Y}$, we conclude

7.585 $$\begin{aligned} \frac{1}{\kappa _2 |\tilde{Y}-Y| + O(|\tilde{Y}-Y|^2)} \ge w(Y) \ge \frac{1}{\kappa _1 |\tilde{Y}-Y| + O(|\tilde{Y}-Y|^2)} \end{aligned}$$

in a possibly smaller open right neighbourhood of $\tilde{Y}$. By (2.60) we have $(\log \chi )'=\frac{1-w}{Y}$, which together with (7.585) shows that there exist some positive constants $0<\tilde{\kappa }_1<\tilde{\kappa }_2$ such that

7.586 $$\begin{aligned} \tilde{\kappa }_1< \left( \log \chi \right) '|\tilde{Y}-Y| < \tilde{\kappa }_2 \end{aligned}$$

in a small open right neighbourhood of $\tilde{Y}$. Integrating (7.586) we conclude that

7.587 $$\begin{aligned} \lim _{Y\rightarrow \tilde{Y}^+}\chi (Y) = 0, \end{aligned}$$

which contradicts the assumption $\chi _*>0$. It follows that in particular $\tilde{Y} = Y^{\text {ms}}$. $\square$

#### Corollary 7.12

(Uniformity-in-$\varepsilon$) There exist a constant $A>0$ and $0<\varepsilon _0\ll 1$ such that for all $\varepsilon \in (0,\varepsilon _0]$ we have the uniform bounds

7.588 $$\begin{aligned} \frac{1}{A}< |Y^{\text {ms}}| < A, \end{aligned}$$

where $-\infty<Y^{\text {ms}}<0$ is the maximal existence interval to the left from Theorem 2.5.

#### Proof

Both constants *C* and *a* in (7.580) can be chosen to be $\varepsilon$-independent for $\varepsilon _0$ sufficiently small and so we obtain a uniform upper bound on the maximal time $Y^{\text {ms}}$. Since by the construction $Y^{\text {ms}}<Y_0<0$, where $Y_0$ is the $\varepsilon$-independent constant from Theorem 7.4, we conclude the proof. $\square$

### The Massive Singularity

#### Definition 7.13

(The massive singularity) The hypersurface $\mathcal {M}\mathcal {S}_\varepsilon$ defined through

$$\begin{aligned} \mathcal {M}\mathcal {S}_\varepsilon&= \left\{ (\tilde{\tau }, R) \, \Big | \, R = \frac{\sqrt{\varepsilon }}{|Y^{\text {ms}}|} \tilde{\tau }\right\} \setminus \{(0,0)\} \end{aligned}$$

is called the massive singularity.

In this section we compute the precise blow up rates of the RLP-solution at the massive singularity. To this end, it is convenient to introduce the quantity

7.589 $$\begin{aligned} \bar{\mathcal {C}} : = -\chi ^{-2}w^{-2} \mathcal {C} = -\chi ^{-2}w^{-2} \left( dY^{-2}\right) ^{-\eta }Y^2 + \left[ (1 + \frac{\varepsilon }{w})^2-\varepsilon (1-\frac{1}{w})^2 + 4\varepsilon \frac{d}{w} \right] . \end{aligned}$$

#### Lemma 7.14

The limits $Q_0{:}{=}\lim _{Y\rightarrow Y^{\text {ms}}}\frac{d(Y)}{w(Y)}$ and $\lim _{Y\rightarrow Y^{\text {ms}}}\bar{\mathcal {C}}(Y)$ exist and are finite. Moreover $0< Q_0\le 1$ and

$$\begin{aligned} \lim _{Y\rightarrow Y^{\text {ms}}}\bar{\mathcal {C}}(Y)=1 - \varepsilon + 4\varepsilon Q_0. \end{aligned}$$

#### Proof

We let

7.590 $$\begin{aligned} Q{:}{=}\frac{d}{w}. \end{aligned}$$

We use (7.556)–(7.557) to derive a differential equation for *Q*:

7.591 $$\begin{aligned} Q'&= \frac{dw(w+\varepsilon )}{(1-\varepsilon )|Y|w^2\bar{\mathcal {C}}} \left[ (1+3\varepsilon )(4Q-1) - \frac{1}{w} \alpha (Y)\right] + \frac{d^{1-\eta }(w+\varepsilon )(1-3w)|Y|^{2+2\eta }}{(1-\varepsilon )|Y|\chi ^2 w^4 \bar{\mathcal {C}}} \nonumber \\&{=}{:} \alpha _1(Y) + \alpha _2(Y)d^{1-\eta }, \end{aligned}$$

where the function $\alpha (Y)$ is given by

7.592 $$\begin{aligned} \alpha (Y) = 1-9\varepsilon +\frac{7\varepsilon -3\varepsilon ^2}{w} - \frac{\varepsilon -\varepsilon ^2}{w^2}. \end{aligned}$$

By the invariance of the flow, we know that $(1+3\varepsilon )(4Q-1)\ge \frac{1}{w}\alpha (Y)$ and in particular the function $\alpha _1$ is nonnegative on $(Y^{\text {ms}},Y_0]$. Integrating (7.591) we conclude that

7.593 $$\begin{aligned} Q(Y)-Q(Y^{\text {ms}}) = \int _{Y^{\text {ms}}}^Y \alpha _1(s)\,ds + \int _{Y^{\text {ms}}}^Y d^{1-\eta }\alpha _2(s)\,ds. \end{aligned}$$

We observe that $\alpha _2$ is a bounded function on $(Y^{\text {ms}},Y_0]$ and $d^{1-\eta }$ is bounded from above by $\kappa ^{1-\eta }(\delta Y)^{-1+\eta }$ and therefore $\left| \int _{Y^{\text {ms}}}^Y d^{1-\eta }\alpha _2(s)\,ds \right| \lesssim \frac{1}{\varepsilon }|Y-Y^{\text {ms}}|^{\eta }$. Since *Q* is bounded, it follows from (7.593) that $\alpha _1\in L^1((Y^{\text {ms}},Y_0])$. For any $Y_1,Y_2\in (Y^{\text {ms}},Y_0]$ we conclude that

$$\begin{aligned} Q(Y_1)-Q(Y_2) = \int _{Y_1}^{Y_2} \alpha _1(s)\,ds + \int _{Y_1}^{Y_2} d^{1-\eta }\alpha _2(s)\,ds \lesssim \int _{Y_1}^{Y_2} \alpha _1(s)\,ds + \frac{1}{\varepsilon }|Y-Y^{\text {ms}}|^{\eta } \end{aligned}$$

In particular, *Q* is uniformly continuous on $(Y^{\text {ms}},Y_0]$ and therefore $Q_0=\lim _{Y\rightarrow Y^{\text {ms}}}Q(Y)$ exists as claimed. Since $0<\frac{d}{w}\le 1$ by Lemma 7.9 we have $Q_0\in (0,1]$. To show that $\lim _{Y\rightarrow Y^{\text {ms}}}\bar{\mathcal {C}}(Y)$ exists it follows from (7.585) and the continuity of *Q* that we only need to show the continuity of $\chi ^{-2}w^{-2} \left( dY^{-2}\right) ^{-\eta }Y^2$ at $Y^{\text {ms}}$. However, by Lemma 7.10 the quantity $\chi ^{-2}w^{-2}$ is uniformly bounded from above on approach to $Y^{\text {ms}}$ and by (7.585) $\left( dY^{-2}\right) ^{-\eta }Y^2 \lesssim |Y-Y^{\text {ms}}|^\eta$ as $Y\rightarrow Y^{\text {ms}}$, so that $\chi ^{-2}w^{-2} \left( dY^{-2}\right) ^{-\eta }Y^2$ necessarily converges to 0 as $Y\rightarrow Y^{\text {ms}}$. Therefore $Y\mapsto \bar{\mathcal {C}}(Y)$ is continuous and it converges to $1 - \varepsilon + 4\varepsilon Q_0$ at $Y^{\text {ms}}$. $\square$

#### Lemma 7.15

There exist positive constants $\hat{w}, \hat{d}>0$ such that

7.594 $$\begin{aligned} w(Y)&= \frac{\hat{w}}{|Y-Y^{\text {ms}}|} + o_{Y\rightarrow Y^{\text {ms}}}(|Y-Y^{\text {ms}}|^{-1}), \end{aligned}$$

7.595 $$\begin{aligned} d(Y)&= \frac{\hat{d}}{|Y-Y^{\text {ms}}|} + o_{Y\rightarrow Y^{\text {ms}}}(|Y-Y^{\text {ms}}|^{-1}). \end{aligned}$$

Moreover

7.596 $$\begin{aligned} \hat{d}= \frac{|Y^{\text {ms}}|}{6}, \ \ \hat{w}= \frac{2|Y^{\text {ms}}|}{3}. \end{aligned}$$

#### Proof

Dividing (2.58) by $d^2$ and using (7.589) we obtain

7.597 $$\begin{aligned} -\frac{d'}{d^2} =\frac{2}{|Y|} \frac{ (1+\frac{\varepsilon }{w})^2(\frac{w}{d}-1)}{\bar{\mathcal {C}}}. \end{aligned}$$

The right-hand side has a strictly positive limit $\frac{1}{\hat{d}}$ by Lemma 7.14, where $\hat{d}$ equals

7.598 $$\begin{aligned} \hat{d}= \frac{|Y^{\text {ms}}|}{2} \frac{Q_0(1+\varepsilon \left( 4 Q_0-1\right) )}{1-Q_0}, \end{aligned}$$

and we may write the limit in the form $\frac{1}{\hat{d}} +o_{Y\rightarrow Y^{\text {ms}}}(|Y-Y^{\text {ms}}|)$. We may now integrate (7.597) to conclude (7.595). Analogously, we divide (2.59) by $w^2$ and obtain

7.599 $$\begin{aligned} -\frac{w'}{w^2} =\frac{\left( 1+\frac{\varepsilon }{w}\right) \left( \frac{1}{w}-3\right) }{(1-\varepsilon )Y} - \frac{2(1+\eta )}{Y} \frac{ (1+\frac{\varepsilon }{w})^2(\frac{d}{w}-1)}{\bar{\mathcal {C}}}. \end{aligned}$$

By Lemma 7.14, the right-hand side converges to a limit denoted by $\frac{1}{\hat{w}}$, given by

7.600 $$\begin{aligned} \frac{1}{\hat{w}} = \frac{3}{(1-\varepsilon )|Y^{\text {ms}}|} - \frac{2(1+\eta )}{|Y^{\text {ms}}|} \frac{1- Q_0}{1+\varepsilon (4Q_0-1)}. \end{aligned}$$

We may now integrate (7.599) to conclude (7.594). Since by (7.594) and (7.595) $Q_0=\frac{\hat{d}}{\hat{w}}$, it follows by multiplying (7.598) and (7.600) that

7.601 $$\begin{aligned} Q_0 = \frac{|Y^{\text {ms}}|}{2} \frac{Q_0(1+\varepsilon \left( 4 Q_0-1\right) )}{1-Q_0} \left( \frac{3}{(1-\varepsilon )|Y^{\text {ms}}|} - \frac{2(1+\eta )}{|Y^{\text {ms}}|} \frac{1- Q_0}{1+\varepsilon (4Q_0-1)}\right) . \end{aligned}$$

Since $Q_0>0$ by Lemma 7.14, we may divide by $Q_0$ above and reduce the problem to the linear equation $(4Q_0-1)\left( 1+\varepsilon \right) =0$, hence $\frac{\hat{d}}{\hat{w}}=Q_0=\frac{1}{4}$. From (7.598) and (7.600) we now conclude (7.596). $\square$

#### Proposition 7.16

(Massive singularity) Let $(\hat{w},\hat{d})$ be given by (7.596). There exists a $\hat{\chi }>0$ such that on approach to the massive singularity $\mathcal {M}\mathcal {S}_\varepsilon$ the solution $(d,w,\chi )$ of (2.58)–(2.60) obeys the following asymptotic behaviour:

7.602 $$\begin{aligned} w(Y)&= \frac{\hat{w}}{|Y-Y^{\text {ms}}|} \left( 1 + O_{Y\rightarrow Y^{\text {ms}}}(|Y-Y^{\text {ms}}|^{\eta })\right) , \end{aligned}$$

7.603 $$\begin{aligned} d(Y)&= \frac{\hat{d}}{|Y-Y^{\text {ms}}|} \left( 1 + O_{Y\rightarrow Y^{\text {ms}}}(|Y-Y^{\text {ms}}|^{\eta })\right) , \end{aligned}$$

7.604 $$\begin{aligned} \chi (Y)&= \hat{\chi }|Y-Y^{\text {ms}}|^{\frac{2}{3(1-\varepsilon )}}\left( 1 + O_{Y\rightarrow Y^{\text {ms}}}(|Y-Y^{\text {ms}}|^{\eta })\right) . \end{aligned}$$

Moreover, the quantities $\tilde{\mu }$, $\tilde{\lambda }$, defined by the extension of (2.68) and (3.107) to $Y\in (Y^{\text {ms}},0)$ respectively, satisfy

7.605 $$\begin{aligned} e^{2\tilde{\mu }} \asymp _{Y\rightarrow Y^{\text {ms}}} (Y-Y^{\text {ms}})^{\frac{2\varepsilon }{1-\varepsilon }}, \ \ e^{2\tilde{\lambda }}\asymp _{Y\rightarrow Y^{\text {ms}}} (Y-Y^{\text {ms}})^{-\frac{2}{3(1-\varepsilon )}}. \end{aligned}$$

The star density $\rho$ and the Ricci scalar $\mathcal {R}$ blow up on approach to $\mathcal {M}\mathcal {S}_\varepsilon$.

#### Proof

We introduce $q{:}{=}Q-\frac{1}{4}=\frac{d}{w}-\frac{1}{4}$, we can rewrite (7.591) in the form

7.606 $$\begin{aligned} q' = \frac{A}{Y-Y^{\text {ms}}} q + B, \end{aligned}$$

where

$$\begin{aligned}{} & {} A: = \frac{4(1+3\varepsilon )d|Y-Y^{\text {ms}}| w(w+\varepsilon )}{(1-\varepsilon )|Y|w^2\bar{\mathcal {C}}}, \\{} & {} B: = - \frac{Q (w+\varepsilon )}{(1-\varepsilon )|Y|w\bar{\mathcal {C}}}\alpha + \frac{d^{1-\eta }(w+\varepsilon )(1-3w)|Y|^{2+2\eta }}{(1-\varepsilon )|Y|\chi ^2 w^4 \bar{\mathcal {C}}}. \end{aligned}$$

From (7.585) and Lemma 7.15 we immediately have

7.607 $$\begin{aligned} \lim _{Y\rightarrow Y^{\text {ms}}} A(Y)&= \frac{4(1+3\varepsilon )\hat{d}}{(1-\varepsilon )|Y^{\text {ms}}|} = \frac{2}{3} (1+2\eta )=A_0, \end{aligned}$$

7.608 $$\begin{aligned} B(Y)&= O_{Y\rightarrow Y^{\text {ms}}}(|Y-Y^{\text {ms}}|^{-1+\eta }). \end{aligned}$$

We now consider $Y^{\text {ms}}<Y<Y_1$ for some fixed $Y_1$ and integrate (7.606). We obtain

7.609 $$\begin{aligned} q(Y) = q(Y_1)e^{\int _{Y_1}^Y\frac{A(\tau )}{\tau -Y^{\text {ms}}}\,d\tau } + \int _{Y_1}^Y e^{\int _{s}^Y\frac{A(\tau )}{\tau -Y^{\text {ms}}}\,d\tau } B(s)\,ds. \end{aligned}$$

For any $0<\delta \ll 1$ there exists a $Y_1>Y^{\text {ms}}$ such that $|A-A_0|<\delta$. It is then easy to see that

$$\begin{aligned} e^{\int _{Y_1}^Y\frac{A(\tau )}{\tau -Y^{\text {ms}}}\,d\tau } = e^{-\int _{Y}^{Y_1}\frac{A(\tau )}{\tau -Y^{\text {ms}}}\,d\tau } \le e^{-\int _{Y}^{Y_1}\frac{A_0-\delta }{\tau -Y^{\text {ms}}}\,d\tau } = \frac{|Y-Y^{\text {ms}}|^{A_0-\delta }}{|Y_1-Y^{\text {ms}}|^{A_0-\delta }}. \end{aligned}$$

We use the bound in (7.609) and conclude

7.610 $$\begin{aligned} \left| q(Y) \right|&\lesssim \left| q(Y_1)\right| \frac{|Y-Y^{\text {ms}}|^{A_0-\delta }}{|Y_1-Y^{\text {ms}}|^{A_0-\delta }} + \int _{Y_1}^Y \frac{|Y-Y^{\text {ms}}|^{A_0-\delta }}{|s-Y^{\text {ms}}|^{A_0-\delta }} |s-Y^{\text {ms}}|^{-1+\eta }\,ds \nonumber \\&\lesssim |Y-Y^{\text {ms}}|^{A_0-\delta } + |Y-Y^{\text {ms}}|^{\eta } \lesssim |Y-Y^{\text {ms}}|^{\eta }. \end{aligned}$$

Plugging this back into (7.597) and (7.599) allows us to obtain the (suboptimal) rates (7.602)–(7.603). Upon dividing (2.60) by $\chi$ and integrating, using (7.602), for any $Y^{\text {ms}}<Y<Y_1$ we obtain

7.611 $$\begin{aligned} \chi (Y)&= \chi (Y_1) e^{\frac{1}{1-\varepsilon }\int _{Y}^{Y_1} \frac{\hat{w}}{\tau (\tau -Y^{\text {ms}})}\left( 1 + O_{\tau \rightarrow Y^{\text {ms}}}(|\tau -Y^{\text {ms}}|^{\eta })\right) \, d\tau } \nonumber \\&= \chi (Y_1) e^{\frac{2}{3(1-\varepsilon )}\int _{Y}^{Y_1} \frac{|Y^{\text {ms}}|}{\tau (\tau -Y^{\text {ms}})}\left( 1 + O_{Y\rightarrow Y^{\text {ms}}}(|Y-Y^{\text {ms}}|^{\eta })\right) \, d\tau } \nonumber \\&= \chi (Y_1) e^{\frac{2}{3(1-\varepsilon )}\int _{Y}^{Y_1} \left( \frac{1}{\tau }-\frac{1}{ \tau -Y^{\text {ms}}}\right) \left( 1 + O_{Y\rightarrow Y^{\text {ms}}}(|Y-Y^{\text {ms}}|^{\eta })\right) \, d\tau }\nonumber \\&= O(1) |Y-Y^{\text {ms}}|^{\frac{2}{3(1-\varepsilon )}}\left( 1 + O_{Y\rightarrow Y^{\text {ms}}}(|Y-Y^{\text {ms}}|^{\eta })\right) , \end{aligned}$$

which proves (7.604).

The asymptotics for $e^{2\tilde{\mu }}$ in (7.605) follows directly from (7.603) and (7.494). The asymptotics for $e^{2\tilde{\lambda }}$ in (7.605) can be read off from (7.603)–(7.604), (2.69), and the identity (3.98), which in the $(d,w,\chi )$ variables reads

7.612 $$\begin{aligned} d^{\frac{1}{1-\varepsilon }}e^{\tilde{\lambda }}\chi ^2 = \alpha Y^{2+\eta }, \end{aligned}$$

for some constant $\alpha >0$. It then follows from (7.612) that as $\delta Y\rightarrow 0^+$, $e^{2\tilde{\lambda }} \asymp (\delta Y)^{-\frac{2}{3(1-\varepsilon )}}$.

From  (2.57), we conclude that $\Sigma (Y) = d(Y)^{\frac{1+\varepsilon }{1-\varepsilon }}\asymp _{Y\rightarrow Y^{\text {ms}}} \delta Y^{-\frac{1+\varepsilon }{1-\varepsilon }}$, where we slightly abuse notation by continuing to denote $\Sigma (Y)$ to signify the selfsimilar energy density (see (2.19)). Therefore by (2.19), for any $\tilde{\tau }>0$

7.613 $$\begin{aligned} \rho (\tilde{\tau },R)&= \frac{1}{\tau (\tilde{\tau },R)^2} \Sigma (Y) = \frac{R^{2\eta }}{\tilde{\tau }^{2\frac{1+\varepsilon }{1-\varepsilon }}\varepsilon ^\eta } \Sigma (Y) =\frac{1}{\tilde{\tau }^2 Y^2} \Sigma (Y) \asymp _{Y\rightarrow Y^{\text {ms}}} \delta Y^{-\frac{1+\varepsilon }{1-\varepsilon }}. \end{aligned}$$

The fluid density therefore implodes along $\mathcal {M}\mathcal {S}_\varepsilon$ and as a consequence of (2.75), the Ricci scalar blows up at $\mathcal {M}\mathcal {S}_\varepsilon$. $\square$

#### Remark 7.17

We can now use the asymptotic rates from from the previous lemma to replace the rough upper bound $\chi ^{-2}w^{-2}\lesssim 1$, by the sharp upper bound rate $|Y-Y^{\text {ms}}|^{2-\frac{4}{3(1-\varepsilon )}}$. This can then be bootstrapped to obtain the near optimal next order correction in the rates (7.603)–(7.602), where $\eta$ can be replaced by $\frac{2}{3}+O(\varepsilon )$. We do not pursue this here, as it will not be needed in the rest of the paper.

## Causal Structure of the RLP Family of Solutions

As explained in Section 2.6, Theorems 2.3, 2.4, and 2.5 imply the existence of a maximally selfsimilarly extended RLP spacetime, recall Definition 2.7. The standard ODE-theory implies that the solution is indeed real-analytic in *Y* on $(Y^{\text {ms}},\infty )$. We next show that the RLP-spacetimes are not asymptotically flat and compute the associated mass-aspect function as $r\rightarrow \infty$.

### Lemma 8.1

($(\mathcal {M}_{\text {RLP},\varepsilon },g_{\text {RLP},\varepsilon })$ is not asymptotically flat) The RLP spacetimes $(\mathcal {M}_{\text {RLP},\varepsilon }, g_{\text {RLP},\varepsilon })$ are not asymptotically flat, i.e. for any $\tau <0$, $\lim _{R\rightarrow \infty }m(\tau ,R)=\infty$. More precisely the mass aspect function $\frac{2m(\tau ,r)}{r}$ satisfies

$$\begin{aligned} \lim _{r\rightarrow \infty }\frac{2m(\tau ,r)}{r}=\lim _{R\rightarrow \infty } \frac{2m(\tau ,R)}{r(\tau ,R)} = 4\varepsilon d_2, \end{aligned}$$

where $d_2>1$ is the $\varepsilon$-independent constant introduced in Lemma 6.5.

### Proof

Fix a $\tau <0$. As *R* increases to $\infty$ we have $y=\frac{R}{-\sqrt{\varepsilon }\tau }\rightarrow \infty$. Recalling (2.10) we have

8.614 $$\begin{aligned} \lim _{R\rightarrow \infty } \frac{m(\tau ,R)}{R}&= \lim _{R\rightarrow \infty } \frac{4\pi \int _0^R \frac{1}{2\pi \tau ^2} \Sigma (y)\, \varepsilon \tau ^2 \tilde{r}^2(y) \tilde{r}'(y) \,d\bar{R}}{R} \nonumber \\&=2\varepsilon \lim _{y\rightarrow \infty } \frac{\int _0^y \Sigma (z) \tilde{r}(z)^2 \tilde{r}'(z) \,dz}{y} \nonumber \\&= 2\varepsilon \lim _{y\rightarrow \infty } \Sigma (y)\tilde{r}(y)^2\tilde{r}'(y) =2\varepsilon d_2, \end{aligned}$$

where we have changed variables and used $y=\frac{R}{-\sqrt{\varepsilon }\tau }$ in the second equality, the l’Hospital rule in the third, and (6.454)–(6.455), (2.33), Remark 7.2 in the last. Note that $\lim _{R\rightarrow \infty }\frac{r}{R}=\lim _{r\rightarrow \infty }\frac{r}{R}=1$ by (7.499)–(7.501). $\square$

As stated in [28], it is clear that along any line of the form $(\tau ,\alpha \tau )$ the density $\rho (\tau ,\alpha \tau ) = \frac{1}{2\pi \tau ^2} \Sigma (\frac{-\alpha }{\sqrt{\varepsilon }})$ diverges as $\tau \rightarrow 0^-$. A similar statement applies when we approach the scaling origin (0, 0) along the lines of the form $(\tilde{\tau },\alpha \tilde{\tau })$, $\tilde{\tau }>0$. In particular, by (2.75) the Ricci scalar blows up at (0, 0) and this is a geometric singularity. We now proceed to study the radial null-geodesics that “emanate" from this singularity. We shall henceforth use the abbreviation RNG for the radial null geodesics.

### Lemma 8.2

*(a)*: In the $(\tau ,R)$-plane the outgoing/ingoing RNG-s respectively satisfy the equations
  8.615 $$\begin{aligned} \frac{dR}{d\tau } = \pm e^{\mu (y)-\lambda (y)}, \ \ y = \frac{R(\tau )}{-\sqrt{\varepsilon }\tau }, \end{aligned}$$
  whenever the right-hand side is well-defined.
*(b)*: Similarly, in the $(\tilde{\tau },R)$-plane the outgoing/ingoing RNG-s respectively satisfy the equations
  8.616 $$\begin{aligned} \frac{dR}{d\tilde{\tau }} = \frac{\pm 1}{e^{\tilde{\lambda }(Y)-\tilde{\mu }(Y)}\mp \frac{2\sqrt{\varepsilon }}{1+\varepsilon }Y}, \ \ Y = \frac{-\sqrt{\varepsilon }\tilde{\tau }}{R(\tilde{\tau })}, \end{aligned}$$
  whenever the right-hand side is well-defined.
*(c)*: If we let $Y = -\frac{\sqrt{\varepsilon }}{\sigma }$, then the curve $(\sigma \tilde{\tau },\tilde{\tau })$ is a simple outgoing/ingoingRNG (see Definition 2.11) if and only if
  8.617 $$\begin{aligned} G_\pm (Y) = 0, \end{aligned}$$
  where
  8.618 $$\begin{aligned} G_\pm (Y){:}{=}\sqrt{\varepsilon }e^{\tilde{\lambda }(Y)-\tilde{\mu }(Y)} \pm \frac{1-\varepsilon }{1+\varepsilon }Y. \end{aligned}$$

### Proof

*Proof of part (a).* Equation (8.615) is just the condition that the radial geodesic has null length in the local coordinates (2.70).

*Proof of part (b)*. In the local coordinates (2.65) the RNG-s satisfy

8.619 $$\begin{aligned} -e^{2\tilde{\mu }(Y)}\left( \dot{\tilde{\tau }}(s)\right) ^2- \frac{4\sqrt{\varepsilon }}{1+\varepsilon } Ye^{2\tilde{\mu }} \dot{R}(s)\dot{\tilde{\tau }}(s) + \left( e^{2\tilde{\lambda }(Y)}-\frac{4\varepsilon }{(1+\varepsilon )^2} Y^2 e^{2\tilde{\mu }(Y)} \right) \left( \dot{R}(s)\right) ^2=0, \end{aligned}$$

where it is understood that $Y = \frac{-\sqrt{\varepsilon }\tilde{\tau }(s)}{R(s)}$ in the above expression. Reparametrising by $\tilde{\tau }$ the above ODE, we obtain

8.620 $$\begin{aligned} 1&= \left( e^{2\tilde{\lambda }(Y)-2\tilde{\mu }(Y)}-\frac{4\varepsilon }{(1+\varepsilon )^2} Y^2 \right) \left( \frac{dR}{d\tilde{\tau }}\right) ^2 - \frac{4\sqrt{\varepsilon }}{1+\varepsilon } Y \frac{dR}{d\tilde{\tau }}, \end{aligned}$$

or equivalently

8.621 $$\begin{aligned} \left( \frac{dR}{d\tilde{\tau }}\right) ^2e^{2\tilde{\lambda }(Y)-2\tilde{\mu }(Y)} = \left( 1+\frac{2\sqrt{\varepsilon }}{1+\varepsilon } Y \frac{dR}{d\tilde{\tau }}\right) ^2. \end{aligned}$$

Upon taking the square root, solutions to the equation (8.621) are given by the solutions of (8.616).

*Proof of part (c)*. The proof follows by direct substitution in (8.616). $\square$

### Remark 8.3

For the purpose of describing the causal structure of the selfsimilar spacetimes under consideration, it is convenient to introduce the function

8.622 $$\begin{aligned} F_\varepsilon (Y): = Y^2 e^{2\tilde{\mu }_\varepsilon (Y)-2\tilde{\lambda }_\varepsilon (Y)}, \ \ Y>Y^{\text {ms}}, \end{aligned}$$

where $Y = \frac{-\sqrt{\varepsilon }\tilde{\tau }}{R}$ is the selfsimilar coordinate associated with the patch (2.65) and we keep the index $\varepsilon$ to emphasise the dependence on $\varepsilon$. It is then straightforward to check from part (c) of Lemma 8.2 that an RNG $(\sigma \tilde{\tau },\tilde{\tau })$, $\sigma \ne 0$, is simple if and only if

8.623 $$\begin{aligned} \frac{1}{\varepsilon }F_\varepsilon (Y) = \left( \frac{1+\varepsilon }{1-\varepsilon }\right) ^2, \ \ Y = - \frac{\sqrt{\varepsilon }}{\sigma }. \end{aligned}$$

Formulas (8.618) and (8.622) give the obvious factorisation property:

8.624 $$\begin{aligned} G_+(Y) G_-(Y) = - \varepsilon \left( \frac{1-\varepsilon }{1+\varepsilon }\right) ^2e^{2\tilde{\lambda }_\varepsilon (Y)-2\tilde{\mu }_\varepsilon (Y)}\left( \frac{1}{\varepsilon }F_\varepsilon (Y) -\left( \frac{1+\varepsilon }{1-\varepsilon }\right) ^2\right) . \end{aligned}$$

### Proposition 8.4

Let $F_\varepsilon (\cdot )$ be the function given by (8.622). There exists an $0<\varepsilon _0\ll 1$ such that the following statements are true.

*(a)*: The function $F_\varepsilon$ satisfies the formula
  8.625 $$\begin{aligned} F_\varepsilon (Y) =\frac{(1+\varepsilon )^2}{(1-\varepsilon )^2} \frac{(d_\varepsilon Y^{-2})^{-\eta }Y^2 + \varepsilon \chi _\varepsilon ^2\left[ (w_\varepsilon -1)^2 - 4 w_\varepsilon d_\varepsilon \right] }{(w_\varepsilon +\varepsilon )^2\chi _\varepsilon ^2}, \ \ Y>Y^{\text {ms}}. \end{aligned}$$
*(b)*: There exists an $Y<0$ such that $Y>Y^{\text {ms}}(=Y^{\text {ms}}_\varepsilon )$ for all $\varepsilon \in (0,\varepsilon _0]$ and
  8.626 $$\begin{aligned} \frac{1}{\varepsilon }F_\varepsilon (Y) >2 \ \ \text { for all } \ \varepsilon \in (0,\varepsilon _0]. \end{aligned}$$
*(c)*: For any fixed $\varepsilon \in (0,\varepsilon _0]$ we have
  8.627 $$\begin{aligned} \lim _{Y\rightarrow 0} F_\varepsilon (Y)&= 0 , \end{aligned}$$
  8.628 $$\begin{aligned} \lim _{Y\rightarrow Y^{\text {ms}}} F_\varepsilon (Y)&= 0. \end{aligned}$$

### Proof

*Proof of part (a).* By (2.68) we have for any $Y>0$

8.629 $$\begin{aligned} F_\varepsilon (Y)&= Y^2 e^{2\tilde{\mu }_\varepsilon (Y)-2\tilde{\lambda }_\varepsilon (Y)} = \frac{(1+\varepsilon )^2}{(1-\varepsilon )^2} Y^{2+2\eta } e^{2\mu _\varepsilon (y)-2\lambda _\varepsilon (y)} \nonumber \\&= \frac{(1+\varepsilon )^2}{(1-\varepsilon )^2} y^{-2}e^{2\mu _\varepsilon (y)-2\lambda _\varepsilon (y)} =\frac{(1+\varepsilon )^2}{(1-\varepsilon )^2} \frac{ \textbf{d}_\varepsilon ^{-\eta } +\varepsilon \tilde{r}_\varepsilon ^2 \left[ (\textbf{w}_\varepsilon -1)^2- 4 \textbf{d}_\varepsilon \textbf{w}_\varepsilon \right] }{(\textbf{w}_\varepsilon +\varepsilon )^2 \tilde{r}_\varepsilon ^2} \nonumber \\&=\frac{(1+\varepsilon )^2}{(1-\varepsilon )^2} \frac{(d_\varepsilon Y^{-2})^{-\eta }Y^2 + \varepsilon \chi _\varepsilon ^2\left[ (w_\varepsilon -1)^2 - 4 w_\varepsilon d_\varepsilon \right] }{(w_\varepsilon +\varepsilon )^2\chi _\varepsilon ^2}, \ \ y, Y>0, \end{aligned}$$

where the index $\varepsilon$ is added to emphasise the dependence on $\varepsilon$. We used the formula (2.56) in the third equality, and (7.498) to express $y^{-2}e^{2\mu _\varepsilon (y)-2\lambda _\varepsilon (y)}$ in terms of $\textbf{w}_\varepsilon$, $\textbf{d}_\varepsilon$, and $\tilde{r}_\varepsilon$ in the fourth. Since the right-most side of (8.629) extends analytically to $Y\in (Y^{\text {ms}},0]$ by Theorems 7.4 and 2.5 the claim in part (a) follows.

*Proof of part (b).* Note that for $Y\in (Y^{\text {ms}},0]$ by (8.629)

8.630 $$\begin{aligned} \frac{1}{\varepsilon }F_\varepsilon = \frac{(1+\varepsilon )^2}{\varepsilon (1-\varepsilon )^2} \frac{(d_\varepsilon Y^{-2})^{-\eta }Y^2}{(w_\varepsilon +\varepsilon )^2\chi _\varepsilon ^2} + \frac{(1+\varepsilon )^2}{(1-\varepsilon )^2} \frac{\left[ (w_\varepsilon -1)^2 - 4 w_\varepsilon d_\varepsilon \right] }{(w_\varepsilon +\varepsilon )^2}. \end{aligned}$$

We now fix $Y=Y_0$, a constant provided by the local extendibility statement of Theorem 7.4. In particular, by part (a) of Theorem 7.4 there exists a $\delta >0$ such that for all $\varepsilon \in (0,\varepsilon _0]$ we have $d_\varepsilon (Y_0)>\delta$, $\chi _\varepsilon (Y_0)>\delta$ and $d_\varepsilon (Y_0)<w_\varepsilon (Y_0)<\frac{1}{\delta }$. By letting $\varepsilon \rightarrow 0$ in (8.630) we conclude that

8.631 $$\begin{aligned} \frac{1}{\varepsilon }F_\varepsilon (Y_0) >2 \ \ \text { for } \ \varepsilon \text { sufficiently small}. \end{aligned}$$

*Proof of part (c).* Claim (8.627) follows from the formula (8.629) and the asymptotic behaviour (7.499)–(7.503). To prove (8.628) we work directly with (8.622) and use (7.605). This gives

8.632 $$\begin{aligned} \lim _{Y\rightarrow (Y^{\text {ms}})^+}F_\varepsilon (Y)&= \lim _{Y\rightarrow (Y^{\text {ms}})^+} \left( Y^2 e^{2\mu _\varepsilon (Y)-2\lambda _\varepsilon (Y)}\right) \nonumber \\&\asymp _{Y\rightarrow Y^{\text {ms}}} c (Y^{\text {ms}})^2 \lim _{(Y-Y^{\text {ms}})\rightarrow 0^+} (\delta Y)^{\frac{2(1+3\varepsilon )}{3(1-\varepsilon )}} = 0. \end{aligned}$$

$$\square $$

We are now ready to prove Theorem 2.12.

*Proof of Theorem* 2.12. Our first goal is to show that for $0<\varepsilon \ll 1$ there exist at least two solutions to the equation (8.623). By parts (b) and (c) of Proposition 8.4 it is now clear that the function $Y\mapsto \frac{1}{\varepsilon }F_\varepsilon (Y)$ converges to 0 at $Y=Y^{\text {ms}}$ and $Y=0$, but necessarily peaks above $\left( \frac{1+\varepsilon }{1-\varepsilon }\right) ^2$ at $Y=Y_0$, where $Y_0$ is given by Theorem 7.4. Therefore there exist $Y_1\in (Y_0,0)$ and $Y_2\in (Y^{\text {ms}},Y_0)$ such that (8.623) holds with $Y=Y_1$ and $Y=Y_2$. Since $Y_1, Y_2$ are strictly negative and necessarily zeroes of $G_\pm$ defined by (8.618), they must in fact be zeroes of $G_+$ and therefore represent outgoing simple RNG-s. Note that the function $Y\mapsto F_\varepsilon (Y) -\varepsilon \left( \frac{1+\varepsilon }{1-\varepsilon }\right) ^2$ is real analytic on $(Y^{\text {ms}},0)$ and therefore the number of zeroes is finite. By slight abuse of notation we enumerate the zeroes as in (2.77). $\square$

We recall the relationship (2.56) between the comoving selfsimilar coordinates *y* and *Y*, as well as the relation between the comoving coordinate *y* and the Schwarzschild coordinates *x* in Subsection 3.2. Recalling the sonic point $\bar{x}_*$, the slope of the sonic line in the *Y*-coordinates is given by

8.633 $$\begin{aligned} Y^{\text {sp}}{:}{=}\left( \tilde{r}^{-1}(\bar{x}_*)\right) ^{-\frac{1}{1+\eta }}. \end{aligned}$$

The next lemma is important for the description of ingoing null-geodesics. It in particular implies that the curve $\mathcal {N}$ is the unique simple ingoing RNG.

### Lemma 8.5

For any $\varepsilon \in (0,\varepsilon _0]$, consider the relativistic Larson-Penston spacetime given by Definition 2.7. Then there exists a $Y_{\mathcal {N}}\in (0,Y^{\text {sp}})$ such that the curve

8.634 $$\begin{aligned} \mathcal {N} : = \{(\tilde{\tau }, R)\in \mathcal {M}_{\text {RLP},\varepsilon }\,\big | \, \frac{-\sqrt{\varepsilon }\tilde{\tau }}{R} = Y_{\mathcal {N}}\}, \end{aligned}$$

represents a simple ingoing null-geodesics i.e. the boundary of the past light cone of the scaling origin $\mathcal {O}$. Moreover, the curve $\mathcal {N}$ is the unique simple ingoing RNG and the following bounds hold:

8.635 $$\begin{aligned} G_-(Y)&>0, \ \ Y\in (Y^{\text {ms}}, Y_{\mathcal {N}}), \end{aligned}$$

8.636 $$\begin{aligned} G_-(Y)&<0, \ \ Y\in (Y_{\mathcal {N}},\infty ), \end{aligned}$$

where $G_-(\cdot )$ is defined in (8.618).

### Proof

By Theorem 2.12 it suffices to show that there exists a $Y_{\mathcal {N}}\in (0,Y^{\text {sp}})$ which solves the equation (8.623). By part (c) of Proposition 8.4 we know that the function $Y\mapsto \frac{1}{\varepsilon }F_\varepsilon (Y)$ converges to 0 at $Y=0$. On the other hand, from (8.622) and (2.68) we have

8.637 $$\begin{aligned} F_\varepsilon (Y)&= \frac{(1+\varepsilon )^2}{(1-\varepsilon )^2} y^{-2} e^{2\mu (y)-2\lambda (y)} = \frac{(1+\varepsilon )^2}{(1-\varepsilon )^2} y^{-2} \left( e^{2\mu (y)-2\lambda (y)}-y^2\right) + \frac{(1+\varepsilon )^2}{(1-\varepsilon )^2}. \end{aligned}$$

Therefore, at the sonic point $Y^{\text {sp}}$ we conclude $F_\varepsilon (Y^{\text {sp}})=\frac{(1+\varepsilon )^2}{(1-\varepsilon )^2}$, which implies that $\frac{1}{\varepsilon }F_\varepsilon (Y^{\text {sp}})$ is larger than $\left( \frac{1+\varepsilon }{1-\varepsilon }\right) ^2$ for all $\varepsilon \in (0,1)$. By continuity, there exists an $Y_{\mathcal {N}}\in (0,Y^{\text {sp}})$ such that $\frac{1}{\varepsilon }F_\varepsilon (Y_{\mathcal {N}})= \left( \frac{1+\varepsilon }{1-\varepsilon }\right) ^2$. Now observe that by (8.637), the zeroes of the function $\frac{1}{\varepsilon }F_\varepsilon (Y)-\left( \frac{1+\varepsilon }{1-\varepsilon }\right) ^2$ are in 1-1 relationship with the zeroes of the function $(0,\infty )\ni y\mapsto e^{2\lambda }y^2 - \frac{1}{\varepsilon }e^{2\mu }$. It is shown in Lemma A.4 that the map $y\mapsto \tilde{r}(y)^2\left( e^{2\lambda }y^2 - \frac{1}{\varepsilon }e^{2\mu }\right)$ is strictly monotone on $(0,\infty )$ and the uniqueness claim follows. Inequalities (8.635)–(8.636) are a simple consequence of the factorisation (8.624), the above monotonicity, and the positivity of $G_+$ for all $Y>0$. $\square$

### Definition 8.6

We refer to the region in the future of the backward null-curve $\mathcal {N}$ as the exterior region, and the region in the past of the null-curve $\mathcal {N}$ as the interior region, following here the terminology in [6], see Figure 5.

### Remark 8.7

For any $(R,\tilde{\tau })$ in the exterior region, we note that $\sqrt{\varepsilon }e^{\tilde{\mu }-\tilde{\lambda }}-\frac{2\varepsilon }{1+\varepsilon }Y$ is clearly positive for $Y\in (Y^{\text {ms}},0]$. When $Y\in (0,Y_{\mathcal {N}})$ we may rewrite this expression as $G_-(Y)+\frac{1-3\varepsilon }{1+\varepsilon }Y$, which is then positive by Lemma 8.5 for sufficiently small $\varepsilon$. On the other hand, the expression $\sqrt{\varepsilon }e^{\tilde{\mu }-\tilde{\lambda }}+\frac{2\varepsilon }{1+\varepsilon }Y$ is clearly positive for $Y\ge 0$. If $Y\in (Y_1,0)$, we may rewrite it as $G_+(Y)-\frac{1-3\varepsilon }{1+\varepsilon }Y$, which is then necessarily positive. This shows that the right-hand side of the null-geodesic equation appearing in (8.616) is well-defined in the exterior region $\{Y_1<Y<Y_{\mathcal {N}}\}$.

### Lemma 8.8

(General structure of radial null geodesics)

- For any point $(R_0,\tilde{\tau }_0)$ in the interior region, the future oriented ingoing null curve $\tilde{\tau }\mapsto R(\tilde{\tau })$ through $(R_0,\tilde{\tau }_0)$ remains in the interior region and intersects the surface $\{R=0\}$ at some $\tilde{\tau }<0$.
- For any point $(R_0,\tilde{\tau }_0)$ in the exterior region, the future oriented ingoing null curve $\tilde{\tau }\mapsto R(\tilde{\tau })$ exits the exterior region by intersecting $\mathcal {B}_1$ and also intersects $\mathcal {B}_2$ at positive values of the *R*-coordinate.
- For any $(R_0,\tilde{\tau }_0)$ in the union of the exterior and the interior region, the outgoing null curve $\tilde{\tau }\mapsto R(\tilde{\tau })$ through $(R_0,\tilde{\tau }_0)$ exists globally-in-$\tilde{\tau }$ and $\lim _{\tilde{\tau }\rightarrow \infty }R(\tilde{\tau })=\infty$. Moreover, no such curve can converge to the scaling origin $\mathcal {O}$ to the past.

### Proof

*Proof of part (a)*. Assume now that $(R_0,\tilde{\tau }_0)$ is in the interior region. The future oriented ingoing geodesic must stay in the interior region, as it cannot cross the backward light cone $Y=Y_{\mathcal {N}}$ by the ODE uniqueness theorem. In the interior region it is more convenient to switch to the original comoving coordinates $(R,\tau )$ as they also cover the centre of symmetry surface $\{(R,\tau )\,\big | \, R=0, \ \tau<0\} = \{(r,\tau ), \, \big | \, r=0,\tau <0\}$. Let $(R_0,\tau _0)$ correspond to $(R_0,\tilde{\tau }_0)$. The ingoing geodesic equation reads $\partial _\tau R = - e^{\mu -\lambda }$ and the interior region is characterised by the condition $e^{\mu (y)-\lambda (y)}>\sqrt{\varepsilon }y$. Let now $T=-\log (-\tau )$ for $\tau <0$. We then have $\frac{dy}{dT} =\frac{dy}{d\tau }\frac{d\tau }{dT} =\left( \frac{R_\tau }{-\sqrt{\varepsilon }\tau } + \frac{R}{\sqrt{\varepsilon }\tau ^2}\right) (-\tau )=-\frac{e^{\mu (y)-\lambda (y)}-\sqrt{\varepsilon }y}{\sqrt{\varepsilon }}.$ In particular $\frac{dy}{dT}<0$, the right-hand side is smooth, and there are no fixed points of the above ODE on the interval $(0, y_{\mathcal {N}})$. We wish to show that the time *T* it takes to reach $y=0$ is finite. Integrating the above ODE, we see that

$$\begin{aligned} T(y)-T_0 = \int _{y_0}^y\frac{-\sqrt{\varepsilon }}{e^{\mu (\theta )-\lambda (\theta )}-\sqrt{\varepsilon }\theta }\, d\theta , \end{aligned}$$

where $T_0 = -\log |\tau _0|$, $y_0 = \frac{R_0}{-\sqrt{\varepsilon }\tau _0}$. Note that $e^{-\lambda (y)}\asymp _{y\rightarrow 0^+} \frac{1}{\tilde{r}'(y)}$ by (3.95). We next use (2.73) and $e^{\mu (0)}>0$ to conclude that, as $y\rightarrow 0^+$, the denominator inside the integral above asymptotes to a constant multiple $y^{-\frac{2}{3(1+\varepsilon )}}$. Since the latter is integrable near $y=0$, it follows that $\lim _{y\rightarrow 0^+}T(y)<\infty$, as desired.

*Proof of part (b)*. Let $(R_0,\tilde{\tau }_0)$ belong to the exterior region. We consider the change of variables $\tilde{\tau }\mapsto Y$ and the particle label *R* as a function of *Y*. By (2.55) and the geodesic equations (8.616), along any null-geodesic we have

8.638 $$\begin{aligned} \frac{dY}{d\tilde{\tau }}&= \frac{d}{d\tilde{\tau }}\left( -\frac{\sqrt{\varepsilon }\tilde{\tau }}{R(\tilde{\tau })}\right) = \frac{-\sqrt{\varepsilon }}{R(\tilde{\tau })} \left( 1-\tilde{\tau }\frac{1}{R(\tilde{\tau })}\frac{dR}{d\tilde{\tau }}\right) \nonumber \\&= \frac{-\sqrt{\varepsilon }}{R(\tilde{\tau })} \left( 1 \pm \frac{1}{\sqrt{\varepsilon }} \frac{Y}{e^{\tilde{\lambda }(Y)-\tilde{\mu }(Y)}\mp \frac{2\sqrt{\varepsilon }Y}{1+\varepsilon }}\right) = -\frac{1}{R(\tilde{\tau })} \frac{G_\pm (Y)}{ e^{\tilde{\lambda }(Y)-\tilde{\mu }(Y)}\mp \frac{2\sqrt{\varepsilon }Y}{1+\varepsilon }}, \end{aligned}$$

where

8.639 $$\begin{aligned} G_\pm (Y){:}{=}\sqrt{\varepsilon }e^{\tilde{\lambda }(Y)-\tilde{\mu }(Y)} \pm \frac{1-\varepsilon }{1+\varepsilon }Y. \end{aligned}$$

We note that by Remark 8.7 all the denominators appearing above are nonzero. Therefore the geodesic equations (8.616) transform into

8.640 $$\begin{aligned} \frac{\pm 1}{e^{\tilde{\lambda }(Y)-\tilde{\mu }(Y)}\mp \frac{2\sqrt{\varepsilon }Y}{1+\varepsilon }}&= \frac{dR}{dY}\frac{dY}{d\tilde{\tau }} = \frac{-1}{R(Y)}\frac{G_\pm (Y)}{ e^{\tilde{\lambda }(Y)-\tilde{\mu }(Y)}\mp \frac{2\sqrt{\varepsilon }Y}{1+\varepsilon }}\frac{dR}{dY}. \end{aligned}$$

For as long as $R>0$ we can rewrite the above ODE in the form

8.641 $$\begin{aligned} \frac{d}{dY}(\log R) = \frac{\mp 1}{G_\pm (Y)}. \end{aligned}$$

We remark that by Lemma 8.5 the denominator is strictly positive in the exterior region and in the case of outgoing geodesics, function $G_+$ is in fact strictly positive for all $Y\in (Y_1,\infty )$ (positivity of $G_+$ characterises the region “below" $\mathcal {B}_1$).

For the ingoing geodesics, the exterior region is invariant by the flow. We integrate (8.641) to conclude that (with $Y_\bullet {:}{=}-\frac{\sqrt{\varepsilon }\tilde{\tau }_0}{R_0}$)

8.642 $$\begin{aligned} \log R(Y) - \log R_0 =- \int _{Y}^{Y_\bullet } \frac{1}{G_-(Z)}\, dZ. \end{aligned}$$

The map $Z\mapsto G_-(Z)$ is smooth and by Lemma 8.5 it is strictly positive on the interval $(Y^{\text {ms}}, Y_{\mathcal {N}})$. Therefore, *R* is positive as the ingoing RNG traverses $\mathcal {B}_1$ and $\mathcal {B}_2$, as the right-hand side of (8.642) is finite for any $Y>Y^{\text {ms}}$.

*Proof of part (c)*. Let $(R_0,\tilde{\tau }_0)$ belong to the exterior region. The outgoing null-geodesic solves the ODE

$$\begin{aligned} \frac{d}{dY}(\log R) = \frac{- 1}{G_+(Y)}. \end{aligned}$$

Note that $G_+$ is smooth on $(Y_1,Y_{\mathcal {N}})$. Since $Y_1<0$ is the largest negative root of $Z \mapsto G_+(Z)$, the right-hand side above is negative. It follows that $Y\mapsto R(Y)$ is decreasing on $(Y_1,Y_{\bullet })$, i.e. *R*(*Y*) increases as *Y* approaches $Y_1$ from the right. In particular, the solution exists for all $Y\in (Y_1, Y_{\bullet }]$ by the strict negativity of the right-hand side above. We consequently have the formula

8.643 $$\begin{aligned} R(Y) = R_0 \exp \left( \int _{Y}^{Y_\bullet } \frac{1}{G_+(Z)}\, dZ\right) , \ \ Y>Y_1. \end{aligned}$$

Since the function $G_+$ is positive for $Z>Y_1$, if $G(Z)=C(Z-Y_1)^m(1+O(|Z-Y_1|))$ is the first term of Taylor expansion of *G* at $Y_1$, with $m\in \mathbb N$ (recall that *G* is real analytic), then necessarily $C>0$. Plugging this expansion into (8.643) and integrating we conclude $\lim _{Y\rightarrow Y_1^+}R(Y)=\infty$, which shows that the outgoing null-geodesic asymptotes to $\mathcal {B}_1$. Since $Y= - \frac{\sqrt{\varepsilon }\tilde{\tau }(Y)}{R(Y)}$ and $\lim _{Y\rightarrow (Y_1)^+}R(Y)=\infty$, it follows that $\lim _{Y\rightarrow (Y_1)^+}\tilde{\tau }(Y)=\infty$, and therefore the outgoing geodesic exists globally on $[\tilde{\tau }_0,\infty )$ and $\lim _{\tilde{\tau }\rightarrow \infty }R(\tilde{\tau })=\infty$. If $(R_0,\tilde{\tau }_0)$ belongs to the interior region, a similar analysis in the original comoving coordinates $(R,\tau )$ yields the same conclusion.

Since the function $G_+(Z)$ is smooth and bounded in a neighbourhood of $Z=0$, it follows from (8.643) that any outgoing geodesic starting at $(R_0,\tilde{\tau }_0)$ with $\tilde{\tau }_0>0$ intersects $\{\tilde{\tau }=0\}$ axis (i.e. $Y=0$) at a positive value of *R* to the past. Due to the monotonicity of the flow (8.643), any outgoing geodesic emanating from $(R_0,\tilde{\tau }_0)$ with $\tilde{\tau }_0\le 0$ remains below $\{\tilde{\tau }=0\}$ axis to the past. $\square$

## Asymptotic Flattening of the Selfsimilar Profile

The key result of this section is the local well-posedness for the characteristic initial value problem for the Einstein-Euler system, see Theorem 9.4. The idea is to suitably truncate the selfsimilar spacetime as described in Section 2.7. We work with the double-null formulation, see Section 2.7.1, and our starting point is the reformulation of the fluid evolution equations (2.87)–(2.88).

### Reformulation of the Fluid Evolution and the Effective Transport Velocity

We introduce the constant

9.644 $$\begin{aligned} k_\pm : = 1\pm \frac{\sqrt{2\eta +\eta ^2}}{1+\eta }, \end{aligned}$$

where we recall $\eta =\eta (\varepsilon )$ is given by (2.38) and from (9.644) it is clear that $k_\pm = 1\pm O(\sqrt{\eta })$.

#### Lemma 9.1

(Reformulation of the Euler equations) Assume that $(\rho ,u^\nu ,r,\Omega )$ is a $C^1$ solution of (2.87)–(2.88). Let

9.645 $$\begin{aligned} \mathcal {U}&{:}{=}(1+\eta ) \Omega ^2 (u^q)^2, \end{aligned}$$

9.646 $$\begin{aligned} f^+&: = \frac{(1-\varepsilon )^{k_+}}{1+\varepsilon } {r^{(2+2\eta )k_+-2}\rho ^{k_+-1}} (u^q)^2, \end{aligned}$$

9.647 $$\begin{aligned} f^-&: = \frac{1+\varepsilon }{(1-\varepsilon )^{k_-} } \frac{ r^{2-(2+2\eta )k_-} \rho ^{1-k_-}}{(u^q)^2}. \end{aligned}$$

Then the new unknowns $f^\pm$ satisfy

9.648 $$\begin{aligned} \partial _pf^+ + k_+ \mathcal {U} \partial _qf^+ + 2k_+ (2\frac{\partial _q\Omega }{\Omega } - 2\eta \frac{\partial _qr}{r}) \mathcal {U} f^+&=0, \end{aligned}$$

9.649 $$\begin{aligned} \partial _pf^- + k_- \mathcal {U} \partial _qf^- - 2k_- (2\frac{\partial _q\Omega }{\Omega } - 2\eta \frac{\partial _qr}{r}) \mathcal {U} f^-&=0. \end{aligned}$$

#### Proof

Let $U{:}{=} \Omega ^4r^2T^{pp}$ and $V{:}{=} \Omega ^2r^{2+2\eta } T^{pq}$. Using (2.91) we rewrite $\Omega ^4r^2T^{qq}= (1+\eta )^2 \frac{\Omega ^4}{r^{4\eta }} \frac{V^2}{U}$. Therefore, (2.87)–(2.88) can be rewritten in the form

9.650 $$\begin{aligned} \partial _pU +\frac{\Omega ^2}{r^{2\eta }} \partial _qV =0, \quad \partial _pV +(1+\eta )^2 \frac{r^{2\eta }}{\Omega ^2}\partial _q( \frac{\Omega ^4}{r^{4\eta }} \frac{V^2}{U} )=0. \end{aligned}$$

For any $k\in \mathbb R$ we now compute $\partial _p\left( \frac{V^k}{U}\right)$ and thereby use (9.650):

9.651 $$\begin{aligned} \partial _p(\frac{V^k}{U})&= k \frac{V^{k-1}}{U} \partial _pV - \frac{V^k}{U^2}\partial _pU \nonumber \\&=- k (1+\eta )^2 \frac{\Omega ^2}{r^{2\eta }} \frac{V}{U} \partial _q(\frac{V^k}{U}) - 2k (1+\eta )^2 \frac{V^{k+1}}{U^2}\partial _q\left( \frac{\Omega ^2}{r^{2\eta }}\right) \nonumber \\&\quad - k (2-k) (1+\eta )^2 \frac{\Omega ^2}{r^{2\eta }} \frac{V^k}{U^2} \partial _qV + \frac{V^k}{U^2} \frac{\Omega ^2}{r^{2\eta }} \partial _qV . \end{aligned}$$

We see that the last line (9.651) vanishes if *k* is a solution of the quadratic equation

9.652 $$\begin{aligned} k^2 - 2k+ \frac{1}{(1+\eta )^2} =0. \end{aligned}$$

The two distinct roots $k_\pm$ of (9.652) are given in (9.644), and the equation for $\frac{V^{k_{\pm }}}{U}$ reads

$$\begin{aligned} \partial _p\left( \frac{V^{k_\pm }}{U}\right) + k_\pm (1+\eta )^2 \frac{\Omega ^2}{r^{2\eta }} \frac{V}{U} \partial _q\left( \frac{V^{k_\pm }}{U}\right) + 2k_\pm (1+\eta )^2 \frac{V^{k_\pm +1}}{U^2}\partial _q\left( \frac{\Omega ^2}{r^{2\eta }}\right) = 0 . \end{aligned}$$

When $k=k_+>1$, we keep $\frac{V^{k_+}}{U}$ as the unknown. When however $k=k_-<1$, we work with $\frac{U}{V^{k}}$ instead, to avoid singularities for small values of $\rho$. From above we obtain the equation

$$\begin{aligned} \partial _p\left( \frac{U}{V^{k_-}}\right) + k_- (1+\eta )^2 \frac{\Omega ^2}{r^{2\eta }} \frac{V}{U} \partial _q\left( \frac{U}{V^{k_-}}\right) - 2k_- (1+\eta )^2 V^{1-k_-}\partial _q\left( \frac{\Omega ^2}{r^{2\eta }}\right) = 0. \end{aligned}$$

Going back to original variables, note that $\frac{V}{U}= \frac{r^{2\eta }}{(1+\eta ) \Omega ^4 (u^p)^2}$ and $\frac{V^k}{U} = \frac{(1-\varepsilon )^k}{1+\varepsilon } \frac{r^{(2+2\eta )n-2}\rho ^{k-1}}{\Omega ^4 (u^p)^2}$ so that

$$\begin{aligned} (1+\eta )^2\frac{\Omega ^2}{r^{2\eta }}\frac{V}{U}&= \frac{(1+\eta )}{ \Omega ^2 (u^p)^2} =(1+\eta ) \Omega ^2 (u^q)^2= \mathcal {U}, \\ \frac{V^{k_+}}{U}&= \frac{(1-\varepsilon )^{k_+}}{1+\varepsilon } \frac{r^{(2+2\eta )k_+-2}\rho ^{k_+-1}}{\Omega ^4 (u^p)^2} = f^+, \\ \frac{U}{V^{k_-}}&= \frac{1+\varepsilon }{(1-\varepsilon )^{k_-} } \Omega ^4 (u^p)^2 r^{2-(2+2\eta )k_-} \rho ^{1-k_-} = f^-, \end{aligned}$$

where we recall (9.645)–(9.647). $\square$

**Fig. 9:**
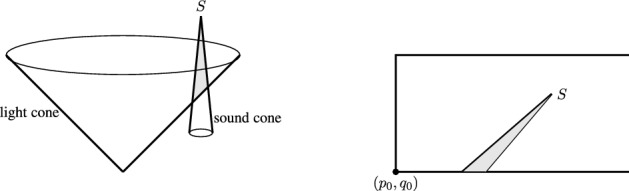
The grey shaded area in the infinite rectangular region $\mathcal {D}$ is a schematic depiction of the region bounded by the backward fluid characteristics emanating from a point $S\in \mathcal {D}$. The opening angle is of order $\sqrt{\varepsilon }\ll 1$, which of course is precisely the speed of sound

#### Remark 9.2

It follows from (9.646)–(9.647) that

9.653 $$\begin{aligned} \rho =\frac{1}{1-\varepsilon } \frac{ (f^+ f^-)^{\frac{1}{k_+-k_-}}}{r^{2+2\eta }}. \end{aligned}$$

Moreover, from (2.82) and (9.646)–(9.647) we have $\frac{f^+}{f^-} = \frac{1}{(1+\eta )^2} r^{4\eta } (u^q)^4$, which leads to the relation

9.654 $$\begin{aligned} \mathcal {U} =(1+\eta )\Omega ^2 (u^q)^2= (1+\eta )^2 \frac{\Omega ^2}{r^{2\eta }} \left( \frac{f^+}{f^-} \right) ^\frac{1}{2}. \end{aligned}$$

### Statement of the Local Well-Posedness Theorem

In order to flatten the the $g_{\text {RLP},\varepsilon }$ metric at asymptotic infinity, we shall treat the system (2.83)–(2.84) and (9.648)–(9.649) as the evolutionary part and equations (2.86)–(2.85) as the constraints.

#### Fixing the Choice of Double-Null Coordinates

We now fix a choice of double-null coordinates which will then be used to dampen the tails of the solution and produce an asymptotically flat spacetimes containing a naked singularity. Let $(\tilde{\tau }_0, R_0)\in \tilde{\mathcal {D}}_{\text {RLP},\varepsilon }$ be a given point in the exterior region (see Definition 8.6 and (2.66) for the definition of $\tilde{\mathcal {D}}_{\text {RLP},\varepsilon }$). Through $(\tilde{\tau }_0, R_0)$ we consider the ingoing null-curve which intersects the outgoing null-curve $\mathcal {B}_1$ (given by $R = \frac{\sqrt{\varepsilon }}{|Y^1|} \tilde{\tau }$) at some $(R_1,\tilde{\tau }_1)$ where by Lemma 8.8, $R_1>0$. We then fix the null-coordinate $p$ by demanding that

9.655 $$\begin{aligned} p= - 2(r-r_1) \ \ \text {along the ingoing null-geodesic through}\quad (R_0, \tilde{\tau }_0), \ \ r_1 : = \tilde{r}(R_1,\tilde{\tau }_1); \end{aligned}$$

and demanding that the level sets of $p$ correspond to outgoing null-geodesics. The choice (9.655) normalises $\mathcal {B}_1$ to correspond to the hypersruface $\{p= 0\}$ in the RLP-spacetime. Note that in the RLP spacetime, the ingoing curve through $(\tilde{\tau }_0, R_0)$ terminates at the massive singularity $\mathcal {M}\mathcal {S}_\varepsilon$ to the future.

Let now the outgoing null geodesic through $(\tilde{\tau }_0, R_0)$ intersect the surface $\mathcal {N}$ (boundary of the past of the scaling origin $\mathcal {O}$) at $(R_*,\tilde{\tau }_*)$. We then fix the null coordinate $q$ by demanding that

9.656 $$\begin{aligned} q= 2(r-r_*) \ \ \text {along the outgoing null-geodesic through}\quad (\tilde{\tau }_0, R_0), \ \ r_*: = \tilde{r}(R_*,\tilde{\tau }_*); \end{aligned}$$

and demanding that the level sets of $q$ correspond to ingoing null-geodesics. The normalisation (9.656) makes the surface $\mathcal {N}$ correspond to the hypersurface $\{q= 0\}$. A more detailed description of the RLP-spacetime in this double-null gauge is given in Lemma A.1.

Let $(p_0,q_0)$ be the point $(\tilde{\tau }_0,R_0)$ in the above double-null gauge. We shall consider the seminfinite rectangular domain

9.657 $$\begin{aligned} \mathcal {D} : =\{(p,q): p_0< p< 0, \ \ q>q_0 \}, \end{aligned}$$

where $|p_0|>0$ is sufficiently small, with data prescribed on the set $\underline{\mathcal {C}} \cup \mathcal {C},$ where

9.658 $$\begin{aligned} \underline{\mathcal {C}}&: = \left\{ (p,q) \ \Big | \ q=q_0, \ \ p\in [p_0,0]\right\} , \end{aligned}$$

9.659 $$\begin{aligned} \mathcal {C}&: = \left\{ (p,q) \ \Big | \ p=p_0, \ \ q\in [q_0,\infty )\right\} \end{aligned}$$

correspond to the ingoing and the outgoing null-curves emanating from $(p_0,q_0)$ respectively. See Figure 6.

#### Norms and Local Well-Posedness

Fix $N_\pm >0$ so that

9.660 $$\begin{aligned} N_+ = N_- - 4\eta , \quad N_- > \frac{k_+-k_-}{2} + 2\eta \end{aligned}$$

and let $0<\theta \ll 1$ be a small fixed constant. For any $f^\pm \in W^{2,\infty }(\overline{\mathcal {D}})$ and $(\Omega ,\frac{r}{q})\in W^{3,\infty }(\overline{\mathcal {D}})$, we define the total norm by

9.661 $$\begin{aligned} {\left| \left| \left| [f^\pm , \Omega , r] \right| \right| \right| }&{:}{=} {\left| \left| \left| [f^\pm ] \right| \right| \right| } + {\left| \left| \left| [\Omega , r] \right| \right| \right| } \end{aligned}$$

where

9.662 $$\begin{aligned} {\left| \left| \left| [f^\pm ] \right| \right| \right| }&{:}{=}\Vert \log (q^{N_+} f^+) \Vert _{\infty } +\sum _{j=1}^2\Vert q^{j} \partial _q^j \log f^+ \Vert _{\infty } \end{aligned}$$

9.663 $$\begin{aligned}&\quad +\Vert \log (q^{N_-} f^-) \Vert _{\infty } + \sum _{j=1}^2\Vert q^{j} \partial _q^j \log f^- \Vert _{\infty },\nonumber \\ {\left| \left| \left| [\Omega , r] \right| \right| \right| }&= \Vert \log \Omega \Vert _\infty + \sum _{j=1}^3 \Vert q^{j+\theta }\partial _q^j \log \Omega \Vert _{\infty } \end{aligned}$$

9.664 $$\begin{aligned}&\quad +\sum _{j=0}^3 \Vert q^{j-2} \partial _q^j (r^2) \Vert _{\infty } +\left\| \frac{q^2}{r^2}\right\| _\infty + \left\| \frac{q}{\partial _q(r^2)}\right\| _\infty , \end{aligned}$$

and the data norm

$$\begin{aligned} {\left| \left| \left| [f^\pm , \Omega , r]|_{\underline{\mathcal {C}}\cup \mathcal {C}} \right| \right| \right| }&{:}{=}{\left| \left| \left| [f^\pm ]|_{\underline{\mathcal {C}}\cup \mathcal {C}} \right| \right| \right| }+{\left| \left| \left| [\Omega , r]|_{\underline{\mathcal {C}}\cup \mathcal {C}} \right| \right| \right| } \\&{:}{=}\Vert \log (q^{N_+} f^+) |_{\underline{\mathcal {C}}\cup \mathcal {C}}\Vert _{\infty }+ \sum _{j=1}^2\Vert q^{j} \partial _q^j \log f^+ |_{\underline{\mathcal {C}}\cup \mathcal {C}}\Vert _{\infty }\\&\quad +\Vert \log (q^{N_-} f^-) |_{\underline{\mathcal {C}}\cup \mathcal {C}}\Vert _{\infty }+ \sum _{j=1}^2\Vert q^{j} \partial _q^j \log f^- |_{\underline{\mathcal {C}}\cup \mathcal {C}}\Vert _{\infty }\\&\quad +\Vert \log \Omega |_{\underline{\mathcal {C}}\cup \mathcal {C}} \Vert _\infty + \sum _{j=1}^3 \Vert q^{j+\theta }\partial _q^j \log \Omega |_{\underline{\mathcal {C}}\cup \mathcal {C}} \Vert _{\infty } \\&\quad + \sum _{j=0}^3 \Vert q^{j-2} \partial _q^j (r^2) |_{\underline{\mathcal {C}}\cup \mathcal {C}}\Vert _{\infty }+\left\| \frac{q^2}{r^2}\Big |_{\underline{\mathcal {C}}\cup \mathcal {C}} \right\| _\infty + \left\| \frac{q}{\partial _q(r^2)}\Big |_{\underline{\mathcal {C}}\cup \mathcal {C}} \right\| _\infty \\&\quad +\Vert q^{-1}\partial _p( r^2) |_{\underline{\mathcal {C}}}\Vert _\infty + \left\| \frac{r}{2} \left( 1+ \frac{4\partial _{p}r \partial _{q}r}{\Omega ^2}\right) \Big |_{\underline{\mathcal {C}}} \right\| _\infty \end{aligned}$$

We choose the data $(\hat{f}^\pm ,\hat{r},\hat{\Omega })$ on $\underline{\mathcal {C}}$ to coincide with the corresponding data obtained by restricting the RLP-solution to $\underline{\mathcal {C}}$. Let $A_0>q_0$ be a real number to be specified later.

(i) The data on $\mathcal {C}$ is chosen so that $[\hat{f}^\pm ,\hat{\Omega },\hat{r}]$ coincide with the exact selfsimilar $g_{\text {RLP},\varepsilon }$ solution on the segment $\{(p,q) \ \Big | p= p_0, \ q\in [q_0,A_0]\}$;
(ii) $\hat{f}^\pm \in W^{2,\infty }(\mathcal {C}\cup \underline{\mathcal {C}})$, and $\hat{\Omega }, \frac{\hat{r}}{q}\in W^{3,\infty }(\underline{\mathcal {C}}\cup \mathcal {C})$ with ${\left| \left| \left| [f^\pm , \hat{\Omega }, \hat{r}]|_{\underline{\mathcal {C}}\cup \mathcal {C}} \right| \right| \right| } <\infty$ and $\hat{f}^\pm , \hat{\Omega }, \hat{r}>0$;
(iii) the constraint equations (2.85)–(2.86) hold on $\mathcal {C}$ and $\underline{\mathcal {C}}$ respectively.

Due to the choice of the double-null gauge (9.655)–(9.656), the metric coefficient $\hat{r}$ is determined along $\mathcal {C}$. In order to impose the constraint (2.85) we can solve it for $\hat{\Omega }$. By (9.656) we have $\partial _qr=\frac{1}{2}$ and by (2.89) and Remark 9.2 we may rewrite (2.85) as

9.665 $$\begin{aligned} \frac{1}{2}\partial _q(\Omega ^{-2}) = - \frac{\pi }{r}(f^+f^-)^{\frac{1}{k_+-k_-}}\left( \frac{f^-}{f^+}\right) ^{\frac{1}{2}} \Omega ^{-2}. \end{aligned}$$

Moving the copy of $\Omega ^{-2}$ to the left-hand side and then integrating, we obtain the formula

9.666 $$\begin{aligned} \Omega (p_0,q) = \Omega (p_0,q_0) \exp \left( \int _{q_0}^{q} \frac{2\pi }{s+2r_*} (f^+f^-)^{\frac{1}{k_+-k_-}}\left( \frac{f^-}{f^+}\right) ^{\frac{1}{2}} \,ds\right) . \end{aligned}$$

##### Remark 9.3

(The Hawking mass) We recall the Hawking mass introduced in (2.10). It can be alternatively expressed via the formula

9.667 $$\begin{aligned} m = \frac{r}{2} \left( 1+ \frac{4\partial _{p}r \partial _{q}r}{\Omega ^2}\right) . \end{aligned}$$

Using (2.83)–(2.86) one can show that for classical solutions of the Einstein-Euler system we have the identities

9.668 $$\begin{aligned} \partial _{p}m&= 2\pi r^2 \Omega ^{2}\left( T^{pq}\partial _{p}r - T^{qq}\partial _{q}r\right) , \end{aligned}$$

9.669 $$\begin{aligned} \partial _{q}m&= 2\pi r^2 \Omega ^{2}\left( T^{pq}\partial _{q}r - T^{pp}\partial _{p}r\right) , \end{aligned}$$

see for example Section 1.2 of [9]. Using (2.89) we may rewrite the right-hand side of (9.669) as $2\pi r^2 (1-\varepsilon )\rho \left( \partial _qr - (1+\eta )\Omega ^2 (u^{p})^2 \partial _{p} r\right)$. Integration along a constant $p$-slice then gives

9.670 $$\begin{aligned} m(p,q) = m(p,q_0) + 2\pi (1-\varepsilon ) \int _{q_0}^qr^2 \rho \left( \partial _qr - (1+\eta )\Omega ^2 (u^{p})^2 \partial _{p} r\right) \, ds. \end{aligned}$$

We now state the main local existence and uniqueness theorem for the characteristic problem described above.

##### Theorem 9.4

There exist sufficiently small $\delta _0>0$ such that for any $\delta \in (0,\delta _0)$ and $p_0=-\delta$, with initial boundary data satisfying *(i)-(iii)* above, there exists a unique asymptotically flat solution $[f^\pm ,\Omega ,r]$ to the system (2.83)–(2.84) and (9.648)–(9.649), with $f^\pm \in W^{2,\infty }(\overline{\mathcal {D}})$ , $[\Omega ,\frac{r}{q}]\in W^{3,\infty }(\overline{\mathcal {D}})$, and such that ${\left| \left| \left| [f^\pm , \Omega , r] \right| \right| \right| }<\infty$. Moreover, this solution is a solution of the original system (2.83)–(2.88).

We shall prove Theorem 9.4 by the method of characteristics. Our strategy is to first solve the fluid evolution equations for $f^\pm$ given the effective fluid velocity $\mathcal {U}$ and the metric components $\Omega ,r$. Then we will feed that back into the wave equations (2.83)–(2.84) for the metric components to obtain the bounds for $[r,\Omega ]$. To make this strategy work, in Section 9.3 we carefully look at the characteristics associated with the fluid evolution. After collecting some preparatory a priori bounds in Section 9.4, in Section 9.5 we use an iteration scheme to conclude the proof of Theorem 9.4.

### Characteristics for the Fluid Evolution

Let $\mathfrak {q}_\pm (s)=\mathfrak {q}_\pm (s; p,q)$ be the backward characteristics associated with the speeds $k_\pm \mathcal {U}$ such that

9.671 $$\begin{aligned} \frac{d \mathfrak {q}_\pm }{ds}(s; p,q)&= k_\pm \mathcal {U} (s, \mathfrak {q}_\pm (s;p,q)), \ \ s<p, \end{aligned}$$

9.672 $$\begin{aligned} \mathfrak {q}_\pm (p; p,q)&= q. \end{aligned}$$

Since our solutions as well as $\mathcal {U}$ reside in the domain $\mathcal {D}$ and the boundary, we track the backward characteristics $(s, \mathfrak {q}_\pm (s))$ until they leave the domain. In the next lemma, we show the existence and regularity properties of $\mathfrak {q}_\pm (s)$ and exit time $p_*=p_*(p,q)$ and position $q_*=q_*(p,q)$.

#### Lemma 9.5

Let $\ell \in \mathbb {N}$. Suppose $\mathcal {U}\in C^\ell (\overline{\mathcal {D}})$ or $W^{\ell ,\infty }(\overline{\mathcal {D}})$ and $\frac{1}{C_0}< \mathcal {U} < C_0$ on $\overline{\mathcal {D}}$ for some $C_0>1$. Then for any given $(p,q)\in \mathcal {D}$, there exist a unique exit time $p_*=p_*(p,q)$ and position $q_*=q_*(p,q)$ such that

1. A unique solution $\mathfrak {q}_\pm \in C^\ell ((p_*, p]; C^{\ell }( \mathcal {D}))$ or $C^\ell ((p_*, p]; W^{\ell ,\infty }( \mathcal {D}))$ of (9.671) and (9.672) exists so that $(s,\mathfrak {q}_\pm (s; p,q) )\in \mathcal {D}$.
2. At $s=p_*$, $(p_*, q_* ) \in \underline{\mathcal {C}}\cup \mathcal {C}$ where $q_*=\mathfrak {q}_\pm (p_*; p,q)$ and it satisfies
  9.673 $$\begin{aligned} q- q_* = \int _{p_*}^pk_\pm \mathcal {U} ( s, \mathfrak {q}_\pm ( s;p,q))\,d s. \end{aligned}$$
  If $$p_*>p_0$$ then $$q_*=q_0$$.
3. $$(p,q)\mapsto p_*(p,q)\in C(\mathcal {D})$$.
4. If $$p_*\ne p_0$$, $$p_* \in C^\ell (\mathcal {D})$$ or $$W^{\ell ,\infty }(\overline{\mathcal {D}})$$.

In particular, if $q>q_0 +k_\pm C_0 (p-p_0)$, then $(p_*,q_*) = (p_0,q_*) \in \mathcal {C}$.

#### Proof

We prove the claims for $\mathcal {U}\in C^\ell$ as the case of $\mathcal {U}\in W^{\ell ,\infty }$ follows in the same way. The local existence and uniqueness of $\mathfrak {q}_\pm \in C^\ell$ follows from $\mathcal {U}\in C^\ell$ via the Picard iteration

9.674 $$\begin{aligned} \mathfrak {q}_\pm (s) = q- \int _{s}^pk_\pm \mathcal {U} (\tilde{s}, \mathfrak {q}_\pm (\tilde{s};p,q))\,d\tilde{s}. \end{aligned}$$

Thanks to the positive uniform bound of $$\mathcal {U}$$ and Grönwall, the solution can be continued as long as the characteristics belong to the domain $$(s,\mathfrak {q}_\pm (s; p,q) )\in {\mathcal {D}}$$. Since $$ \mathcal {U}>\frac{1}{C_0}$$, $$\mathfrak {q}_\pm (s) < q - \frac{ k_\pm }{C_0} (p-s)$$, $$(s,\mathfrak {q}_\pm (s))$$ will exit the domain $$\mathcal {D}$$ either through the outgoing boundary $$\mathcal {C}$$ or through the ingoing boundary $$\underline{\mathcal {C}}$$. We denote such an exit time by $$p_*$$ and the associated value $$q_*=q_\pm (p_*; p,q)$$ where $$(p_*, q_* ) \in \underline{\mathcal {C}}\cup \mathcal {C}$$. Note that for each given $$(p,q)\in \mathcal {D}$$, $$(p_*,q_*)$$ is uniquely determined since the backward characteristics $$\mathfrak {q}_\pm $$ are unique. By integrating (9.671) from $$s=p$$ to $$s=p_*$$, we see that $$(p_*, q_* ) $$ satisfies (9.673). We observe that if $$(p_*, q_* ) \in \underline{\mathcal {C}}$$, $$q_*=q_0$$ and $$p_0\le p_*< p$$, and (9.673) reads as

9.675 $$\begin{aligned} q- q_0 = \int _{p_*}^pk_\pm \mathcal {U} (s, \mathfrak {q}_\pm (s;p,q)) ds. \end{aligned}$$

If $(p_*, q_* ) \in \mathcal {C}$, $p_*= p_0$ and $q_*$ is given by (9.673).

Clearly $p_*$ is continuous in $p$ and $q$. Since higher regularity of $p_*$ fails in general at the corner of the domain where $(p_*,q_*)=(p_0,q_0)$, we show the regularity when $p\ne p_0$. First let $(\tilde{p},\tilde{q})\in \mathcal {D}$ be given. Suppose $\tilde{p}_{*}= p_*(\tilde{p},\tilde{q})>p_0$. Consider a small neighborhood $\mathcal {B}$ of $(\tilde{p},\tilde{q})$ in $\mathcal {D}$ such that $I_1=p_*(\mathcal {B})$, $\inf I_1>p_0$. Recalling (9.675), we define an auxiliary function $H: I_1 \times \mathcal {B}\rightarrow \mathbb R$ by

$$\begin{aligned} H(\bar{p}_*,p,q) = q-q_0- \int _{\bar{p}_*}^pk_\pm \mathcal {U} (s, \mathfrak {q}_\pm (s;p,q)) ds . \end{aligned}$$

Then $H\in C^\ell$ since $\mathcal {U}\in C^\ell$, while we have $H(\tilde{p}_*, \tilde{p},\tilde{q})=0$ and $\partial _{\bar{p}_*} H (\tilde{p}_*,\tilde{p},\tilde{q}) =k_\pm \mathcal {U} (\tilde{p}_*,\tilde{q}_* ) >0$. Therefore, by the implicit function theorem, $p_*=p_*(p,q)\in C^\ell$ in a small neighborhood of $(\tilde{p},\tilde{q})$.

Lastly, since $\mathcal {U}<C_0$, if $q>q_0 +k_\pm C_0 (p-p_0)$, the backward characteristics will intersect the outgoing surface $\mathcal {C}$, in which case $p_*= p_0$. $\square$

### A Priori Bounds

In this subsection, we provide estimates for various quantities appearing the iteration scheme in terms of our norms. We will frequently use the following inequality: for any positive function $g>0$

9.676 $$\begin{aligned} \max \{\Vert g\Vert _\infty , \ \Vert g^{-1}\Vert _\infty \} \le e^{\Vert \log g \Vert _\infty } \end{aligned}$$

which directly follows from $g= e^{\log g}$ and $g^{-1}= e^{-\log g}$.

#### Lemma 9.6

Suppose ${\left| \left| \left| [\Omega , r] \right| \right| \right| }<\infty$. Then the following holds:

9.677 $$\begin{aligned} \sum _{j=0}^2\left\| q^{j+1}\partial _q^j\left( \frac{\partial _q\Omega }{\Omega } - \eta \frac{\partial _qr}{r}\right) \right\| _\infty \le M_1({\left| \left| \left| [\Omega , r] \right| \right| \right| }) , \end{aligned}$$

9.678 $$\begin{aligned} \sum _{j=1}^2 \left\| q^{j+3}\partial _q^j\left( \frac{\Omega ^2}{r^3}\right) \right\| _\infty \le M_2({\left| \left| \left| [\Omega , r] \right| \right| \right| }), \end{aligned}$$

where $M_1$ and $M_2$ are continuous functions of their arguments.

#### Proof

We start with (9.677). For $j=0$, it is easy to see that

9.679 $$\begin{aligned} \left\| q\left( \frac{\partial _q\Omega }{\Omega } - \eta \frac{\partial _qr}{r}\right) \right\| _\infty \le \frac{1}{q_0^\theta } \Vert q^{1+\theta } \partial _q\log \Omega \Vert _\infty + \frac{\eta }{2} \left\| \frac{q^2}{r^2}\right\| _\infty \left\| \frac{\partial _q(r^2)}{q}\right\| _\infty \end{aligned}$$

where the right-hand side is a continuous function of ${\left| \left| \left| [\Omega , r] \right| \right| \right| }$ by (9.676). For $j=1$, since

$$\partial _q\left( \frac{\partial _q\Omega }{\Omega } - \eta \frac{\partial _qr}{r} \right) = \partial _q^2\log \Omega - \frac{\eta }{2} \frac{\partial _q^2(r^2)}{r^2} + \frac{\eta }{2}\frac{(\partial _q(r^2))^2}{r^4} $$

we have

9.680 $$\begin{aligned}{} & {} \left\| q^{2}\partial _q\left( \frac{\partial _q\Omega }{\Omega } - \eta \frac{\partial _qr}{r}\right) \right\| _\infty \le \frac{1}{q_0^\theta }\Vert q^{2+\theta } \partial _q^{2}\log \Omega \Vert _\infty \nonumber \\{} & {} \quad + \frac{\eta }{2} \left\| \frac{q^2}{r^2}\right\| _\infty \Vert \partial _q^2( r^2)\Vert _\infty +\frac{ \eta }{2}\left\| \frac{q^2}{r^2}\right\| _\infty ^2 \left\| \frac{\partial _q(r^2)}{q}\right\| _\infty ^2 \end{aligned}$$

which shows (9.677). Lastly, from

$$\begin{aligned} \partial _q^2 \left( \frac{\partial _q\Omega }{\Omega } - \eta \frac{\partial _qr}{r} \right) = \partial _q^3\log \Omega - \frac{\eta }{2} \frac{\partial _q^3(r^2)}{r^2} + \frac{3\eta }{2}\frac{\partial _q(r^2)\partial _q^2( r^2)}{r^4}- \eta \frac{(\partial _q(r^2))^3}{r^6} \end{aligned}$$

we obtain

9.681 $$\begin{aligned} \begin{aligned} \left\| q^{3}\partial _q^2\left( \frac{\partial _q\Omega }{\Omega } - \eta \frac{\partial _qr}{r}\right) \right\| _\infty&\le \frac{1}{q_0^\theta }\Vert q^{3+\theta } \partial _q^{3}\log \Omega \Vert _\infty + \frac{\eta }{2} \left\| \frac{q^2}{r^2}\right\| _\infty \Vert q\partial _q^3( r^2)\Vert _\infty \\&\quad +\frac{3\eta }{2}\left\| \frac{q^2}{r^2}\right\| _\infty ^2 \left\| \frac{\partial _q(r^2)}{q}\right\| _\infty ^2 \Vert \partial _q^2( r^2)\Vert _\infty + \frac{\eta }{2} \left\| \frac{q^2}{r^2}\right\| _\infty ^3 \left\| \frac{\partial _q(r^2)}{q}\right\| _\infty ^3. \end{aligned} \end{aligned}$$

This completes the proof of (9.677). The estimation of (9.678) follows similarly, we omit the details. $\square$

#### Lemma 9.7

($\mathcal {U}$ bounds) Suppose ${\left| \left| \left| [f^\pm , \Omega , r] \right| \right| \right| }<\infty$. Then the following holds:

9.682 $$\begin{aligned} \Vert \mathcal {U}\Vert _\infty + \Vert \mathcal {U}^{-1}\Vert _\infty + \sum _{j=1}^2\Vert q^{j}\partial _q^j \mathcal {U}\Vert _{\infty } \le M_3 ({\left| \left| \left| [f^\pm , \Omega , r] \right| \right| \right| }) \end{aligned}$$

where $M_3$ is a continuous function of its argument.

#### Proof

From the first condition of our choice $N_\pm$ in (9.660), we may rewrite $\mathcal {U}$ as

$$\begin{aligned} \mathcal {U}=(1+\eta )^2\Omega ^2 \left( \frac{q}{r}\right) ^{2\eta } \left( \frac{q^{N_+}f^+}{q^{N_-}f^-} \right) ^\frac{1}{2}. \end{aligned}$$

Then by (9.676), we see that

9.683 $$\begin{aligned} \Vert \mathcal {U}^{\pm 1}\Vert _\infty \le (1+\eta )^{\pm 2}\left\| \frac{q^2}{r^2}\right\| ^{\pm \eta }_\infty e^{2\Vert \log \Omega \Vert _\infty + \frac{1}{2} \Vert \log (q^{N_+}f^+) \Vert _\infty +\frac{1}{2} \Vert \log (q^{N_-}f^-) \Vert _\infty } \end{aligned}$$

which shows (9.682) for $\Vert \mathcal {U}\Vert _\infty + \Vert \mathcal {U}^{-1}\Vert _\infty$. Next computing $\partial _q\mathcal {U}$ as

9.684 $$\begin{aligned} \partial _q\mathcal {U} = \left( 2\left( \frac{\partial _q\Omega }{\Omega } - \eta \frac{\partial _qr}{r}\right) + \frac{1}{2} \partial _q\log f^+ - \frac{1}{2}\partial _q\log f^- \right) \mathcal U \end{aligned}$$

we obtain

9.685 $$\begin{aligned} \Vert q\partial _q\mathcal {U}\Vert _\infty \le \left( 2\left\| q\left( \frac{\partial _q\Omega }{\Omega } - \eta \frac{\partial _qr}{r}\right) \right\| _\infty +\frac{1}{2}\Vert q\partial _q\log f^+\Vert _\infty +\frac{1}{2}\Vert q\partial _q\log f^-\Vert _\infty \right) \Vert \mathcal {U}\Vert _\infty . \end{aligned}$$

Together with (9.679), it implies (9.682) for $j=1$. Moreover, since

9.686 $$\begin{aligned} \partial _q^2 \mathcal {U} = \left( 2\partial _q\left( \frac{\partial _q\Omega }{\Omega } - \eta \frac{\partial _qr}{r}\right) + \frac{1}{2} \partial _q^2\log f^+ - \frac{1}{2}\partial _q^2\log f^- \right) \mathcal U + \frac{(\partial _q\mathcal {U})^2}{\mathcal {U}} \end{aligned}$$

we have

9.687 $$\begin{aligned} \begin{aligned} \Vert q^2\partial _q^2 \mathcal {U}\Vert _\infty&\le \Big (2\left\| q^{2}\partial _q\left( \frac{\partial _q\Omega }{\Omega } - \eta \frac{\partial _qr}{r}\right) \right\| _\infty +\frac{1}{2}\Vert q^2\partial _q^2\log f^+\Vert _\infty +\frac{1}{2}\Vert q^2\partial _q^2\log f^-\Vert _\infty \Big ) \Vert \mathcal {U}\Vert _\infty \\&\qquad + \Vert \mathcal {U}^{-1}\Vert _\infty \Vert q\partial _q\mathcal {U}\Vert _\infty ^2. \end{aligned} \end{aligned}$$

Using (9.680) and (9.685), we deduce (9.682), where we recall (9.661). $\square$

#### Remark 9.8

The relation $N_+ = N_- - 4\eta$ in (9.660) is importantly used to ensure the boundedness (both upper and lower) of the transport speed $\mathcal {U}$.

We introduce the constant $\beta >0$:

9.688 $$\begin{aligned} \beta {:}{=}2\eta + \frac{N_++N_-}{k_+-k_-} - 1=2\eta + \frac{2N_-- (k_+-k_- +4\eta )}{k_+-k_-} >0, \end{aligned}$$

where we have used (9.660) in the second equality.

#### Lemma 9.9

Suppose ${\left| \left| \left| [f^\pm , \Omega , r] \right| \right| \right| }<\infty$. Then the following bounds hold:

9.689 $$\begin{aligned} \sum _{j=0}^2\left\| q^{j+1+\beta }\partial _q^j\left( \frac{\Omega ^2}{r^{2\eta }} (f^+f^-) ^{\frac{1}{k_+-k_-}}\right) \right\| _\infty \le M_4({\left| \left| \left| [f^\pm , \Omega , r] \right| \right| \right| }), \end{aligned}$$

9.690 $$\begin{aligned} \sum _{j=0}^2\left\| q^{j+3+\beta }\partial _q^j\left( \frac{\Omega ^2}{r^{2+2\eta }} (f^+f^-) ^{\frac{1}{k_+-k_-}}\right) \right\| _\infty \le M_5 ({\left| \left| \left| [f^\pm , \Omega , r] \right| \right| \right| }), \end{aligned}$$

where $M_4$ and $M_5$ are continuous functions of their arguments.

#### Proof

We will first prove (9.689). We start with $j=0$. Using (9.688), we rewrite $\mathfrak H{:}{=}\frac{\Omega ^2}{r^{2\eta }} (f^+f^-) ^{\frac{1}{k_+-k_-}}$ as

$$\begin{aligned} q^{1+\beta }\mathfrak H= q^{1+\beta }\frac{\Omega ^2}{r^{2\eta }} (f^+f^-) ^{\frac{1}{k_+-k_-}} = \Omega ^2\left( \frac{q}{r}\right) ^{2\eta } \left( q^{N_+}f^+q^{N_-}f^-\right) ^{\frac{1}{k_+-k_-}} \end{aligned}$$

from which we have

9.691 $$\begin{aligned} \left\| q^{1+\beta } \mathfrak H\right\| _\infty \le \Vert \Omega \Vert ^2_\infty \left\| \frac{q^2}{r^2}\right\| _\infty ^{\eta }(\Vert q^{N_+} f^+\Vert _\infty \Vert q^{N_-} f^-\Vert _\infty )^{\frac{1}{k_+-k_-}} . \end{aligned}$$

By (9.676), the claim immediately follows. We next compute

9.692 $$\begin{aligned} \partial _q\mathfrak H =\left( 2\left( \frac{\partial _q\Omega }{\Omega } - \eta \frac{\partial _qr}{r}\right) + \frac{\partial _q\log f^+ + \partial _q\log f^-}{k_+-k_-} \right) \mathfrak H \end{aligned}$$

and obtain

9.693 $$\begin{aligned} \Vert q^{2+\beta }\partial _q\mathfrak H \Vert _\infty \le \left( 2\left\| q\left( \frac{\partial _q\Omega }{\Omega } - \eta \frac{\partial _qr}{r}\right) \right\| _\infty +\frac{\Vert q\partial _q\log f^+\Vert _\infty +\Vert q\partial _q\log f^-\Vert _\infty }{k_+-k_-} \right) \Vert q^{1+\beta }\mathfrak H \Vert _\infty . \end{aligned}$$

With (9.679) and (9.691), it gives (9.689) for $j=1$. We next have

9.694 $$\begin{aligned} \begin{aligned} \partial _q^2 \mathfrak H&= \left( 2\partial _q\left( \frac{\partial _q\Omega }{\Omega } - \eta \frac{\partial _qr}{r}\right) + \frac{\partial _q^2 \log f^+ + \partial _q^2\log f^-}{k_+-k_-} \right) \mathfrak H \\&\quad + \left( 2\left( \frac{\partial _q\Omega }{\Omega } - \eta \frac{\partial _qr}{r}\right) + \frac{\partial _q\log f^+ + \partial _q\log f^-}{k_+-k_-} \right) \partial _q\mathfrak H, \end{aligned} \end{aligned}$$

and therefore

9.695 $$\begin{aligned} \begin{aligned} \Vert q^{3+\beta }\partial _q^2 \mathfrak H\Vert _\infty&\le \Big (2\left\| q^{2}\partial _q\left( \frac{\partial _q\Omega }{\Omega } - \eta \frac{\partial _qr}{r}\right) \right\| _\infty +\frac{\Vert q^2\partial _q^2\log f^+\Vert _\infty +\Vert q^2\partial _q^2\log f^-\Vert _\infty }{k_+-k_-} \Big ) \Vert q^{1+\beta } \mathfrak H\Vert _\infty \\&+ \left( 2\left\| q\left( \frac{\partial _q\Omega }{\Omega } - \eta \frac{\partial _qr}{r}\right) \right\| _\infty +\frac{\Vert q\partial _q\log f^+\Vert _\infty +\Vert q\partial _q\log f^-\Vert _\infty }{k_+-k_-} \right) \Vert q^{2+\beta }\partial _q\mathfrak H \Vert _\infty . \end{aligned} \end{aligned}$$

Hence the claim follows from (9.691), (9.693) and (9.677).

The proof of (9.690) follows easily from (9.689) by applying the product rule for $\frac{\Omega ^2}{r^{2+2\eta }} (f^+f^-) ^{\frac{1}{k_+-k_-}}= r^{-2}\mathfrak H$ or by estimating them directly in the same way as done for (9.689). We omit the details. $\square$

In the iteration scheme, we will make use of the Hawking mass *m* given in (9.667). From (9.669) and (2.82) we see that the Hawking mass satisfies

9.696 $$\begin{aligned} \begin{aligned} \partial _qm&= 2\pi r^2 \left[ (1-\varepsilon )\rho \partial _qr - (1+\varepsilon ) \Omega ^{-2}\rho (u^{q})^{-2}\partial _{p}r\right] \\&= 2\pi (f^+f^-)^\frac{1}{k_+-k_-} \left[ \frac{1 }{r^{2\eta }} \partial _qr - \Omega ^{-2} \left( \frac{f^-}{f^+}\right) ^\frac{1}{2} \partial _{p}r\right] . \end{aligned} \end{aligned}$$

We observe that *p*-derivatives are not featured in our function space hierarchy, however $\partial _{p}r$ appears in the above expression. To go around this difficulty, we observe that (2.83) can be formally rewritten in the form

9.697 $$\begin{aligned} \partial _{pq}(r^2) = -\frac{\Omega ^2}{2}+2\pi r^2 \Omega ^4 T^{pq} = -\frac{\Omega ^2}{2} + \frac{2\pi \Omega ^2 }{r^{2\eta } } (f^+ f^-)^{\frac{1}{k_+-k_-}}, \end{aligned}$$

where we have used (2.89) and (9.653) in the second equality. Therefore, rather than directly estimating *m* from (9.667), we slightly abuse notation, and redefine *m* to be

9.698 $$\begin{aligned} m(p,q) {:}{=} m(p,q_0)+ \int ^{q}_{q_0} \left( 2\pi (f^+f^-)^\frac{1}{k_+-k_-} \left[ \frac{1 }{r^{2\eta }} \partial _qr - \frac{ \Omega ^{-2} }{2r} \left( \frac{f^-}{f^+}\right) ^\frac{1}{2} \mathfrak s\right] \right) d\tilde{q} \end{aligned}$$

where

9.699 $$\begin{aligned} m(p,q_0)&{:}{=} \frac{\hat{r}}{2} \left( 1+ \frac{4\partial _{p}\hat{r} \partial _{q}\hat{r}}{\hat{\Omega }^2}\right) \Big |_{(p,q_0)}, \end{aligned}$$

9.700 $$\begin{aligned} \mathfrak s(p,q)&{:}{=} \partial _p(\hat{r}^2) \Big |_{(p,q_0)} + \int _{q_0}^q\left[ - \frac{\Omega ^2}{2} + \frac{2\pi \Omega ^2 }{r^{2\eta } } (f^+ f^-)^{\frac{1}{k_+-k_-}} \right] d\tilde{q}. \end{aligned}$$

We observe that $\partial _{p}\hat{r}$ is well-defined at $(p,q_0)$ since the data there is given by the exact selfsimilar RLP-solution. We note that $\mathfrak s$ corresponds exactly to $\partial _{p}(r^2)$ for a $C^2$-solution of the problem. In the following, we show that *m* in (9.698) can be estimated by using the norm ${\left| \left| \left| [f^\pm , \Omega , r] \right| \right| \right| }$.

#### Lemma 9.10

Suppose ${\left| \left| \left| [f^\pm , \Omega , r] \right| \right| \right| }<\infty$. Then the following holds:

9.701 $$\begin{aligned} \Vert m\Vert _\infty&\le {\left| \left| \left| [ \Omega , r]|_{\underline{\mathcal {C}}\cup \mathcal {C}} \right| \right| \right| } + M_6 ( {\left| \left| \left| [f^\pm , \Omega , r] \right| \right| \right| }), \end{aligned}$$

9.702 $$\begin{aligned} \sum _{j=1}^2\Vert q^{j+\beta } \partial _q^j m \Vert _\infty&\le M_7 ({\left| \left| \left| [f^\pm , \Omega , r] \right| \right| \right| }), \end{aligned}$$

where $M_6$ and $M_7$ are continuous functions of its argument.

#### Proof

We first observe that from (9.700) using $q>q_0$ and $\frac{q-q_0}{q}<1$

9.703 $$\begin{aligned} \Vert q^{-1} \mathfrak s\Vert _\infty \le \left\| \frac{\partial _p(\hat{r}^2)}{q}\Big |_{(p,q_0)}\right\| _\infty + \frac{\Vert \Omega \Vert _\infty ^2}{2} +\frac{2\pi }{q_0^{1+\beta }} \left\| q^{1+\beta } \frac{ \Omega ^2 }{r^{2\eta } } (f^+ f^-)^{\frac{1}{k_+-k_-}} \right\| _\infty \end{aligned}$$

and

9.704 $$\begin{aligned} \Vert \partial _q\mathfrak s \Vert _\infty \le \frac{\Vert \Omega \Vert _\infty ^2}{2} +\frac{2\pi }{q_0^{1+\beta }} \left\| q^{1+\beta } \frac{ \Omega ^2 }{r^{2\eta } } (f^+ f^-)^{\frac{1}{k_+-k_-}} \right\| _\infty \end{aligned}$$

From the definition of the boundary data norm, we have $|m(p,q_0)|\le {\left| \left| \left| [ \Omega , r]|_{\underline{\mathcal {C}}\cup \mathcal {C}} \right| \right| \right| }$. For the integral term in (9.698), using (9.688) and (9.660), we rewrite the integrand as

9.705 $$\begin{aligned} \begin{aligned} q^{-1-\beta }(q^{N_+}f^+q^{N_-} f^-)^\frac{1}{k_+-k_-} \left[ \frac{q^{2\eta } }{r^{2\eta }} \partial _qr - \Omega ^{-2} \frac{q}{2r} \left( \frac{q^{N_-} f^-}{q^{N_+}f^+}\right) ^\frac{1}{2} \frac{\mathfrak s}{q}\right] \end{aligned} \end{aligned}$$

so that

$$\begin{aligned} \begin{aligned}&\left| \int ^{q}_{q_0 } \left( 2\pi (f^+f^-)^\frac{1}{k_+-k_-} \left[ \frac{1 }{r^{2\eta }} \partial _qr - \frac{ \Omega ^{-2} }{2r} \left( \frac{f^-}{f^+}\right) ^\frac{1}{2}\mathfrak s\right] \right) d\tilde{q} \right| \\&\le \frac{1}{\beta q_0^\beta } (\Vert q^{N_+}f^+\Vert _\infty \Vert q^{N_-} f^-\Vert _\infty )^\frac{1}{k_+-k_-} \\&\quad \cdot \left[ \frac{1}{2}\Vert \frac{q}{r}\Vert ^{2\eta +1}_\infty \Vert q^{-1}\partial _q( r^2)\Vert _\infty + \frac{1}{2}\Vert \Omega ^{-1}\Vert ^2_\infty \Vert \frac{q}{r}\Vert _\infty \Vert q^{N_-} f^-\Vert _\infty ^\frac{1}{2} \Vert (q^{N_+}f^+)^{-1}\Vert _\infty ^\frac{1}{2} \Vert q^{-1} \mathfrak s\Vert _\infty \right] \end{aligned} \end{aligned}$$

where we have used $\int _{q_0 }^q\tilde{q}^{-1-\beta } d\tilde{q} \le \frac{1}{\beta q_0^\beta }$. Hence, using (9.676), we deduce (9.701). Moreover since

9.706 $$\begin{aligned} \partial _qm= 2\pi (f^+f^-)^\frac{1}{k_+-k_-} \left[ \frac{1 }{r^{2\eta }} \partial _qr - \frac{ \Omega ^{-2} }{2r} \left( \frac{f^-}{f^+}\right) ^\frac{1}{2}\mathfrak s\right] \end{aligned}$$

from (9.705), we immediately obtain

9.707 $$\begin{aligned} \begin{aligned} \Vert q^{1+\beta } \partial _qm\Vert _\infty&\le 2\pi (\Vert q^{N_+}f^+\Vert _\infty \Vert q^{N_-} f^-\Vert _\infty )^\frac{1}{k_+-k_-} \\&\cdot \left[ \frac{1}{2}\Vert \frac{q}{r}\Vert ^{2\eta +1}_\infty \Vert q^{-1}\partial _q( r^2)\Vert _\infty + \frac{ 1}{2}\Vert \Omega ^{-1}\Vert _\infty ^2 \Vert \frac{q}{r}\Vert _\infty \Vert q^{N_-} f^-\Vert _\infty ^\frac{1}{2} \Vert (q^{N_+}f^+)^{-1}\Vert _\infty ^\frac{1}{2} \Vert q^{-1} \mathfrak s\Vert _\infty \right] \end{aligned} \end{aligned}$$

which shows (9.702) for $j=1$. Lastly, we compute $\partial _q^2m$ as

9.708 $$\begin{aligned} \begin{aligned}&\partial _q^2 m= \pi (f^+f^-)^\frac{1}{k_+-k_-} \left[ \frac{1 }{r^{2\eta +1}} \partial _q^2 (r^2) - \frac{\Omega ^{-2} }{r} \left( \frac{f^-}{f^+}\right) ^\frac{1}{2}\partial _q\mathfrak s\right] \\&+ \pi \frac{ (f^+f^-)^\frac{1}{k_+-k_-}}{r^{2\eta }} \frac{\partial _q(r^2)}{r} \left( \frac{\partial _q\log f^+ + \partial _q\log f^-}{k_+-k_-} - \frac{2\eta +1}{2}\frac{\partial _q(r^2)}{r^2}\right) \\&- \pi \Omega ^{-2} (f^+f^-)^\frac{1}{k_+-k_-} \left( \frac{f^-}{f^+}\right) ^\frac{1}{2} \frac{\mathfrak s}{r} \left( -2\partial _q\log \Omega + c_1 \partial _q\log f^+ + c_2\partial _q\log f^+ -\frac{1}{2}\frac{\partial _q(r^2)}{r^2} \right) \end{aligned} \end{aligned}$$

where $c_1=\frac{1}{k_+-k_-} -\frac{1}{2}$ and $c_2= \frac{1}{k_+-k_-} +\frac{1}{2}$. Following the same strategy, it is now clear that $\Vert q^{2+\beta }\partial _q^2m\Vert _\infty$ is bounded by a continuous function of ${\left| \left| \left| [f^\pm , \Omega , r] \right| \right| \right| }$. This finishes the proof. $\square$

### Proof of the Local Well-Posedness

#### Iteration Scheme

We now set up the iteration scheme. Let $[f^\pm _n,\Omega _n,r_n]$ be given so that ${\left| \left| \left| [f^\pm _n, \Omega _n, r_n] \right| \right| \right| }<\infty$. And let $\mathcal {U}_n$ and $m_n$ be given in (9.654) and (9.698) where $[f,\Omega ,r]=[f^\pm _n,\Omega _n,r_n]$:

$$\begin{aligned} \mathcal {U}_n= (1+\eta )^2 \frac{(\Omega _n)^2}{(r_n)^{2\eta }} \left( \frac{f^+_n}{f^-_n} \right) ^\frac{1}{2} \end{aligned}$$

and

$$\begin{aligned} m_n= m(p,q_0)+ \int ^{q}_{q_0} \left( 2\pi (f_n^+f_n^-)^\frac{1}{k_+-k_-} \left[ \frac{1 }{(r_n)^{2\eta }} \partial _qr_n - \frac{1}{2}\frac{(\Omega _n)^{-2} }{(r_n)} \left( \frac{f^-_n}{f_n^+}\right) ^\frac{1}{2} \mathfrak s_n\right] \right) d\tilde{q} . \end{aligned}$$

Here $\mathfrak s_n$ is given by the right-hand side of (9.700) where $[f^\pm ,\Omega ,r]=[f^\pm _n,\Omega _n,r_n]$. We then define $[f^\pm _{n+1},\Omega _{n+1},r_{n+1}]$ to be the solution of the following system

9.709 $$\begin{aligned}&\partial _pf^\pm _{n+1} + k_\pm \mathcal {U}_n \partial _qf^\pm _{n+1} \pm 2k_\pm (2\frac{\partial _q\Omega _n}{\Omega _n} - 2\eta \frac{\partial _qr_n}{r_n}) \mathcal {U}_n f^\pm _{n+1} =0, \end{aligned}$$

9.710 $$\begin{aligned}&\partial _p\partial _q[(r_{n+1})^2]= - \frac{(\Omega _n)^2}{2} + 2\pi \frac{(\Omega _n)^2}{(r_n)^{2\eta }} (f^+_nf^-_n) ^{\frac{1}{k_+-k_-}}, \end{aligned}$$

9.711 $$\begin{aligned}&\partial _p\partial _q\log {\Omega _{n+1}} = \frac{ (\Omega _n)^2}{2 (r_n)^2} \frac{m_n}{r_n} - (1+\eta ) \pi \frac{ (\Omega _n)^2 }{(r_n)^{2+2\eta }}(f^+_n f^-_n)^{\frac{1}{k_+-k_-}}, \end{aligned}$$

with given characteristic data $[f^\pm _{n+1},\Omega _{n+1},r_{n+1}]= [\hat{f}^\pm , \hat{\Omega }, \hat{r}]|_{\underline{\mathcal {C}}\cup \mathcal {C}}$ satisfying the conditions (i)–(iii) as in Theorem 9.4. We note that for exact solutions, (9.710)-(9.711) are equivalent to (2.83), (2.84).

Our next goal is to prove the solvability of the iterative system (9.709)-(9.711) and derive the uniform bounds of ${\left| \left| \left| [f^\pm _{n+1},\Omega _{n+1},r_{n+1}] \right| \right| \right| }$ for sufficiently small $\delta >0$. Let $A{:}{=} \max \{4, 1+ \frac{1}{\theta q_0^\theta }\}$.

##### Proposition 9.11

Let the characteristic data $[\hat{f}^{\pm }, \hat{\Omega }, \hat{r}]$ satisfying the assumptions of Theorem 9.4 be given. Suppose ${\left| \left| \left| [f^\pm _{n},\Omega _{n},r_{n}] \right| \right| \right| }\le 2A{\left| \left| \left| [\hat{f}^\pm , \hat{\Omega }, \hat{r}]|_{\underline{\mathcal {C}}\cup \mathcal {C}} \right| \right| \right| }$. Then there exist sufficiently small $\delta _0>0$ depending only on ${\left| \left| \left| [\hat{f}^\pm , \hat{\Omega }, \hat{r}]|_{\underline{\mathcal {C}}\cup \mathcal {C}} \right| \right| \right| }$ such that for all $\delta \in (0,\delta _0)$ there exists a unique solution $f^\pm _{n+1} \in W^{2,\infty }(\overline{\mathcal {D}})$, $[\Omega _{n+1}, \frac{r_{n+1}}{q}]\in W^{3,\infty }(\overline{\mathcal {D}})$ to (9.709)-(9.711) with $[f_{n+1}^{\pm } ,\Omega _{n+1},r_{n+1}]= [\hat{f}^{\pm }, \hat{\Omega }, \hat{r}]$ on $\underline{\mathcal {C}}\cup \mathcal {C}$ satisfying

9.712 $$\begin{aligned} {\left| \left| \left| [f^\pm _{n+1},\Omega _{n+1},r_{n+1}] \right| \right| \right| }\le 2A{\left| \left| \left| [\hat{f}^\pm , \hat{\Omega }, \hat{r}]|_{\underline{\mathcal {C}}\cup \mathcal {C}} \right| \right| \right| } . \end{aligned}$$

Proposition 9.11 immediately follows from the following two lemmas.

##### Lemma 9.12

(Solving the Euler part) Assume the same as in Proposition 9.11. Then there exist sufficiently small $\delta _0>0$ depending only on ${\left| \left| \left| [\hat{f}^\pm , \hat{\Omega }, \hat{r}]|_{\underline{\mathcal {C}}\cup \mathcal {C}} \right| \right| \right| }$ such that for all $\delta \in (0,\delta _0)$ there exists a unique solution $f^\pm _{n+1} \in W^{2,\infty }(\overline{\mathcal {D}})$ to (9.709) with $f_{n+1}^{\pm } = \hat{f}^{\pm }$ on $\underline{\mathcal {C}}\cup \mathcal {C}$ satisfying

9.713 $$\begin{aligned} {\left| \left| \left| [f^\pm _{n+1}] \right| \right| \right| }\le 2{\left| \left| \left| [\hat{f}^\pm ]|_{\underline{\mathcal {C}}\cup \mathcal {C}} \right| \right| \right| } + A {\left| \left| \left| [\hat{f}^\pm , \hat{\Omega }, \hat{r}]|_{\underline{\mathcal {C}}\cup \mathcal {C}} \right| \right| \right| } . \end{aligned}$$

##### Lemma 9.13

(Solving the metric part) Assume the same as in Proposition 9.11. Then there exist sufficiently small $\delta _0>0$ depending only on ${\left| \left| \left| [\hat{f}^\pm , \hat{\Omega }, \hat{r}]|_{\underline{\mathcal {C}}\cup \mathcal {C}} \right| \right| \right| }$ such that for all $\delta \in (0,\delta _0)$ there exists a unique solution $[\Omega _{n+1}, \frac{r_{n+1}}{q}]\in W^{3,\infty }(\overline{\mathcal {D}})$ to (9.710)-(9.711) with $[\Omega _{n+1},r_{n+1}]= [ \hat{\Omega }, \hat{r}]$ on $\underline{\mathcal {C}}\cup \mathcal {C}$ satisfying

9.714 $$\begin{aligned} {\left| \left| \left| [\Omega _{n+1},r_{n+1}] \right| \right| \right| }\le A{\left| \left| \left| [\hat{\Omega }, \hat{r}]|_{\underline{\mathcal {C}}\cup \mathcal {C}} \right| \right| \right| } +A {\left| \left| \left| [\hat{f}^\pm , \hat{\Omega }, \hat{r}]|_{\underline{\mathcal {C}}\cup \mathcal {C}} \right| \right| \right| } . \end{aligned}$$

In what follows, we prove the above two lemmas. We start with Lemma 9.12.

##### Proof

(Proof of Lemma 9.12 (Solving the Euler part).) For the sake of notational convenience, throughout the proof, we use $\mathcal {U}=\mathcal {U}_n$, $\Omega =\Omega _n$, $r=r_n$, $f^\pm =f_n^\pm$ so that the variables without the indices refer to the ones from the *n*-th step.

Uniqueness follows from the uniqueness of characteristics since $W^{2,\infty }\subset C^1$ solutions $f_{n+1}^\pm >0$ satisfy the ODE along the characteristics

9.715 $$\begin{aligned} \frac{d}{dp} \log f_{n+1}^\pm (p, \mathfrak {q}_\pm ) = \mp 2k_\pm (2\frac{\partial _q\Omega }{\Omega } - 2\eta \frac{\partial _qr}{r}) \mathcal {U} (p, \mathfrak {q}_\pm ) . \end{aligned}$$

The existence follows by the integral representation of (9.715)

9.716 $$\begin{aligned} f_{n+1}^\pm (p, q) = \hat{f}^{\pm }_*(p,q) \exp \left\{ \mp \int _{p_*}^p2 k_\pm \left[ (2\frac{\partial _q\Omega }{\Omega } - 2\eta \frac{\partial _qr}{r}) \mathcal {U} \right] (s, \mathfrak {q}_\pm (s)) ds \right\} \end{aligned}$$

where $\hat{f}^{\pm }_*(p,q) = \hat{f}^{\pm }(p_*, q_*),$ and $(p_*,q_*)$ is the exit time and position associated with $(p,q)$ constructed in Lemma 9.5. We focus on verifying the desired regularity and estimates.

First of all, clearly $f_{n+1}^\pm \in C(\overline{\mathcal {D}})$ since $p_*$ is continuous. To estimate $\Vert \log (q^{N_\pm } f^\pm _{n+1})\Vert _\infty$, we take log of (9.716) and rewrite it as

9.717 $$\begin{aligned} \log (q^{N_\pm } f^\pm _{n+1}) = \log (q^{N_\pm } \hat{f}^{\pm }_*) \mp \int _{p_*}^p2 k_\pm \left[ (2\frac{\partial _q\Omega }{\Omega } - 2\eta \frac{\partial _qr}{r}) \mathcal {U} \right] (s, \mathfrak {q}_\pm (s)) ds. \end{aligned}$$

For the first term, we may write it as

$$\begin{aligned} \log (q^{N_\pm } \hat{f}^{\pm }_*) = \log (q_*^{N_\pm } \hat{f}^{\pm }_*) + N_\pm \log \left( \frac{q}{q_*}\right) . \end{aligned}$$

Since $| \log (q_*^{N_\pm } \hat{f}^{\pm }_*)|\le \Vert \log (q^{N_\pm } f^\pm ) |_{\underline{\mathcal {C}}\cup \mathcal {C}} \Vert _\infty$, it suffices to estimate $\log (\frac{q}{q_*})$. To this end, first let $p_*= p_0$. Then we have

$$\begin{aligned} q_*=\mathfrak {q}_\pm (p_0;p,q) = q- \int _{p_0}^pk_\pm \mathcal {U} (s) ds > q- k_\pm \Vert \mathcal {U}\Vert _\infty (p-p_0)\ge q- k_\pm \Vert \mathcal {U}\Vert _\infty \delta . \end{aligned}$$

In particular, using $q\ge q_0$, we have $q_* \ge \left( 1-k_\pm \Vert \mathcal {U}\Vert _\infty q_0^{-1}\delta \right) q$. Hence,

9.718 $$\begin{aligned} | \log \left( \frac{q}{q_*}\right) | \le - \log (1-k_\pm \Vert \mathcal {U}\Vert _\infty q_0^{-1}\delta ) \le q_0^{-1}\delta k_\pm \Vert \mathcal {U}\Vert _\infty \end{aligned}$$

for sufficiently small $\delta >0$. If $p_*>p_0$, we have $q_*=q_0$ and $q<q_0 + k_\pm \Vert \mathcal {U}\Vert _\infty (p-p_0)$. Hence in this case, for sufficiently small $\delta >0$,

9.719 $$\begin{aligned} | \log \left( \frac{q}{q_*}\right) | \le \log (1+ k_\pm \Vert \mathcal {U}\Vert _\infty q_0^{-1}\delta )\le k_\pm \Vert \mathcal {U}\Vert _\infty q_0^{-1}\delta . \end{aligned}$$

We next estimate the integral term in (9.717). Using $\mathfrak {q}_\pm (s) \ge q_*$ for all $p_*\le s\le p$, and $p-p_* \le \delta$,

9.720 $$\begin{aligned} \begin{aligned}&\left| \int _{p_*}^p2 k_\pm \left[ (2\frac{\partial _q\Omega }{\Omega } - 2\eta \frac{\partial _qr}{r}) \mathcal {U} \right] (s, \mathfrak {q}_\pm (s)) ds \right| \\&\quad = \left| 4k_\pm \int _{p_*}^p\frac{1}{\mathfrak {q}_\pm } \left[ \mathfrak {q}_\pm (\frac{ \partial _q\Omega }{\Omega } - \eta \frac{\partial _qr}{r}) \mathcal {U} \right] (s, \mathfrak {q}_\pm (s)) ds \right| \\&\quad \le 4k_\pm \Vert \mathcal {U}\Vert _\infty \left\| \frac{q\partial _q\Omega }{\Omega } - \eta \frac{q\partial _qr}{r} \right\| _\infty \frac{p-p_*}{q_*}\\&\quad \le 4k_\pm \Vert \mathcal {U}\Vert _\infty \left\| \frac{q\partial _q\Omega }{\Omega } - \eta \frac{q\partial _qr}{r} \right\| _\infty \frac{\delta }{q_0}. \end{aligned} \end{aligned}$$

Therefore by (9.717), (9.718), (9.719), (9.720), we obtain

9.721 $$\begin{aligned} \begin{aligned} \Vert \log (q^{N_\pm } f_{n+1}^\pm ) \Vert _\infty&\le \Vert \log (q^{N_\pm } f^\pm ) |_{\underline{\mathcal {C}}\cup \mathcal {C}} \Vert _\infty + N_\pm k_\pm \Vert \mathcal {U}\Vert _\infty \frac{\delta }{q_0} \\&\quad + 4k_\pm \Vert \mathcal {U}\Vert _\infty \left\| \frac{q\partial _q\Omega }{\Omega } - \eta \frac{q\partial _qr}{r} \right\| _\infty \frac{\delta }{q_0} . \end{aligned} \end{aligned}$$

Next we show that $\partial _qf_{n+1}^\pm$ is continuous. Since $p_*\in C^1$ if $p_*\ne p_0$ by Lemma 9.5, the right-hand side of (9.716) is $C^1$ and thus if $p_*\ne p_0$, $\partial _qf_{n+1}^\pm$ is clearly continuous. Therefore, it suffices to show that $\partial _qf_{n+1}^\pm (p,q)$ is continuous when $p_*=p_0$ and $q_*=q_0$. To this end, let $p_*(p,q)=p_0$ and $q_*(p,q)=q_0$, and take any $(\bar{p},\bar{q})\ne (p,q)$ in a small neighborhood of such $(p,q)$. Then we have $p_*(\bar{p},\bar{q})\ne p_0$ and hence $\partial _qf_{n+1}^\pm (\bar{p},\bar{q})$ is continuous. In fact, for any $(\bar{p},\bar{q})$ with $\bar{p}_*{:}{=} p_*(\bar{p},\bar{q})\ne p_0$ we can solve the equations for $\partial _q\log f_{n+1}^\pm$:

$$\begin{aligned} \begin{aligned} \partial _p(\partial _q\log f_{n+1}^\pm ) + k_\pm \mathcal {U} \partial _q(\partial _q\log f_{n+1}^\pm ) + k_\pm \partial _q\mathcal {U} \partial _q\log f_{n+1}^\pm \pm 4k_\pm \partial _q\left( (\frac{\partial _q\Omega }{\Omega } - \eta \frac{\partial _qr}{r}) \mathcal {U}\right) =0 \end{aligned} \end{aligned}$$

along the characteristics and obtain the integral representation

9.722 $$\begin{aligned} \begin{aligned} \partial _q\log f_{n+1}^\pm (\bar{p},\bar{q})&= \partial _q\log \hat{f}^{\pm }_*(\bar{p},\bar{q}) \\&- \int _{\bar{p}_*}^{\bar{p}} \left[ k_\pm \partial _q\mathcal {U} \partial _q\log f_{n+1}^\pm \pm 4k_\pm \partial _q\left( (\frac{\partial _q\Omega }{\Omega } - \eta \frac{\partial _qr}{r}) \mathcal {U}\right) \right] (s, \mathfrak {q}_\pm (s)) ds \end{aligned} \end{aligned}$$

where

$$\begin{aligned} \partial _q\log \hat{f}^{\pm }_*(\bar{p},\bar{q}) = \textbf{1}_{\bar{p}_*>p_0} \partial _q\log \hat{f}\big \vert _{\underline{\mathcal {C}}}^{\pm }(\bar{p}_*,\bar{q}_*) + \delta _{\bar{p}_*p_0} \partial _q\log \hat{f}^{\pm }(\bar{p}_*,\bar{q}_*), \end{aligned}$$

where $\delta _{\bar{p}_*p_0}$ is the usual Kronecker delta. Notice that the integral terms are all continuous due to the continuity of $p_*$ in Lemma 9.5 and therefore $f_{n+1}^\pm \in C^1(\overline{\mathcal {D}})$.

To estimate $q\partial _q\log f_{n+1}^\pm$, as done in (9.718) and (9.719), we first observe that $\frac{q}{q_*}\le \frac{1 }{1-k_\pm \Vert \mathcal {U}\Vert _\infty q_0^{-1}\delta }$. Then for sufficiently small $\delta >0$ we have

9.723 $$\begin{aligned} \frac{q}{q_*}, \ \frac{q^2}{q_*^2} \le 1+2k_\pm \Vert \mathcal {U}\Vert _\infty \frac{\delta }{q_0}. \end{aligned}$$

Based on the integral representation (9.722) we proceed to estimate

9.724 $$\begin{aligned} |q\partial _q\log \hat{f}^{\pm }_*(\bar{p},\bar{q}) |\le \left( 1+2k_\pm \Vert \mathcal {U}\Vert _\infty \frac{\delta }{q_0} \right) \Vert q\partial _q\log \hat{f}^\pm |_{\underline{\mathcal {C}}\cup \mathcal {C}} \Vert _\infty , \end{aligned}$$

where we have used the first bound in (9.723).

For the second term in (9.722),

$$\begin{aligned} \begin{aligned}&\left| q\int _{p_*}^{p} \left[ k_\pm \partial _q\mathcal {U} \partial _q\log f_{n+1}^\pm \pm 4k_\pm \partial _q\left( (\frac{\partial _q\Omega }{\Omega } - \eta \frac{\partial _qr}{r}) \mathcal {U}\right) \right] (s, \mathfrak {q}_\pm (s)) ds \right| \\&\le k_\pm \left( \Vert q\partial _q\mathcal {U}\Vert _\infty \Vert q\partial _q\log f_{n+1}^\pm \Vert _\infty +4 \left\| q^2 \partial _q\left( (\frac{\partial _q\Omega }{\Omega } - \eta \frac{\partial _qr}{r}) \mathcal {U}\right) \right\| _\infty \right) \frac{q(p-p_*)}{q_*^2} \\&\le k_\pm \left( \Vert q\partial _q\mathcal {U}\Vert _\infty \Vert q\partial _q\log f_{n+1}^\pm \Vert _\infty +4 \left\| q^2 \partial _q\left( (\frac{\partial _q\Omega }{\Omega } - \eta \frac{\partial _qr}{r}) \mathcal {U}\right) \right\| _\infty \right) 2\frac{\delta }{q_0} \end{aligned} \end{aligned}$$

where we have used the upper bound $\frac{q}{q_*} \le 2$ as it follows from (9.723) for $\delta >0$ sufficiently small. Therefore, we deduce that

9.725 $$\begin{aligned} \begin{aligned}&\left( 1-2 k_\pm \Vert q\partial _q\mathcal {U}\Vert _\infty \frac{\delta }{q_0} \right) \Vert q\partial _q\log f_{n+1}^\pm \Vert _\infty \\&\le \left( 1+2k_\pm \Vert \mathcal {U}\Vert _\infty \frac{\delta }{q_0} \right) \Vert q\partial _q\log \hat{f}^\pm |_{\underline{\mathcal {C}}\cup \mathcal {C}} \Vert _\infty + 8k_\pm \frac{\delta }{q_0} \left\| q^2 \partial _q\left( (\frac{\partial _q\Omega }{\Omega } - \eta \frac{\partial _qr}{r}) \mathcal {U}\right) \right\| _\infty . \end{aligned} \end{aligned}$$

From (9.716), since $f_{n+1}^\pm \in W^{2,\infty }$, we just need to estimate $q^2\partial _q^2\log f^\pm _{n+1}$. After applying $\partial _{qq}$ to the equation (9.709), we have the following integral representation:

9.726 $$\begin{aligned} \begin{aligned}&\partial _q^2\log f_{n+1}^\pm (p,q)= \partial _q^2\log \hat{f}_{\pm ,* }(p,q) \\&- \int _{p_*}^{p} \left[ k_\pm \partial _q^2 \mathcal {U} \partial _q\log f_{n+1}^\pm + 2k_\pm \partial _q\mathcal {U} \partial _q^2 \log f_{n+1}^\pm \pm 4k_\pm \partial _q^2 \left( (\frac{\partial _q\Omega }{\Omega } - \eta \frac{\partial _qr}{r}) \mathcal {U}\right) \right] (s, \mathfrak {q}_\pm (s)) ds. \end{aligned} \end{aligned}$$

Analogously to the previous step, we obtain

9.727 $$\begin{aligned} \begin{aligned}&\left( 1-2 k_\pm \Vert q\partial _q\mathcal {U}\Vert _\infty \frac{\delta }{q_0} \right) \Vert q^2 \partial _q^2 \log f_{n+1}^\pm \Vert _\infty \le \left( 1+2k_\pm \Vert \mathcal {U}\Vert _\infty \frac{\delta }{q_0} \right) \Vert q^2 \partial _q^2 \log \hat{f}^\pm |_{\underline{\mathcal {C}}\cup \mathcal {C}} \Vert _\infty \\&+2k_\pm \frac{\delta }{q_0} \Vert q^2\partial _q^2\mathcal {U}\Vert _\infty \Vert q\partial _q\log f_{n+1}^\pm \Vert _\infty +8k_\pm \frac{\delta }{q_0}\left\| q^3 \partial _q^2 \left( (\frac{\partial _q\Omega }{\Omega } - \eta \frac{\partial _qr}{r}) \mathcal {U}\right) \right\| _\infty . \end{aligned} \end{aligned}$$

We now collect (9.721), (9.725), (9.727) and use (9.677), (9.682) as well as ${\left| \left| \left| [f^\pm _{n},\Omega _{n},r_{n}] \right| \right| \right| }\le 2A{\left| \left| \left| [\hat{f}^\pm , \hat{\Omega }, \hat{r}]|_{\underline{\mathcal {C}}\cup \mathcal {C}} \right| \right| \right| }$ to deduce (9.713) for sufficiently small $\delta >0$. This completes the proof. $\square$

##### Proof

(Proof of Lemma 9.13 (Solving the metric part).) As in the previous proof, for the sake of notational convenience, throughout the proof, we use $\Omega =\Omega _n$, $r=r_n$, $f^\pm =f_n^\pm$, $m=m_n$ so that the variables without the indices refer to the ones from the *n*-th step.

Since (9.710), (9.711) are linear inhomogeneous ODEs, we directly integrate them to solve for $$ r_{n+1}$$ and $$\Omega _{n+1}$$. As the existence is clear, we focus on the estimates.

We now integrate (9.710) along an ingoing null curve from $p_0$ to $p$ to obtain

9.728 $$\begin{aligned} \partial _q( (r_{n+1})^2) (p,q) = \partial _q(\hat{r}^2) (p_0, q)+ \int _{p_0}^p\left[ - \frac{\Omega ^2}{2} + \frac{2\pi \Omega ^2 }{r^{2\eta } } (f^+ f^-)^{\frac{1}{k_+-k_-}} \right] d\tilde{p} \end{aligned}$$

with

9.729 $$\begin{aligned} ( r_{n+1})^2 (p,q) = \hat{r}^2(p, q_0)+ \int _{q_0}^q\partial _q(( r_{n+1})^2) (p,\tilde{q})d \tilde{q} . \end{aligned}$$

We first estimate $\frac{1}{q}\partial _q(( r_{n+1})^2)$. To this end, we bound the integral term of (9.728) as

9.730 $$\begin{aligned} \begin{aligned}&\left| \frac{1}{q} \int _{p_0}^p\left[ - \frac{\Omega ^2}{2} + \frac{2\pi \Omega ^2 }{r^{2\eta } } (f^+ f^-)^{\frac{1}{k_+-k_-}} \right] d\tilde{p}\right| \\&\qquad \le \frac{\delta }{q_0} \left( \frac{ \Vert \Omega \Vert ^2_\infty }{2} + \frac{2\pi }{q_0^\beta } \left\| q^{1+\beta } \frac{ \Omega ^2 }{r^{2\eta } } (f^+ f^-)^{\frac{1}{k_+-k_-}} \right\| _\infty \right) \end{aligned} \end{aligned}$$

where we have used $\frac{p-p_0}{q}\le \frac{\delta }{q_0}$. Therefore, we have

9.731 $$\begin{aligned} \begin{aligned} \left\| \frac{1}{q}\partial _q((r_{n+1})^2) \right\| _\infty&\le \left\| \frac{1}{q}\partial _q(\hat{r}^2)|_{\mathcal {C}} \right\| _\infty +\frac{\delta }{q_0} \left( \frac{ \Vert \Omega \Vert ^2_\infty }{2} + \frac{2\pi }{q_0^\beta } \left\| q^{1+\beta } \frac{ \Omega ^2 }{r^{2\eta } } (f^+ f^-)^{\frac{1}{k_+-k_-}} \right\| _\infty \right) . \end{aligned} \end{aligned}$$

We also have

9.732 $$\begin{aligned} \begin{aligned} \frac{1}{q}\partial _q((r_{n+1})^2) (p,q)&\ge \frac{1}{q} \partial _q(\hat{r}^2) (p_0, q) \\&-\frac{\delta }{q_0}\left( \frac{ \Vert \Omega \Vert ^2_\infty }{2} + \frac{2\pi }{q_0^\beta } \left\| q^{1+\beta } \frac{ \Omega ^2 }{r^{2\eta } } (f^+ f^-)^{\frac{1}{k_+-k_-}} \right\| _\infty \right) . \end{aligned} \end{aligned}$$

Since $\frac{1}{q} \partial _q(\hat{r}^2) (p_0, q) \ge \frac{1}{ \Vert \frac{q}{\partial _q(\hat{r}^2)}|_{\mathcal {C}}\Vert _\infty }$, for sufficiently small $\delta$ we obtain

9.733 $$\begin{aligned} \begin{aligned}&\frac{q}{\partial _q(( r_{n+1})^2) (p,q)} \le \frac{1}{ \frac{1}{ \Vert \frac{q}{\partial _q(\hat{r}^2)}|_{\mathcal {C}}\Vert _\infty } -\frac{\delta }{q_0}\left( \frac{ \Vert \Omega \Vert ^2_\infty }{2} + \frac{2\pi }{q_0^\beta } \left\| q^{1+\beta } \frac{ \Omega ^2 }{r^{2\eta } } (f^+ f^-)^{\frac{1}{k_+-k_-}} \right\| _\infty \right) }\\&\le \left\| \frac{q}{\partial _q(\hat{r}^2)}|_{\mathcal {C}}\right\| _\infty +2\frac{\delta }{q_0} \left\| \frac{q}{\partial _q(\hat{r}^2)}|_{\mathcal {C}}\right\| _\infty ^2 \left( \frac{ \Vert \Omega \Vert ^2_\infty }{2} + \frac{2\pi }{q_0^\beta } \left\| q^{1+\beta } \frac{ \Omega ^2 }{r^{2\eta } } (f^+ f^-)^{\frac{1}{k_+-k_-}} \right\| _\infty \right) . \end{aligned} \end{aligned}$$

On the other hand, from (9.729) and (9.731), the upper bound of $\frac{( r_{n+1})^2}{q^2}$ is easily obtained: since $q_0<q$ and using (9.731),

9.734 $$\begin{aligned} \begin{aligned} \frac{( r_{n+1})^2(p,q)}{q^2}&=\frac{\hat{r}^2(p, q_0)}{q^2} + \frac{1}{q^2} \int _{q_0}^q\tilde{q} \frac{\partial _q((r_{n+1})^2) (p,\tilde{q})}{\tilde{q}}d \tilde{q} \\&\le \left\| \frac{\hat{r}^2}{q^2}|_{\underline{\mathcal {C}}\cup \mathcal {C}} \right\| _\infty + \frac{1}{2}\left\| \frac{1}{q}\partial _q(\hat{r}^2)|_{\mathcal {C}} \right\| _\infty \\&\quad +\frac{1}{2}\frac{\delta }{q_0} \left( \frac{ \Vert \Omega \Vert ^2_\infty }{2} + \frac{2\pi }{q_0^\beta } \left\| q^{1+\beta } \frac{ \Omega ^2 }{r^{2\eta } } (f^+ f^-)^{\frac{1}{k_+-k_-}} \right\| _\infty \right) . \end{aligned} \end{aligned}$$

Next we use (9.732) to first obtain from (9.729)

9.735 $$\begin{aligned} \begin{aligned} \frac{( r_{n+1})^2(p,q)}{q^2}&\ge \frac{\hat{r}^2(p, q_0)}{q^2} + \frac{q^2-(q_0)^2}{2q^2} \inf _{\underline{\mathcal {C}}\cup \mathcal {C}} \left[ \frac{1}{q} \partial _q(\hat{r}^2)\right] \\&- \frac{1}{2}\frac{\delta }{q_0} \left( \frac{ \Vert \Omega \Vert ^2_\infty }{2} + \frac{2\pi }{q_0^\beta } \left\| q^{1+\beta } \frac{ \Omega ^2 }{r^{2\eta } } (f^+ f^-)^{\frac{1}{k_+-k_-}} \right\| _\infty \right) \\&\ge \frac{1}{3}\min \left\{ \frac{1}{\Vert \frac{q^2}{\hat{r}^2}|_{\underline{\mathcal {C}}\cup \mathcal {C}}\Vert _\infty }, \frac{1}{\Vert \frac{q}{\partial _q(\hat{r}^2)}|_{\underline{\mathcal {C}}\cup \mathcal {C}}\Vert _\infty } \right\} \\&- \frac{1}{2}\frac{\delta }{q_0} \left( \frac{ \Vert \Omega \Vert ^2_\infty }{2} + \frac{2\pi }{q_0^\beta } \left\| q^{1+\beta } \frac{ \Omega ^2 }{r^{2\eta } } (f^+ f^-)^{\frac{1}{k_+-k_-}} \right\| _\infty \right) , \end{aligned} \end{aligned}$$

where we have used the following: if $\frac{q}{\sqrt{3}}\le q_0$, then $\frac{\hat{r}^2(p, q_0)}{q^2} \ge \frac{\hat{r}^2(p, q_0)}{3(q_0)^2}\ge \frac{1}{3} \frac{1}{\Vert \frac{q^2}{\hat{r}^2}|_{\underline{\mathcal {C}}\cup \mathcal {C}}\Vert _\infty }$, while if $q_0 < \frac{q}{\sqrt{3}}$, $\frac{q^2-(q_0)^2}{2q^2} \inf _{\underline{\mathcal {C}}\cup \mathcal {C}} \left[ \frac{1}{q} \partial _q(\hat{r}^2)\right] \ge \frac{1}{3} \frac{1}{\Vert \frac{q}{\partial _q(\hat{r}^2)}|_{\underline{\mathcal {C}}\cup \mathcal {C}}\Vert _\infty }$. As done in (9.733), we deduce

9.736 $$\begin{aligned}&\frac{q^2}{( r_{n+1})^2(p,q)}\le 3\max \left\{ \left\| \frac{q^2}{\hat{r}^2}|_{\underline{\mathcal {C}}\cup \mathcal {C}}\right\| _\infty ,\left\| \frac{q}{\partial _q(\hat{r}^2)}|_{\underline{\mathcal {C}}\cup \mathcal {C}}\right\| _\infty \right\} \\&+ 9 \frac{\delta }{q_0} \max \left\{ \left\| \frac{q^2}{\hat{r}^2}|_{\underline{\mathcal {C}}\cup \mathcal {C}}\right\| _\infty ,\left\| \frac{q}{\partial _q(\hat{r}^2)}|_{\underline{\mathcal {C}}\cup \mathcal {C}}\right\| _\infty \right\} ^2 \left( \frac{ \Vert \Omega \Vert ^2_\infty }{2} + \frac{2\pi }{q_0^\beta } \left\| q^{1+\beta } \frac{ \Omega ^2 }{r^{2\eta } } (f^+ f^-)^{\frac{1}{k_+-k_-}} \right\| _\infty \right) . \nonumber \end{aligned}$$

We now differentiate (9.710) with respect to $\partial _q$ and integrate:

9.737 $$\begin{aligned} \begin{aligned}x \partial _q^2 (( r_{n+1})^2) (p,q) = \partial _q^2 (\hat{r}^2) (p_0, q)+ \int _{p_0}^p\partial _q\left[ - \frac{\Omega ^2}{2} + \frac{2\pi \Omega ^2 }{r^{2\eta } } (f^+ f^-)^{\frac{1}{k_+-k_-}} \right] d\tilde{p} . \end{aligned} \end{aligned}$$

Now the estimation of $\partial _q^2((r_{n+1})^2)$ follows similarly. Using $\frac{p-p_0}{q}\le \frac{\delta }{q_0}$,

9.738 $$\begin{aligned} \begin{aligned}&\Vert \partial _q^2 (( r_{n+1})^2)\Vert _\infty \le \Vert \partial _q^2 (\hat{r}^2)|_{\mathcal {C}} \Vert _\infty \\&\quad + \frac{\delta }{q_0} \left( \frac{\Vert \Omega \Vert ^2_\infty }{q_0^\theta } \Vert q^{1+\theta }\partial _q\log \Omega \Vert _\infty + \frac{2\pi }{q_0^{1+\beta }} \left\| q^{2+\beta } \partial _q\left( \frac{ \Omega ^2 }{r^{2\eta } } (f^+ f^-)^{\frac{1}{k_+-k_-}} \right) \right\| _\infty \right) . \end{aligned} \end{aligned}$$

The third derivative $q\partial _q^3((r_{n+1})^2)$ can be estimated analogously:

9.739 $$\begin{aligned} \begin{aligned} \Vert q\partial _q^3 ((r_{n+1})^2)\Vert _\infty \le&\Vert q\partial _q^3 (\hat{r}^2)|_{\mathcal {C}} \Vert _\infty + \frac{\delta }{q_0} \Vert \Omega \Vert ^2_\infty \left( \frac{ \Vert q^{2+\theta }\partial _q^2 \log \Omega \Vert _\infty }{q_0^\theta } + \frac{2 \Vert q^{1+\theta }\partial _q\log \Omega \Vert ^2_\infty }{q_0^{2\theta }} \right) \\&+ \frac{\delta }{q_0} \frac{2\pi }{q_0^{1+\beta }} \left\| q^{3+\beta } \partial _q^2\left( \frac{ \Omega ^2 }{r^{2\eta } } (f^+ f^-)^{\frac{1}{k_+-k_-}} \right) \right\| _\infty . \end{aligned} \end{aligned}$$

We proceed in the same way for $\Omega _{n+1}$. By integrating (9.711) along an ingoing curve we obtain the expression for $\partial _q\log \Omega _{n+1} (p,q)$

9.740 $$\begin{aligned} \begin{aligned} \partial _q\log \Omega _{n+1} (p,q)&= \partial _q(\log \hat{\Omega }) (p_0, q) + \int _{p_0}^p\left[ \frac{ \Omega ^2}{2 r^2} \frac{m}{r} - (1+\eta ) \pi \frac{ \Omega ^2 }{r^{2+2\eta }}(f^+ f^-)^{\frac{1}{k_+-k_-}} \right] d\tilde{p} \end{aligned} \end{aligned}$$

with

9.741 $$\begin{aligned} \Omega _{n+1} (p,q) =\hat{\Omega }(p, q_0) e^{\int _{q_0}^q\partial _q\log \Omega _{n+1} (p,\tilde{q}) d\tilde{q} }. \end{aligned}$$

To estimate $q^{1+\theta }\partial _q\log \Omega _{n+1}$, we start with the integral term of (9.740). By taking the sup norm, and using $\frac{p-p_0}{q}\le \frac{\delta }{q_0}$, we obtain

9.742 $$\begin{aligned} \begin{aligned}&\left| q^{1+\theta } \int _{p_0}^p\left[ \frac{ \Omega ^2}{2 r^2} \frac{m}{r} - (1+\eta ) \pi \frac{ \Omega ^2 }{r^{2+2\eta }}(f^+ f^-)^{\frac{1}{k_+-k_-}} \right] d\tilde{p} \right| \\&\le \frac{\delta }{q_0} \left( \frac{1}{2}\frac{1}{q^{1-\theta }} \Vert \Omega \Vert ^2_\infty \left\| \frac{q}{r}\right\| ^3_\infty \Vert m\Vert _\infty + \frac{(1+\eta )\pi }{q^{1-\theta +\beta }} \left\| q^{3+\beta } \frac{ \Omega ^2 }{r^{2+2\eta } } (f^+ f^-)^{\frac{1}{k_+-k_-}} \right\| _\infty \right) \end{aligned} \end{aligned}$$

and hence

9.743 $$\begin{aligned} \begin{aligned}&\Vert q^{1+\theta } \partial _q\log \Omega _{n+1}\Vert _\infty \le \Vert q^{1+\theta } \partial _q\log \hat{\Omega }|_{\mathcal {C}}\Vert _\infty \\&+\frac{\delta }{q_0} \left( \frac{1}{2}\frac{1}{q_0^{1-\theta }} \Vert \Omega \Vert ^2_\infty \left\| \frac{q}{r}\right\| ^3_\infty \Vert m\Vert _\infty + \frac{(1+\eta )\pi }{q_0^{1-\theta +\beta }} \left\| q^{3+\beta } \frac{ \Omega ^2 }{r^{2+2\eta } } (f^+ f^-)^{\frac{1}{k_+-k_-}} \right\| _\infty \right) . \end{aligned} \end{aligned}$$

For $\Omega _{n+1}$, we first estimate the integral of (9.741).

$$\begin{aligned} \begin{aligned} \left| \int _{q_0}^q\partial _q\log \Omega _{n+1} (p,\tilde{q}) d\tilde{q}\right|&=\left| \int _{q_0}^q\frac{1}{\tilde{q}^{1+\theta }} \tilde{q}^{1+\theta }\partial _q\log \Omega _{n+1} (p,\tilde{q}) d\tilde{q}\right| \\&\le \Vert q^{1+\theta } \partial _q\log \Omega _{n+1}\Vert _\infty \frac{1}{\theta } \frac{1}{q_0^\theta }. \end{aligned} \end{aligned}$$

Therefore we deduce

9.744 $$\begin{aligned} \begin{aligned}&\Vert \log \Omega _{n+1}\Vert _\infty \le \Vert \log \hat{\Omega }|_{\underline{\mathcal {C}}\cup \mathcal {C}}\Vert _\infty +\frac{1}{\theta } \frac{1}{(q_0)^\theta } \Vert q^{1+\theta } \partial _q\log \Omega _{n+1}|_{\mathcal {C}}\Vert _\infty \\&+ \frac{1}{\theta } \frac{1}{q_0^\theta }\frac{\delta }{q_0} \left( \frac{1}{2}\frac{1}{q_0^{1-\theta }} \Vert \Omega \Vert ^2_\infty \left\| \frac{q}{r}\right\| ^3_\infty \Vert m\Vert _\infty + \frac{(1+\eta )\pi }{q_0^{1-\theta +\beta }} \left\| q^{3+\beta } \frac{ \Omega ^2 }{r^{2+2\eta } } (f^+ f^-)^{\frac{1}{k_+-k_-}} \right\| _\infty \right) . \end{aligned} \end{aligned}$$

To estimate $\partial _q^2\log \Omega _{n+1}$, we first observe that

9.745 $$\begin{aligned} \begin{aligned} \partial _q^2 \log \Omega _{n+1} (p,q)&= \partial _q^2 (\log \hat{\Omega }) (p_0, q) + \int _{p_0}^p\partial _q\left[ \frac{ \Omega ^2}{2 r^2} \frac{m}{r} - (1+\eta ) \pi \frac{ \Omega ^2 }{r^{2+2\eta }}(f^+ f^-)^{\frac{1}{k_+-k_-}} \right] d\tilde{p}, \end{aligned} \end{aligned}$$

wherefrom we deduce

9.746 $$\begin{aligned} \begin{aligned}&\Vert q^{2+\theta }\partial _q^2 \log \Omega _{n+1}\Vert _\infty \le \Vert q^{2+\theta }\partial _q^2 \log \hat{\Omega }|_{\mathcal {C}}\Vert _\infty \\&+ \frac{\delta }{q_0} \frac{1}{2} \left( \Vert \Omega \Vert _\infty ^2\left\| \frac{q}{r}\right\| ^3_\infty \frac{ \Vert q^{1+\beta }\partial _qm \Vert _\infty }{q_0^{1-\theta +\beta }} + \left\| q^{4}\partial _q\left( \frac{\Omega ^2}{r^3}\right) \right\| _\infty \frac{\Vert m\Vert _\infty }{q_0^{1-\theta }}\right) \\&+ \frac{\delta }{q_0} \frac{(1+\eta )\pi }{q_0} \left\| q^{4+\beta } \partial _q\left( \frac{ \Omega ^2 }{r^{2+2\eta } } (f^+ f^-)^{\frac{1}{k_+-k_-}}\right) \right\| _\infty . \end{aligned} \end{aligned}$$

Similarly one can derive

9.747 $$\begin{aligned} \begin{aligned}&\Vert q^{3+\theta }\partial _q^3 \log \Omega _{n+1}\Vert _\infty \le \Vert q^{3+\theta }\partial _q^3 \log \hat{\Omega }|_{\mathcal {C}}\Vert _\infty \\&+ \frac{\delta }{q_0} \frac{1}{2} \left( \Vert \Omega \Vert _\infty ^2\left\| \frac{q}{r}\right\| ^3_\infty \frac{ \Vert q^{2+\beta }\partial _q^2 m \Vert _\infty }{q_0^{1-\theta +\beta }} + 2 \left\| q^{4}\partial _q\left( \frac{\Omega ^2}{r^3}\right) \right\| _\infty \frac{ \Vert q^{1+\beta }\partial _qm \Vert _\infty }{q_0^{1-\theta +\beta }} + \left\| q^{5}\partial _q^2\left( \frac{\Omega ^2}{r^3}\right) \right\| _\infty \frac{\Vert m\Vert _\infty }{q_0^{1-\theta }}\right) \\&+ \frac{\delta }{q_0} \frac{(1+\eta )\pi }{q_0} \left\| q^{5+\beta } \partial _q^2\left( \frac{ \Omega ^2 }{r^{2+2\eta } } (f^+ f^-)^{\frac{1}{k_+-k_-}}\right) \right\| _\infty . \end{aligned} \end{aligned}$$

We now collect (9.731), (9.733), (9.734), (9.736), (9.738), (9.739), (9.743), (9.744), (9.746), (9.747) and use (9.689), (9.690), (9.678), (9.701), (9.702) as well as ${\left| \left| \left| [f^\pm _{n},\Omega _{n},r_{n}] \right| \right| \right| }\le 2A{\left| \left| \left| [\hat{f}^\pm , \hat{\Omega }, \hat{r}]|_{\underline{\mathcal {C}}\cup \mathcal {C}} \right| \right| \right| }$ to deduce (9.713) for sufficiently small $\delta >0$. This concludes the proof. $\square$

#### Convergence of the Iteration Scheme

From Proposition 9.11, the solutions $[f^\pm _{n+1},\Omega _{n+1},r_{n+1}]$ of (9.709), (9.710), (9.711) have the uniform bounds ${\left| \left| \left| [f^\pm _{n+1},\Omega _{n+1},r_{n+1}] \right| \right| \right| }\le 2A{\left| \left| \left| [\hat{f}^\pm , \hat{\Omega }, \hat{r}]|_{\underline{\mathcal {C}}\cup \mathcal {C}} \right| \right| \right| }$ for all $n\ge 0$. In this section, we show the convergence of the sequence of approximations $[f^\pm _{n+1},\Omega _{n+1},r_{n+1}]$. We will estimate the difference between $[f^\pm _{n+1},\Omega _{n+1},r_{n+1}]$ and $[f^\pm _{n},\Omega _{n},r_{n}]$. Let

9.748 $$\begin{aligned}{}[\triangle f_{n+1}^\pm , \triangle \Omega _{n+1},\triangle r_{n+1} ]{:}{=}[\log f^\pm _{n+1}-\log f^\pm _n, \log \Omega _{n+1}-\log \Omega _n, (r_{n+1})^2-(r_n)^2] \end{aligned}$$

for $n\ge 0$. Our next task is to prove the corresponding difference bounds.

##### Proposition 9.14

Let $[f^\pm _{n+1},\Omega _{n+1},r_{n+1}]$ be the solution (9.709), (9.710), (9.711) enjoying the uniform bounds ${\left| \left| \left| [f^\pm _{n+1},\Omega _{n+1},r_{n+1}] \right| \right| \right| }\le 2A{\left| \left| \left| [\hat{f}^\pm , \hat{\Omega }, \hat{r}]|_{\underline{\mathcal {C}}\cup \mathcal {C}} \right| \right| \right| }$ for all $n\ge 0$. Then the difference norm for $[\triangle f_{n+1}^\pm , \triangle \Omega _{n+1},\triangle r_{n+1} ]$ defined in (9.748) satisfies the following recursive inequality

9.749 $$\begin{aligned} a_{n+1} \le C \frac{\delta }{q_0} a_n \ \ \text { for } \ \ n\ge 1 \end{aligned}$$

where

9.750 $$\begin{aligned} a_{n+1}=\Vert \triangle f_{n+1}^\pm \Vert _\infty + \Vert q^{-2}{\triangle r_{n+1}}\Vert _\infty + \Vert q^{-1}{\partial _q\triangle r_{n+1}}\Vert _\infty +\Vert \triangle \Omega _{n+1}\Vert _\infty + \Vert q^{1+\theta }\partial _q\triangle \Omega _{n+1}\Vert _\infty \end{aligned}$$

and *C* does not depend on *n* but only on $2A{\left| \left| \left| [\hat{f}^\pm , \hat{\Omega }, \hat{r}]|_{\underline{\mathcal {C}}\cup \mathcal {C}} \right| \right| \right| }$.

The proof relies on $C^0$ estimates of $[\triangle f_{n+1}^\pm ]$ and $C^1$ estimates of $[\triangle \Omega _{n+1},\triangle r_{n+1} ]$ in the spirit of Lemma 9.12 and Lemma 9.13. Before giving the proof, we present some preliminary estimates. First, we note that $[\triangle f_{n+1}^\pm , \triangle \Omega _{n+1},\triangle r_{n+1} ]$ satisfy

9.751 $$\begin{aligned}&\partial _p\triangle f_{n+1}^\pm + k_\pm \mathcal {U}_n \partial _q\triangle f_{n+1}^\pm + k_\pm (\mathcal {U}_n-\mathcal {U}_{n-1})\partial _q\log f^\pm _n \end{aligned}$$

9.752 $$\begin{aligned}&\qquad \quad \ \pm 2k_\pm \left[ (2\partial _q\log \Omega _n - \eta \frac{\partial _q(r_n)^2}{(r_n)^2}) \mathcal {U}_n -(2\partial _q\log \Omega _{n-1} - \eta \frac{\partial _q(r_{n-1})^2}{(r_{n-1})^2}) \mathcal {U}_{n-1} \right] =0 , \nonumber \\&\partial _p\partial _q\triangle r_{n+1}= \frac{(\Omega _{n-1})^2-(\Omega _n)^2 }{2} + 2\pi \left[ \frac{(\Omega _n)^2}{(r_n)^{2\eta }} (f^+_nf^-_n) ^{\frac{1}{k_+-k_-}} -\frac{(\Omega _{n-1})^2}{(r_{n-1})^{2\eta }} (f^+_{n-1}f^-_{n-1}) ^{\frac{1}{k_+-k_-}} \right] , \end{aligned}$$

9.753 $$\begin{aligned}&\partial _p\partial _q\triangle \Omega _{n+1} =\frac{1}{2}\left[ \frac{ (\Omega _n)^2}{ (r_n)^2} \frac{m_n}{r_n} - \frac{ (\Omega _{n-1})^2}{ (r_{n-1})^2} \frac{m_{n-1}}{r_{n-1}} \right] \nonumber \\&\qquad \qquad - (1+\eta ) \pi \left[ \frac{ (\Omega _n)^2 }{(r_n)^{2+2\eta }}(f^+_n f^-_n)^{\frac{1}{k_+-k_-}} - \frac{ (\Omega _{n-1})^2 }{(r_{n-1})^{2+2\eta }}(f^+_{n-1} f^-_{n-1})^{\frac{1}{k_+-k_-}} \right] , \end{aligned}$$

with $$[\triangle f_{n+1}^\pm , \triangle \Omega _{n+1},\triangle r_{n+1} ] |_{\underline{\mathcal {C}}\cup \mathcal {C}} =0$$.

We start with the following inequality, which will allow us to compare the difference of two functions to the logarithm of their ratio.

##### Lemma 9.15

Let $L>0$ be given. For any $-1< x \le L$, the following holds

9.754 $$\begin{aligned} |x|\le (1+L) |\log (1+ x )| . \end{aligned}$$

##### Proof

If $x\in (-1,0)$, we claim $-x \le -(1+L)\log (1+x)$. Letting $h(x) = (1+L)\log (1+x)-x$, we have $h(0)=0$ and $h'(x) = \frac{1+L}{1+x} - 1 >0$ for $x\in (-1,0)$. Hence $h<0$ for $x\in (-1,0)$. Now let $0\le x \le L$. Then since $h'(x)\ge 0$ for $0\le x \le L$, $h\ge 0$ for $0\le x \le L$, which shows (9.754). $\square$

##### Lemma 9.16

For any $b>0$ let

9.755 $$\begin{aligned} \ell _\Omega {:}{=} \max \left\{ \sup _{n} \frac{(\Omega _{n})^2}{(\Omega _{n-1})^2}, \ \sup _{n} \frac{(\Omega _{n-1})^2}{(\Omega _{n})^2}\right\} , \quad \ell ^\pm =\max \left\{ \sup _n \left( \frac{f_n^\pm }{f_{n-1}^\pm }\right) ^b, \ \sup _n \left( \frac{f_{n-1}^\pm }{f_{n}^\pm }\right) ^b \right\} , \end{aligned}$$

and

9.756 $$\begin{aligned} \ell _R {:}{=}\max \left\{ \sup _{n} \left\| \frac{q^b}{(r_{n-1})^b} \frac{(q^{-1}r_n)^b - (q^{-1}r_{n-1})^b}{ (q^{-1}r_n)^2 -(q^{-1} r_{n-1})^2 } \right\| _\infty , \ \sup _{n} \left\| \frac{q^b}{(r_{n})^b} \frac{(q^{-1}r_n)^b - (q^{-1}r_{n-1})^b}{ (q^{-1}r_n)^2 -(q^{-1} r_{n-1})^2 } \right\| _\infty \right\} . \end{aligned}$$

Note that $\ell _\Omega$, $\ell ^\pm$, $\ell _R$ are finite by the uniform bound ${\left| \left| \left| [f^\pm _{n+1},\Omega _{n+1},r_{n+1}] \right| \right| \right| }\le 2A{\left| \left| \left| [\hat{f}^\pm , \hat{\Omega }, \hat{r}]|_{\underline{\mathcal {C}}\cup \mathcal {C}} \right| \right| \right| }$. Then the following bounds hold:

9.757 $$\begin{aligned}&\left\| \left( \frac{f_n^\pm }{f_{n-1}^\pm }\right) ^b - 1\right\| _\infty , \quad \left\| \left( \frac{f_{n-1}^\pm }{f_{n}^\pm } \right) ^b- 1\right\| _\infty \le b \ell ^\pm \Vert \triangle f_{n} \Vert _\infty , \end{aligned}$$

9.758 $$\begin{aligned}&\left\| \frac{(\Omega _{n})^2}{(\Omega _{n-1})^2} - 1\right\| _\infty , \quad \left\| \frac{(\Omega _{n-1})^2}{(\Omega _{n})^2}- 1\right\| _\infty \le 2 \ell _\Omega \Vert \triangle \Omega _{n} \Vert _\infty , \end{aligned}$$

9.759 $$\begin{aligned}&\left\| \frac{(r_n)^b}{(r_{n-1})^b} -1 \right\| _\infty , \quad \left\| \frac{(r_{n-1})^b}{(r_{n})^b} -1 \right\| _\infty \le \ell _R \left\| q^{-2}\triangle r_{n} \right\| _\infty . \end{aligned}$$

##### Proof

(9.757) and (9.758) are direct consequences of (9.754), (9.755) with $x+1=( \frac{f_n^\pm }{f_{n-1}^\pm })^b$, $(\frac{f_{n-1}^\pm }{f_{n}^\pm } )^b$, $\frac{(\Omega _{n})^2}{(\Omega _{n-1})^2}$, $\frac{(\Omega _{n-1})^2}{(\Omega _{n})^2}$. (9.759) directly follows by writing

$$\begin{aligned} \frac{(r_n)^b}{(r_{n-1})^b} -1 = \frac{q^b}{(r_{n-1})^b} \frac{(q^{-1}r_n)^b - (q^{-1}r_{n-1})^b}{ (q^{-1}r_n)^2 -(q^{-1} r_{n-1})^2 } \left( q^{-2} [ (r_n)^2 - (r_{n-1})^2 ]\right) . \end{aligned}$$

$$\square $$

We are now ready to prove Proposition 9.14.

##### Proof of Proposition 9.14

We start with $\Vert \triangle f_{n+1}^\pm \Vert _\infty$. We recall the notations from Lemma 9.12. By integrating (9.751) along the characteristics and using the zero data, we have the representation of $\triangle f_{n+1}^\pm$:

9.760 $$\begin{aligned} \begin{aligned} \triangle f_{n+1}^\pm (p,q)= k_\pm \int _{p_*}^p(S_1 +S_2) (s, \mathfrak {q}_\pm (s)) ds \end{aligned} \end{aligned}$$

where

9.761 $$\begin{aligned} \begin{aligned} S_1&= - (\mathcal {U}_n-\mathcal {U}_{n-1})\partial _q\log f^\pm _n , \\ S_2&= \mp 2 \left[ (2\partial _q\log \Omega _n - \eta \frac{\partial _q(r_n)^2}{(r_n)^2}) \mathcal {U}_n -(2\partial _q\log \Omega _{n-1} - \eta \frac{\partial _q(r_{n-1})^2}{(r_{n-1})^2}) \mathcal {U}_{n-1} \right] . \end{aligned} \end{aligned}$$

Using $\mathfrak {q}_\pm (s)\ge q_*$ for all $p_* \le s\le p$ and $p-p_* \le \delta$,

9.762 $$\begin{aligned} \begin{aligned} \left| \int _{p_*}^pS_1 (s, \mathfrak {q}_\pm (s)) ds \right|&= \left| \int _{p_*}^p\frac{1}{\mathfrak {q}_\pm } (\mathcal {U}_n-\mathcal {U}_{n-1}) \mathfrak {q}_\pm \partial _q\log f^\pm _n (s, \mathfrak {q}_\pm (s)) ds \right| \\&\le \Vert \mathcal {U}_{n-1}\Vert _\infty \Vert q\partial _q\log f_n^\pm \Vert _\infty \left\| \frac{\mathcal {U}_n}{\mathcal {U}_{n-1}} -1 \right\| _\infty \frac{\delta }{q_0}. \end{aligned} \end{aligned}$$

For $\left\| \frac{\mathcal {U}_n}{\mathcal {U}_{n-1}} -1 \right\| _\infty$, we rewrite

9.763 $$\begin{aligned} \begin{aligned} \frac{ \mathcal {U}_n}{ \mathcal {U}_{n-1}}-1&=\left( \frac{\Omega _n}{\Omega _{n-1}}\right) ^2 \left( \frac{f^+_n}{f^+_{n-1}}\right) ^\frac{1}{2} \left( \frac{f^-_{n-1}}{f^-_{n}}\right) ^\frac{1}{2} \left( \frac{(r_{n-1})^2}{(r_n)^2}\right) ^\eta -1 \\&= \left[ \left( \frac{\Omega _n}{\Omega _{n-1}}\right) ^2 -1 \right] \left( \frac{f^+_n}{f^+_{n-1}}\right) ^\frac{1}{2} \left( \frac{f^-_{n-1}}{f^-_{n}}\right) ^\frac{1}{2} \left( \frac{(r_{n-1})^2}{(r_n)^2}\right) ^\eta \\&+ \left[ \left( \frac{f^+_n}{f^+_{n-1}}\right) ^\frac{1}{2}-1 \right] \left( \frac{f^-_{n-1}}{f^-_{n}}\right) ^\frac{1}{2} \left( \frac{(r_{n-1})^2}{(r_n)^2}\right) ^\eta \\&+ \left[ \left( \frac{f^-_{n-1}}{f^-_{n}}\right) ^\frac{1}{2}-1 \right] \left( \frac{(r_{n-1})^2}{(r_n)^2}\right) ^\eta + \left( \frac{(r_{n-1})^2}{(r_n)^2}\right) ^\eta -1 . \end{aligned} \end{aligned}$$

Note that factors next to the rectangular brackets are all uniformly bounded. By Lemma 9.16, we deduce that

9.764 $$\begin{aligned} \left\| \frac{\mathcal {U}_n}{\mathcal {U}_{n-1}} -1 \right\| _\infty \le c_1 (\Vert \triangle f_{n}\Vert _\infty + \Vert \triangle f_{n}\Vert _\infty +\Vert q^{-2}\triangle r_{n}\Vert _\infty ) \end{aligned}$$

where $c_1>0$ is independent of *n* and hence using the uniform bounds of the uniform bounds of ${\left| \left| \left| [f^\pm _{n-1},\Omega _{n-1},r_{n-1}] \right| \right| \right| }$ and ${\left| \left| \left| [f^\pm _{n},\Omega _{n},r_{n}] \right| \right| \right| }$, we have

9.765 $$\begin{aligned} \left| \int _{p_*}^pS_1 (s, \mathfrak {q}_\pm (s)) ds \right| \le c_2 \frac{\delta }{q_0}(\Vert \triangle f_{n}\Vert _\infty + \Vert \triangle \Omega _{n}\Vert _\infty +\Vert q^{-2}\triangle r_{n}\Vert _\infty ) \end{aligned}$$

where $c_2>0$ is independent of *n*. For $S_2$, we first rewrite

9.766 $$\begin{aligned} \begin{aligned} \mp \frac{ S_2}{2}&= \left( 2\partial _q\triangle \Omega _{n} - \eta \left( \frac{\partial _q\triangle r_{n}}{(r_n)^2} + \frac{\partial _q(r_{n-1})^2}{(r_{n-1})^2} \left[ \frac{(r_{n-1})^2}{(r_n)^2}-1 \right] \right) \right) \mathcal {U}_n \\&\quad +(2\partial _q\log \Omega _{n-1} - \eta \frac{\partial _q(r_{n-1})^2}{(r_{n-1})^2}) \mathcal {U}_{n-1} \left( \frac{\mathcal {U}_n}{\mathcal {U}_{n-1}} - 1 \right) . \end{aligned} \end{aligned}$$

Now by using the uniform bounds on ${\left| \left| \left| [f^\pm _{n-1},\Omega _{n-1},r_{n-1}] \right| \right| \right| }$ and ${\left| \left| \left| [f^\pm _{n},\Omega _{n},r_{n}] \right| \right| \right| }$, Lemma 9.16, (9.764), and $\frac{p-p_*}{q_*}\le \frac{\delta }{q_0}$, we deduce

9.767 $$\begin{aligned} \begin{aligned} \left| \int _{p_*}^pS_2 (s, \mathfrak {q}_\pm (s)) ds \right|&\le c_3 \frac{\delta }{q_0}( \Vert q^{1+\theta }\partial _q\triangle \Omega _{n}\Vert _\infty +\Vert q^{-1}\partial _q\triangle r_{n}\Vert _\infty +\Vert \triangle \Omega _{n}\Vert _\infty \\&\quad + \Vert \triangle f_{n}\Vert _\infty +\Vert q^{-2}\triangle r_{n}\Vert _\infty ). \end{aligned} \end{aligned}$$

We now estimate $\Vert q^{-2}{\triangle r_{n+1}}\Vert _\infty + \Vert q^{-1}{\partial _q\triangle r_{n+1}}\Vert _\infty$. We recall the notations used in the proof of Lemma 9.13. We first integrate (9.752) along an ingoing null curve from the initial point $p_0=p_0$ to $p$ to obtain

9.768 $$\begin{aligned} \partial _q\triangle r_{n+1}(p,q) = \int _{p_0}^pS_3 +S_4 \, d\tilde{p} \end{aligned}$$

where

9.769 $$\begin{aligned} \begin{aligned} S_3&=- \frac{( \Omega _{n-1})^2}{2} \left[ \frac{(\Omega _n)^2}{(\Omega _{n-1})^2}-1 \right] , \\ S_4&= 2\pi \frac{(\Omega _{n-1})^2}{(r_{n-1})^{2\eta }} (f^+_{n-1}f^-_{n-1}) ^{\frac{1}{k_+-k_-}} \left( \frac{(\Omega _n)^2}{(\Omega _{n-1})^2} \frac{(r_{n-1})^{2\eta }}{(r_n)^{2\eta }} (\frac{f^+_n}{f^+_{n-1}})^{\frac{1}{k_+-k_-}} (\frac{f^-_n}{f^-_{n-1}})^{\frac{1}{k_+-k_-}} -1\right) . \end{aligned} \end{aligned}$$

The last factor of $S_4$ can be rewritten via add and subtract trick as done for $\frac{ \mathcal {U}_n}{ \mathcal {U}_{n-1}}-1$ in (9.763). Then using $\frac{p-p_0}{q} \le \frac{\delta }{q_0}$, Lemma 9.16, the uniform bounds, we deduce that

9.770 $$\begin{aligned} \Vert q^{-1} \partial _q\triangle r_{n+1}\Vert _\infty \le c_4 \frac{\delta }{q_0}(\Vert \triangle f_{n}\Vert _\infty + \Vert \triangle \Omega _{n}\Vert _\infty +\Vert q^{-2}\triangle r_{n}\Vert _\infty ). \end{aligned}$$

By integrating (9.768) with respect to $q$, we have $\triangle r_{n+1} (p,q)=\int _{q_0}^q\partial _q\triangle r_{n+1}(p,\tilde{q}) d\tilde{q}$. Then by writing $q^{-2}\triangle r_{n+1} (p,q)=q^{-2}\int _{q_0}^q\tilde{q} \tilde{q}^{-1} \partial _q\triangle r_{n+1}(p,\tilde{q}) d\tilde{q}$, we deduce that

9.771 $$\begin{aligned} \Vert q^{-2} \triangle r_{n+1}\Vert _\infty \le \frac{c_4}{2} \frac{\delta }{q_0}(\Vert \triangle f_{n}\Vert _\infty + \Vert \triangle \Omega _{n}\Vert _\infty +\Vert q^{-2}\triangle r_{n}\Vert _\infty ). \end{aligned}$$

The estimates for $\Vert \triangle \Omega _{n+1}\Vert _\infty + \Vert q^{1+\theta }\partial _q\triangle \Omega _{n+1}\Vert _\infty$ can be derived in the same fashion. By integrating (9.753),

9.772 $$\begin{aligned} \partial _q\triangle \Omega _{n+1}(p,q) = \int _{p_0}^pS_5 +S_6\, d\tilde{p}, \end{aligned}$$

where

9.773 $$\begin{aligned} \begin{aligned} S_5&=\frac{1}{2}\left[ \frac{ (\Omega _n)^2}{ (r_n)^2} \frac{m_n}{r_n} - \frac{ (\Omega _{n-1})^2}{ (r_{n-1})^2} \frac{m_{n-1}}{r_{n-1}} \right] , \\ S_6&=- (1+\eta ) \pi \frac{ (\Omega _{n-1})^2 }{(r_{n-1})^{2+2\eta }}(f^+_{n-1} f^-_{n-1})^{\frac{1}{k_+-k_-}} \left[ \frac{(\Omega _n)^2}{(\Omega _{n-1})^2} \frac{(r_{n-1})^{2+2\eta }}{(r_n)^{2+2\eta }} (\frac{f^+_n}{f^+_{n-1}})^{\frac{1}{k_+-k_-}} (\frac{f^-_n}{f^-_{n-1}})^{\frac{1}{k_+-k_-}} - 1\right] . \end{aligned} \end{aligned}$$

The structure of $S_6$ is similar to $S_4$ in the previous case. Using the uniform bounds for ${\left| \left| \left| [f^\pm _{n-1},\Omega _{n-1},r_{n-1}] \right| \right| \right| }$ and ${\left| \left| \left| [f^\pm _{n},\Omega _{n},r_{n}] \right| \right| \right| }$ and Lemma 9.16, we have

9.774 $$\begin{aligned} \left| q^{1+\theta } \int _{p_0}^pS_6 d\tilde{p} \right| \le c_5 \frac{\delta }{q_0}(\Vert \triangle f_{n}\Vert _\infty + \Vert \triangle \Omega _{n}\Vert _\infty +\Vert q^{-2}\triangle r_{n}\Vert _\infty ) \end{aligned}$$

for some $c_5>0$ independent of *n*. For $S_5$, we rewrite it

9.775 $$\begin{aligned} 2S_5 = \frac{ (\Omega _n)^2}{ (r_n)^3} (m_n-m_{n-1}) +\left( \frac{ (\Omega _n)^2}{ (\Omega _{n-1})^2}\frac{(r_{n-1})^3}{ (r_n)^3} - 1\right) \frac{ (\Omega _{n-1})^2}{ (r_{n-1})^3} m_{n-1}. \end{aligned}$$

The second term is in the form where we can apply Lemma 9.16. For the first term, note that

9.776 $$\begin{aligned} \begin{aligned} m_n-m_{n-1} = \int _{q_0}^q\left( 2\pi (f_n^+f_n^-)^\frac{1}{k_+-k_-} \left[ \frac{1 }{(r_n)^{2\eta }} \partial _qr_n - \frac{1}{2}\frac{ (\Omega _n)^{-2} }{r_n} \left( \frac{f^-_n}{f_n^+}\right) ^\frac{1}{2} \mathfrak s_n\right] \right) \\ - \left( 2\pi (f_{n-1}^+f_{n-1}^-)^\frac{1}{k_+-k_-} \left[ \frac{1 }{(r_{n-1})^{2\eta }} \partial _qr_{n-1} - \frac{1}{2}\frac{(\Omega _{n-1})^{-2}}{r_{n-1}} \left( \frac{f^-_{n-1}}{f_{n-1}^+}\right) ^\frac{1}{2} \mathfrak s_{n-1}\right] \right) d\tilde{q} \end{aligned} \end{aligned}$$

where we also recall (9.700) for $\mathfrak s_n$. As done for $\frac{ \mathcal {U}_n}{ \mathcal {U}_{n-1}}-1$ in (9.763), it is evident that the integrand can be written as the sum of the terms that contain one of the forms in Lemma 9.16 or $\partial _q\triangle r_{n}$ or $\mathfrak s_n -\mathfrak s_{n-1}$ where

$$\begin{aligned} \mathfrak s_n -\mathfrak s_{n-1} = \int _{q_0}^q\left[ \frac{(\Omega _{n-1})^2-(\Omega _n)^2}{2} + \frac{2\pi (\Omega _n)^2 }{(r_n)^{2\eta } } (f_n^+ f_n^-)^{\frac{1}{k_+-k_-}} - \frac{2\pi (\Omega _{n-1})^2 }{(r_{n-1})^{2\eta } } (f_{n-1}^+ f_{n-1}^-)^{\frac{1}{k_+-k_-}} \right] d\tilde{q} . \end{aligned}$$

Following the strategy of the proof of (9.701) in Lemma 9.10, using Lemma 9.16 and the uniform bounds of ${\left| \left| \left| [f^\pm _{n-1},\Omega _{n-1},r_{n-1}] \right| \right| \right| }$ and ${\left| \left| \left| [f^\pm _{n},\Omega _{n},r_{n}] \right| \right| \right| }$, we deduce

9.777 $$\begin{aligned} \Vert m_n-m_{n-1}\Vert _\infty \le c_6 \frac{\delta }{q_0}(\Vert \triangle f_{n}\Vert _\infty + \Vert \triangle \Omega _{n}\Vert _\infty +\Vert q^{-2}\triangle r_{n}\Vert _\infty + \Vert q^{-1}\partial _q\triangle r_{n}\Vert _\infty ). \end{aligned}$$

Thus the $S_5$ term gives the desired estimates and hence

9.778 $$\begin{aligned} \Vert q^{1+\theta }\partial _q\triangle \Omega _{n+1}\Vert _\infty \le c_7 \frac{\delta }{q_0}(\Vert \triangle f_{n}\Vert _\infty + \Vert \triangle \Omega _{n}\Vert _\infty +\Vert q^{-2}\triangle r_{n}\Vert _\infty + \Vert q^{-1}\partial _q\triangle r_{n}\Vert _\infty ) \end{aligned}$$

for some constant $c_7>0$ independent of *n*. The estimation of $\Vert \triangle \Omega _{n+1}\Vert _\infty$ directly follows from $\triangle \Omega _{n+1}(p,q) = \int _{q^*}^q\partial _q\triangle \Omega _{n+1} (p,\tilde{q})d\tilde{q}$ and (9.778):

9.779 $$\begin{aligned} \Vert \triangle \Omega _{n+1}\Vert _\infty \le c_8 \frac{\delta }{q_0}(\Vert \triangle f_{n}\Vert _\infty + \Vert \triangle \Omega _{n}\Vert _\infty +\Vert q^{-2}\triangle r_{n}\Vert _\infty + \Vert q^{-1}\partial _q\triangle r_{n}\Vert _\infty ) \end{aligned}$$

for some constant $c_8>0$ independent of *n*. Collecting (9.765), (9.767), (9.770), (9.771), (9.778), (9.779), we obtain (9.749). $\square$

We will now finish the proof of Theorem 9.4.

##### Proof of Theorem 9.4

*Convergence and uniqueness.* By Proposition 9.11 and Proposition 9.14, the iterates $[f^\pm _{n},\Omega _{n},r_{n}]_{n\in \mathbb N}$ satisfy the uniform bounds ${\left| \left| \left| [f^\pm _{n},\Omega _{n},r_{n}] \right| \right| \right| }\le 2A{\left| \left| \left| [\hat{f}^\pm , \hat{\Omega }, \hat{r}]|_{\underline{\mathcal {C}}\cup \mathcal {C}} \right| \right| \right| }$ for all $n\in \mathbb N$. Moreover $\Vert \triangle f_{n}^\pm \Vert _\infty + \Vert q^{-2}{\triangle r_{n}}\Vert _\infty + \Vert q^{-1}{\partial _q\triangle r_{n}}\Vert _\infty +\Vert \triangle \Omega _{n}\Vert _\infty + \Vert q^{1+\theta }\partial _q\triangle \Omega _{n}\Vert _\infty \rightarrow 0$ as $n\rightarrow \infty$, which in turn implies the strong convergence in $C^1$ for $f^\pm _n$ and in $C^2$ for $[\Omega _n, \frac{r_n}{q}]$ by standard interpolation. Hence, as $n\rightarrow \infty$, $[\Omega _{n},\frac{r_{n}}{q}]$ converges to $[\Omega ,\frac{r}{q}]$ strongly in $C^2$ and $f^\pm _n$ to $f^\pm$ in $C^1$. Using the strong convergence shown above, the fundamental theorem of calculus, and the formulas (9.722), (9.728)–(9.729), (9.737), (9.740)–(9.741), and (9.745), we may pass to the limit as $n\rightarrow \infty$ to conclude that $\partial _{p}r(p,\cdot ), \partial _{p}\Omega (p,\cdot )\in W^{2,\infty }([q_0,\infty ))$ and $\partial _{p}f(p,\cdot )\in W^{1,\infty }([q_0,\infty ))$. In particular, using (9.697) and (9.710), upon passing to the limit we obtain a classical solution of (2.83). After passing to the limit in (9.709)-(9.711), we see that $[f^\pm ,\Omega ,\frac{r}{q}]$ is the desired classical solution to (2.83)–(2.84) and (9.648)–(9.649) in $\mathcal {D}$. Note that in fact $\partial _{pp}r(p,\cdot ),\partial _{pp}\Omega (p,\cdot )\in W^{1,\infty }([q_0,\infty ))$, which follows easily from (2.84), (9.697) and the bootstrap argument (involving one application of the $\partial _{q}$-derivative) similarly to above.

Observe that $f^\pm \in W^{2,\infty }$ and $[\Omega ,r]\in W^{3,\infty }$ by standard weak-* convergence arguments, since the iterates are uniformly bounded in the same spaces. Uniqueness easily follows an adaptation of the difference estimates of Proposition 9.14. To show that the solution corresponds to the solution of the original problem (2.83)–(2.88), it only remains to show that the constraint equations (2.86)–(2.85) are satisfied on the interior of the domain $\mathcal {D}$. This is a standard argument, which follows from the observation that for the solutions of (2.83)–(2.84) and (2.87)–(2.88) necessarily $\partial _{p}\left( \partial _{q}\left( \Omega ^{-2}\partial _{q}r\right) + \pi r \Omega ^2 T^{pp}\right) =0$ and $\partial _q\left( \partial _{p}\left( \Omega ^{-2}\partial _{p}r\right) + \pi r \Omega ^2 T^{qq}\right) =0$. Since the constraints are satisfied by the characteristic data, this gives the claim. Note that these expressions make sense given the above shown regularity.

*Asymptotic flatness.* To show the asymptotic flatness we must show that $\lim _{q\rightarrow \infty }m(p,q)<\infty$ for all $p\in [p_0,p_0+\delta ]$. By (9.670) this follows if we can show that $\rho \partial _{q}r$ and $\rho \Omega ^2 (u^{p})^2\partial _{p}r$ are integrable on $[q_0,\infty )$. Note that $\Vert \partial _{q}r\Vert _{L^\infty ([q_0,\infty ))}<\infty$ by the boundedness of our norms. Moreover, by integrating (9.697) with respect to $q$ and using $q\lesssim r\lesssim q$ and the boundedness of $\Omega$, we conclude that $\Vert \partial _{p}r\Vert _{\infty }\lesssim 1.$ From (9.653) we have

9.780 $$\begin{aligned} \begin{aligned} \rho =\frac{ (f^+ f^-)^{\frac{1}{k_+-k_-}}}{(1-\varepsilon ) r^{2+2\eta }}&=\frac{1}{1-\varepsilon } q^{-\frac{N_++N_-}{k_+-k_-} -2-2\eta } (\frac{q}{r})^{2+2\eta } (q^{N_+}f^+ q^{N_-} f^-)^{\frac{1}{k_+-k_-}} \\&\le \frac{1}{1-\varepsilon } q^{-3-\beta } \left\| \frac{q}{r}\right\| _\infty ^{2+2\eta }(\Vert q^{N_+} f^+\Vert _\infty \Vert q^{N_-} f^-\Vert _\infty )^{\frac{1}{k_+-k_-}}, \end{aligned} \end{aligned}$$

where we recall $\beta$ from (9.688). Therefore $\rho (p,\cdot )\in L^1([q_0,\infty ))$ since ${\left| \left| \left| [f^\pm , \Omega , r] \right| \right| \right| }<\infty$. To show that $\rho \Omega ^2 (u^{p})^2$ is integrable, we observe that

$$\begin{aligned} \rho \Omega ^2 (u^{p})^2 = \frac{ (f^+ f^-)^{\frac{1}{k_+-k_-}}}{(1-\varepsilon ) r^{2+2\eta }} \frac{1}{1+\eta }\frac{r^{2\eta }}{\Omega ^2} \left( \frac{f^-}{f^+}\right) ^{\frac{1}{2}} = \frac{ (f^+)^{\frac{1}{k_+-k_-}-\frac{1}{2}} (f^-)^{\frac{1}{k_+-k_-}+\frac{1}{2}}}{(1+\varepsilon ) \Omega ^2 r^{2}} . \end{aligned}$$

Using (2.82) and (9.654), we also have the expression

9.781 $$\begin{aligned} \Omega ^2 (u^{p})^2 = \frac{1}{1+\eta }\frac{r^{2\eta }}{\Omega ^2} \left( \frac{f^-}{f^+}\right) ^{\frac{1}{2}} \lesssim q^{2\eta +\frac{N_+-N_-}{2}}\left( \frac{q^{N_-}f^-}{q^{N_+}f^+}\right) ^{\frac{1}{2}} \lesssim 1, \end{aligned}$$

where we have used (9.660), (9.676), and the boundedness of our norms. Therefore, from (9.780) we conclude $\rho \Omega ^2 (u^{p})^2\in L^1([q_0,\infty ))$, and the spacetime is therefore asymptotically flat. $\square$

### Proof of Theorem 1.2: Existence of Naked Singularities

Recall the discussion of naked singularities in the introduction and in Section 2.7.3. Following [32], a spacetime contains a naked singularity if it corresponds to a maximal hyperbolic development of suitably regular data, and the future null-infinity is geodesically incomplete. The latter statement does not actually require the construction of future null-infinity as an idealised boundary attached to a suitable spacetime compactification. Instead, we define it to mean that affine length of a sequence of maximal ingoing null geodesics initiated along a sequence of points (approaching infinity) along an asymptotically flat outgoing null-surface, and suitably normalised, is uniformly bounded by some positive constant.

*Proof of Theorem* 1.2. For any $\varepsilon \in (0,\varepsilon _0]$ we consider the associated RLP spacetime $(\mathcal {M}_{\text {RLP},\varepsilon },g_{\text {RLP},\varepsilon })$ given in Definition 2.7. We use it to prescribe the data for the characteristic problem in the region $\mathcal {D}$ as described in Section 9.2. The associated solution to the Einstein-Euler system exists in region $\mathcal {D}$ by Theorem 9.4. Since $\mathcal {U}_{\pm }=1+O(\sqrt{\varepsilon })>0$, and since the data are exactly selfsimilar on $\underline{\mathcal {C}}$ and on the finite segment $\{(p_0,q) \ \big |\,\ q\in [q_0,q_0+A_0]\}\subset \mathcal {C}$, we conclude that the solution coincides with the selfsimilar RLP-solution in the region $\mathcal {D}_{A_0} = \{(p,q)\,\big | \, p_0\le p<0, \, q_0\le q<q_0+A_0\}$, see Figure 7. We now consider a new spacetime $(\mathcal {M}_\varepsilon ,g_\varepsilon )$ obtained by gluing together the solution in the region $\mathcal {D}$ and to the past of the ingoing null-segment $\underline{\mathcal {C}}$ inside $\mathcal {M}_{\text {RLP},\varepsilon }$. Clearly, the new spacetime is identical to the RLP spacetime (and therefore smooth) in an open neighbourhood across $\underline{\mathcal {C}}$. It therefore coincides with the exact selfsimilar RLP-spacetime $(\mathcal {M}_{\text {RLP},\varepsilon },g_{\text {RLP},\varepsilon })$ in the past of $\underline{\mathcal {C}}$.

The exterior region, viewed as a development of the characteristic problem with data prescribed along the semi-infinite rectangle with outgoing data prescribed on $\{q\ge 0\}$ and ingoing data on $p\in [p_0,0)$ is maximal, as the ingoing null-curve $\mathcal {N}$ is incomplete on approach to the singularity. In fact, since along backward null-cone $\mathcal {N}$ we have $\frac{R}{-\sqrt{\varepsilon }\tau }=y_{\mathcal {N}}$, we have

9.782 $$\begin{aligned} \lim _{\begin{array}{c} \tau \rightarrow 0^- \\ (\tau ,R)\in \mathcal {N} \end{array}} \rho (\tau , R) = \frac{1}{2\pi \tau ^2} \Sigma (y_\mathcal {N}) = \infty , \end{aligned}$$

since $\Sigma (y_{\mathcal {N}})\ne 0$, where we recall that the density $\Sigma$ is in fact strictly positive on $[0,\infty )$. Therefore, by (2.75) the Ricci scalar blows up as the observer approaches the scaling origin $\mathcal {O}$ along $\mathcal {N}$.

It remains to show that the future null-infinity is incomplete in the sense of Definition 1.1. from [32]. Consider now a sequence of points $(p_0,q_n)$ such that $\lim _{n\rightarrow \infty }q_n=\infty$. For any $n\in \mathbb N$, consider the future oriented ingoing radial null-geodesic emanating from $(p_0,q_n)$. Let the affine parameter^4^ be denoted by $\ell$. Then $q(\ell )\equiv q_n$, and the angular coordinates are also constant. The $p$-component satisfies the ODE

9.783 $$\begin{aligned} 0 = \ddot{p}(\ell ) + \Gamma ^p_{pp} (\dot{p}(\ell ))^2 = \ddot{p}(\ell ) + \partial _{p}\log (\Omega ^2)(\dot{p}(\ell ))^2, \end{aligned}$$

where we have used (3.869). By our assumptions $p(0)=p_0$ and we normalise the tangent vector to be parallel to $\partial _{p}$ so that at $(p_0,q_n)$, we have $-2 = g(\partial _{q}, \dot{p}(0)\partial _{p}) = - \dot{p}(0) \frac{1}{2}\Omega _n^2$, where $\Omega _n: = \Omega (p_0,q_n)$ for all $n\in \mathbb N$. Therefore $\dot{p}(0) = 4\Omega _n^{-2}.$ We may integrate (9.783) once to conclude that $\frac{d}{d\ell }(-\frac{1}{\dot{p}}+\log (\Omega ^2))=0$ and therefore

9.784 $$\begin{aligned} -\frac{1}{\dot{p}}+\log (\Omega ^2) = -\frac{\Omega _n^2}{4} + \log (\Omega _n^2). \end{aligned}$$

It then follows that

9.785 $$\begin{aligned} \dot{p}(\ell ) = \frac{1}{\frac{\Omega _n^2}{4} -2\log \left( \frac{\Omega _n}{\Omega (p(\ell ),q_n)}\right) }. \end{aligned}$$

However, by the proof of Theorem 9.4 there exists a constant *C* which depends only on the data ${\left| \left| \left| [f^\pm , \Omega , r]|_{\underline{\mathcal {C}}\cup \mathcal {C}} \right| \right| \right| }$ such that $\Vert \partial _{p}\log \Omega \Vert _{L^\infty (\mathcal {D})}\le C$. In particular, by the mean value theorem $\log \left( \frac{\Omega _n}{\Omega (p(\ell ),q_n)}\right) \le C |p-p_0| \le C\delta$, for any $p\in [p_0,0)$ and $n\in \mathbb N$. Note further that by (9.666) and our bounds on the data along $\mathcal {C}$, $\Omega _n^2$ is bounded from below and above uniformly in *n*. For $\delta \ll 1$ sufficiently small, we conclude that $\dot{p}$ remains positive and $p$ reaches 0 (i.e. the Cauchy horizon) in finite $\ell$-time, independent of *n*. $\square$

#### Remark 9.17

(The Cauchy horizon) By construction, the Cauchy horizon coincides with the null-curve $\{p=0\}$.

#### Remark 9.18

By construction, for any $\varepsilon \in (0,\varepsilon _0]$ there exists in fact an infinite family of naked singularity solutions. This freedom comes from the essentially arbitrary choice of the truncation in the region $\mathcal {D}$, modulo the size and decay limitations imposed by the local existence theorem, Theorem 9.4. In a neighbourhood of the scaling origin $\mathcal {O}$, our solutions are however exactly selfsimilar.

## Data Availability

All data generated or analysed during this study are included in this published article.

## Proof of Local Existence Around the Sonic Point: Combinatorial Argument

The main goal of the rest of this section is to show the convergence of the power series $\sum _{N=0}^\infty \mathcal {R}_N (\delta x)^N$ and $\sum _{N=0}^\infty \mathcal {W}_N (\delta x)^N$. We will do so by induction on the coefficients $\mathcal {R}_N$ and $\mathcal {W}_N$. Before proceeding, we record some technical lemmas. The proofs of Lemmas A.1–A.4 below are given in detail in [16] and we therefore only state the lemmas without proof for reader’s convenience.

### Lemma A.1

There exists a constant $c>0$ such that for all $N\in \mathbb N$, the following holds

1.786 $$\begin{aligned} \sum _{\begin{array}{c} \ell +m=N\\ \ell ,m \ge 1 \end{array}} \frac{1}{\ell ^{3} m^{3}} \le \frac{c}{N^3}, \end{aligned}$$

1.787 $$\begin{aligned} \sum _{\begin{array}{c} \ell +m=N\\ \ell ,m \ge 1 \end{array}} \frac{1}{\ell ^{3} m^{2}} \le \frac{c}{N^2} , \end{aligned}$$

1.788 $$\begin{aligned} \sum _{\begin{array}{c} \ell +m+n=N\\ \ell , m, n \ge 1 \end{array}} \frac{1}{\ell ^{3} m^{3} n^{3}} \le \frac{c}{N^3}, \end{aligned}$$

1.789 $$\begin{aligned} \sum _{\begin{array}{c} \ell +m+n=N\\ \ell , m, n \ge 1 \end{array}} \frac{1}{\ell ^{3} m^{2} n^{3}} \le \frac{c}{N^2}. \end{aligned}$$

### Lemma A.2

There exists a constant $c>0$ such that for all $N\ge 3$ and all $C\ge 2$, the following holds

1.790 $$\begin{aligned} \sum _{\ell =2}^{N-1} \frac{C^{\ell -1}}{\ell ^q} \le c \frac{C^{N-2}}{N^q}, \quad q=2, 3. \end{aligned}$$

For any $\alpha \in \mathbb R$, we let

$$\begin{aligned} \left( {\begin{array}{c}\alpha \\ j\end{array}}\right) =\frac{(\alpha )_j}{j!}= \frac{\alpha (\alpha -1)\cdots (\alpha -j+1)}{j!} \ \ \text {for} \ \ j\in \mathbb N, \ \ \text {and} \ \ \left( {\begin{array}{c}\alpha \\ 0\end{array}}\right) =1. \end{aligned}$$

### Lemma A.3

Recall the set $\pi (n,m)$ defined in (4.188).

1. For each $$n\in \mathbb N$$,
  1.791 $$\begin{aligned} \sum _{m=1}^n\sum _{\begin{array}{c} \pi (n,m) \end{array} } \frac{(-1)^m m! }{ \lambda _1 ! \dots \lambda _n!} \left( {\begin{array}{c}\frac{1}{2}\\ 1\end{array}}\right) ^{\lambda _1} \cdots \left( {\begin{array}{c}\frac{1}{2}\\ n\end{array}}\right) ^{\lambda _n} = 2 (n+1)\left( {\begin{array}{c}\frac{1}{2}\\ n+1\end{array}}\right) \end{aligned}$$
  holds.
2. There exist universal constants $c_1,c_2>0$ such that
  1.792 $$\begin{aligned} c_1 \frac{1}{n^\frac{3}{2}}\le (-1)^{n-1} \left( {\begin{array}{c}\frac{1}{2}\\ n\end{array}}\right) \le c_2 \frac{1}{n^\frac{3}{2}}, \quad n\in \mathbb N . \end{aligned}$$

### Lemma A.4

Let $p>0$ be a given positive number. Let $(\lambda _1,\dots , \lambda _\ell ) \in \pi (\ell ,m)$ where $1\le m\le \ell$ and $\ell \ge 2$ be given.

1. If $1\le m\le \left[ \tfrac{\sqrt{\ell }}{\sqrt{3}}\right]$, there exists a constant $c_3=c_3(p)>0$ such that
  1.793 $$\begin{aligned} \prod _{n=1}^\ell \left( \frac{1}{n^{\lambda _n}}\right) ^p \le \frac{c_3}{\ell ^{p}}. \end{aligned}$$
2. There exist $c_4=c_4(p)>0$ and $L_0=L_0(p)>1$ such that if $L\ge L_0$, the following holds:
  1.794 $$\begin{aligned} \frac{1}{L^{m-1}}\prod _{n=1}^\ell \left( \frac{1}{n^{\lambda _n}} \right) ^p \le \frac{c_4}{\ell ^{p}}, \text { for all } 1\le m \le \ell . \end{aligned}$$
3. Let $\ell \ge 3$. Then there exists $c_5=c_5(p)>0$ such that if $L\ge L_0$, the following holds:
  1.795 $$\begin{aligned} \frac{1}{L^{m-2}}\prod _{n=1}^\ell \left( \frac{1}{n^{\lambda _n}} \right) ^p \le \frac{c_5}{\ell ^{p}}, \text { for all } 2\le m \le \ell . \end{aligned}$$

By Lemmas 4.1 and 4.6 there exist constants $0<m<M<1$ such that

1.796 $$\begin{aligned}&|\mathcal {R}_0|, \ |\mathcal {W}_0|, \ |\mathcal {R}_1|, \ |\mathcal {W}_1| \le M, \end{aligned}$$

1.797 $$\begin{aligned}&\mathcal {R}_0>m, \end{aligned}$$

for all $\varepsilon \in (0,\varepsilon _0]$, with $\varepsilon _0>0$ chosen sufficiently small as in Lemma 4.1.

### Lemma A.5

Let $x_*\in [x_{\text {crit}}+\kappa ,x_{\text {max}}]$ and $\alpha \in (1,2)$. Assume that

1.798 $$\begin{aligned} |\mathcal {R}_m| \le \frac{C^{m-\alpha }}{m^3}, \quad 2\le m\le N-1, \end{aligned}$$

1.799 $$\begin{aligned} |\mathcal {W}_m| \le \frac{C^{m-\alpha }}{m^3}, \quad 2\le m\le N-1, \end{aligned}$$

for some $C\ge 1$ and $N\ge 3$. Then there exists a constant $\hat{C}=\hat{C}(M)>0$ such that

1.800 $$\begin{aligned} |(\mathcal {W}^2)_\ell | +|(\mathcal {R}\mathcal {W})_\ell | +|(\mathcal {R}^2)_\ell |&\le {\left\{ \begin{array}{ll} \hat{C} &{} \ \text { if } \ \ell =0,1,\\ \hat{C} \frac{C^{\ell -\alpha }}{\ell ^3} &{} \ \text { if } \ 2\le \ell \le N-1, \end{array}\right. } \end{aligned}$$

1.801 $$\begin{aligned} |H_\ell |&\le {\left\{ \begin{array}{ll} \hat{C} &{} \ \text { if } \ \ell =0,1, \\ \hat{C} (1+\varepsilon ) \frac{C^{\ell -\alpha }}{\ell ^3} &{} \ \text { if } \ 2\le \ell \le N-1. \end{array}\right. } \end{aligned}$$

### Proof

We first prove the bounds for $|(\mathcal {W}^2)_\ell |$, $\ell \ge 0$. The bounds $|(\mathcal {W}^2)_0|\le M^2$ and $|(\mathcal {W}^2)_1|\le 2M^2$ are obvious from (1.796). Clearly

1.802 $$\begin{aligned} |(\mathcal {W}^2)_2|\le 2M|\mathcal {W}_2|+M^2 \le 2M \frac{C^{2-\alpha }}{2^3}+M^2\le (2M +2^3 M^2) \frac{C^{2-\alpha }}{2^3} \end{aligned}$$

where we have used $C^{2-\alpha }\ge 1$. If $\ell \ge 3$ we then have

1.803 $$\begin{aligned} |(\mathcal {W}^2)_\ell |&\le \sum _{m=0}^\ell |\mathcal {W}_m||\mathcal {W}_{\ell -m}| \nonumber \\&\le 2|\mathcal {W}_0||\mathcal {W}_\ell | + 2|\mathcal {W}_1||\mathcal {W}_{\ell -1}| + \sum _{m=2}^{\ell -2}|\mathcal {W}_m||\mathcal {W}_{\ell -m}| \nonumber \\&\le 2M \frac{C^{\ell -\alpha }}{\ell ^3} + 2M \frac{C^{\ell -1-\alpha }}{(\ell -1)^3} + \sum _{m=2}^{\ell -2} \frac{C^{\ell -2\alpha }}{m^3(\ell -m)^3} \nonumber \\&\le 2M C^{\ell -\alpha } \left( \frac{1}{\ell ^3} + \frac{1}{(\ell -1)^3} + \frac{1}{2M}\sum _{m=2}^{\ell -2} \frac{1}{m^3(\ell -m)^3} \right) \nonumber \\&\le 2M \tilde{C} \frac{C^{\ell -\alpha }}{\ell ^3}, \end{aligned}$$

for some constant $\tilde{C}$. It is now clear, that the estimates for $(\mathcal {R}\mathcal {W})_\ell$ and $(\mathcal {R}^2)_\ell$, $\ell \ge 0$ follow in the same way, as the only estimates we have used are (1.796) and the inductive assumptions (1.798)–(1.798), which both depend only on the index, and are symmetric with respect to $\mathcal {R}$ and $\mathcal {W}$. Recalling $H_\ell$ from (4.194), the bounds (1.801) now immediately follow from (1.800) and (1.799). $\square$

### Lemma A.6

Let $x_*\in [x_{\text {crit}}+\kappa ,x_{\text {max}}]$ and $\alpha \in (1,2)$. Assume that (1.798) and (1.799) for $N\ge 3$ and some large enough $C>1$ satisfying

1.804 $$\begin{aligned} C >\frac{4L_0}{c_1} \end{aligned}$$

where $c_1$ and $L_0=L_0(\frac{3}{2})$ are universal constants in (1.792) and Lemma A.4. Then there exists a constant $\hat{C}=\hat{C}(\mathcal {R}_0)$ such that

1.805 $$\begin{aligned} |(\mathcal {R}^{-\eta })_\ell |&\le {\left\{ \begin{array}{ll} \hat{C} &{} \ \text { if } \ \ell =1, \\ \hat{C} \left( \frac{C^{\ell -\alpha }}{\ell ^3} + \frac{C^{\ell -2}}{\ell ^2}\right) &{} \ \text { if } \ 2\le \ell \le N-1. \end{array}\right. } \end{aligned}$$

### Proof

When $\ell =1$, $|(\mathcal {R}^{-\eta })_1| = | - \eta \mathcal {R}_0^{-\eta -1} \mathcal {R}_1 | \le \mathcal {R}_0^{-\eta -1}.$ When $\ell =2$ it is easy to see from (4.190) that

1.806 $$\begin{aligned} (\mathcal {R}^{-\eta })_2 = -\eta \mathcal {R}_0^{-\eta }\left( \mathcal {R}_0^{-1}R_2 -(\eta +1)\mathcal {R}_0^{-2}\mathcal {R}_1^2\right) . \end{aligned}$$

In particular, $\left| (\mathcal {R}^{-\eta })_2 \right| \le c m^{-\eta -2}\left( R_2 + \mathcal {R}_1^2\right) \le c m^{-\eta -2}\left( \frac{C^{2-\alpha }}{2^3} + M^2\right) \le \hat{C} \left( \frac{C^{2-\alpha }}{2^3} + \frac{1}{2^2}\right)$ for some universal constant $\hat{C}$ and the claim is thus clear for $\ell =2$. For $\ell \ge 3$, we rewrite $(\mathcal {R}^{-\eta })_\ell$ in the form

1.807 $$\begin{aligned} (\mathcal {R}^{-\eta })_\ell = -\eta \mathcal {R}_0^{-\eta -1} \mathcal {R}_\ell + \mathcal {R}_0^{-\eta } \sum _{m=2}^\ell \frac{1}{\mathcal {R}_0^m}\sum _{\pi (\ell ,m)} (- \eta )_m \frac{1}{\lambda _1 ! \dots \lambda _\ell !} {\mathcal {R}_1}^{\lambda _1}\dots {\mathcal {R}_\ell }^{\lambda _\ell }. \end{aligned}$$

Now clearly, by the inductive assumption

1.808 $$\begin{aligned} \left| -\eta \mathcal {R}_0^{-\eta -1} \mathcal {R}_\ell \right| \le \hat{C} \frac{C^{\ell -\alpha }}{\ell ^2} \end{aligned}$$

for a universal constant $\hat{C}>0$. To bound the second summand on the right-hand side of (1.807), we use (1.798) and Lemma A.3 first to conclude

$$\begin{aligned} \begin{aligned} \left| {\mathcal {R}_1}^{\lambda _1}\dots {\mathcal {R}_\ell }^{\lambda _\ell } \right| =\left| \prod _{n=1}^\ell R_n^{\lambda _n} \right|&\le \left( \tfrac{1}{1^3}\right) ^{\lambda _1} \left( \tfrac{C^{2-\alpha }}{2^3}\right) ^{\lambda _2} \dots \left( \tfrac{C^{\ell -\alpha }}{\ell ^3}\right) ^{\lambda _\ell } \\&= C^{(\alpha -1)\lambda _1 + \sum _{i=1}^\ell (i\lambda _i -\alpha \lambda _i)}\left[ \prod _{n=1}^\ell \left( \frac{1}{n^{\frac{3}{2}}}\right) ^{\lambda _n} \right] \left[ \prod _{n=1}^\ell \left( \frac{1}{n^{\lambda _n}}\right) ^\frac{3}{2} \right] \\&\le C^{(\alpha -1)m} C^{\ell -\alpha m} c_1^{-m}\left[ \prod _{n=1}^\ell \left( (-1)^{n-1} \left( {\begin{array}{c}\frac{1}{2}\\ n\end{array}}\right) \right) ^{\lambda _n} \right] \left[ \prod _{n=1}^\ell \left( \frac{1}{n^{\lambda _n}}\right) ^\frac{3}{2}\right] \end{aligned} \end{aligned}$$

where we have used $(\alpha -1)\lambda _1\le (\alpha -1)m$ in the third line since $\alpha >1$ and $\lambda _1\le m$. Hence, using $|(- \eta )_m| \le m!$, we observe that

1.809 $$\begin{aligned} \begin{aligned}&\left| (- \eta )_m \frac{1}{\lambda _1 ! \dots \lambda _\ell !} {\mathcal {R}_1}^{\lambda _1}\dots {\mathcal {R}_\ell }^{\lambda _\ell }\right| \\&\quad \le C^{\ell -m} c_1^{- m } (-1)^{\ell } \frac{(-1)^m m!}{\lambda _1 ! \dots \lambda _\ell !} \left( {\begin{array}{c}\frac{1}{2}\\ 1\end{array}}\right) ^{\lambda _1}\dots \left( {\begin{array}{c}\frac{1}{2}\\ \ell \end{array}}\right) ^{\lambda _\ell } \left[ \prod _{n=1}^\ell \left( \frac{1}{n^{\lambda _n}}\right) ^\frac{3}{2}\right] . \end{aligned} \end{aligned}$$

In turn by recalling (4.190) and using Lemma A.3 and Lemma A.4 with $p=\frac{3}{2}$, we see that

1.810 $$\begin{aligned} \begin{aligned}&\left| \mathcal {R}_0^{-\eta } \sum _{m=2}^\ell \frac{1}{\mathcal {R}_0^m}\sum _{\pi (\ell ,m)} (- \eta )_m \frac{1}{\lambda _1 ! \dots \lambda _\ell !} {\mathcal {R}_1}^{\lambda _1}\dots {\mathcal {R}_\ell }^{\lambda _\ell }\right| \\&\le \mathcal {R}_0^{-\eta } \frac{C^\ell (-1)^\ell }{(c_1C\mathcal {R}_0)^2} \sum _{m=2}^\ell \sum _{\pi (\ell ,m)} \left[ \frac{1}{(c_1C\mathcal {R}_0)^{m-2}} \prod _{n=1}^\ell \left( \frac{1}{n^{\lambda _n}}\right) ^\frac{3}{2}\right] \frac{(-1)^m m!}{\lambda _1 ! \dots \lambda _\ell !} \left( {\begin{array}{c}\frac{1}{2}\\ 1\end{array}}\right) ^{\lambda _1}\dots \left( {\begin{array}{c}\frac{1}{2}\\ \ell \end{array}}\right) ^{\lambda _\ell } \\&\le \mathcal {R}_0^{-\eta } \frac{C^\ell }{(c_1C\mathcal {R}_0)^2}\frac{c_4}{\ell ^\frac{3}{2}} (-1)^\ell 2 (\ell +1) \left( {\begin{array}{c}\frac{1}{2}\\ \ell +1\end{array}}\right) \\&\le \mathcal {R}_0^{-\eta -1} \tfrac{2c_2c_4}{c_1^2}\tfrac{C^{\ell -2}}{\ell ^\frac{3}{2}(\ell +1)^\frac{1}{2}} \end{aligned} \end{aligned}$$

where *C* is large enough so that (1.804) holds. This proves (1.805). $\square$

### Remark A.7

In (1.804) the constant 4 in the numerator ensures that $C>\frac{L_0}{c_1 \mathcal {R}_0}$ for all $x_*\in [x_{\text {crit}}+\kappa ,x_{\text {max}}]$, since for $\varepsilon _0>0$ sufficiently small, there exists a constant $0<\delta <1$ such that $\frac{1}{\mathcal {R}_0}<3+\delta$ for all $\varepsilon \in (0,\varepsilon _0]$.

### Lemma A.8

Let $x_*\in [x_{\text {crit}}+\kappa ,x_{\text {max}}]$ and $\alpha \in (1,2)$. Then there exists a constant $C_*>0$ such that if $C>C_*$ and for any $N\ge 3$, the following assumptions hold

1.811 $$\begin{aligned} |\mathcal {R}_m| \le \frac{C^{m-\alpha }}{m^3}, \quad 2\le m\le N-1, \end{aligned}$$

1.812 $$\begin{aligned} |\mathcal {W}_m| \le \frac{C^{m-\alpha }}{m^3}, \quad 2\le m\le N-1, \end{aligned}$$

then we have

1.813 $$\begin{aligned} |\mathcal {S}_N|&\le \beta \frac{C^{N-\alpha }}{N^2}\left[ \frac{1}{C^{\alpha -1}}+ \frac{1}{C^{2-\alpha }} +\frac{1}{C} \right] , \end{aligned}$$

1.814 $$\begin{aligned} |\mathcal {V}_N|&\le \beta \frac{C^{N-\alpha }}{N^2}\left[ \frac{1}{C^{\alpha -1}}+ \frac{1}{C^{2-\alpha }} + \frac{1}{CN} \right] , \end{aligned}$$

for some universal constant $\beta >0$.

### Proof

We start with (1.813). Recall (4.255). First we show

1.815 $$\begin{aligned} \left| \mathcal {R}_1\mathcal {R}_0^{-\eta } \sum _{m=2}^N \frac{1}{\mathcal {R}_0^m}\sum _{\pi (N,m)} (- \eta )_m \frac{1}{\lambda _1 ! \dots \lambda _N !} {\mathcal {R}_1}^{\lambda _1}\dots {\mathcal {R}_N}^{\lambda _N}\right| \lesssim \frac{C^{N-2}}{N^2}. \end{aligned}$$

Note that $\lambda _N=0$ and thus (1.815) does not depend on $\mathcal {R}_N$. As in (1.809), using (1.811) and Lemma A.3, we have

$$\begin{aligned} \begin{aligned}&\left| (- \eta )_m \frac{1}{\lambda _1 ! \dots \lambda _N!} {\mathcal {R}_1}^{\lambda _1}\dots {\mathcal {R}_N}^{\lambda _N}\right| \\&\quad \le C^{N-m} c_1^{- m } (-1)^{N} \frac{(-1)^m m!}{\lambda _1 ! \dots \lambda _N !} \left( {\begin{array}{c}\frac{1}{2}\\ 1\end{array}}\right) ^{\lambda _1}\dots \left( {\begin{array}{c}\frac{1}{2}\\ N\end{array}}\right) ^{\lambda _N} \left[ \prod _{n=1}^N \left( \frac{1}{n^{\lambda _n}}\right) ^\frac{3}{2}\right] . \end{aligned} \end{aligned}$$

Hence by using (1.795) of Lemma A.4, the left-hand side of (1.815) is bounded by

$$\begin{aligned} \begin{aligned}&\text {LHS of (1.815) }\\&\le \mathcal {R}_1\mathcal {R}_0^{-\frac{2k}{1-k}} C^N \sum _{m=2}^N \sum _{\pi (N,m)} \frac{(-1)^N}{(c_1C\mathcal {R}_0)^m} \left[ \prod _{n=1}^N \left( \frac{1}{n^{\lambda _n}}\right) ^\frac{3}{2}\right] \frac{(-1)^m m!}{\lambda _1 ! \dots \lambda _l !} \left( {\begin{array}{c}\frac{1}{2}\\ 1\end{array}}\right) ^{\lambda _1}\dots \left( {\begin{array}{c}\frac{1}{2}\\ N\end{array}}\right) ^{\lambda _N} \\&\le \mathcal {R}_1\mathcal {R}_0^{-\frac{2k}{1-k}} \frac{C^N}{(c_1C\mathcal {R}_0)^2}\frac{c_5}{N^\frac{3}{2}}(-1)^N 2(N+1) \left( {\begin{array}{c}\frac{1}{2}\\ N+1\end{array}}\right) \\&\le \mathcal {R}_1\mathcal {R}_0^{-\frac{2k}{1-k}-2} \frac{2c_2c_5}{c_1^2} \frac{C^{N-2}}{N^\frac{3}{2} (N+1)^\frac{1}{2}} \end{aligned} \end{aligned}$$

which shows (1.815). To estimate the second term on the right-hand side of (4.255), we use (1.811), (1.812) and (1.786) to obtain

1.816 $$\begin{aligned} \begin{aligned}&\left| x_*^2 \mathcal {R}_1 \Big [ (1-\varepsilon ) \sum _{\begin{array}{c} \ell +m =N\\ 1\le m\le N-1 \end{array}} \mathcal {W}_\ell \mathcal {W}_m+ \sum _{\begin{array}{c} \ell +m =N\\ 1\le m\le N-1 \end{array}} 4\varepsilon \mathcal {R}_\ell \mathcal {W}_m\Big ]\right| \\&\le \left| x_*^2 \mathcal {R}_1 \Big [ 2(1-\varepsilon )\mathcal {W}_1\mathcal {W}_{N-1} + 4\varepsilon (\mathcal {R}_1W_{N-1}+\mathcal {R}_{N-1}\mathcal {W}_1) \Big ] \right| \\&\quad + \left| x_*^2 \mathcal {R}_1 \Big [ (1-\varepsilon ) \sum _{m=2}^{N-2}\mathcal {W}_{N-m} \mathcal {W}_m + \sum _{m=2}^{N-2} 4\varepsilon \mathcal {R}_{N-m} \mathcal {W}_m\Big ]\right| \\&\lesssim (1+\varepsilon ) \frac{C^{N-1-\alpha }}{N^{3}} + (1+\varepsilon ) \sum _{m=2}^{N-2} \frac{C^{N-2\alpha }}{(N-m)^3 m^3}\lesssim (1+\varepsilon )\frac{C^{N-1-\alpha }}{N^3}. \end{aligned} \end{aligned}$$

Finally, we estimate the last term on the right-hand side of (4.255) - the expression $\tilde{\mathcal {S}}_N$ - given by (4.256). To treat the first line of (4.256), by (1.811) and (1.805), we obtain

1.817 $$\begin{aligned} \begin{aligned} \left| \sum _{\begin{array}{c} \ell +m=N \\ 1\le m\le N-2 \end{array}} (m+1) \mathcal {R}_{m+1} (\mathcal {R}^{-\eta })_\ell \right|&\lesssim \sum _{\begin{array}{c} \ell +m=N \\ 1\le m\le N-2 \end{array}} \frac{C^{m+1-\alpha }}{(m+1)^2}\left( \frac{C^{\ell -\alpha }}{\ell ^3} + \frac{C^{\ell -2}}{\ell ^2} \right) \\&\lesssim C^{N+1-2\alpha }\sum _{\begin{array}{c} \ell +m=N \\ 1\le m\le N-2 \end{array}} \frac{1}{m^2\ell ^3} + {C^{N-1-\alpha }}\sum _{m=1}^{[\frac{N}{2}]} \frac{1}{m^2}\frac{1}{(N-m)^2}\\&\lesssim \frac{C^{N-\alpha }}{N^2} \left( \frac{1}{C^{\alpha -1}}+ \frac{1}{C}\right) . \end{aligned} \end{aligned}$$

For the first term of the second line of (4.256), by (1.811), (1.801), (1.787)

1.818 $$\begin{aligned} \begin{aligned} \left| x_*^2 \sum _{\begin{array}{c} l+m=N\\ 1\le m\le N-2 \end{array}} (m+1) \mathcal {R}_{m+1} H_l \right|&\lesssim \sum _{\begin{array}{c} \ell +m=N \\ 1\le m\le N-2 \end{array}} \frac{C^{m+1-\alpha }}{(m+1)^2}\frac{C^{\ell -\alpha }}{(\ell +1)^3}\\&\lesssim \frac{C^{N+1-2\alpha }}{N^2}. \end{aligned} \end{aligned}$$

The other two terms of the second line can be estimated analogously. For the first term of the third line of (4.256), by (1.811), (1.812), (1.800), (1.786) we obtain

1.819 $$\begin{aligned}&\left| 2x_*^2(1-\varepsilon )\sum _{\begin{array}{c} \ell +m+n=N\\ 1\le n\le N-1 \end{array}} \mathcal {R}_\ell ( \mathcal {W}+ \varepsilon )_m ( \mathcal {R}- \mathcal {W})_n \right| \nonumber \\&\lesssim \left| ( \mathcal {R}(\mathcal {W}+\varepsilon ) )_{N-1} (\mathcal {R}_1-\mathcal {W}_1) \right| + \left| ( \mathcal {R}(\mathcal {W}+\varepsilon ) )_{1} (\mathcal {R}_{N-1}-\mathcal {W}_{N-1}) \right| \nonumber \\&\quad + \left| \sum _{n=2}^{N-2}( \mathcal {R}(\mathcal {W}+\varepsilon ) )_{N-n} (\mathcal {R}-\mathcal {W})_{n} \right| \nonumber \\&\lesssim \frac{C^{N-1-\alpha }}{(N-1)^3} + \frac{C^{N-1-\alpha }}{(N-1)^3}+ \sum _{n=2}^{N-2}\frac{C^{N-n-\alpha }}{(N-n)^3}\frac{C^{n-\alpha }}{n^3}\nonumber \\&\lesssim \frac{C^{N-1-\alpha }}{N^3} + \frac{C^{N-2\alpha }}{N^3}. \end{aligned}$$

The bound on the remaining term in the third line of (4.256) is entirely analogous. Collecting all the bounds (1.815), (1.816), (1.817), (1.818), (1.819), we conclude

1.820 $$\begin{aligned} |\mathcal {S}_N|\lesssim \frac{C^{N-\alpha }}{N^2} \left[ \frac{1}{C^{\alpha -1}}+ \frac{1}{CN} +\frac{1}{C^\alpha N}+ \frac{1}{C}\right] , \end{aligned}$$

which leads to (1.813) since $1<\alpha <2$ and $C>1$.

To prove (1.814), we first recall (4.258). Two first two terms on the right-hand side of (4.258) have the same structure as the first two terms in (4.255) and hence, by using the same uniform bound (1.796), they can be bounded analogously to (1.815) and (1.816). It remains to estimate $\tilde{\mathcal {V}}_N$ given in (4.259). The first, second and sixth lines of (4.259) have the same formal structure as the first, second and third lines of (4.256) and hence we obtain the same bounds as in (1.817), (1.818), and (1.819) by using (1.812) in place of (1.811) when necessary. We focus on the third, fourth and fifth lines of (4.259). For the first term of the third line, by using (1.805)

1.821 $$\begin{aligned} \begin{aligned} \left| \sum _{\begin{array}{c} \ell +m=N \\ 1\le m\le N \end{array}} (\mathcal {R}^{-\eta })_\ell (-1)^m \right|&\lesssim \mathcal {R}_0^{-\eta } + \left| (\mathcal {R}^{-\eta })_1\right| + \sum _{\ell =2}^{N-1} \left| (\mathcal {R}^{-\eta })_\ell \right| \\&\lesssim 1 + \hat{C} \sum _{\ell =2}^{N-1} \left( \frac{C^{\ell -\alpha }}{\ell ^3} + \frac{C^{\ell -2}}{\ell ^2} \right) \\&\lesssim \frac{C^{N-1-\alpha }}{N^3} + \frac{C^{N-3}}{N^2} \end{aligned} \end{aligned}$$

where we have used $N^3\lesssim C^{N-1-\alpha }$ for all $N\ge 3$ and (1.790) with $q=2,3$ and $C\ge 2$. For the second term of the third line, we isolate $m=N$, $m=N-1$ cases, $\ell =0, 1$ cases, further $\ell =N-m$ and $\ell =N-m-1$, and use (1.796), (1.805), (1.812)

1.822 $$\begin{aligned} \begin{aligned}&\left| 3\sum _{\begin{array}{c} \ell +m+n=N \\ 1\le m\le N \end{array}} \mathcal {W}_n (\mathcal {R}^{-\eta })_\ell (-1)^m \right| \lesssim 1+ \varepsilon + \sum _{m=1}^{N-2} \sum _{\ell =0}^{N-m} \left| \mathcal {W}_{N-m-\ell } (\mathcal {R}^{-\eta })_\ell \right| \\&\lesssim 1+ \sum _{m=1}^{N-2} |\mathcal {W}_{N-m}| + \sum _{m=1}^{N-2} |\mathcal {W}_{N-m-1}| + \hat{C} \sum _{m=1}^{N-2} \sum _{\ell =2}^{N-m} \left| \mathcal {W}_{N-m-\ell } \right| \left( \frac{C^{\ell -\alpha }}{\ell ^3}+\frac{C^{\ell -2}}{\ell ^2}\right) \\&\lesssim 1+ \sum _{m=1}^{N-2} \frac{C^{N-m-\alpha }}{(N-m)^3} + \sum _{m=1}^{N-3} \frac{C^{N-m-1-\alpha }}{(N-m-1)^3} \\&\quad + \sum _{m=1}^{N-2}\left[ \frac{C^{N-m-\alpha }}{(N-m)^3} + \frac{C^{N-m-2}}{(N-m)^2}\right] + \sum _{m=1}^{N-2}\left[ \frac{C^{N-m-1-\alpha }}{(N-m-1)^3} + \frac{C^{N-m-3}}{(N-m-1)^2}\right] \\&\quad + \sum _{m=1}^{N-2} \sum _{\ell =2}^{N-m-2} \frac{C^{N-m-\ell -\alpha }}{(N-m-\ell )^3} \left( \frac{C^{\ell -\alpha }}{\ell ^3}+\frac{C^{\ell -2}}{\ell ^2}\right) . \end{aligned} \end{aligned}$$

To bound the summations appearing in the third and the fourth line of (1.822), we apply (1.790) six times with $\ell = N-m$ and additionally we use $1\lesssim \frac{C^{N-1-\alpha }}{N^3}$ to bound them by

1.823 $$\begin{aligned} S_{1L} \lesssim \frac{C^{N-1-\alpha }}{N^3} + \frac{C^{N-3}}{N^2}. \end{aligned}$$

To bound the last line of (1.822) we use (1.787) and (1.790) to bound it by

1.824 $$\begin{aligned}&\sum _{m=1}^{N-2} C^{N-m-\alpha } \sum _{\ell =2}^{N-m-2} \frac{1}{(N-m-\ell )^3} \frac{1}{\ell ^3}+\sum _{m=1}^{N-2} C^{N-m-2-\alpha } \sum _{\ell =2}^{N-m-2} \frac{1}{(N-m-\ell )^3} \frac{1}{\ell ^2}\nonumber \\&\lesssim \sum _{m=1}^{N-2} \frac{ C^{N-m-\alpha }}{(N-m)^3} + \sum _{m=1}^{N-2} \frac{ C^{N-m-2-\alpha }}{ (N-m)^2 }\lesssim \frac{C^{N-1-\alpha }}{N^3} + \frac{ C^{N-3-\alpha }}{N^2}. \end{aligned}$$

For the fourth line of (4.259) we only present the details for the first term as the other two terms are estimated analogously. We first isolate $m=N$ and $m=N-1$ and then use (1.801), (1.790), and (1.786) to obtain

1.825 $$\begin{aligned} \begin{aligned} \left| x_*^2 \sum _{\begin{array}{c} \ell +m=N \\ 1\le m\le N \end{array}} H_\ell (-1)^m \right|&\lesssim 1 + \sum _{\ell =2}^{N-1} |H_\ell |\lesssim 1+ \sum _{\ell =2}^{N-1} \frac{C^{\ell -\alpha }}{\ell ^3} \lesssim \frac{C^{N-1-\alpha }}{N^3}. \end{aligned} \end{aligned}$$

For the fifth line of (4.259) we only present the detail for the first two terms, as the estimate for the remaining two terms in the fifth line of the right-hand side of (4.259) is analogous and strictly easier. By (1.796), (1.812), (1.801) we have

1.826 $$\begin{aligned} \begin{aligned} \left| 3x_*^2 \sum _{\begin{array}{c} \ell +n=N\\ 1\le n\le N-1 \end{array}} \mathcal {W}_n H_\ell \right|&\lesssim |\mathcal {W}_{N-1} H_1| + |\mathcal {W}_1 H_{N-1} | + \sum _{\ell =2}^{N-2} |\mathcal {W}_{N-\ell } H_\ell | \\&\lesssim \frac{C^{N-1-\alpha }}{(N-1)^3} + \sum _{\ell =2}^{N-2} \frac{C^{N-\ell -\alpha }}{(N-\ell )^3}\frac{C^{\ell -\alpha }}{\ell ^3}\\&\lesssim \frac{C^{N-1-\alpha }}{(N-1)^3} + \frac{C^{N-2\alpha }}{N^3}. \end{aligned} \end{aligned}$$

The second term of the fifth line of (4.259) can be estimated in a similar way as in (1.822) by using (1.801) instead of (1.805):

1.827 $$\begin{aligned} \begin{aligned}&\left| 3x_*^2 \sum _{\begin{array}{c} \ell +m+n=N\\ 1\le m\le N \end{array}} \mathcal {W}_n H_\ell (-1)^m \right| \lesssim 1+ \sum _{m=1}^{N-2} \sum _{\ell =0}^{N-m}|\mathcal {W}_{N-m-\ell } H_\ell | \\&\lesssim 1+ \sum _{m=1}^{N-2} |\mathcal {W}_{N-m}| + \sum _{m=1}^{N-3} |\mathcal {W}_{N-m-1}|+ \sum _{m=1}^{N-2} \sum _{\ell =2}^{N-m}|\mathcal {W}_{N-m-\ell } H_\ell | \\&\lesssim 1+ \sum _{m=1}^{N-2}\frac{C^{N-m-\alpha }}{(N-m)^3}+ \sum _{m=1}^{N-3}\frac{C^{N-m-1-\alpha }}{(N-m-1)^3} + \sum _{m=1}^{N-2} \left[ \frac{C^{N-m-\alpha }}{(N-m)^3}+ \frac{C^{N-m-1-\alpha }}{(N-m-1)^3}\right] \\&\quad + \sum _{m=1}^{N-2} \sum _{\ell =2}^{N-m-2}\frac{C^{N-m-\ell -\alpha }}{(N-m-\ell )^3 } \frac{C^{\ell -\alpha }}{\ell ^3} \\&\lesssim \frac{C^{N-1-\alpha }}{N^3} + \frac{C^{N-1-2\alpha }}{N^3} \end{aligned} \end{aligned}$$

where we have used (1.790) and (1.786) as before. Combining all the estimates, we deduce the desired bound (1.814). $\square$

### Lemma A.9

Let $x_*\in [x_{\text {crit}}+\kappa ,x_{\text {max}}]$ and $\alpha \in (1,2)$. Consider $(\mathcal {R}_0,\mathcal {W}_0)$, $(\mathcal {R}_1,\mathcal {W}_1)$ constructed in Lemma 4.1 and Lemma 4.6, and let $(\mathcal {R}_N,\mathcal {W}_N)$ be given recursively by (4.264) and (4.265). There exist a constant $C>1$ and $\varepsilon _0>0$ such that for all $0<\varepsilon < \varepsilon _0$ and all $x_*\in (x_{\text {min}},x_{\text {max}})$

1.828 $$\begin{aligned} |\mathcal {R}_N|\le \frac{C^{N-\alpha }}{N^3}, \end{aligned}$$

1.829 $$\begin{aligned} |\mathcal {W}_N|\le \frac{C^{N-\alpha }}{N^3}, \end{aligned}$$

for all $$N\ge 2$$.

### Proof

The proof is based on induction on *N*. When $N=2$, it is clear that there exists $C_0=C_0(\alpha ) >1$ such that for any $C> C_0$ the bound

1.830 $$\begin{aligned} |\mathcal {R}_2|, \ |\mathcal {W}_2| \le \frac{C^{2-\alpha }}{2^3} \end{aligned}$$

holds true for any $x_*\in [x_{\text {crit}}+\kappa ,x_{\text {max}}]$. Fix an $N\ge 3$ and suppose the claim is true for all $2\le m\le N-1$. Then (1.811) and (1.812) are satisfied and therefore by Lemma A.8 we conclude that (1.813) and (1.814) hold for all $C>C_*$. Together with (4.266) and (4.267), those bounds lead to

$$\begin{aligned} |\mathcal {R}_N|&\le \beta _0\beta \left( 1+\frac{\varepsilon }{N} \right) \left[ \frac{1}{C^{\alpha -1}} +\frac{1}{CN} +\varepsilon \right] \frac{C^{N-\alpha }}{N^3} \\&\le \beta _0\beta \left( 1+\frac{\varepsilon }{3} \right) \left[ \frac{1}{C^{\alpha -1}} +\frac{1}{3C} +\varepsilon \right] \frac{C^{N-\alpha }}{N^3} {=}{:} c_1 \frac{C^{N-\alpha }}{N^3}, \\ |\mathcal {W}_N|&\le \beta _0\beta \left( 1+\frac{1}{N} \right) \left[ \frac{1}{C^{\alpha -1}} +\frac{1}{CN} +\varepsilon \right] \frac{C^{N-\alpha }}{N^3}\\&\le \frac{4}{3} \beta _0\beta \left[ \frac{1}{C^{\alpha -1}} +\frac{1}{3C} +\varepsilon \right] \frac{C^{N-\alpha }}{N^3} {=}{:} c_2 \frac{C^{N-\alpha }}{N^3}. \end{aligned}$$

It is now clear that since $\alpha >1$ we can choose $C>C_*,C_0$ sufficiently large and $\varepsilon <\varepsilon _0$ with $\varepsilon _0>0$ sufficiently small so that $c_1,c_2<1$ and hence (1.828) and (1.829) hold true. $\square$

## Null-Geodesic Flow in the RLP-Spacetime

We shall carry out the analysis of nonradial null-geodesics (NNG) in the comoving coordinates $(\tau ,R,\theta ,\phi )$. Partial analysis of simple NNG-s in Schwarzschild coordinates was already carried out in [28] and a further analysis of non-spacelike geodesics was done in [19]. A related problem for the selfsimilar dust collapsing clouds was studied in detail in [29].

It is convenient to introduce

2.831 $$\begin{aligned} z{:}{=} \frac{\sqrt{\varepsilon }\tau }{R} \left( = -\frac{1}{y} \right) , \quad s{:}{=}- \log R, \end{aligned}$$

so that

2.832 $$\begin{aligned} z_{\mathcal {N}} {:}{=} - \frac{1}{y_{\mathcal {N}}}, \ \ z_1{:}{=}-\frac{1}{y_1}, \end{aligned}$$

correspond to the boundary of the backward light cone $\mathcal {N}$ ($y_{\mathcal {N}}= Y_{\mathcal {N}}^{-1-\eta }$) and the “first" outgoing null-geodesic $\mathcal {B}_1$ ($y_1=-|Y_1|^{-1-\eta }$) respectively. Then $\partial _z \tau = \frac{1}{\sqrt{\varepsilon }}e^{-s}$ and $\partial _s \tau = - \frac{1}{\sqrt{\varepsilon }} z e^{-s}$ and hence $d\tau = \frac{e^{-s}}{\sqrt{\varepsilon }} (dz - z ds)$ and $dR = - e^{-s} ds$. It is straightforward to check that the homothetic Killing vector field $\xi = {\tau }{\partial _\tau } + R\partial _R$ takes the form $\xi = -\partial _s$ and the metric *g* in these coordinates reads

2.833 $$\begin{aligned} \begin{aligned} g&= e^{-2s} \left[ - \frac{e^{2\mu }}{\varepsilon } dz^2 + 2 \frac{e^{2\mu } }{\varepsilon } zdzds + \left( e^{2\lambda } - \frac{e^{2\mu }}{\varepsilon } z^2 \right) ds^2 + {\varvec{\chi }}^2 d\phi ^2 \right] . \end{aligned} \end{aligned}$$

We note that the metric is not regular at $z=0$. This corresponds to a harmless coordinate singularity which can be easily avoided by introducing a suitable change of variables, see Section 7. We shall nevertheless work with (2.833) to avoid further notational complications and formally limit our analysis to the regions of $\mathcal {M}_{\text {RLP},\varepsilon }$ satisfying $\{z<0\}$ and $\{z>0\}$.

Let $\gamma ^\kappa$, $\kappa =z,s,\theta ,\phi$ be a null-geodesic and we denote the associated tangent vector by $V^\kappa {:}{=}\dot{\gamma }^\kappa$, $\kappa =z,s,\theta ,\phi$. We let $\ell$ denote the affine parameter. Due to spherical symmetry, we have

2.834 $$\begin{aligned} \gamma ^\theta (\ell ) = \theta (\ell )=\frac{\pi }{2}. \end{aligned}$$

The remaining geodesic equations read

2.835 $$\begin{aligned} \frac{dV_\kappa }{d\ell } = \frac{1}{2} (\partial _\kappa g_{\alpha \beta }) V^\alpha V^\beta , \ \ \kappa =z,s,\phi , \end{aligned}$$

where $$V_\kappa = g_{\kappa \nu } V^\nu $$.

### Lemma A.1

(Geodesic flow) Let $V^\kappa$ be the tangent to a null-geodesic as above. Then, there exist constants $C,L\in \mathbb R$ such that

2.836 $$\begin{aligned} - \frac{e^{2\mu }}{\varepsilon } (V^z)^2 + 2 \frac{e^{2\mu } }{\varepsilon } zV^z V^s + \left( e^{2\lambda } - \frac{e^{2\mu }}{\varepsilon } z^2 \right) (V^s)^2 + {\varvec{\chi }}^2 (V^\phi )^2&=0, \end{aligned}$$

2.837 $$\begin{aligned} V_\phi&=L, \end{aligned}$$

2.838 $$\begin{aligned} V_s&= C. \end{aligned}$$

Moreover, the $V^s$-component satisfies the quadratic equation

2.839 $$\begin{aligned} e^{\lambda +\mu } \left( z^2 - \varepsilon e^{2\lambda - 2\mu } \right) \left( \frac{V^s}{e^{2s}} \right) ^2 + 2 C\varepsilon e^{\lambda -\mu }\left( \frac{V^s}{e^{2s}} \right) - e^{-\lambda -\mu }\varepsilon C^2 +e^{-\lambda +\mu } z^2\frac{L^2}{{\varvec{\chi }}^2}=0. \end{aligned}$$

### Proof

Equation (2.836) is just the statement that the 4-vector $V^\alpha$ is null. We now let $\alpha =\phi$ in (2.835). Since $g_{\alpha \beta }$-terms do not depend on $\phi$, from (2.835) we immediately see that $\frac{dV_\phi }{d\ell }=0$, which implies (2.837). Since $V_\phi = g_{\phi \phi } V^{\phi }$, we also obtain

2.840 $$\begin{aligned} V^\phi = \frac{L}{{\varvec{\chi }}^2} e^{2s}. \end{aligned}$$

Next let $\kappa =s$ in (2.835). Since $\partial _s g = - 2 g$ (cf. (2.833)) we deduce that $\frac{dV_s}{d\ell }=0$ from (2.835) and (2.838) follows.

Since $V_s= g_{ss}V^s + g_{sz}V^z$, we obtain

2.841 $$\begin{aligned} \left( e^{2\lambda } - \frac{e^{2\mu }}{\varepsilon } z^2 \right) \frac{V^s}{e^{2s}} +\frac{e^{2\mu }}{\varepsilon } z \frac{V^z}{e^{2s}} =C, \end{aligned}$$

which in turn gives

2.842 $$\begin{aligned} z\frac{V^z}{e^{2s}} = \frac{\varepsilon C }{ e^{2\mu }}+ \left( z^2 - \varepsilon e^{2\lambda - 2\mu } \right) \frac{V^s}{e^{2s}}. \end{aligned}$$

Using (2.840), we may rewrite (2.836) as

2.843 $$\begin{aligned} - \frac{e^{2\mu }}{\varepsilon } \left( \frac{V^z}{e^{2s}}\right) ^2 + 2 \frac{e^{2\mu } }{\varepsilon } z\left( \frac{V^z}{e^{2s}} \right) \left( \frac{V^s}{e^{2s}}\right) + \left( e^{2\lambda } - \frac{e^{2\mu }}{\varepsilon } z^2 \right) \left( \frac{V^s}{e^{2s}} \right) ^2 + \frac{L^2}{ {\varvec{\chi }}^2} =0 . \end{aligned}$$

Recall that we work in a patch where $z\ne 0$. Plugging (2.842) into (2.843) to replace $V^z$, we get

$$\begin{aligned} \begin{aligned}&- \frac{e^{2\mu }}{\varepsilon } \left( \frac{\varepsilon C }{z e^{2\mu }}-+ \frac{1}{z} \left( z^2 - \varepsilon e^{2\lambda - 2\mu } \right) \frac{V^s}{e^{2s}} \right) ^2\\&\quad + 2 \frac{e^{2\mu } }{\varepsilon } z\left( \frac{\varepsilon C }{z e^{2\mu }} + \frac{1}{z} \left( z^2 - \varepsilon e^{2\lambda - 2\mu } \right) \frac{V^s}{e^{2s}} \right) \left( \frac{V^s}{e^{2s}}\right) \\&\quad - \frac{e^{2\mu }}{\varepsilon } \left( z^2 - \varepsilon e^{2\lambda - 2\mu } \right) \left( \frac{V^s}{e^{2s}} \right) ^2 + \frac{L^2}{ {\varvec{\chi }}^2} =0 \end{aligned} \end{aligned}$$

which is easily simplified to give (2.839). $\square$

### Remark A.2

Relations (2.837)–(2.838) are the Hamiltonian constraints associated with the geodesic flow. Equation (2.837) expresses the conservation of angular momentum associated with the Killing vector field $\partial _\phi$, while (2.838) is the conservation law generated by the homothetic Killing vector field $-\partial _s = \tau \partial _\tau +R\partial _R$.

### Lemma A.3

Assume that there exists a simple nonradial null-geodesic, i.e. a null-geodesic such that $L\ne 0$ and $z(\ell )=\bar{z}\equiv \text {const}$ for all $\ell \in \mathbb R$. Then $\bar{z}$ is a critical point of the function

2.844 $$\begin{aligned} \mathcal {H}(z) : = \left( e^{2\lambda (z) } - \frac{e^{2\mu (z)}}{\varepsilon } z^2 \right) {\varvec{\chi }}(z)^{-2}. \end{aligned}$$

### Proof

By our assumption, for any simple geodesic we have $V^z=0$. By letting $\kappa =z$ in (2.835), we then obtain the formula

2.845 $$\begin{aligned} \frac{d}{d\ell } \left( \frac{e^{2\mu }}{\varepsilon }e^{-2s}V^s\right) = \frac{e^{-2s}}{2} \partial _z\left( e^{2\lambda } - \frac{e^{2\mu }}{\varepsilon } z^2 \right) (V^s)^2 + \frac{e^{-2s}}{2} \partial _z({\varvec{\chi }}^2)(V^\phi )^2. \end{aligned}$$

Since $V^z=0$ in (2.841), we conclude that $V^se^{-2s}$ is $\ell$-independent and therefore the left-hand side of (2.845) vanishes. Letting $V^z=0$ in (2.836) we have $(V^\phi )^2= -\left( e^{2\lambda }- \frac{e^{2\mu }}{\varepsilon } z^2 \right) \frac{(V^s)^2}{{\varvec{\chi }}^2}$. We plug this back into (2.845) and the claim follows. $\square$

### Lemma A.4

(Monotonicity of $\mathcal {H}$) The function $\mathcal {H}$ defined by (2.844) is strictly monotone on $(-\infty ,0)$.

### Proof

Recalling the notation $x=\tilde{r}(y)$, ${\varvec{\chi }}(y)=\frac{x}{y}$, and $z=-\frac{1}{y}$, we see that $\mathcal {H}$, written as a function of *y* can be written in the form

2.846 $$\begin{aligned} \mathcal {H}(y) = x^{-2}\left( e^{2\lambda }y^2 - \frac{1}{\varepsilon }e^{2\mu }\right) . \end{aligned}$$

Therefore

2.847 $$\begin{aligned} \mathcal {H}'(y) =&-2 x^{-2}\frac{x'}{x} \left( e^{2\lambda }y^2 - \frac{1}{\varepsilon }e^{2\mu }\right) + x^{-2} \left( 2e^{2\lambda }y^2\left( \lambda '+\frac{1}{y}\right) -\frac{2}{\varepsilon }e^{2\mu }\mu '\right) \nonumber \\ =&-2 x^{-2}\frac{\textbf{w}+\varepsilon }{(1+\varepsilon )y} \left( e^{2\lambda }y^2 - \frac{1}{\varepsilon }e^{2\mu }\right) + 2x^{-2} \left( e^{2\lambda }y^2\left( -\frac{1}{1+\varepsilon }\left( \frac{ \Sigma '}{ \Sigma }+\frac{2}{y}\right) \right. \right. \nonumber \\&\quad \left. \left. - 2\left( \frac{x'}{x}-\frac{1}{y}\right) +\frac{1}{y}\right) +\frac{1}{\varepsilon }e^{2\mu } \frac{\varepsilon }{1+\varepsilon } \frac{ \Sigma '}{ \Sigma }\right) \nonumber \\ =&-2 x^{-2}\frac{\textbf{w}+\varepsilon }{(1+\varepsilon )y} \left( e^{2\lambda }y^2 - \frac{1}{\varepsilon }e^{2\mu }\right) + \frac{2x^{-2}}{1+\varepsilon } \left( e^{2\lambda }y^2\left( -\frac{ \Sigma '}{ \Sigma }+\frac{1+3\varepsilon }{y} - 2\frac{\textbf{w}+\varepsilon }{y} \right) \right. \nonumber \\&\quad \left. +e^{2\mu } \frac{ \Sigma '}{ \Sigma } \right) \nonumber \\ =&-2 x^{-2}\frac{\textbf{w}+\varepsilon }{(1+\varepsilon )y} \left( e^{2\lambda }y^2 - \frac{1}{\varepsilon }e^{2\mu }\right) + \frac{2x^{-2}}{1+\varepsilon } \left( \frac{ \Sigma '}{ \Sigma }\left( e^{2\mu }-e^{2\lambda }y^2\right) +e^{2\lambda }y\left( 1+\varepsilon - 2\textbf{w}\right) \right) , \end{aligned}$$

where we have used the relation $\frac{x'}{x}=\frac{\textbf{w}+\varepsilon }{(1+\varepsilon )y}$ (see (2.33)), formula (3.97) for $\lambda '$, and (3.99) to substitute for $\mu '$. We now use (3.117) and part (c) of Lemma 3.3 to obtain the formula

2.848 $$\begin{aligned} \frac{ \Sigma '}{(1+\varepsilon ) \Sigma }\left( e^{2\mu }-e^{2\lambda }y^2\right) = -2ye^{2\lambda }K = -\frac{2(\textbf{d}-\textbf{w})}{(1+\varepsilon )}e^{2\lambda }y. \end{aligned}$$

Using this in (2.847) we obtain

2.849 $$\begin{aligned} \mathcal {H}'(y)&=-2 x^{-2}\frac{\textbf{w}+\varepsilon }{(1+\varepsilon )y} \left( e^{2\lambda }y^2 - \frac{1}{\varepsilon }e^{2\mu }\right) + \frac{2x^{-2}}{1+\varepsilon } e^{2\lambda }y\left( 1+\varepsilon - 2\textbf{d}\right) \nonumber \\&=\frac{2 x^{-2}}{(1+\varepsilon )y} e^{2\lambda } \left( \frac{1}{\varepsilon }(\textbf{w}+\varepsilon )e^{2\mu -2\lambda } + y^2 \left( 1-\textbf{w}-2\textbf{d}\right) \right) . \end{aligned}$$

We now recall the formula

2.850 $$\begin{aligned} e^{2\mu -2\lambda } = \frac{y^2}{x^2(\textbf{w}+\varepsilon )^2}\left( \textbf{d}^{-\eta }+\varepsilon x^2(\textbf{w}-1)^2 - 4\varepsilon \textbf{d}\textbf{w}x^2\right) , \end{aligned}$$

see (3.121). We plug it back into (2.849) to conclude that

2.851 $$\begin{aligned} \mathcal {H}'(y)&=\frac{2 x^{-2}y}{(1+\varepsilon )} e^{2\lambda } \left( \frac{1}{\varepsilon }\frac{\textbf{d}^{-\eta }}{x^2(\textbf{w}+\varepsilon )} + \frac{(\textbf{w}-1)^2}{\textbf{w}+\varepsilon }-4\frac{\textbf{d}\textbf{w}}{\textbf{w}+\varepsilon }+1-\textbf{w}-2\textbf{d}\right) \nonumber \\&= \frac{2 x^{-2}y}{(1+\varepsilon )} e^{2\lambda } \left( \frac{\textbf{d}^{-\eta }}{x^2}\left[ \frac{1}{\varepsilon }\frac{1}{\textbf{w}+\varepsilon } - \textbf{d}^{1+\eta }x^2\left( 2+\frac{4\textbf{w}}{\textbf{w}+\varepsilon }\right) \right] + \frac{(\textbf{w}-1)^2}{\textbf{w}+\varepsilon } + 1-\textbf{w}\right) . \end{aligned}$$

Recall that $\frac{1}{6}\le \textbf{w}\le 1$ for all $y\in (0,\infty )$. Moreover, by (6.469) and the inequality $\textbf{w}(y)=W(x)<1$ for all $y,x\ge 0$ we know that that the function $x\mapsto D^{1+\eta }x^2 W(x)$ is increasing. It follows from (6.468) that there exists an $\varepsilon$-independent constant *C* such that $D^{1+\eta }x^2\le C$ for all $\varepsilon \in (0,\varepsilon _0]$, with $\varepsilon _0$ sufficiently small. Since $\frac{1}{\textbf{w}+\varepsilon }\ge \frac{1}{1+\varepsilon }$, $2+\frac{4\textbf{w}}{\textbf{w}+\varepsilon } \le 2+\frac{4}{\frac{1}{3}+\varepsilon }$, and $\frac{(\textbf{w}-1)^2}{\textbf{w}+\varepsilon } + 1-\textbf{w}>0$, we conclude from (2.851) that for $\varepsilon >0$ sufficiently small that $\mathcal {H}'$ is strictly positive for $y\in (0,\infty )$. $\square$

### Lemma A.5

($L=0$: radial null-geodesics) Let $V^\kappa$ as above. If $L=0$ the flow takes the form

2.852 $$\begin{aligned} \frac{ds}{dz}=\frac{V^s}{V^z} =\frac{1}{ z \pm \sqrt{\varepsilon } e^{\lambda -\mu }}, \end{aligned}$$

while $V^s$, $V^z$ are then recovered using (2.842). The behaviour of the solutions is described by Lemma 8.8.

### Proof

Equation (2.852) is a simple consequence of (2.843). Equation (2.852) is equivalent to the radial null geodesic equation (8.641) expressed in $(Y,\log R)$-variables. $\square$

### Lemma A.6

($L\ne 0$: nonradial null-geodesics) Let $V^\kappa$ as above and $L\ne 0$. Then any such geodesic that emanates from the union of the exterior and the interior region exists globally in its affine time and does not converge to the scaling origin.

### Proof

*Step 1. We first consider the case where* $C=0$. Then (2.842) gives the relation

2.853 $$\begin{aligned} \frac{ds}{dz} =\frac{V^s}{V^z} = - \frac{z}{\varepsilon e^{2\lambda -2\mu } - z^2}. \end{aligned}$$

In order for $V^s$ to satisfy (2.839) we must have $\varepsilon e^{2\lambda -2\mu } - z^2 > 0$ since $\frac{L^2}{{\varvec{\chi }}^2}>0$. In particular, the null geodesics are confined to the exterior region $z_{\mathcal {N}}< z< z_1$ where $z_{\mathcal {N}} {:}{=} - |Y_{\mathcal {N}}|^{1+\eta }<0$ and $z_1{:}{=} |Y_1|^{1+\eta }>0$. Let $s_0\in \mathbb R$ and $z_0 \in ( z_{\mathcal {N}}, z_1)$ be given initial point in the exterior region. Then by integrating (2.853), we obtain

2.854 $$\begin{aligned} s=s_0 - \int _{z_0}^{z(s)} \frac{z}{\varepsilon e^{2\lambda -2\mu } - z^2} dz . \end{aligned}$$

We recall that $\varepsilon e^{2\lambda -2\mu } - z^2|_{z= z_{\mathcal {N}}, z_1} =0$ and $\varepsilon e^{2\lambda -2\mu } - z^2 > 0$ for $z_{\mathcal {N}}< z< z_1$. Since $s= -\log R$, we deduce that $R\rightarrow \infty$ ($s \rightarrow -\infty$) as $z(s)\rightarrow z_{\mathcal {N}}$ or $z(s)\rightarrow z_1$. Therefore a null geodesic in this case asymptotes to the backward light cone $z=z_{\mathcal {N}}$ in the past and to the Cauchy horizon $z=z_1$ in the future. In particular, there are no null geodesics emanating from or going into the origin.

*Step 2. We now consider the general case* $C\ne 0$. Assume without loss of generality $C>0$. In order for the solution of (2.839) to exist the discriminant must be nonnegative. This amounts to requiring

2.855 $$\begin{aligned} a(z){:}{=} \frac{e^{2\mu } (z^2 - \varepsilon e^{2\lambda - 2\mu })}{{\varvec{\chi }}^2 }\le \frac{\varepsilon C^2}{L^2}. \end{aligned}$$

Note that $\lim _{z\rightarrow -\infty } a(z) = +\infty$, which follows from $z= -\frac{1}{y}$, (2.74), and $e^{\mu }|_{y=0}>0$, while $\lim _{z\rightarrow z^{\text {ms}}} a(z)= -\infty$ by Proposition 7.16. Moreover, we also know $a(z_{\mathcal {N}})=0$, $a(z_1)=0$ and $a(z)<0$ for $z_{\mathcal {N}}< z< z_1$ from the analysis of radial null geodesics. Therefore, for given $\frac{\varepsilon C^2}{L^2}>0$, there exists $\bar{z}<z_{\mathcal {N}}$ such that $a(z) > \frac{\varepsilon C^2}{L^2}$ for all $z\in (-\infty , \bar{z})$ and hence, the non-radial null geodesics cannot enter the part of the interior region $z<\bar{z}$.

Under the assumption (2.855), the solution to (2.839) is given by

2.856 $$\begin{aligned} \frac{V^s}{e^{2s}} = C\sqrt{\varepsilon }\frac{- \sqrt{\varepsilon }e^{\lambda -\mu } \pm |z| \sqrt{ 1 - \frac{e^{2\mu }(z^2- \varepsilon e^{2\lambda -2\mu })}{{\varvec{\chi }}^2} \frac{L^2}{\varepsilon C^2} } }{e^{\lambda +\mu }\left( z^2 - \varepsilon e^{2\lambda -2\mu } \right) }. \end{aligned}$$

From (2.842) we also have (recall $z\ne 0$)

2.857 $$\begin{aligned} z\frac{V^z}{e^{2s}} = \pm \frac{C\sqrt{\varepsilon }|z|}{e^{\lambda +\mu }} \sqrt{ 1 - \frac{e^{2\mu }(z^2- \varepsilon e^{2\lambda -2\mu })}{{\varvec{\chi }}^2} \frac{L^2}{\varepsilon C^2} }. \end{aligned}$$

Let

2.858 $$\begin{aligned} Q(z){:}{=} \sqrt{ 1 - \frac{e^{2\mu }(z^2- \varepsilon e^{2\lambda -2\mu })}{{\varvec{\chi }}^2} \frac{L^2}{\varepsilon C^2} }. \end{aligned}$$

We then conclude that

2.859 $$\begin{aligned} \frac{ds}{dz} = \frac{V^s}{V^z}= \frac{z\left[ - \sqrt{\varepsilon }e^{\lambda -\mu } \pm |z| Q(z) \right] }{\pm |z| Q(z)( z^2 - \varepsilon e^{2\lambda -2\mu })}. \end{aligned}$$

*Step 3. No simple NNG-s*. By definition, we have $\frac{dz}{ds}=0$ for simple NNG-s. Therefore by (2.859) they must satisfy either $z^2-\varepsilon e^{2\lambda - 2\mu }=0$ or $Q(z)=0$. The first case corresponds to simple radial null geodesics, while zeros of $Q(z)=0$ possibly describe the simple non-radial geodesics. We know that $z^2 - \varepsilon e^{2\lambda -e\mu } <0$ for all $z_{\mathcal {N}}< z<z_1$ and thus, $Q >0$ for all $z_{\mathcal {N}}< z<z_1$. Therefore we deduce that there is no simple non-radial null geodesics in the exterior (i.e. in the causal past of $\mathcal {B}_1$). In the interior region however $\bar{z}$ cannot correspond to a geodesic, since otherwise by Lemma A.3 we would have $\mathcal {H}'(\bar{z})=0$, but this is impossible by Lemma A.4.

*Step 4. NNG-s cannot emanate from or go towards the scaling origin* $\mathcal {O}$.

*Step 4.1. First let*$z_1>z_0>0$ *and* $s_0\in \mathbb R$ *be given in the exterior region above* $\tau =0$ ($z>0$). Then the null geodesics satisfy

2.860 $$\begin{aligned} \frac{ds}{dz} = \frac{ - \sqrt{\varepsilon }e^{\lambda -\mu } \pm z Q(z)}{\pm Q(z)( z^2 - \varepsilon e^{2\lambda -2\mu })}. \end{aligned}$$

We start with $(-)$ part. In this case, the right-hand side of (2.860) is negative and hence the null geodesics are outgoing. We claim that outgoing null geodesics $(-)$ starting from $z_0>0$ and $s_0\in \mathbb R$ meet $z=0$ (namely $\tau =0$) for some positive $R>0$ in the past. Observe that there exist $-\infty<M_1< M_0<0$ such that $M_1<Q(z) ( z^2 - \varepsilon e^{2\lambda -2\mu }) < M_0$ for all $z\in [0,z_0]$. Integrating (2.860), we have

2.861 $$\begin{aligned} s(0) = s(z_0) + \int _{z_0}^0 \frac{ \sqrt{\varepsilon }e^{\lambda -\mu } + z Q(z)}{ Q(z)( z^2 - \varepsilon e^{2\lambda -2\mu })} dz . \end{aligned}$$

The integral in the right-hand side is finite and using $s=-\log R$, the claim follows. On the other hand, in the future direction, since $z^2-\varepsilon e^{2\lambda -2\mu } \rightarrow 0$ as $z\rightarrow z_1^-$, $s \rightarrow -\infty$ as $z\rightarrow z_1^-$ and thus it asymptotes to the Cauchy horizon $z=z_1$.

We move onto $(+)$ part, the ingoing case. As above, the null geodesics meet $\tau =0$ for some positive $R>0$ in the past as the corresponding integral stays finite. We claim that the null geodesics meets $\mathcal {B}_1$ at positive $R>0$ in the future. Integrating (2.860), we have

2.862 $$\begin{aligned} s = s(z_0) + \int _{z_0}^{z(s)} \frac{ - \sqrt{\varepsilon }e^{\lambda -\mu } + z Q(z)}{ Q(z)( z^2 - \varepsilon e^{2\lambda -2\mu })} dz \end{aligned}$$

for $z=z(s)< z_1$. To prove the claim, it suffices to show the integral is finite when $z(s)=z_1$. To this end, we rewrite the integrand as

$$\begin{aligned} \frac{ - \sqrt{\varepsilon }e^{\lambda -\mu } + z Q(z)}{ Q(z)( z^2 - \varepsilon e^{2\lambda -2\mu })}= & {} \frac{ - \sqrt{\varepsilon }e^{\lambda -\mu } + z + z (Q(z)-1)}{ Q(z)( z^2 - \varepsilon e^{2\lambda -2\mu })} \\= & {} \frac{1}{Q(z) (z+ \sqrt{\varepsilon }e^{\lambda -\mu })} + \frac{ z (Q(z)^2-1)}{ Q(z)( z^2 - \varepsilon e^{2\lambda -2\mu }) (Q(z) +1)} \\= & {} \frac{1}{Q(z) (z+ \sqrt{\varepsilon }e^{\lambda -\mu })} - \frac{z \frac{e^{2\mu } }{{\varvec{\chi }}^2} \frac{L^2}{\varepsilon C^2}}{Q(z) (Q(z)+1)} \end{aligned}$$

for $z_0<z<z_1$, where we have used (2.858) in the last line. It is now clear that both terms are finite and thus the integral is indeed bounded, thereby proving the claim.

*Step 4.2. Next we let* $z_{\mathcal {N}}<z_0<0$ *and* $s_0\in \mathbb R$ *be given in the exterior region below* $\tau =0$ $(z<0)$. The null geodesics satisfy

2.863 $$\begin{aligned} \frac{ds}{dz} = \frac{ - \sqrt{\varepsilon }e^{\lambda -\mu } \mp z Q(z)}{\mp Q(z)( z^2 - \varepsilon e^{2\lambda -2\mu })}. \end{aligned}$$

We start with $(+)$ part. In this case, we claim that the null geodesics meet $\tau =0$ for positive $R>0$ in the future and asymptotes to the past light cone $z_{\mathcal {N}}$ in the past. The geodesic in the future satisfies

2.864 $$\begin{aligned} s(0) = s(z_0) + \int _{z_0}^0 \frac{ - \sqrt{\varepsilon }e^{\lambda -\mu } + z Q(z)}{Q(z)( z^2 - \varepsilon e^{2\lambda -2\mu })} dz . \end{aligned}$$

Observe that the integrand is positive and uniformly bounded. Therefore, the claim for the future follows. Now in the past, the right-hand side of (2.863) is positive and the denominator goes to 0 as $z\rightarrow z_{\mathcal {N}}$. Therefore $s\rightarrow -\infty$ and $R\rightarrow \infty$ as $z\rightarrow z_{\mathcal {N}}$, and hence the null geodesics asymptote to $z=z_{\mathcal {N}}$.

We move onto $(-)$ part. Since the denominator is uniformly bounded for $z\in [z_0,0]$, the integral is finite and hence we deduce that the null geodesics meet $\tau =0$ for some positive $R>0$ in the future. On the other hand, the null geodesics in the past satisfy

2.865 $$\begin{aligned} s = s(z_0) + \int _{z_0}^{z(s)} \frac{ \sqrt{\varepsilon }e^{\lambda -\mu } + z Q(z)}{Q(z)( z^2 - \varepsilon e^{2\lambda -2\mu })} dz . \end{aligned}$$

We claim that they meet $z=z_{\mathcal {N}}$ for finite $R>0$. To this end, we rewrite the integrand as

$$\begin{aligned} \begin{aligned} \frac{ \sqrt{\varepsilon }e^{\lambda -\mu } + z Q(z)}{ Q(z)( z^2 - \varepsilon e^{2\lambda -2\mu })}&= \frac{ \sqrt{\varepsilon }e^{\lambda -\mu } + z + z (Q(z)-1)}{ Q(z)( z^2 - \varepsilon e^{2\lambda -2\mu })} = \frac{1}{Q(z) (z- \sqrt{\varepsilon }e^{\lambda -\mu })} - \frac{z \frac{e^{2\mu } }{{\varvec{\chi }}^2} \frac{L^2}{\varepsilon C^2}}{Q(z) (Q(z)+1)} \end{aligned} \end{aligned}$$

for $z_{\mathcal {N}}<z<z_0<0$, where we have used (2.858) in the last line. It is now clear that both terms are finite and thus the integral is bounded, thereby proving the claim.

*Step 5. NNG-s in the interior region.* Using the same strategy as above, one can show that nonradial geodesics ($L\ne 0$) starting in the interior region will asymptote to the past to the boundary of the backward light cone $\mathcal {N}$, and in the future they will asymptote to the Cauchy horizon $\mathcal {B}_1$ to the future. In between, they will go through a turning point, which corresponds to the point $(\bar{s}, \bar{z})$, where $\bar{z}<z_{\mathcal {N}}$ is the zero of *Q* discussed above. We omit the details. $\square$

## Einstein-Euler System in the Double-Null Gauge

### Proof of Lemma 2.13

#### Proof of Lemma 2.13

Note that

3.866 $$\begin{aligned} T_{pq} = \frac{1}{4} \Omega ^{4} T^{pq}, \ \ T_{pp} = \frac{1}{4} \Omega ^{4} T^{qq}, \ \ T_{qq} = \frac{1}{4} \Omega ^{4} T^{pp}. \end{aligned}$$

We use (2.89) to obtain $\gamma ^{AB}T_{AB} = 2 \varepsilon \rho r^2= \eta \Omega ^2 r^2 T^{pq}$. Equations (2.83)–(2.86) now follow directly from Section 5 of [11]. Formulas (2.89)-(2.90) follow directly from (1.4) and (2.80). The fluid evolution equations (2.87)–(2.88) are a consequence of the Bianchi identities $\nabla _\mu T^{\mu \nu }=0$, $\nu = p,q,2,3$. The Christoffel symbols associated with the purely angular degrees of freedom $\Gamma ^A_{BC}$, $A,B,C\in \{2,3\}$ may not vanish, but are not needed in the computations to follow, so we do not compute them. All the remaining non-vanishing Christoffel symbols are given by (Appendix A in [11])

3.867 $$\begin{aligned}&\Gamma ^p_{AB} = - g^{pq} r \partial _qr \gamma _{AB}, \ \ \Gamma ^q_{AB} = - g^{pq} r \partial _pr \gamma _{AB}, \end{aligned}$$

3.868 $$\begin{aligned}&\Gamma ^A_{Bq} = \Gamma ^A_{qB}= \frac{\partial _qr}{r} \delta ^A_B, \ \ \Gamma ^A_{Bp} = \Gamma ^A_{pB} = \frac{\partial _pr}{r} \delta ^A_B, \end{aligned}$$

3.869 $$\begin{aligned}&\Gamma ^p_{pp} = \partial _{p}\log (\Omega ^2), \ \ \Gamma ^q_{qq} = \partial _{q}\log (\Omega ^2). \end{aligned}$$

By (2.90) we note that

3.870 $$\begin{aligned} \gamma _{AB}T^{AB} = \varepsilon \rho r^{-2} \gamma _{AB}\gamma ^{AB} = 2\varepsilon \rho r^{-2}. \end{aligned}$$

We also observe that by (3.870) and (2.89)

3.871 $$\begin{aligned} \Omega ^{-2}\gamma _{AB}T^{AB} = 2\varepsilon \rho \Omega ^{-2} r^{-2} = \frac{2\varepsilon }{1-\varepsilon } r^{-2} T^{pq} = \eta r^{-2} T^{pq} , \end{aligned}$$

where we recall the notation $\eta = \frac{2\varepsilon }{1-\varepsilon }.$ To express the Bianchi identities in coordinates, we use the formula

3.872 $$\begin{aligned} \nabla _\delta T^{\alpha \beta } = \partial _\delta T^{\alpha \beta } + \Gamma ^\alpha _{\gamma \delta }T^{\gamma \beta } + \Gamma ^{\beta }_{\gamma \delta }T^{\alpha \gamma } \end{aligned}$$

and the formulas (3.867)–(3.869). We start the with the *p*-equation and obtain from (1.2)

3.873 $$\begin{aligned} 0 = \nabla _\mu T^{\mu p} = \nabla _pT^{pp} + \nabla _qT^{qp} + \nabla _AT^{Ap}. \end{aligned}$$

By (3.872) and (3.867)–(3.869) we have

3.874 $$\begin{aligned} \nabla _pT^{pp}&= \partial _{p}T^{pp} + 2\Gamma ^p_{pp}T^{pp} = \left( \partial _p+ 4\frac{\partial _{p}\Omega }{\Omega }\right) T^{pp}, \end{aligned}$$

3.875 $$\begin{aligned} \nabla _qT^{qp}&= \partial _qT^{pq} + \Gamma ^{q}_{qq}T^{pq} = \left( \partial _q+ 2\frac{\partial _{q}\Omega }{\Omega }\right) T^{pq} , \end{aligned}$$

3.876 $$\begin{aligned} \nabla _A T^{Ap}&= \partial _A T^{Ap} + \Gamma ^A_{\gamma A}T^{\gamma p} + \Gamma ^p_{\gamma A}T^{A\gamma } \nonumber \\&= \Gamma ^A_{pA}T^{pp} + \Gamma ^A_{qA}T^{qp} +\Gamma ^p_{AB}T^{AB} \nonumber \\&= 2\frac{\partial _{p}r}{r} T^{pp}+2\frac{\partial _{q}r}{r} T^{pq} -g^{pq} r \partial _{q}r \gamma _{AB}T^{AB} \nonumber \\&= 2\frac{\partial _{p}r}{r} T^{pp}+2\frac{\partial _{q}r}{r} T^{pq} + 2\Omega ^{-2}r \partial _{q}r \gamma _{AB}T^{AB} \nonumber \\&= 2\frac{\partial _{p}r}{r} T^{pp}+(2+2\eta )\frac{\partial _{q}r}{r} T^{pq} , \end{aligned}$$

where we have used (2.80) in the next-to-last line and (3.871) in the last. Similarly, we compute the $q$-equation of the Bianchi identities (1.2).

3.877 $$\begin{aligned} 0 = \nabla _\mu T^{\mu q} = \nabla _pT^{pq} + \nabla _qT^{qq} + \nabla _A T^{Aq}. \end{aligned}$$

By (3.872) and (3.867)–(3.869) we have just like above

3.878 $$\begin{aligned} \nabla _pT^{pq}&= \left( \partial _p+ 2\frac{\partial _{p}\Omega }{\Omega }\right) T^{pq}, \nonumber \\ \nabla _qT^{qq}&= \left( \partial _q+ 4\frac{\partial _{q}\Omega }{\Omega }\right) T^{qq}, \end{aligned}$$

3.879 $$\begin{aligned} \nabla _A T^{Aq}&= \Gamma ^A_{pA}T^{pq} + \Gamma ^A_{qA}T^{qq} +\Gamma ^q_{AB}T^{AB} \nonumber \\&= 2\frac{\partial _{p}r}{r} T^{pq} + 2\frac{\partial _{q}r}{r} T^{qq}+ 2\eta \frac{\partial _{p}r}{r}T^{pq} \nonumber \\&= \left( 2+2\eta \right) \frac{\partial _{p}r}{r} T^{pq}+ 2\frac{\partial _{q}r}{r} T^{qq}. \end{aligned}$$

Therefore, keeping in mind equations (3.873) and (3.877) can be rewritten in the form

3.880 $$\begin{aligned} \left( \partial _p+ \partial _{p}\log \left( \Omega ^4r^2\right) \right) T^{pp} + \left( \partial _q+ \partial _{q}\log \left( \Omega ^2r^{2+2\eta }\right) \right) T^{pq}&= 0, \end{aligned}$$

3.881 $$\begin{aligned} \left( \partial _{p}+ \partial _{p}\log \left( \Omega ^2r^{2+2\eta }\right) \right) T^{pq} + \left( \partial _q+ \partial _{q}\log \left( \Omega ^4r^2\right) \right) T^{qq}&= 0, \end{aligned}$$

which is equivalent to (2.87)–(2.88). From (2.89)–(2.90) and (2.82) we have the additional relationship

3.882 $$\begin{aligned} T^{pp} T^{qq}&= (1+\varepsilon )^2 \rho ^2 (u^p)^2 (u^q)^2 = (1+\varepsilon )^2 \rho ^2\Omega ^{-4} \nonumber \\&= \left( \frac{1+\varepsilon }{1-\varepsilon }\right) ^2 (T^{pq})^2 = (1+\eta )^2(T^{pq})^2 . \end{aligned}$$

$$\square $$

### Double-Null Description of the RLP-Spacetime

#### Lemma A.1

The region to the past of the curve $\mathcal {B}_1$ in the RLP spacetime $(\mathcal {M}_{\text {RLP},\varepsilon },g_{\text {RLP},\varepsilon })$ is well-defined in the double-null coordinates, with the normalisation conditions (9.655)–(9.656).

#### Proof

Recalling the expressions (2.7) and (2.79), it is easy to see that

3.883 $$\begin{aligned} \Omega ^2 \partial _\tau p\partial _\tau q= e^{2\mu }, \ \ \Omega ^2 \partial _R p\partial _R q= -e^{2\lambda }, \ \ \frac{\partial _\tau p}{\partial _R p} + \frac{\partial _\tau q}{\partial _R q} = 0. \end{aligned}$$

Using (3.883) we conclude that $\frac{\partial _\tau p}{\partial _R p} = -\frac{\partial _\tau q}{\partial _R q} = e^{\mu -\lambda }.$ Recall that the 4-velocity $u^\nu$ in the comoving frame takes the form $e^{-\mu }\partial _\tau$ and therefore

3.884 $$\begin{aligned} \mathcal {V} = e^{-\mu }\partial _\tau r = u^p\partial _{p}r + u^q\partial _{q}r = \frac{1}{\Omega ^2 u^q}\partial _{p}r + u^q\partial _{q}r. \end{aligned}$$

It follows that $u^q$ solves the quadratic equation $\partial _{q}r (u^q)^2 - \mathcal {V} u^q+ \frac{1}{\Omega ^2}\partial _{p}r = 0$. Therefore

3.885 $$\begin{aligned} u^p&= \frac{\mathcal {V} -\sqrt{\mathcal {V}^2-\frac{4\partial _{p}r \partial _{q}r }{\Omega ^2}}}{2\partial _{p}r}= \frac{\mathcal {V} -\sqrt{\mathcal {V} ^2+1-\frac{2m}{r}}}{2\partial _{p}r}, \end{aligned}$$

3.886 $$\begin{aligned} u^q&= \frac{\mathcal {V} +\sqrt{\mathcal {V}^2-\frac{4\partial _{p}r \partial _{q}r}{\Omega ^2}}}{2\partial _{q}r} = \frac{\mathcal {V} +\sqrt{\mathcal {V}^2+1-\frac{2m}{r}}}{2\partial _{q}r}, \end{aligned}$$

where the choice of signs in the solutions of the quadratic equations is consistent with the normalisation that $e^{-\mu }\partial _\tau =u^p\partial _{p}+ u^q\partial _{q}$ is future pointing.

We now let $r\rightarrow \infty$ along the outgoing geodesic through $(\tau _0,R_0)$. By Lemma 8.8 we know that this curve asymptotes to $\mathcal {B}_1$ in the $(\tilde{\tau },R)$ plane, or equivalently, *y* converges to $y_1$ in the $(\tau ,R)$-plane. Recalling (2.21), (3.99)–(3.101), and (2.27), we see that $\mathcal {V}(\tau ,R) = \sqrt{\varepsilon }V(y)=\sqrt{\varepsilon }\tilde{r}(y) \frac{\textbf{w}(y)-1}{1+\varepsilon } \Sigma (y)^{\frac{\varepsilon }{1+\varepsilon }} \rightarrow \bar{C}_1\sqrt{\varepsilon }$ as $y\rightarrow y_1$ for some constant $\bar{C}_1>0$. Recall here that the density $\Sigma (y)=\textbf{d}(y)^{\frac{1-\varepsilon }{1+\varepsilon }}=d(Y)^{\frac{1-\varepsilon }{1+\varepsilon }}$ for $y<0$. Observe that along the outgoing geodesic

3.887 $$\begin{aligned} \lim _{q\rightarrow \infty } \left( \frac{2m}{r}-1\right) = -1+C\varepsilon \end{aligned}$$

for some $\varepsilon$-independent constant $C>0$ by an argument analogous to (8.614). Using the normalisation condition (9.656), we see that there exists a constant $\bar{C}_2>0$ such that

3.888 $$\begin{aligned} \lim _{q\rightarrow \infty } (u^q)^2=\bar{C}_2>0. \end{aligned}$$

Using the normalisation condition (9.656), equation (2.85) along the outgoing null-geodesic through $(\tilde{\tau }_0,R_0)$ takes the form

3.889 $$\begin{aligned} \partial _{q}(\Omega ^{-2})=-2\pi (1+\varepsilon )(\frac{1}{2}q+r_*) \Omega ^{-2}\rho (u^q)^{-2}, \end{aligned}$$

where we have used (2.82) and (2.82). Equivalently, after multiplying by $\Omega ^2$ and integrating, we obtain the formula

3.890 $$\begin{aligned} \partial _{q}\log \Omega = (1+\varepsilon )\pi (\frac{1}{2} q+ r_*) \rho (u^q)^{-2}. \end{aligned}$$

Note that by (2.19) $\rho (\tau ,R)=\frac{\varepsilon }{R^2}y^2 \Sigma (y)$. Since we know by Lemma 8.8 that as $q\rightarrow \infty$, $y\rightarrow y_1$, and thereby $\frac{r}{R}\rightarrow {\varvec{\chi }}(y_1)$ it follows by $q=2(r-r_*)$ that there exists a constant $C_1$ independent of $\varepsilon$ such that

$$\begin{aligned} \frac{\rho (\tau ,R)}{\frac{1}{q^2}} \rightarrow _{q\rightarrow \infty } C_1\varepsilon . \end{aligned}$$

We then use (3.888) to conclude that that the leading order asymptotic behaviour of the right-hand side of (3.890) is $\frac{C\varepsilon }{q}$ for some $\varepsilon$-independent constant $C>0$. Therefore $\Omega (p_0,\cdot )$ is well-defined and $\Omega \sim _{q\rightarrow \infty }q^{C\varepsilon }$. Moreover, from (9.667) and (3.887), we have

3.891 $$\begin{aligned} \lim _{q\rightarrow \infty } \frac{4\partial _{p}r\partial _{q}r}{\Omega ^2} = \lim _{q\rightarrow \infty } \frac{2m}{r}-1 = -1+C\varepsilon . \end{aligned}$$

It follows therefore, that along the outgoing geodesic $\partial _{p}r\approx _{q\rightarrow \infty } \Omega ^2\approx _{q\rightarrow \infty } q^{2C\varepsilon }$. We can similarly use the normalisation condition (9.655) to determine $\Omega$ along the ingoing geodesic through $(\tilde{\tau }_0,R_0)$ in the past of $\mathcal {B}_1$. Using (2.83)–(2.84), it is straightforward to show that the the solution exists everywhere in the past of $\mathcal {B}_1$ with the asymptotic behaviour $\Omega \asymp _{q\rightarrow \infty } q^{C\varepsilon }$, $\partial _{p}r \asymp _{q\rightarrow \infty } q^{2C\varepsilon }$, $\partial _{q}r\asymp _{q\rightarrow \infty } 1$. $\square$

## Footnotes

1 For two non-vanishing functions $x\mapsto A(x)$, $x\mapsto B(x)$ we write $A\asymp _{x\rightarrow \bar{x}} B$ to mean that there exist $\varepsilon$-independent constants $C_1, C_2>0$ such that

$$\begin{aligned} C_1 \le \liminf _{x\rightarrow \bar{x}} \frac{A(x)}{B(x)} \le \limsup _{x\rightarrow \bar{x}} \frac{A(x)}{B(x)} \le C_2. \end{aligned}$$

2 The LP- and Hunter-type solutions coincide at $x_*=2$.

3 The nomenclature is motivated by their Newtonian analogue discovered by Larson [20] and Penston [31] in 1969, see [15] for the rigorous proof of existence of the Newtonian solution.

4 A geodesic $\gamma ^\nu$ is parametrised by an affine parameter $\ell$ if $\ddot{\gamma }^{\nu }(\ell )+\Gamma ^\nu _{\alpha \beta }\dot{\gamma }^\alpha (\ell )\dot{\gamma }^\beta (\ell )=0$.
